# Heavier tetrylene- and tetrylyne-transition metal chemistry: it's no carbon copy

**DOI:** 10.1039/d3cs00226h

**Published:** 2024-09-04

**Authors:** Terrance J. Hadlington

**Affiliations:** a Fakultät für Chemie, Technische Universität München, Lichtenbergstraße 4 85748 Garching bei München Germany terrance.hadilngton@tum.de

## Abstract

Since the late 19th century, heavier tetrylene- and tetrylyne-transition metal chemistry has formed an important cornerstone in both main-group and organometallic chemistry alike. Driven by the success of carbene systems, significant efforts have gone towards the thorough understanding of the heavier group 14 derivatives, with examples now known from across the d-block. This now leads towards applications in cooperative bond activation, and moves ultimately towards well-defined catalytic systems. This review aims to summarise this vast field, from initial discoveries of tetrylene and tetrylyne complexes, to the most recent developments in reactivity and catalysis, as a platform to the future of this exciting, blossoming field.

## Abstract 2

Developments in synthetic methods for and reactivity of heavier tetrylene and tetrylyne complexes of the d-block elements are summarised, with a view towards cooperative bond activation and utilisation in catalysis.

## Graphical abstract

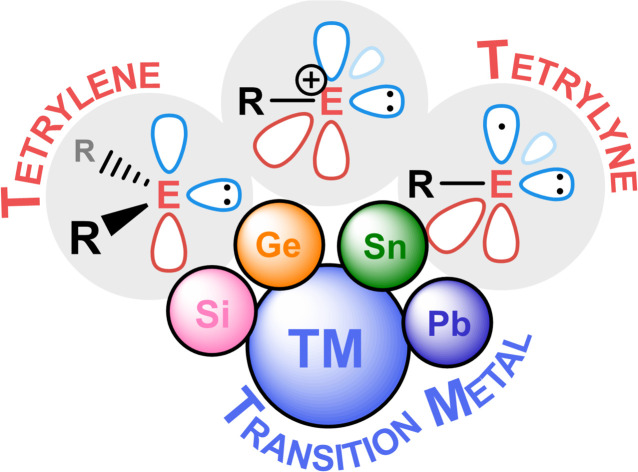

## 1. Introduction

The discovery and development of organometallic tranisition metal (TM) chemistry has been formative in guiding modern synthesis, forming the basis for the (catalytic) transformation of hydrocarbon building blocks. Amongst these key developments, formally carbon(ii) and carbon(i) species have been of a great importance. Low oxidation state organometallics, such as carbene-,^1–6^ and carbyne-complexes^7–10^ have laid the fundamental ground-work for indispensible reactive processes such as alkene and alkyne metathesis.^11–15^ Stable carbene ligands have also come to be ubiquitous in countless facets of chemical synthesis, from powerful ligands in catalytic transition metal systems,^16,17^ to stabilising ligands for exotic main group species.^18^ It is therefore not surprising that interest in the related chemistry of the heavier group 14 elements has also been fervent throughout the past 50 years, in the pursuit of direct comparisons with the lightest member of the group. This has taught us that the heavier elements behave rather differently to carbon, increasingly favouring the +2 oxidation state, and forming gradually weaker, even insignificant π-interactions.^19–21^ Such observations directly relate to the HOMO–LUMO separation in these systems, leading a reduced sp-hybridsation on descending the group, and a reduced tendency for the valence s-electrons to partake in bonding. In some ‘extreme’ cases this is clearly borne out by the bonding and molecular structure in TM complexes: metallotetrylenes are common for Ge–Pb, but essentially unknown for carbon; stannylenes and plumbylenes are known to behave as Z-type ligands, which is not the case for carbenes. The potential utility of these electronic differences is beginning to be exploted in bond activation and catalysis, whereby low oxidation state tetryl ligands operate in concert with a TM centre in bond scission and group transfer. We therefore envisage this as a timely review in summarising the methods typically employed in accessing such TM complexes, key aspect of their bonding and electronic nature, and ultimately their reactive capacity moving towards applications in catalysis.

This review aims to summarise the broad field of tetrylene- and tetrylyne-transition metal chemistry, focusing largely on (i) structurally characterised systems, and (ii) systems with an unsaturated tetryl centre. The main exception in the latter case regards the amidinato-stabilised tetrylene systems which have seen significant attention.††This therefore negates a broad survey of base-stabilised systems, metal-bridging tetrylenes, cluster compounds, and related compound classes with a higher coordinate tetryl centre. In introducing the early break-throughs regarding this field, base-stabilised examples will be included for clarity and completeness. On discussing tetrylyne complexes, their tautomeric form, *i.e.* metallotetrylenes, will also be considered. On the whole, then, this review considers monoatomic heavier group 14 element ligands in the oxidation states of +2 and +1, formally speaking. Where pertinent, the reactivity of the E-TM moiety in these complexes will be described, in view of the utility of such linkages to affect cooperative bond activation (*vide infra*). To the best of our knowledge, a comprehensive and accessible review of this burgeoning selection of research area is lacking, with a few more focused pieces being published in the past 15 years.^22–27^ We thus aim to bench-mark the standing of these intrinsically related fields, and particularly identify key aspects for the targeted design of group 14 – TM systems which can achieve the (reversible) activation of challenging small molecules, for applications in catalysis using these Earth-abundant elements.

## 2. Bond activation at the low-oxidation state group 14 – transition metal interface

**Fig. 1:**
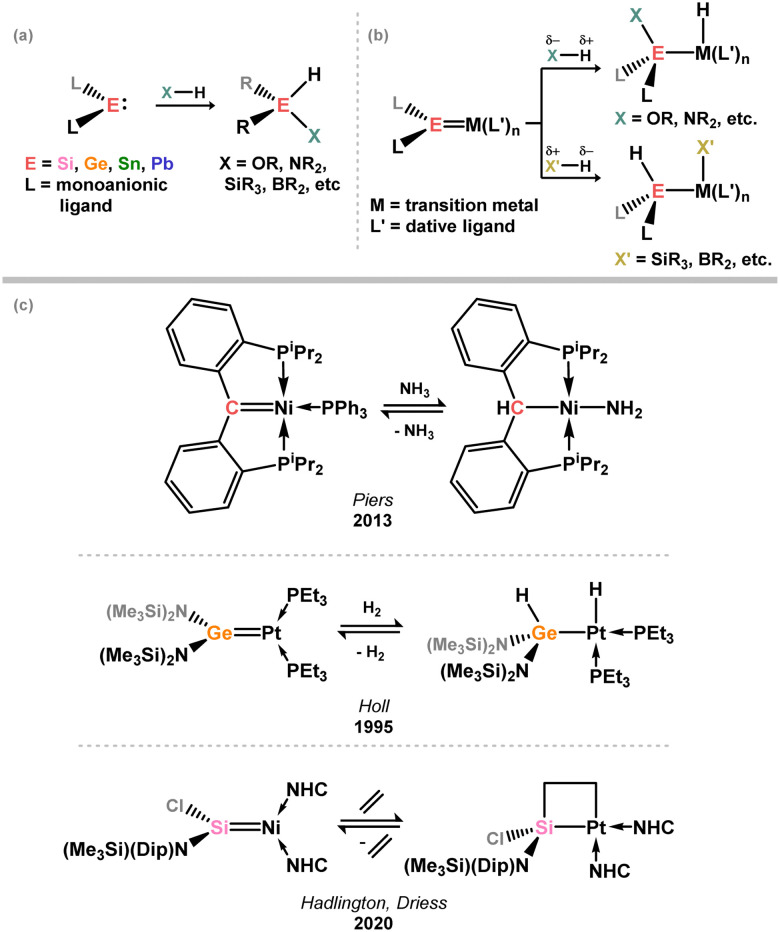
(a) Oxidative processes of tetrylenes; (b) the typical and/or expected addition products in heavier tetrylene transition metal complexes; (c) examples of reversible bond activation at the group 14 – transition metal interface.

Uncovering chemical reactivity in the main group which mimics that of the TMs has seen significant attention since the identification of systems which can activate catalytically relevant molecules such as dihydrogen. The key point in pursuing this direction lies in displacing our dependence on low-abundance, expensive, and toxic heavier TMs, particularly the Noble metals. Although molecular group 14 systems are now known which effect the activation of numerous small molecules (*i.e*. H_2_,^28,29^ R_3_SiH,^30^ R_2_BH,^30,31^ NH_3_,^32,33^ C_6_H_6_,^34^ C_2_H_4_,^35,36^ CO,^37–39^ CO_2_;^40,41^*e.g.*Fig. 1(a)),^42–44^ reversible activation remains quite rare. A single group 14 system which can affect more than one or two elementary steps of a redox-active catalytic cycle (*viz.* oxidative addition/insertion/reductive elimination) is also essentially unknown. Still, tetrylenes and ditetrylynes are capable of activing substrates which are particularly challenging for TMs, a key example being NH_3_.^32,33^ In this regard, non-innocent carbene ligands have shown the capacity to enable the reversible activation of NH_3_ in combination with Ni,^45,46^ whilst a number of heavier tetrylene–TM systems have demonstrated the reversible addition of *e.g.* H_2_ and alkenes (Fig. 1(c)).^47–49^ Conceptually, then, the utility of tetrylenes in modulating the energetics of bond activation in TM complexes, and indeed in enabling otherwise inaccessible bond activation processes, is powerful. This notion was addressed in a recent review from Campos and co-workers.^27^ An additional key point here is the typical polarity of an E–TM bond (E = Si–Pb): given that these elements are significantly more electropositive than C, one expects the tetryl centre to be electrophilic. This leads to an Umpolong of protic substrates upon activation, and would also lead to reactive metal-boryl and -silyl systems upon activation of hydridic boranes and silanes (Fig. 1(b)). Finally, given the ease of access to the +2 oxidation state for the heavier group 14 elements, one may even argue that a broad range of tetrylene ligands featuring diverse electronic and steric properties is more accessible than carbenes, particularly suited to the development of non-π-stabilised, acyclic derivatives, which are expected to play a more active role in bond activation.

## 3. Heavier tetrylene systems

**Fig. 2:**
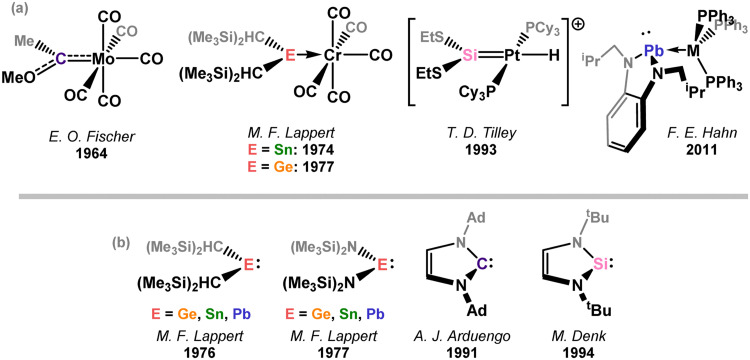
(a) The first example of a carbene-TM and heavier tetrylene–TM complexes; (b) the historical occurance of persistent tetrylenes for all group 14 elements.

Carbene chemistry has been formative in the contemporary field of MG – TM organometallics, fuelled by the first discoveries of stable TM–carbene complexes, and later persistent carbenes (*e.g.* NHCs, cAACs). It is therefore not surprising that heavier congeners of TM–carbene complexes have been thoroughly investigated, the initial report of structurally characterised derivative species appearing in 1974 (Fig. 2(a)). This complex was accessed through combination of a persistent stannylene with [THF·Cr(CO)_5_],^50^ which was later extended to Ge, demonstrating for the first time the ease of access to this compound class.^51^ It was some 20 years later that Tilley and co-workers accessed the first example of a base-free silylene complex,^52^ and a further 20 years until the intial example of a plumbylene derivative.^53^ Still, persistent germylenes, stannylenes, and plumbylenes were reported by Lappert and co-workers from 1973,^54–56^ earlier than their persistent carbon congeners, which were not forthcoming until some time later, in 1991.^57^ Notably, the first persistent silylenes were not forthcoming until 1994,^58^ attesting to the high reactivity of Si^II^ when compared to the heavier divalent species of group 14. Aside from the addition of persistent tetrylenes to a TM fragment, heavier tetrylene–TM complexes have been accessed through various additional methods, such as tetrylene transfer (*e.g.* from tetrylene adducts), and tetrylane (*i.e.* R_*n*_EH_4−*n*_; *n* = 1–3) activation, the latter having implications in hydrotetrylation catalyses.^59,60^ A great number of tetrylene–TM complexes have been approached from the fundamental synthetic level, giving key insights into their bonding and electronic nature. More recently, cooperative bond activation and catalysis have also become a key focal point.^27,61^ Indeed, it has even been suggested that silylenes and germylenes may perform better than widely employed carbenes in catalytic regimes,^62^ a point which still requires considerable research effort to affirm.

**Fig. 3:**
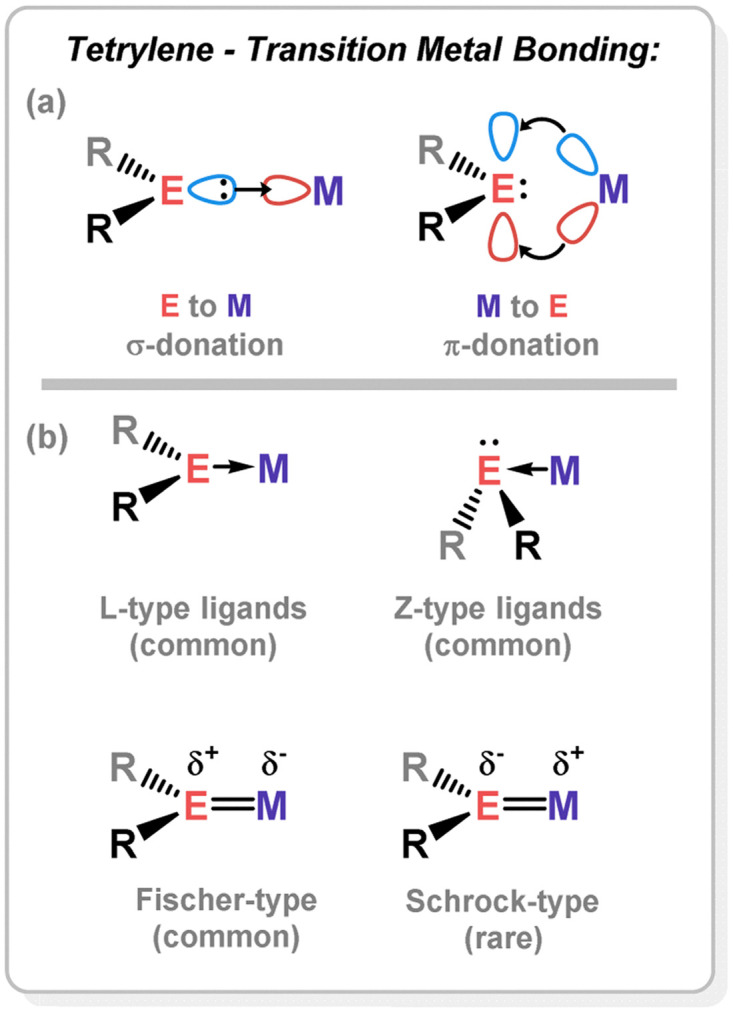
(a) Typical dative bonding model in tetrylene–TM complexes; (b) some known binding modes in tetrylene–TM complexes.

The bonding nature of the heavier tetrylenes towards a transition metal differs from that of carbon due in large to the decrease in sp-hybridisation for the heavier homologues, and the concurrent increased HOMO–LUMO separation. In the vast majority of cases, tetrylenes are expected to behave as σ-donor ligands, with varying degres of π-acceptor character (Fig. 3(a)), greatly affected by the substituents at E (E = Si–Pb). The tetryl centre remains electrophilic in most cases, again being greatly affected by the substituents at E, and indeed by the electronic nature of the bound metal. In extreme cases, such as in binding to highly electropositive early TMs, a nucleophilic tetryl centre is formed, which relates to classic Schrock carbenes (Fig. 3(b)). A decreased propensity of the s-character electron pair at the tetrylene centre to partake in bonding interactions is observed on decending the group, leading to a pronounced change in bonding charactersitics, for example, in plumbylene complexes, whereby a Z-type binding is observed as opposed to the expected L-type bonding.

There are numerous classes of tetrylene ligand, which can be collected as N-heterocyclic tetrylenes (NHTets), further cyclic derivatives, and acyclic systems, the former being by far the most common, and thus the most thoroughly studied in the context of TM coordination chemistry. A number of bis(tetrylene) ligands have also been reported, which are particularly effective chelating ligands in catalytic systems. For clarity, reported TM complexes described here will be approached group-by-group, from group 3 to 12. Further, aside from the broadly applied amidinato-tetrylenes and a small number of select examples, we will not discuss base-stabilised tetrylene–TM complexes, given that their valency lies closer to formal tetryl than to tetrylene complexes. Historically, the discovery of base-free (*i.e.* two-coordinate) tetrylenes in the coordination sphere of a TM was a key step forward in developing a thorough understanding of the bonding and reactivity of these heavier carbene complexes. Access to base-stabilised derivatives saw significant developments in the late 1980s,^63–68^ and paved the way to lower coordinate systems which are the focus of the following discussion. These systems compare to broadly applied Schrock, Fischer, and N-heterocyclic carbenes, for example, and hence their utility and/or presence in synthetic processes, and their applications as spectator, and now even non-innocent ligands in bond activation and catalysis is of significant interest. This is particularly true given their ready variability, and ready access to low-oxidation state starting materials, which is not the case for lightest member of group 14.

### 3.1. Silylene – transition metal chemistry

#### 3.1.1. N-heterocyclic silylenes

**Fig. 4:**
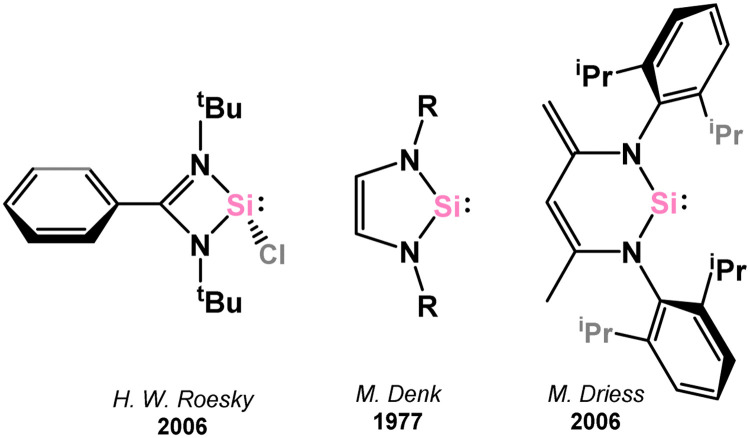
Examples of 4-, 5-, and 6-membered N-heterocyclic silylenes.

Through π-donation and conjugation, N-heterocyclic systems are highly effective in stabilising tetrylenes, and as such this class of tetrylene–TM complex is the most abundant in the known literature. These can be further separated into 4-, 5-, and 6-membered NHTets. The 4- and 6-membered species typically feature the monoanionic amidinate and Nacnac ligands, respectively, whilst 5-membered species are typically built upon the well-known diazabutadiene scaffold, which has been so successful in NHC development. N-heterocyclic silylenes (NHSis) are arguably the most prominent of the NHTets, when taking into account chelating derivatives, as demonstrated by this section, with key examples of 4-, 5-, and 6-membered systems shown in Fig. 4.^58,69,70^

##### 4-Membered

**Scheme 1:**
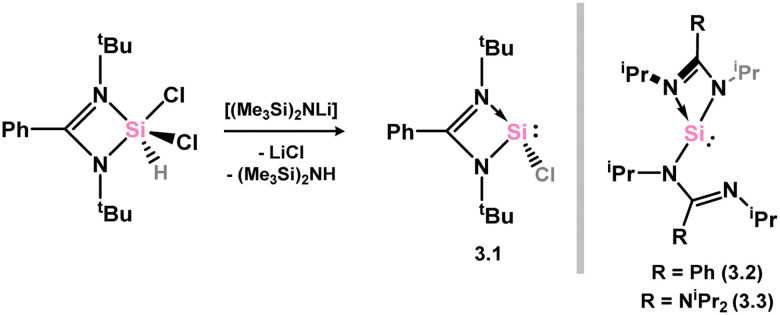
Synthesis of the so-called Roesky silylene, and related systems.

As mentioned, the vast majority of 4-membered NHTets are based upon the monoanionic amidinate scaffold. The divalent group 14 element can therefore accommodate a second substituent, which has led to an expansive array of these ligands. For Si (*i.e.* NHSis), this was initiated by the reproducible and scalable synthesis of the so-called Roesky silylene (*viz.*3.1, Scheme 1),^71^ whereby the chloride substituent at the Si centre can be exchanged for any number of organyl fragments. The breadth of readily available ligands accessible *via* this route will not be discussed in depth here, but a synthetic route to the chloro silylene is given in Scheme 1. Direct access to an additional bis(amidinato)silylene is also known (3.2),^72^ as is the closely related synthesis of a bis(guanidinato)silylene (3.3).^73^

**Scheme 2:**
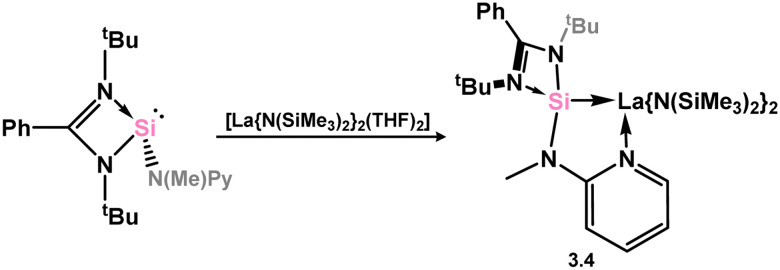
Accessing an (amido)lanthanum(ii) complex supported by a pyiridine-functionalised silylene.

In the category of 4-membered NHSis, only one example of a group 3 complex is known (3.4, Scheme 2), featuring a chelating pyridine-functionalised silylene ligand. This species is accessed through simple combination of the free silylene ligand with [Y{N(SiHMe_2_)_2_}_2_(THF)_2_].^74^

**Scheme 3:**
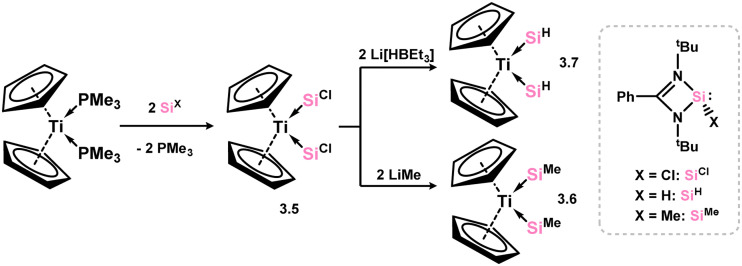
The synthetic route to bis(amidinato)silylene titanium(ii) complexes.

No group 3 complexes are known featuring NHSis ligands. Three complexes are known from group 4, both with the [Cp_2_Ti] unit. Here, the bis(phosphine) complex [Cp_2_Ti(PMe_3_)_2_] was reacted with the chloro silylene 3.1, leading to phosphine ligand exchange in forming 3.5 (Scheme 3).^75^ The chloro–silylene complex undergoes salt-metathesis reactions at the Si–Cl moiety, in forming bis(methyl-silylene) and bis(hydrido-silylene) complexes 3.6 and 3.7.

**Scheme 4:**
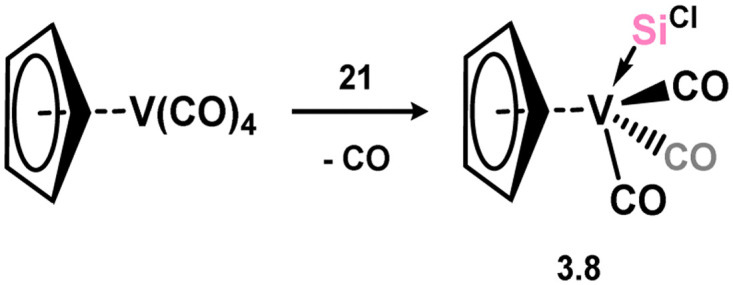
Synthesis of the sole example of a vanadium(i) amidinato–silylene complex.

One group 5 complex is reported, accessible through combination of chloro–silylene 3.1 with [CpV(CO)_4_], leading to substitution of one carbonyl ligand in forming 3.8 (Scheme 4).^76^

**Scheme 5:**
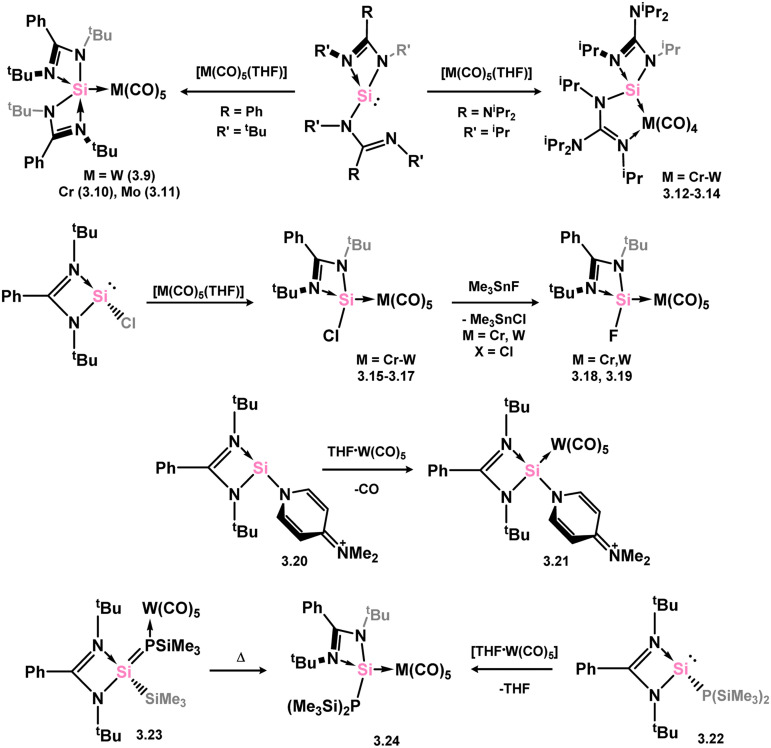
Synthetic access to a range of amidinato–silylene chromium, molybdenum, and tungsten carbonyl complexes.

A number of group 6 complexes featuring 4-membered NHSis are known (Scheme 5). The first was reported in 2012, featuring bis(amidinato)silylene 3.2, which reacts with [W(CO)_6_] in exchange of one carbonyl ligand, forming 3.9. Closely related complexes of Cr and Mo were later accessed (3.10 and 3.11).^77^ Interestingly, the bis(guanidinato) NHSi derivative 3.3 led to chelating(imino-silylene) complexes of [M(CO)_4_] fragments when employed in the same reaction (*viz.*3.12, 3.13, and 3.14), highlighting key differences between the amidinate and guanidinate ligand scaffolds.^78^ [M(CO)_5_] complexes of chloro–silylene 3.1 (*viz.*3.15, 3.16, and 3.17) proved to be suitable precursors for the formation of rare examples of fluoro-silylene systems 3.18 and 3.19, through reaction of the former complexes with Me_3_SnF.^79^ A formally cationic-silylene complex of [W(CO)_5_] is known, utilising DMAP-bound (DMAP = *N,N*-dimethylaminopyridine) cationic silylene 3.20, forming 3.21. This product is thus also additionally base-stabilised at Si with DMAP, accessed utilising the free silylene.^80^ A phosphino–silylene (*viz.*3.22) complex of [W(CO)_5_] is known, which can interestingly be accessed through thermal rearrangement of the W@P silaphosphene complex 3.23, leading to 3.24.^81^ A unique chelating silylene complex of [Mo(CO)_4_] can be accessed through addition of [Mo(CO)_6_] to bis(silylene) 3.25, so forming 3.26.^82^ The same species is also generated by combination of the free chelating silylene ligand, which forms *via* CO activation by 3.25, to irradiated [W(CO)_6_] (*vide infra*).

**Scheme 6:**
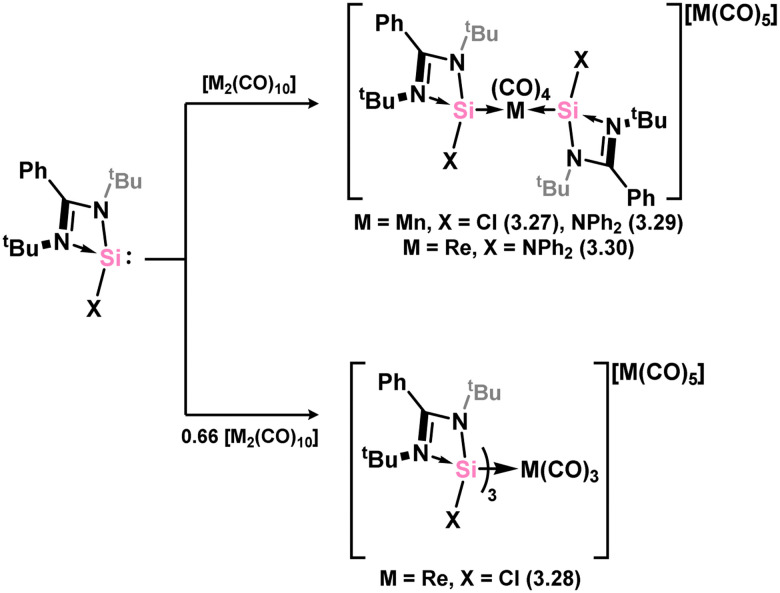
Access to the rhenium and manganese complexes featuring amidinato–silylene ligands.

**Scheme 7:**
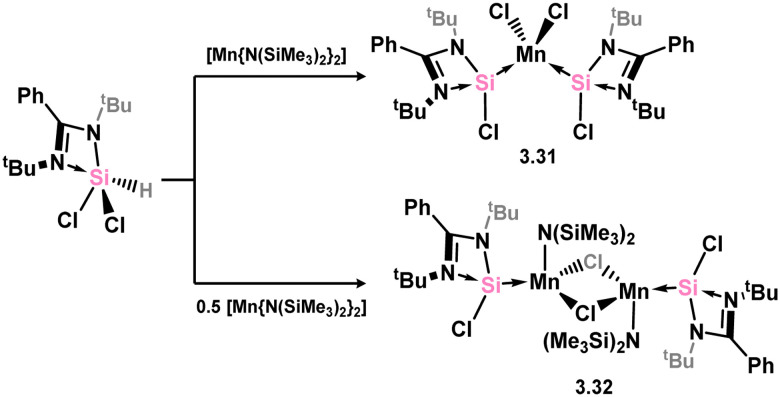
Formal reduction of silanes by manganese(ii), forming silylene complexes.

A number of Mn and Re complexes featuring 4-membered NHSis are known (Scheme 6). Cationic [Mn(CO)_4_] and [Re(CO)_3_] complexes, bearing two (*viz.*3.27) and three chloro–silylene ligands (*viz.*3.28), respectively, are isolated from stoichiometric mixtures of the [M(CO)_6_] (M = Mn, Re) complexes and silylene ligand 3.1.^83^ Utilising a slightly modified silylene led instead to cationic complexes bearing two silylene ligands at the [M(CO)_4_]^+^ centres for both Mn and Re (3.29 and 3.30).^84^ A novel approach to the formation of an (amido)(chloro)manganese(ii) silylene complex was recently reported, in which the bis(amido)manganese(ii) species, [{(Me_3_Si)N}_2_Mn], formally reduces the silicon centre through amine elimination, and is complexed by the resultant silylene forming complexes 3.31 and 3.32, depending on stoichiometry (Scheme 7).^85^ This methodology was also extended to iron and cobalt (*vide infra*).

**Scheme 8:**
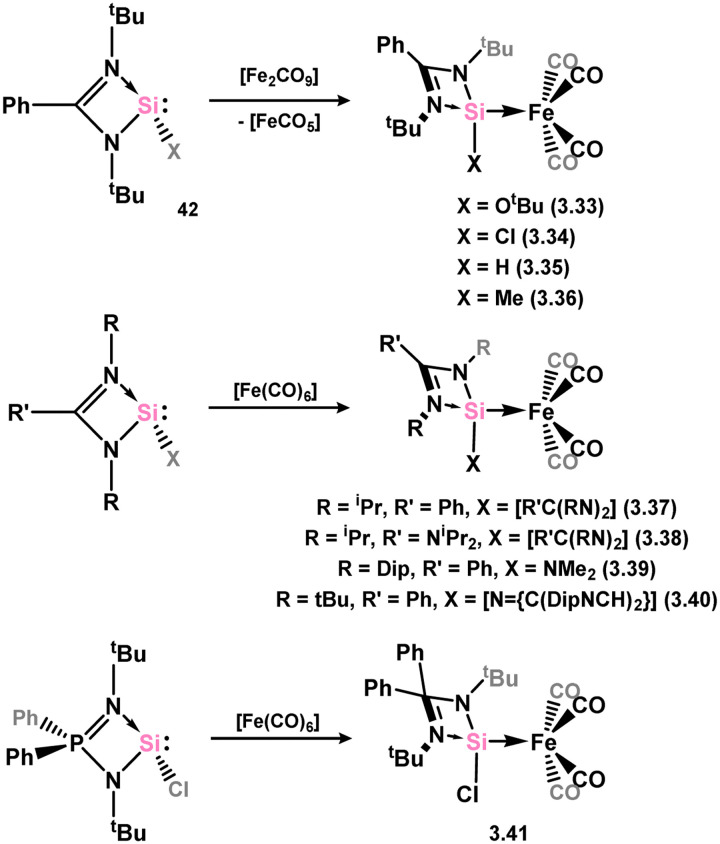
Access to a broad range of iron(0) tetracarbonyl complexes bearing amidinato–silylene ligands.

**Scheme 9:**
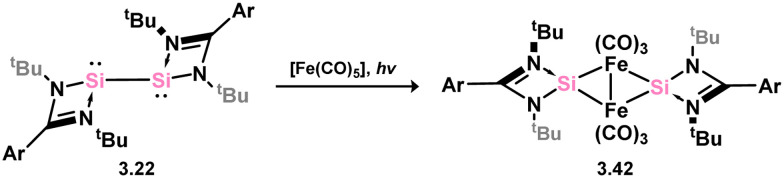
Reactivity of an amidinato-stabilise bis-silylene towards iron(0) pentacarbonyl.

Many 4-membered NHSi adducts of the [Fe(CO)_4_] fragment are known, typically synthesised to demonstrate the basicity of the Si centre, and accessed through the addition of the free silylene ligand to [Fe(CO)_5_] or [Fe_2_(CO)_9_] (Scheme 8), or dihalosilanes with [K_2_Fe(CO)_4_].^86^ The initial study utilised an (amidinato)(*tert*-butoxy)silylene, in reaction with [Fe_2_(CO)_9_]. This resulted in the displacement of [Fe(CO)_5_] and the generation of complex 3.33.^87^ Similar reactivity is observed, or with the closely related [Fe(CO)_6_], for (amidinato)(chloro)- (3.34), (amidinato)(hydrido)- (3.35), (amidinato)(methyl)- (3.36), bis(amidinato)- (3.37), bis(guanidinato)- (3.38), (amidinato)(amido)- (3.39), (amidinato)(N-heterocyclicamino)- (3.40), and (iminophosphonamido)-silylenes (3.41), which readily form [Fe(CO)_4_] adducts on reaction with [Fe(CO)_5_].^77,78,88–90^ Somewhat related, bis(silylene) 3.22 reacts with [Fe(CO)_5_] in the formation of [Fe_2_(CO)_6_] complex 3.42 (Scheme 9).^82^

**Scheme 10:**
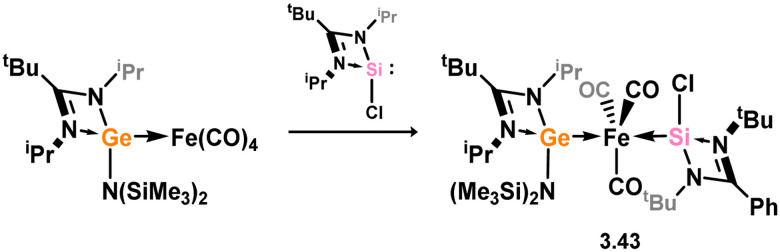
The synthetic route to mixed germylene–silylene complexes of iron(0).

Mixed germylene–silylene complexes of [Fe(CO)_3_] have been reported as effective precursors for iron germanide nanoparticles, accessed through the addition of silylene 3.1 to a germylene-[Fe(CO)_4_] adduct (*vide infra*), leading to heteroleptic complex 3.43 (Scheme 10).^91^

**Scheme 11:**
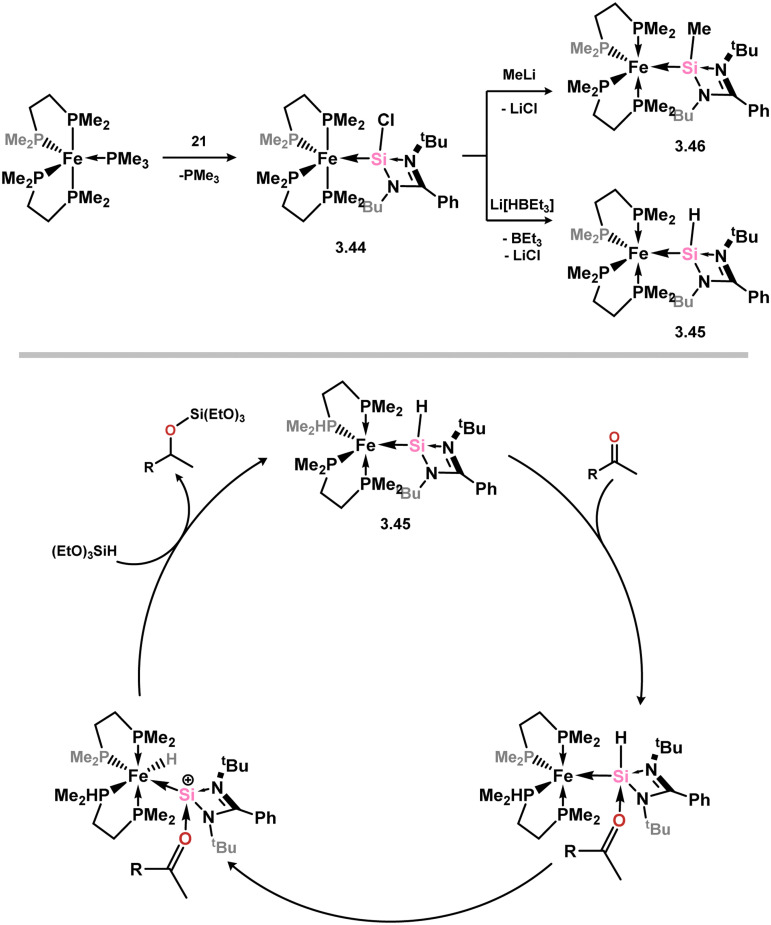
Access to a range of dmpe-stabilised iron(0) complexes bearing amidinato–silylene ligands (above), and the postulated outer sphere mechanism for ketone hydrosilylation catalysis utilising 3.45.

Subsequent investigations revealed that the introduction of chloro–silylene 3.1 to the Fe^0^ complex [(dmpe)_2_Fe(PMe_3_)] leads to PMe_3_ substitution, leading to the formation of complex 3.44 (Scheme 11).^92^ Similar to the behavior observed with Ti^II^ complex 3.5, the chloride ligand in 3.44 could undergo substitution with hydride or methyl ligands, yielding complexes 3.45 and 3.46, respectively. Furthermore, complex 3.45, containing a hydride ligand, demonstrated catalytic activity in the hydrosilylation of ketones. This activity was attributed to an outer-sphere mechanism where the ketone coordinates to the Si^II^ prior to H-transfer facilitated by the Fe^0^ center (Scheme 11, below).

**Scheme 12:**
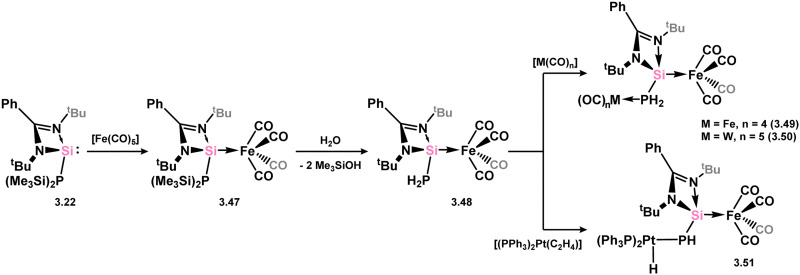
Access to phosphide–silylene iron(0) complexes, and subsequent synthesis of hetero-bimetallic complexes through P-coordination.

The phosphine-modified silylene 3.22, as per the many examples above, readily displaces on CO ligand in [Fe(CO)_5_], resulting in the formation of complex 3.47, which upon hydrolysis, yielded the primary-phosphine complex 3.48 (Scheme 12).^93^ Subsequent interactions of 3.48 with [Fe_2_(CO)_9_] or [THF·W(CO)_5_] led to the formation of homo- and hetero-bimetallic complexes 3.49 and 3.50, facilitated by P → M donation (M = Fe or W). Furthermore, reaction with [(C_2_H_4_)Pt(PPh_3_)_2_] resulted in the formation of the Pt^II^-phosphide complex 3.51*via* insertion into one P–H bond of the PH_2_ moiety.

**Scheme 13:**
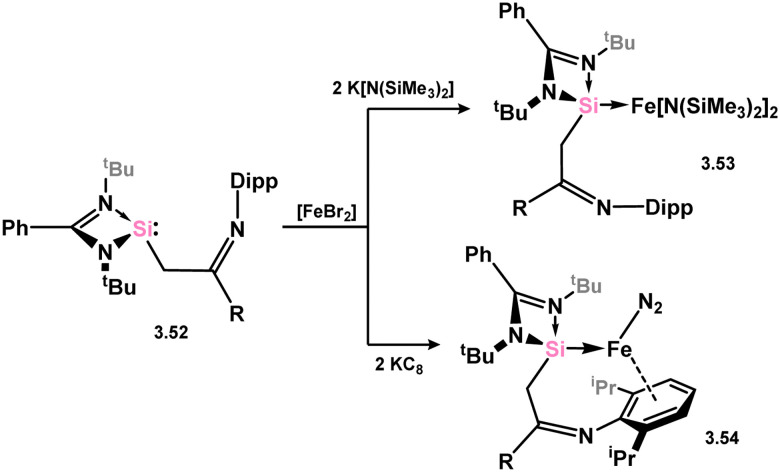
Synthesis of iron(0) and iron(ii) complexes bearing an imine-functionalise amidinato–silylene.

A novel imine-functionalised silylene, 3.52, has been developed and demonstrated its ability to stabilise Fe^0^ in complex 3.53, coordinated with both arene and nitrogen ligands (Scheme 13). This complex is accessed by reducing the [FeBr_2_] precursor ligated with silylene using KC_8_.^94^ Interestingly, under an argon atmosphere, the nitrogen ligand dissociates, while under N_2_, complex 3.54 exhibits catalytic activity in the reductive silylation of N_2_ to (Me_3_Si)_3_N, utilising the KC_8_/Me_3_SiCl couple, generating up to 47 equiv. of (Me_3_Si)_3_N, comparing favourably with reported systems which can achieve this impressive N_2_ functionalisation process.^95^ Additionally, the bis(amido)-Fe^II^ complex 3.54 can be synthesised by initially reacting silylene 3.52 with [FeBr_2_], followed by K[N(SiMe_3_)_2_].

**Scheme 14:**
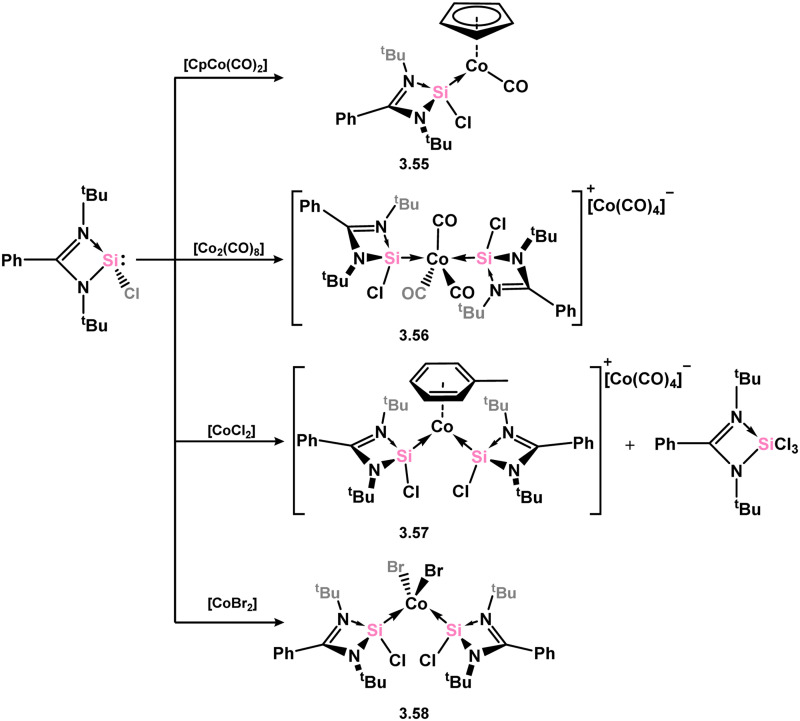
Access to cobalt(i) complexes bearing the Roesky silylene.

A number of cobalt complexes bearing silylenes derived from 3.1 are known, as are a small number of rhodium and iridium species. The first cobalt example, 3.55, was accessed in a straight-forward manner through the addition of 3.1 to [CpCo(CO)_2_] (Scheme 14).^76^ It was further shown that the similar reaction involving [Co_2_(CO)_8_] led to the cationic bis-silylene complex 3.56, bearing the [Co(CO)_4_]^−^ counter anion. It was later reported that addition of 3.1 to [CoCl_2_] in the ratio of 5 : 4 led to partial reduction of cobalt, in forming the cationic complex 3.57, in which the central cobalt is in the +1 oxidation state.^96^ The similar reaction with [CoBr_2_] circumvented the reductive process, leading to bis-silylene complex 3.58.

**Scheme 15:**
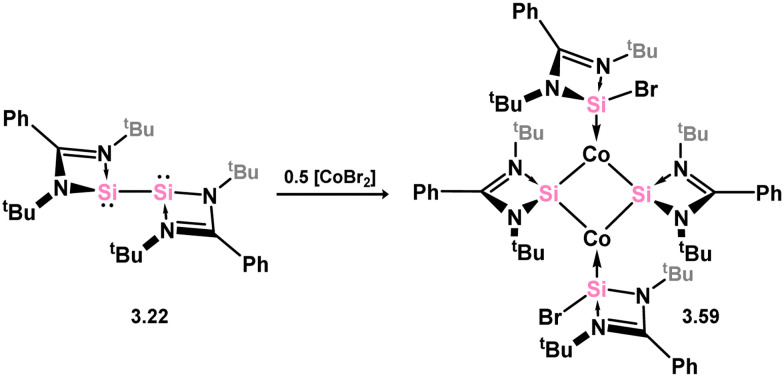
Reactivity of an amidinato-stabilised bis-silylene towards cobalt(ii) bromide.

**Scheme 16:**
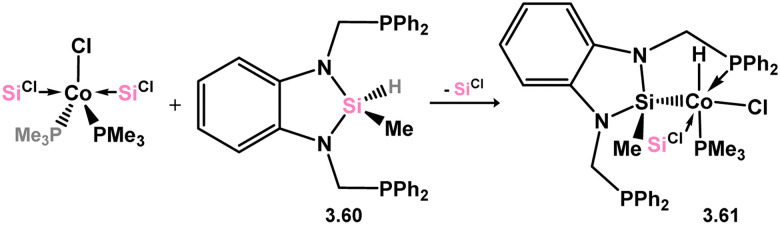
Synthesis of a pincer-silyl complex of cobalt(iii), additionally supported by an amidinato–silylene.

The reaction between cobalt(ii) bromide and bis(silylene) 3.22 resulted in a reductive insertion reaction, leading to the formation of tetrameric 3.59.^97^ This complex can be described as having two bromo-silylene fragments and two cobalto-silylene fragments (Scheme 15). This particular compound exhibited catalytic activity in the C–H functionalisation of arylpyridines with alkynes. Another instance of cobalt catalysis coordinated by a silylene was subsequently documented, employing complex 3.60, which was obtained through the addition of a pincer silyl-ligand to a silylene-ligated Co^I^ complex (Scheme 16).^98^ Complex 3.61, containing a Co^III^ center, demonstrated the ability to catalyse the Kumada coupling of aryl Grignard reagents with mono- and di-chloroarenes, as well as bromoarenes, displaying a moderate tolerance towards various functional groups.

**Scheme 17:**
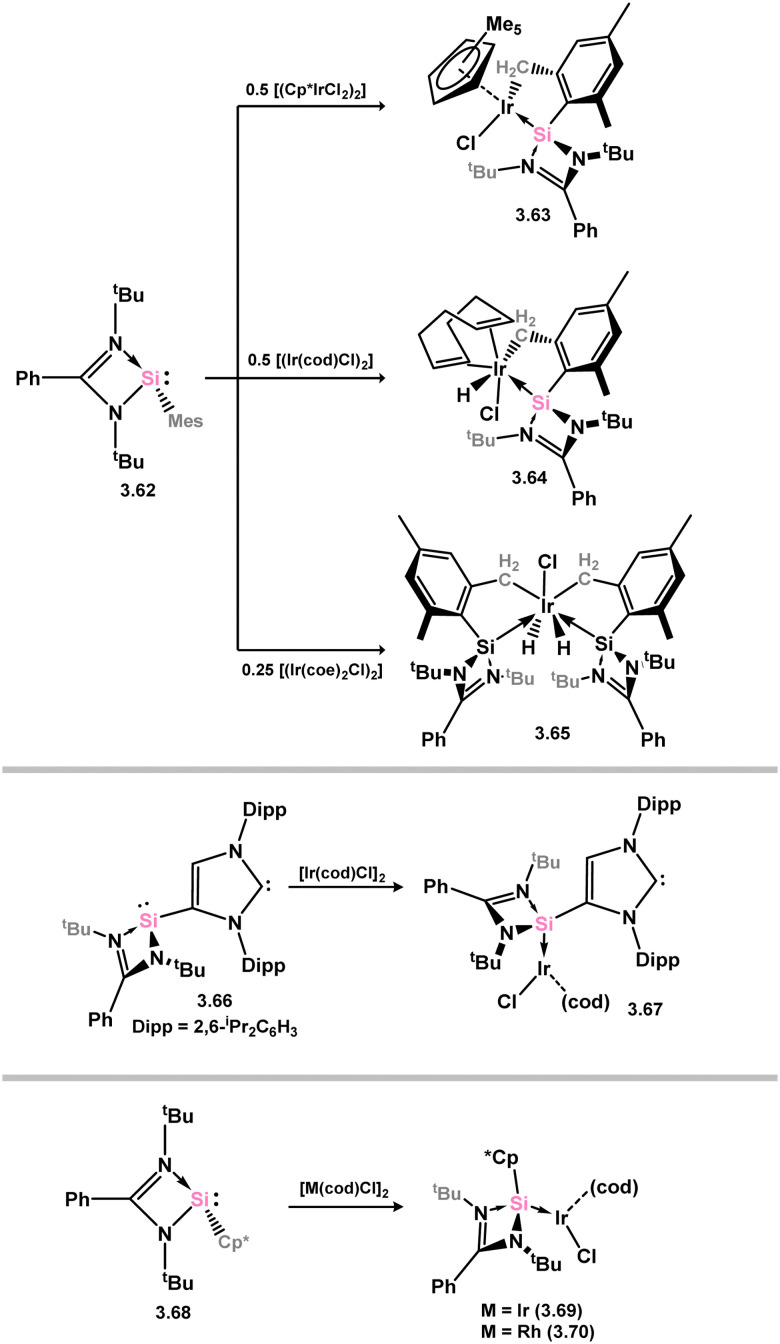
Synthesis of iridum complexes bearing amidinato–silylene ligands.

A variety of iridium complexes were synthesised through C–H activation on the mesityl group in mesityl-silylene 3.62. The iridium sources used were [(Cp*IrCl_2_)_2_], [{(cod)IrCl}_2_], or [{(coe)_2_IrCl}_2_] (cod = 1,5-cyclooctadiene; coe = cyclooctene), resulting in the formation of complexes 3.63, 3.64, and 3.65, respectively (Scheme 17).^99^ Selective coordination of Ir^I^ by the Si^II^ center was observed when silylene-functionalised NHC 3.66 reacted with [{(cod)IrCl}_2_], leading to the formation of complex 3.67.^100^ In a similar manner, silylene 3.68, substituted with Cp*, was found to coordinate Ir^I^ and Rh^I^ upon reaction with [{(cod)MCl}_2_] (M = Ir, Rh), resulting in the formation of 3.69 and 3.70.^101^

**Scheme 18:**
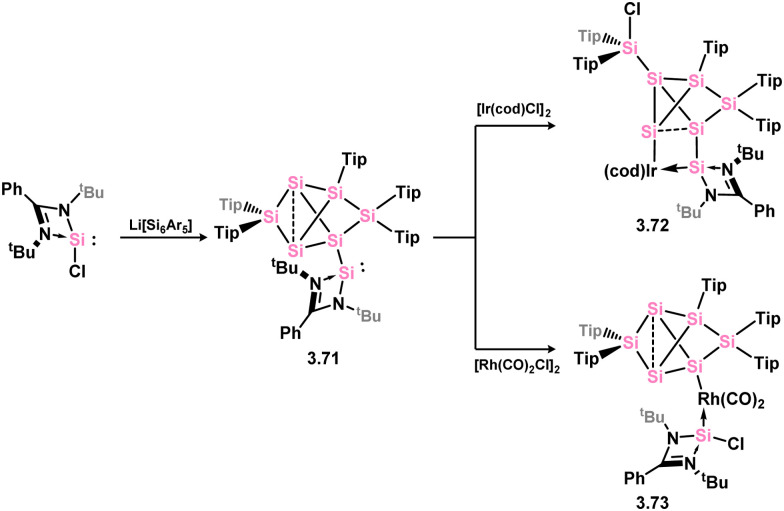
Activation of silylene-appended Si_6_ cluster by rhodium(i) and iridium(i).

In somewhat related chemistry, the silylene-functionalised siliconoid species 3.71 reacted with M^I^ complexes [{(cod)IrCl}_2_] and [{Rh(CO)_2_Cl}_2_], leading to M–Cl activation in forming complexes 3.72 and 3.73 (Scheme 18).^102^ These are each rather interesting examples of the selective activation of a silicon cluster by a TM, and is presumably made feasible by the presence of the functional (amidinato)silylene moiety.

**Scheme 19:**
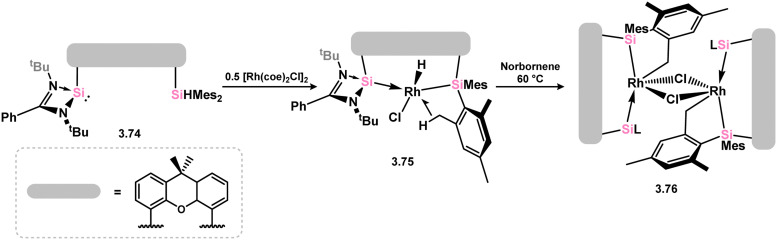
The addition of a chelating silyl-silylene ligand to rhodium(i), leading to rhodium(iii) species.

A more intricate scenario was observed when the silane-functionalised silylene 3.74 was reacted with [(coe)_2_RhCl]_2_, resulting in silylene coordination and activation of the Si–H bond, forming rhodium hydride complex 3.75, in which an agostic C–H⋯Rh interaction is formed with one mesityl-Me group (Scheme 19).^103^ This complex undergoes a dehydrogenative C–H activation of one mesityl-Me group in the presence of norbornene, which results in the formation of dimeric complex 3.76. Both complexes 3.75 and 3.76 displayed catalytic activity in the C–H alkylation of 2-phenylpyridine with norbornene, surpassing the activity of related phosphine, NHC, or bis(NHSi) Rhodium complexes.

**Scheme 20:**
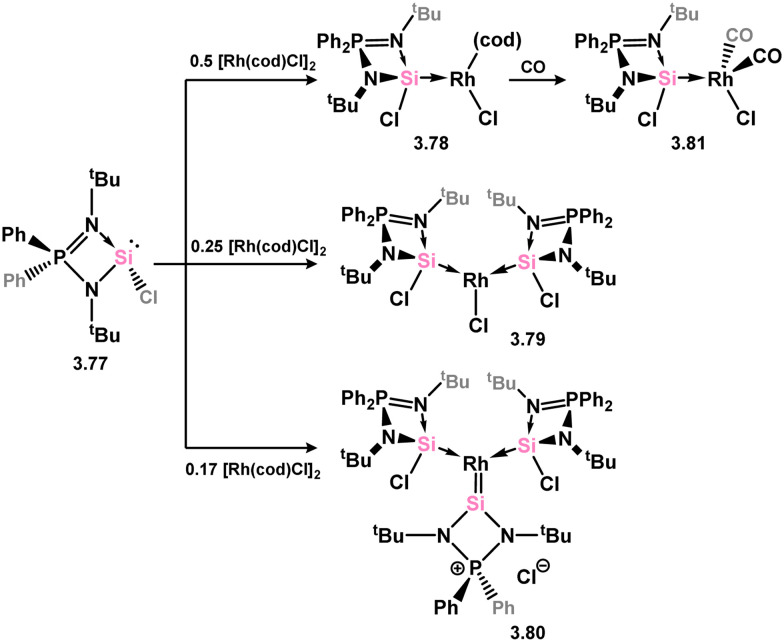
Reactivity of a (chloro)(iminophosphonamido)silylene towards rhodium(i).

The (chloro)(iminophosphonamido)silylene 3.77 was also recently reported, and its coordination chemistry towards [{Rh(cod)Cl}_2_] demonstrated. Up to three equiv. of the silylene bind Rh (3.78, 3.79, and 3.80; Scheme 20), with the third undergoing a resonance tautomerism to form two coordinate silylene ligand [Ph_2_P(^*t*^BuN)_2_Si:]^+^, in which the charge resides on the P centre.^104^ This is a distinct difference between this iminophosphonamido and the classical amidinate ligand, which leads to a non-innocence of the ligand due to the P^III^/P^V^ redox couple. Complex 3.78 further reacts with CO in substitution of the cod ligand, giving 3.81.

**Scheme 21:**
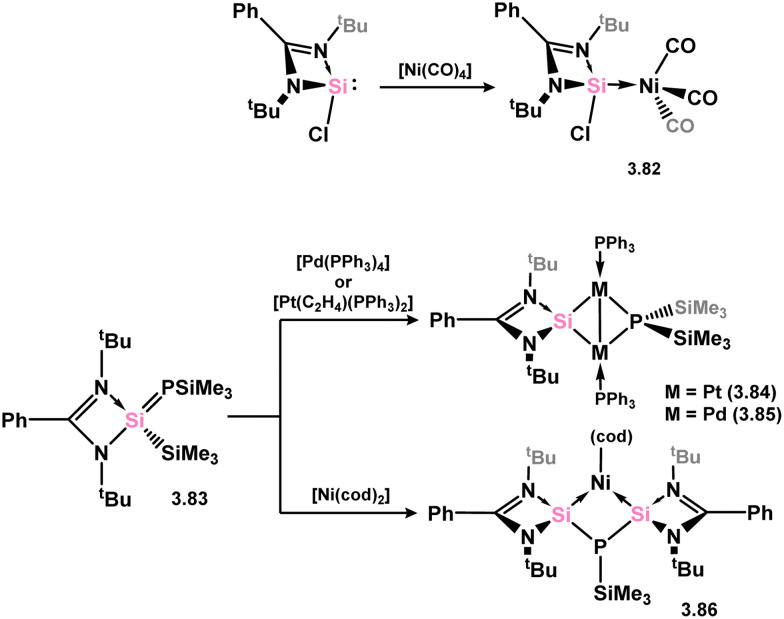
Reactions of an phosphasilene with nickel, palladium, and platinum species, leading to stable silylene complexes.

The majority of silylene complexes involving group 10 metals typically consist of chelating bis(NHSis), which are discussed at a latter point in this review. Similar to several examples mentioned earlier, the direct combination of 3.1 with [Ni(CO)_4_] resulted in carbonyl exchange and the formation of silylene complex 3.82 (Scheme 21).^105^ The reaction between phosphasilene 3.83 and the Pt^0^ complex [(C_2_H_4_)Pt(PPh_3_)_2_] led to the cleavage of the Si–P bond and the generation of a novel metallosilylene complex 3.84, with the Si-center of the ligand bridging two Pt centres. A similar reaction was observed with [Pd(PPh_3_)_4_], yielding complex 3.85 (Scheme 21).^52^ In comparison, the reaction with [Ni(cod)_2_] resulted in the elimination of (Me_3_Si)_3_P and the formation of a bis(silylenyl)phosphine complex of Ni^0^ (3.86).

**Scheme 22:**
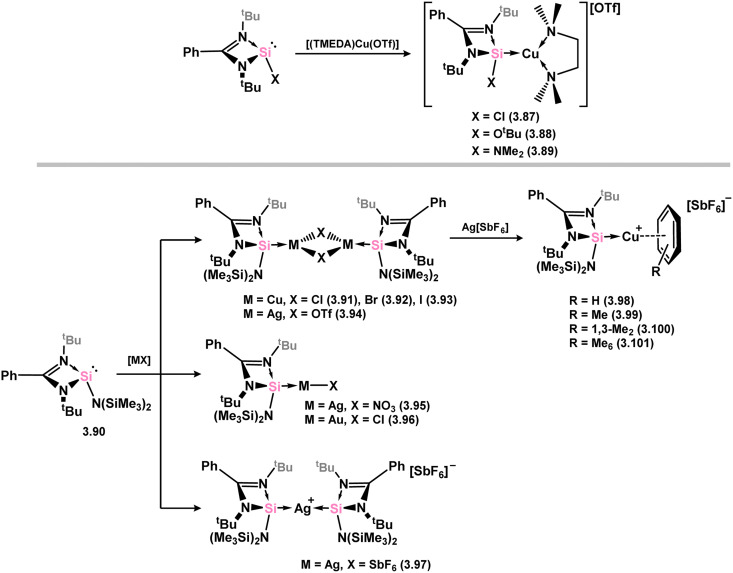
Synthesis of coinage metal complexes featuring amidinato–silylene ligands.

The initial discovery of 4-membered NHSi complexes of coinage metals focused on the Cu^I^ cation, [(TMEDA)Cu]OTf (TMEDA = *N*,*N*,*N*′,*N*′-tetramethylethylenediamine; OTf = triflate), along with previously described silylene ligands (*e.g.*3.1), leading to direct formation of complexes 3.87, 3.88, and 3.89 (Scheme 22).^106^ Bulky amido-silylene ligand 3.90 has also been utilised to support Cu^I^, Ag^I^, and Au^I^ complexes. [CuX] complexes (X = Cl, Br, I) were readily obtained by direct addition of 3.90 to Cu(i) halides, in forming 3.91, 3.92, and 3.93.^107,108^ Similar methods were employed to synthesise Ag^I^ and Au^I^ complexes 3.94, 3.95, and 3.96, as well as the cationic bis-silylene Ag^I^ complex 3.97.^107,108^ A range of cationic copper-arene complexes (arene = C_6_H_6_, MeC_6_H_5_, 1,3-Me_2_C_6_H_4_, Me_6_C_6_; 3.98, 3.99, 3.100, and 3.101) were accessed by bromide abstraction from 3.92 using Ag[SbF_6_] in aromatic solvents.^109,110^ Among them, toluene derivative 3.99 exhibited catalytic activity in the ‘click’ reaction of benzyl azides with both conjugated and aliphatic alkynes.

**Scheme 23:**
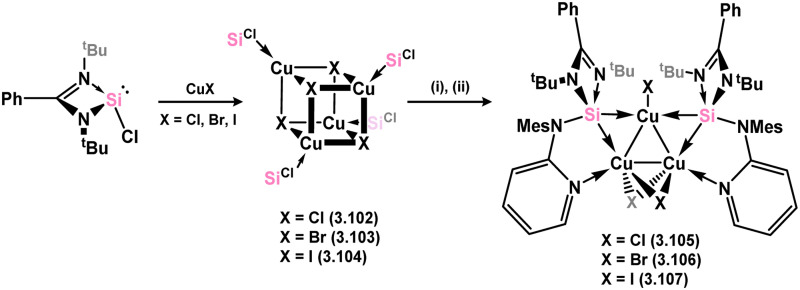
Synthesis of cubic and triangular copper(i) complexes featuring amidinato–silylene ligands.

Distinct copper(i) halide clusters with cubic [Cu_4_X_4_] cores were formed (3.102, 3.103, and 3.104, Scheme 23) by directly reacting chloro–silylene 3.1 with Cu^I^ halides, providing insights into the influence of silylene bulk on species aggregation.^111^ Further reaction of these clusters with a pyridine-functionalised secondary amine ligand, such as (Mes)(2-Py)NH, followed by Li[N(SiMe_3_)_2_], resulted in [Cu_3_X_3_] clusters stabilised by two pyridine-functionalised silylene ligands (3.105, 3.106, and 3.107). All complexes displayed activity in ‘click’ catalysis, with the introduction of the pyridine arm leading to significant enhancement in efficiency.

**Scheme 24:**
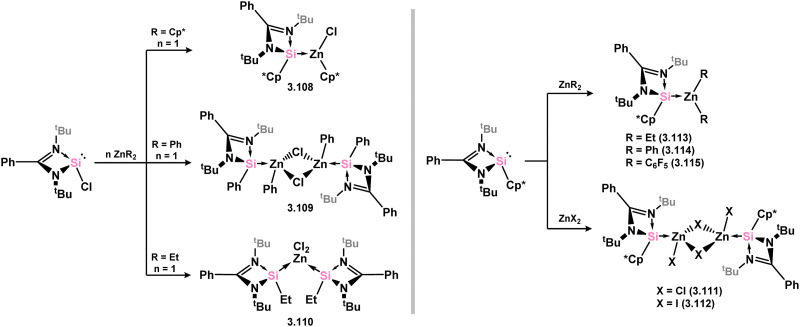
Synthesis of a range of monomeric and dimeric zinc(ii) complexes bearing amidinato–silylene ligands.

**Scheme 25:**
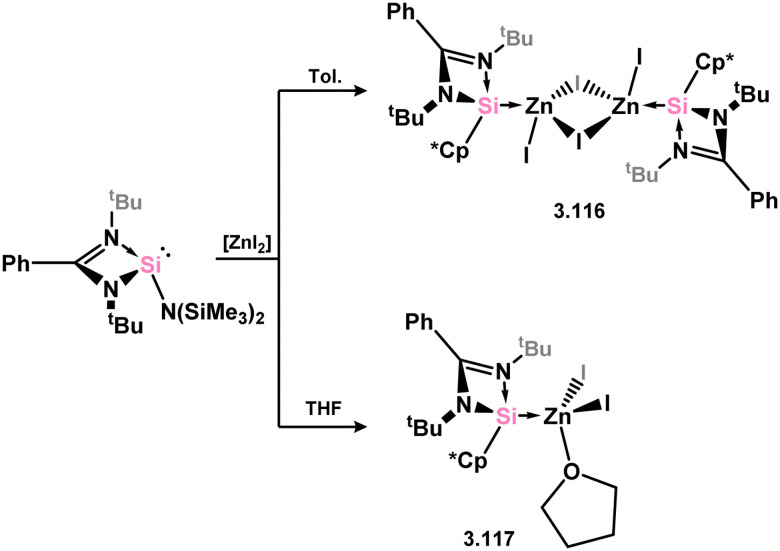
Solvent-dependant mono-dimer switchability of a 4-membered NHSi adduct of zinc(ii) iodide.

Only Zn^II^ complexes featuring 4-membered NHSi ligands have been reported among the group 12 metals. The initial instances of such complexes involved chloro–silylene 3.1, illustrating the facile ligand exchange between Si^II^ and Zn^II^ (Scheme 24).^112^ Specifically, the reaction of 3.1 with [Cp*_2_Zn], [Et_2_Zn], and [Ph_2_Zn] yielded cyclopentadienyl–, ethyl–, and phenyl–silylene complexes 3.108, 3.109, and 3.110, respectively. Additional cyclopentadienyl–silylene complexes with various Zn^II^ species were subsequently prepared by combining the independently synthesised ‘free’ (amidinato)(cyclopentadienyl)silylene with [ZnX_2_] (X = Cl, I, Et, Ph, C_6_F_5_; compounds 3.111, 3.112, 3.113, 3.114, and 3.115, respectively).^113^ Complexes of the bulky amido-silylene 3.90 with [ZnI_2_] were also synthesised by directly combining the ligand with [ZnI_2_], followed by recrystallisation from toluene, resulting in dimeric 3.116, or from THF/dioxane, leading to monomeric 3.117 (Scheme 25).^114^

##### 5-Membered

**Fig. 5:**
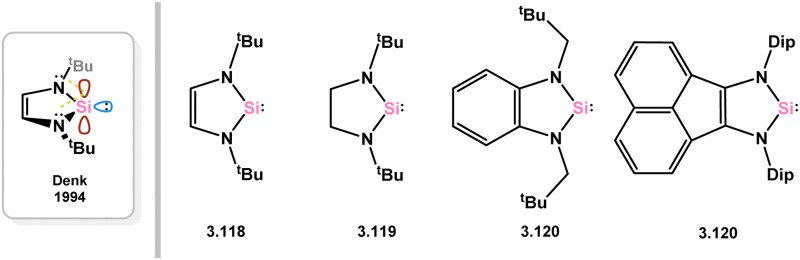
Examples of reported 5-membered NHSis, and a schematic for the frontier orbitals in such species.

Since the initial isolation of a stable 5-membered NHSi by the group of Denk (*viz.*3.118),^58^ which is stabilised by N → Si donation as per NHCs, a number of such compounds have been isolated (*e.g.*3.119, 3.120, 3.121, Fig. 5) and employed as ligands towards TM centres, albeit to a considerably lesser degree than closely related NHCs. Here, reported 5-membered NHSi complexes will be described, moving from group 5 to group 12 (no such complexes have been reported for group 4).

**Scheme 26:**
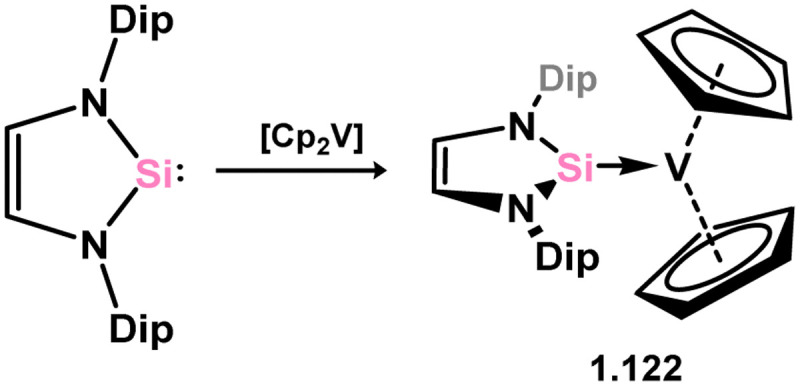
Synthesis of the sole reported example of a 5-membered NHSi complex of a group 5 metal.

A single complex has been documented of the group 5 metals, namely the [Cp_2_V] complex 3.122, which was obtained by directly combining the ‘free’ NHSi with the organometallic V^II^ fragment (Scheme 26).^115^ Interestingly, the same reaction does not occur for the related NHC ligand IPr (IPr = [(H)CN(Dip)]_2_C:; Dip = 2,6-^i^PrC_6_H_3_), highlighting the favorable π-acceptor properties of silylene ligands.

**Scheme 27:**
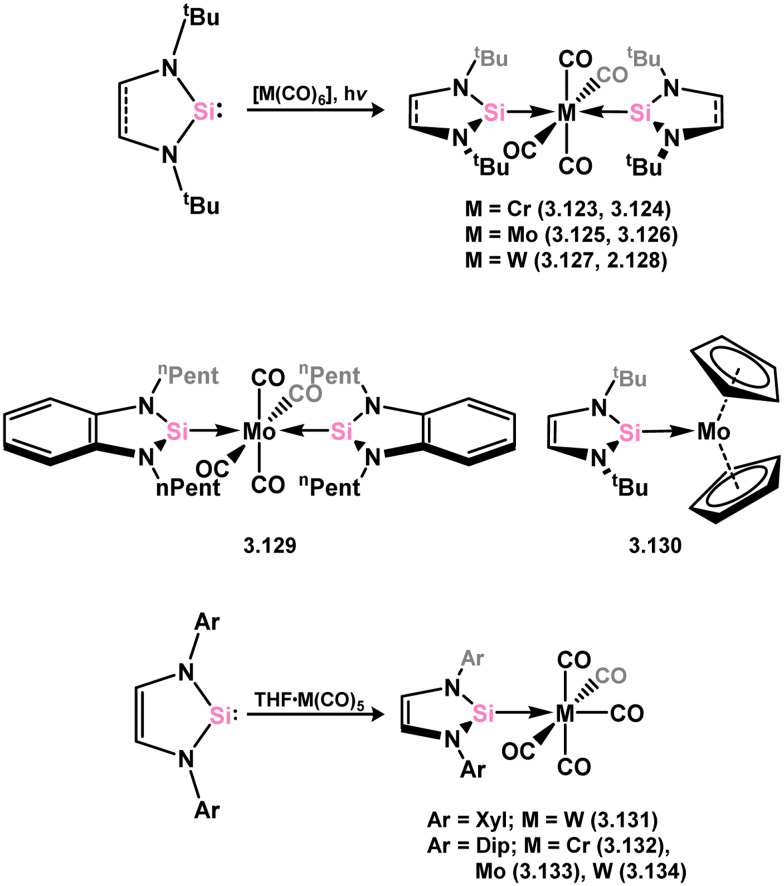
Synthesis and examples of group 6 complexes bearing 5-membered NHSis.

All examples of group 6 systems in this category were accessed through the direct addition of NHSis to metal carbonyl species under irradiation (Scheme 27). A comprehensive series of bis-silylene [M(CO)_4_] complexes were reported, employing both unsaturated and saturated ^*t*^Bu-substituted silylenes 3.118 and 3.119 (namely, 3.123, 3.124, 3.125, 3.126, 3.127, and 3.128).^116^ Additionally, the related Mo^0^ complex 3.129 with the *n*-pentyl-substituted silylene 3.120, as well as the mono-silylene complex of molybdocene (3.130), were reported.^117,118^ More recently, it was demonstrated that the use of bulkier NHSis, featuring flanking Xyl or Dip groups, leads to the formation of mono-silylene adducts of [M(CO)_5_] fragments (M = Cr–W). These adducts were accessed through addition of the silylene ligand to [THF·M(CO)_5_] species through THF substitution (3.131, 3.132, 3.133, and 3.134, Scheme 27).^119,120^

**Scheme 28:**
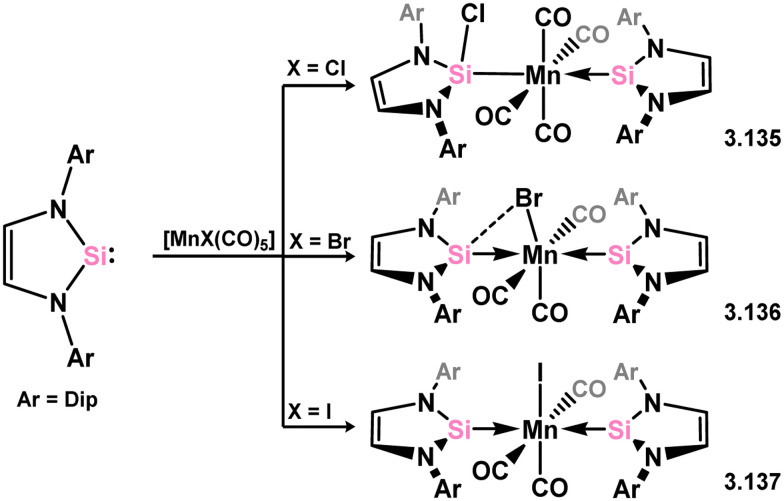
Synthetic access to manganese(i) complexes bearing 5-membered NHSis.

The reaction of Dip-substituted NHSi with pentacarbonyl Mn^I^ halides, [(CO)_5_MnX] (X = Cl, Br, I), resulted in two distinct outcomes. For the chloride complex, both double carbonyl substitution and insertion into the Mn–Cl bond occurred, giving rise to the (silylene)(silyl)manganese complex 3.135.^121^ In the case of the bromide and iodide complexes, double carbonyl substitution took place without Mn–X bond cleavage (3.136 and 3.137, respectively, Scheme 28).^120,121^

**Scheme 29:**
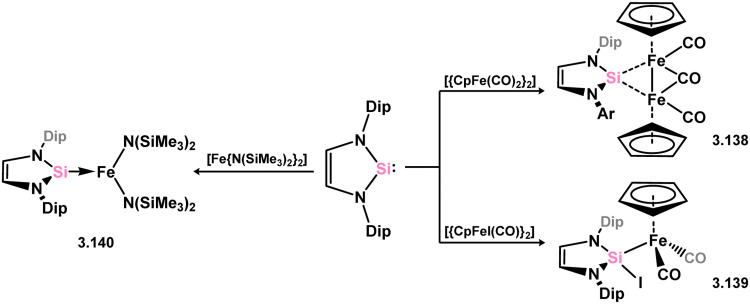
Reactivity of a 5-membered NHSi with iron(i) and iron(ii) species.

In addition to the previously reported NHSi complexes of iron, recent findings demonstrated that the same Dip-substituted NHSi reacts with dimeric [{CpFe(CO)_2_}_2_] through mono-carbonyl substitution, resulting in the formation of the silylene-bridged complex 3.138. On the other hand, reaction of the same silylene with the Fe^II^ complex [CpFeI(CO)_2_] exclusively led to insertion into the Fe–I bond, yielding 3.139 (Scheme 29).^120,121^ Similarly, the addition of the same silylene to bis-amido Fe^II^ species [Fe{N(SiMe_3_)_2_}_2_] produced the anticipated mono adduct 3.140.^122^

**Scheme 30:**
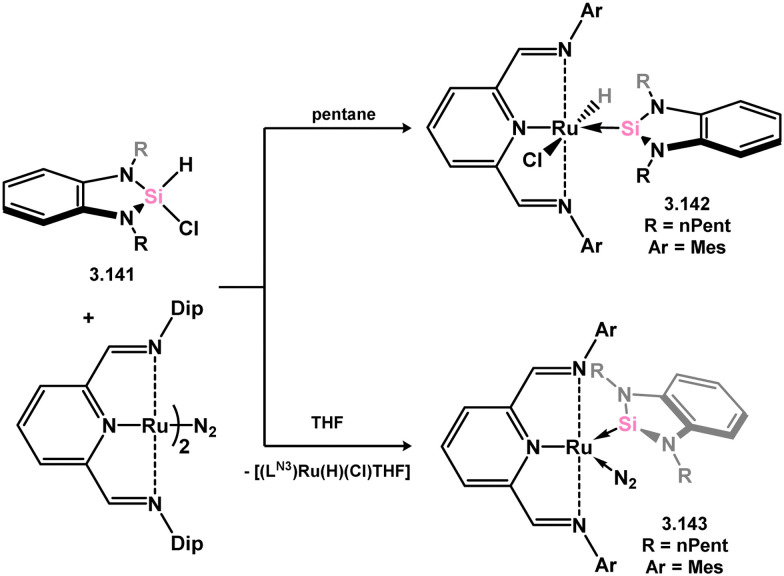
Reactivity of an N-heterocyclic silane toward ruthenium(0), leading to formation of 5-membered NHSi complexes.

It has been demonstrated that 5-membered NHSi complexes can be obtained from silane precursors through the reaction of silane 3.141 with bis(amino)pyridine Ru^0^ complexes (Scheme 30).^123^ When the reaction is carried out in pentane, the formation of silylene-coordinated Ru^II^ complex 3.142 is observed, proceeding *via* initial oxidative addition of the silane at Ru^0^ followed by chloride migration. On the other hand, conducting the reaction in THF leads to a mixture of THF-coordinated Ru^II^ complex [(LN_3_)Ru(H)(Cl)·THF] (LN_3_ = 2,6-CNAr-Py; Ar = Dip) and the dinitrogen- and silylene-bound Ru^0^ complex 3.143. It is proposed that NHSi complex 3.142 is initially formed, which then eliminates the NHSi through THF substitution. The free NHSi subsequently reacts with the Ru^0^ starting material, leading to the formation of 3.143.

**Scheme 31:**
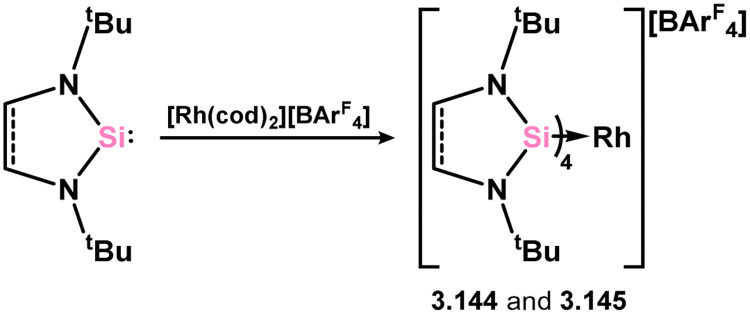
Access to rhodium(i) complexes featuring 5-memebered NHSi ligands.

Among the group 9 metals, only Rh complexes of 5-membered NHSis have been reported. Two complexes, incorporating the unsaturated or saturated ^*t*^Bu-substituted NHSis (3.118 and 3.119), were obtained by adding four equivalents of the NHSi ligand to [(cod)_2_Rh][BAr^F^_4_] (Ar^F^ = 3,5-CF_3_C_6_H_3_), resulting in the substitution of both cod ligands and the formation of 3.144 and 3.145.^124^ It was observed that, regardless of the number of equivalents of the NHSi ligand added, the tetrakis-complexes were consistently formed (Scheme 31).

**Scheme 32:**
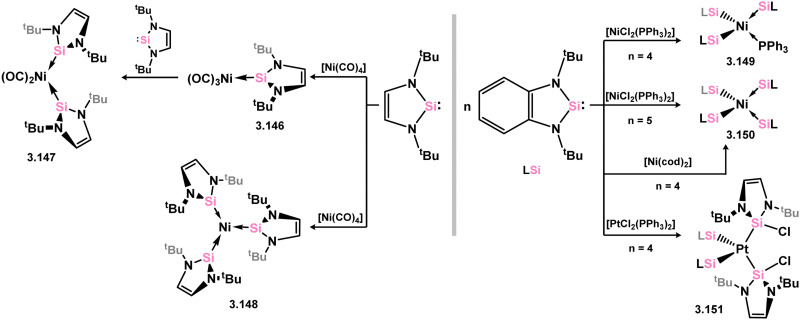
Early examples of 5-membered NHSi compounds of group 10 metals.

**Scheme 33:**
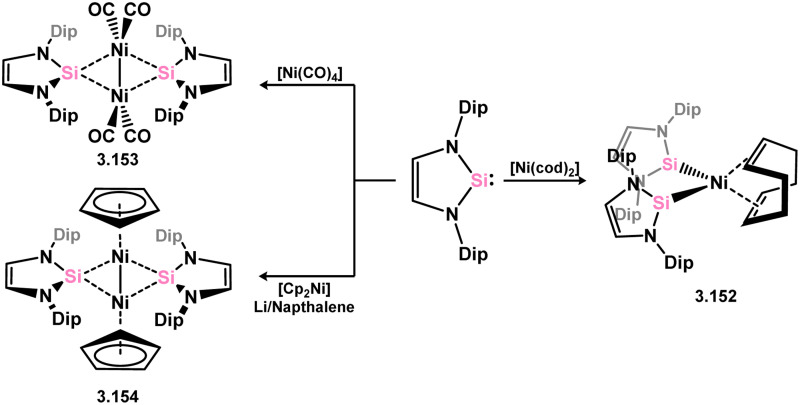
Synthesis of nickel(0) and nickel(i) species bearing 5-membered NHSi ligands.

Early examples of group 10 5-membered NHSi–TM complexes were reported by the groups of West, Denk and co-workers, or Lappert and co-workers, accessed through the addition of the free silylene ligands to either [Ni(CO)_4_], or [MCl_2_] species (M = Ni, Pt; Scheme 32). This led to mono- (3.146), bis- (3.147), tris- (3.148 and 3.149), and tetra-NHSi (3.150) complexes of Ni^0^, in addition to a Pt–Cl activated bis-complex, bearing two NHSi ligands (3.151).^125^ Since then, a small number of additional nickel complexes have been documented (Scheme 33). However, employing the bulkier Dip-substituted derivative only led to bis-ligation, forming the bis(NHSi)–Ni(cod) complex 3.152.^126^ Addition of the same silylene to [Ni(CO)_4_] resulted in the loss of two equivalents of CO and dimerisation, leading to the NHSi-bridged complex 3.153, again contrasting with the related reaction for the less bulky ^*t*^Bu derivative.^120^ Reaction with [Cp_2_Ni], along with simultaneous reduction with Li naphthalenide, produced the similarly bridged Ni^I^ dimer 3.154, with the loss of [LiCp].^121^

**Scheme 34:**
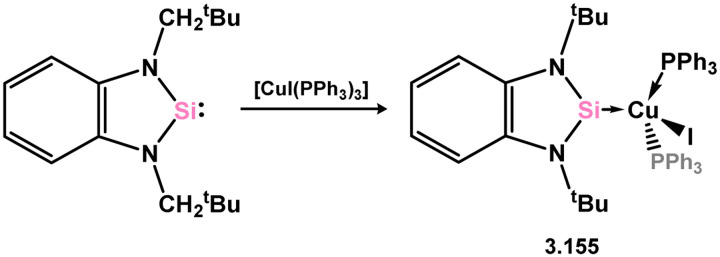
Synthesis of the sole example of a 5-membered NHSi adduct of a coinage metal.

One example of a copper NHSi complex has been reported, accessed through the direct addition of the NHSi ligand to [CuI(PPh_3_)_3_] (3.155, Scheme 34).^127^ No such species have been documented for any group 12 metal.

##### 6-Membered

**Scheme 35:**
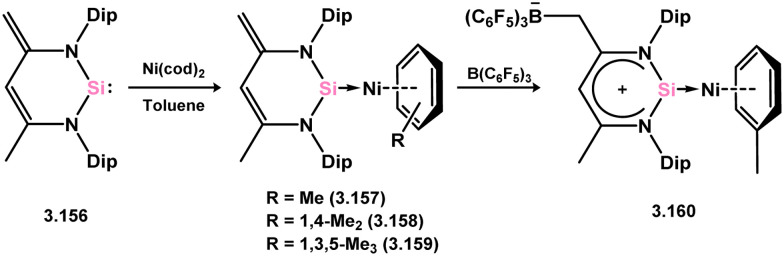
Accessing nickel(0) arene complexes supported by a 6-membered NHSi.

The group of Driess has extensively reported on the chemistry involving 6-membered silylene 3.156, with a focus on nickel complexes. The initial complex of this family was obtained by addition of 3.156 to [Ni(cod)_2_] in aromatic solvents, resulting in silylene Ni^0^ complexes 3.157, 3.158, and 3.159, where arene ligands are η^6^-coordinated to Ni^0^ (Scheme 35).^128^ It was also demonstrated that the addition of strongly Lewis acidic borane B(C_6_F_5_)_3_ to toluene-capped complex 3.157 led to the formation of a cationic species 3.160, with enhanced bonding interactions between Si^II^ and Ni^0^, attributed to the charge delocalisation in the unsaturated ligand backbone of silylene 1.356.

**Scheme 36:**
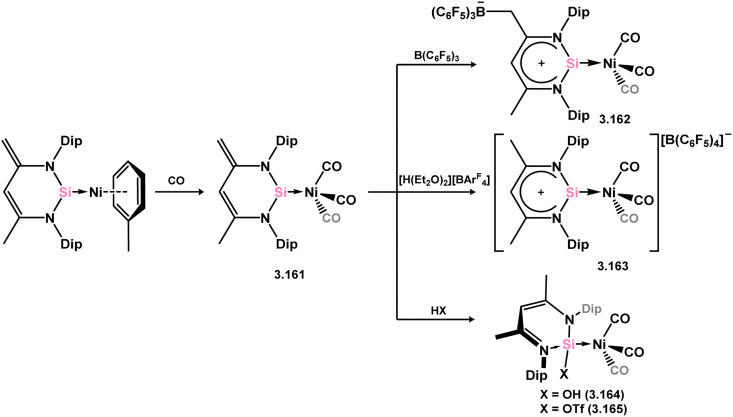
Ligand exchange chemistry of a 6-membered NHSi-supported nickel(0) arene complex.

**Scheme 37:**
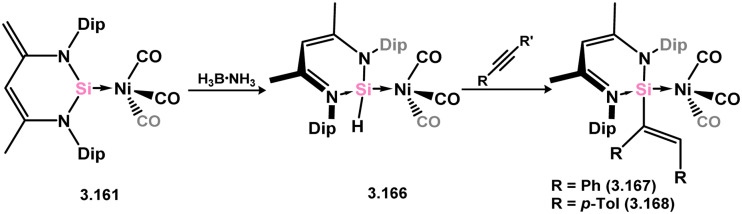
Synthesis and insertion chemsitry of a (hydrido)silylene–nickel(0) complex.

Further investigations revealed that the Ni^0^ arene fragment could be replaced with the Ni^0^ tris-carbonyl fragment, resulting in the formation of 3.161 under a CO atmosphere. This species enabled the addition of acidic species across the unsaturated ligand backbone/Si^II^ center, leading to the synthesis of various Si-substituted nickel-silylene complexes (3.162, 3.163, 3.164, and 3.165, Scheme 36).^129,130^ Additionally, the synthesis of Ni-stabilised Si^II^ hydride complex 3.166 was achieved by hydrogenation of 3.157 with ammonia borane (Scheme 37). Complex 3.166 exhibited higher reactivity compared to classical Si^IV^ hydrides, readily undergoing insertion reactions with a variety of alkynes to form vinyl-silylene complexes of Ni^0^, 3.167 and 3.168. Computational analyses suggested that this process involved alkyne coordination at Ni^0^, highlighting the potential for synergistic metal–ligand effects in such systems.^131^

**Scheme 38:**
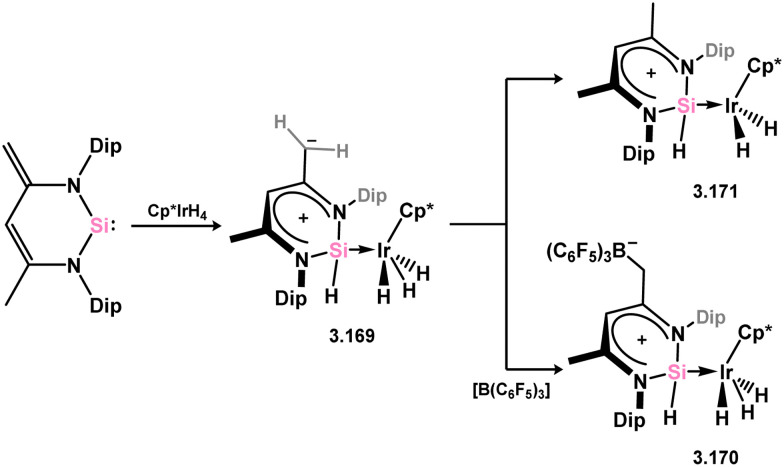
Reactivity of a 6-membered NHSi towards an iridium(v) tetryhydride complex.

Iridium di- and tri-hydride complexes supported by the 6-membered NHSi ligand have been obtained through the addition of [Cp*IrH_4_] to 3.157 (Scheme 38).^132^ Initially, a silyl–iridium complex 3.169 is formed *via* Si^II^ insertion into one Ir–H bond. Subsequent addition of B(C_6_F_5_)_3_ to this intermediate species yields the hydrido–silylene complex 3.170 with three hydride ligands coordinated to Ir. After 24 h in solution in the absence of B(C_6_F_5_)_3_, hydride migration from Ir to the silylene ligand backbone occurs, resulting in the hydrido-silylene complex 3.171 with two hydride ligands at Ir.

#### 3.1.2. Further cyclic-silylene complexes

**Fig. 6:**
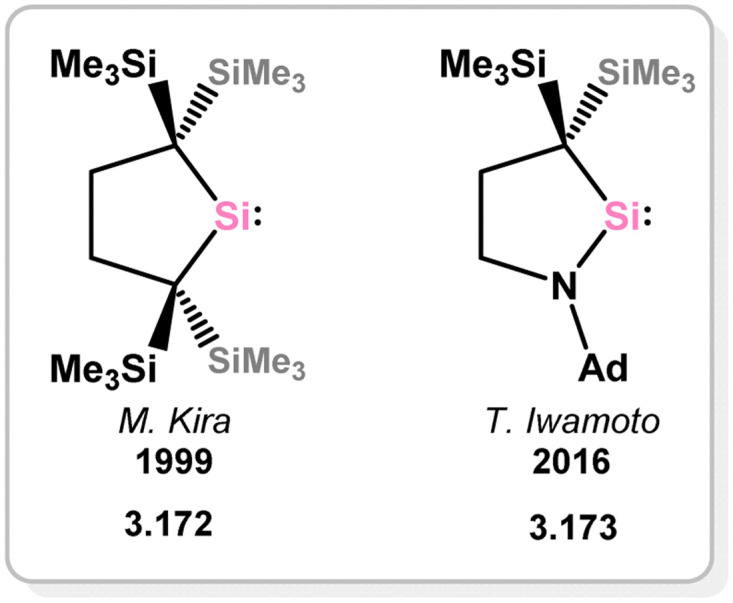
Examples of addition cyclic silylene derivatives.

In addition to the mentioned 4-, 5-, and 6-membered NHSi ligands, a limited number of complexes incorporating related cyclic silylenes have been documented, primarily involving silylenes 3.172 and 3.173 (Fig. 6).^133,134^

**Scheme 39:**
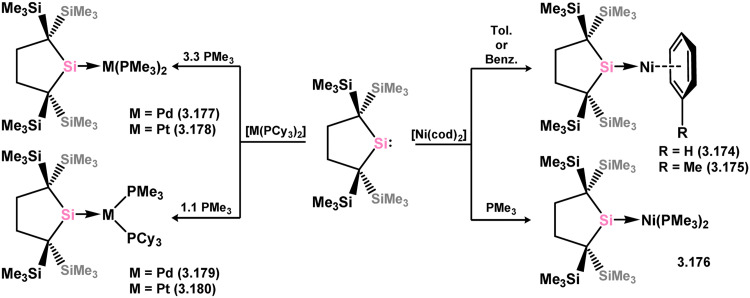
Synthesis of nickel(0), palladium(0), and platinum(0) complexes bearing a cyclic bis(alkyl)silylene ligand, with additional arene and phosphine ligands.

The 5-membered cyclic bis(alkyl)silylene 3.172 has been utilised in generating mono(silylene) Ni^0^ (3.174, 3.175, and 3.176), Pd^0^ (3.177 and 3.178), Pt^0^ (3.179, and 3.180) complexes, through direct reactions with M^0^ precursors (M = Ni–Pt), followed by ligand exchange processes (Scheme 39).^135^ Due to the electron-deficient nature of the Si^II^ center in this silylene ligand compared to N-heterocyclic derivatives, significant backbonding from the M^0^ centers to Si^II^ is observed, resulting in contracted M–Si double bonds.

**Scheme 40:**
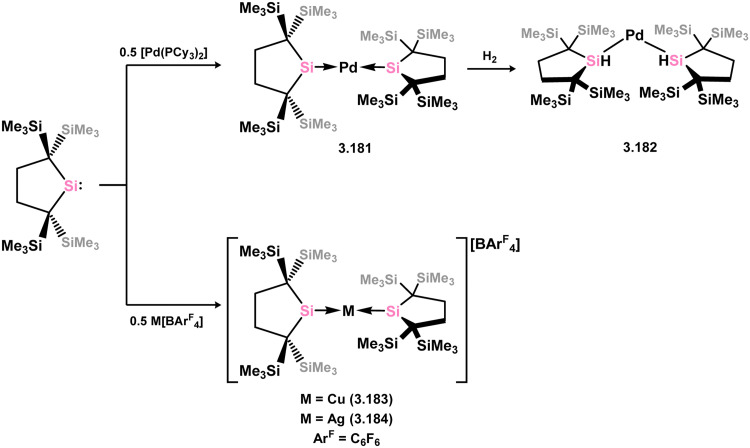
Synthesis of palladium(0) and coinage metal complexes bearing a cyclic bis(alkyl)silylene ligand, and dihydrogen activation by the former.

The 14-electron bis(silylene)palladium complex 3.181 was also obtained by combining two equivalents of 3.172 with [(Cy_3_P)_2_Pd], which demonstrated the ability to readily cleave H_2_ at room temperature, yielding the Pd^II^ disilane complex 3.182 (Scheme 40).^136^ Isoelectronic cationic Cu^I^ and Ag^I^ complexes were also synthesised by directly combining silylene 3.172 with cationic Cu and Ag fragments, resulting in 3.183 and 3.184.^137^

**Scheme 41:**
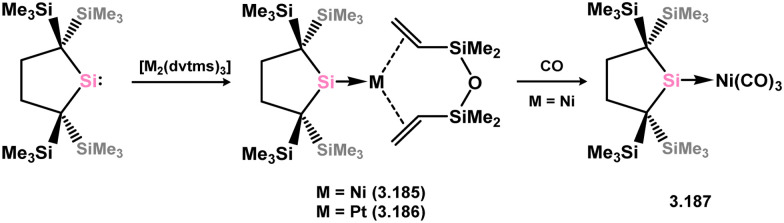
Synthesis of nickel(0) and platinum(0) complexes bearing a cyclic bis(alkyl)silylene ligand, with additional alkene or carbonyl ligands.

**Scheme 42:**
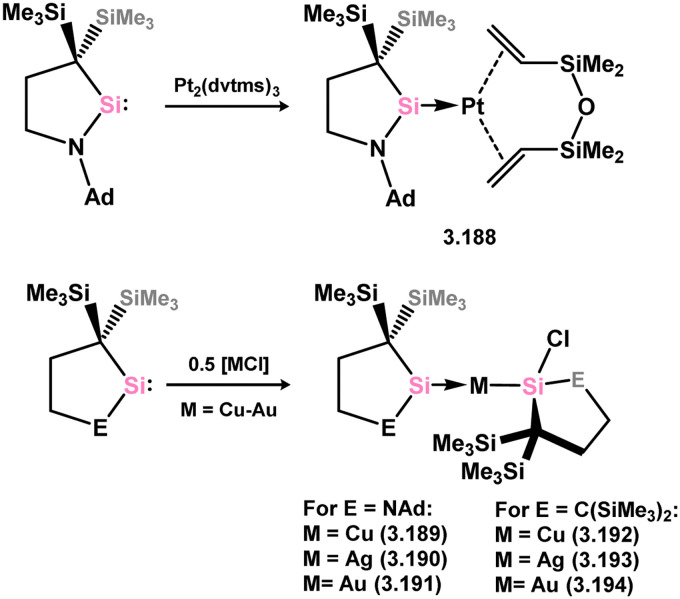
Formation of a platinum(0) complexes bearing a cyclic (alkyl)(amido)silylene, and related reactivity towards coinage metal halides.

Further examples of Ni^0^ and Pt^0^ complexes were reported (*viz.*3.185, 3.186, and 3.187, Scheme 41), accessed by combining silylene 3.172 with [M_2_(dvtms)_3_], followed by ligand exchange with CO for the Ni system.^138,139^ In regards to the cyclic (alkyl)(amido)silylene 3.173, one group 10 complex has been reported, namely the Pt^0^ bis(alkene) species 3.188, synthesised by combination of the silylene ligand with [Pt_2_(dvtms)_3_] (Scheme 42).^140^ Additionally, coinage metal complexes have also been reported, whereby addition of two equiv. of the ligand to [MCl] (M = Cu, Ag, Au) led to insertion into the M–Cl bond by one silylene ligand, forming (silyl)(silylene) complexes 3.189, 3.190, and 3.191. This was also shown to occur with the related cyclic bis(alkyl)silylene, forming complexes 3.192, 3.193, and 3.194.^141^

**Scheme 43:**
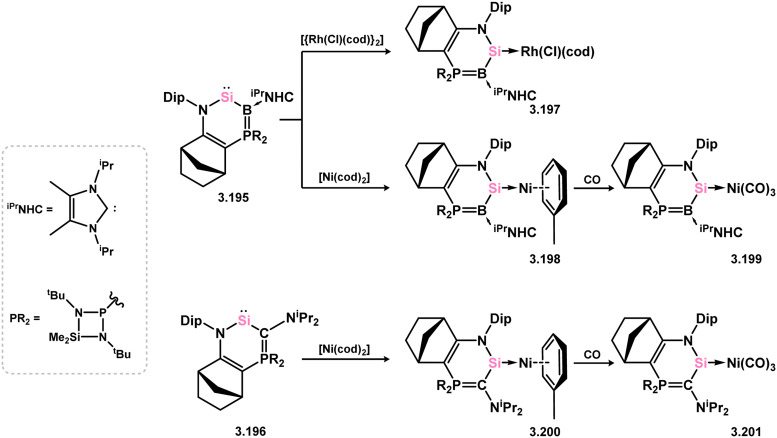
Transition metal complexation of unique heterocyclic silylenes featuring bora- and phospha-ylidic moieties.

The unique phospha- and bora-ylidic heterocyclic silylenes 3.195 and 3.196 have demonstrated their ability to act as particularly strong donor ligands in complexes 3.197, 3.198, and 3.199 (for 3.195), and 3.200, and 3.201 (for 3.196) (Scheme 43).^142,143^ Notably, the CO stretching frequencies in [Ni(CO)_3_] complexes were compared to those of known phosphine, carbene, and silylene complexes, suggesting that both 3.195 and 3.196 exhibit stronger donor characteristics than the majority of these ligands. However, this observation has not been implemented in catalytic or further reactivity studies.

#### 3.1.3. Acyclic silylene complexes

While the majority of cyclic NHSi TM complexes are derived from isolable NHSis, the case is quite different for most acyclic silylene complexes, with room-temperature stable base-free derivatives only very recently being realised.^29,144–146^ Here, base-free acyclic silylene TM complexes will be described systematically, moving from group 4 to group 12. Since several of these complexes exhibit reactivity relevant to intermediates in hydrosilylation catalysis or demonstrate interesting reactivity in their own right, we will also highlight key examples of those scenarios.

**Scheme 44:**
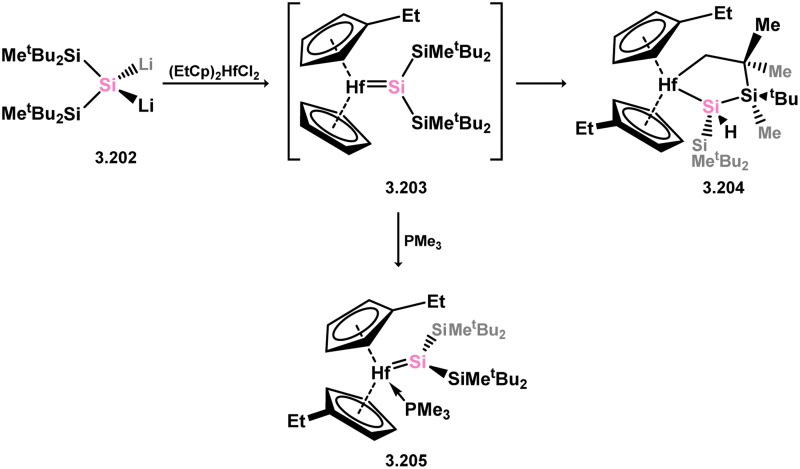
Synthesis of a hafnium complex bearing an acyclic silylene ligand, *via* salt-metathesis.

Only one structurally characterised acyclic silylene group 4 complex is known, being a hafnocene complex (Scheme 44).^63^ This was obtained by reacting dilithiosilane 3.202 with hafnocene dichloride, initially forming the unstable 16-electron complex 3.203. Under these conditions, C–H activation occurred, resulting in the formation of 3.204. However, the addition of PMe_3_ led to the isolation of a stable 18-electron complex, 3.205, which exhibits significant Si–Hf double bond character.

**Scheme 45:**
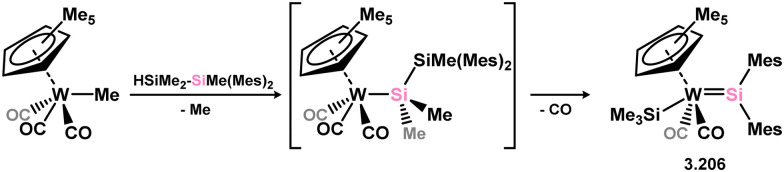
Synthesis of a tungsten complex bearing an acyclic silylene ligand, *via* σ-metathesis and group migration.

Given the ability of group 6 metals to form alkylidyne complexes, they have been extensively investigated for the formation of their heavier analogues, metallo–silylidyne complexes, which will be discussed later in this review. Several synthetic routes to access these species involve acyclic–silylene complexes, which themselves are relatively rare. The first example of a base-free silylene complex of a group 6 metal was the tungsten species 3.206, featuring the Mes_2_Si ligand (Scheme 45). This complex possesses a very short Si–W double bond (2.3850 Å), particularly when compared to the Si–W single bond in the same molecule (2.6456 Å).^147^ The formation of this complex involved the photolysis of the methyl tungsten complex, [Cp*WMe(CO)_3_], in the presence of the disilane HMe_2_Si–SiMeMes_2_, leading to initial dealkylation followed by a series of migratory reactions resulting in the formation of the silylene complex.

**Scheme 46:**
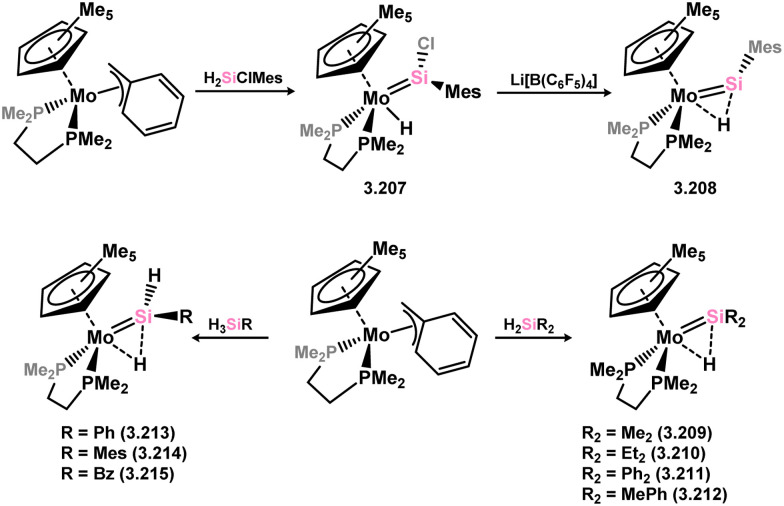
Synthesis of molybdenum complexes bearing acyclic silylene ligands, *via* σ-metathesis and hydride migration.

**Scheme 47:**
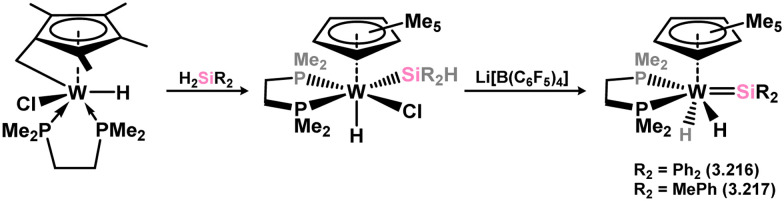
Synthesis of a tungsten complex bearing an acyclic silylene ligand, *via* σ-metathesis, chloride abstraction, and hydride migration.

The (mesityl)(chloro)silylene complex 3.207 was obtained using a similar alkyl elimination strategy, involving the reaction of MesSiClH_2_ with [Cp*Mo(dmpe)Bz] (Scheme 46).^148^ Initially, the elimination of toluene takes place, followed by H-migration to Mo, resulting in the formation of the desired silylene complex. This species was utilised in accessing the first example of a metal complex with some degree of M–Si triple bond character (*e.g.*3.208). However, due to the bridging nature of the hydride ligand, the triple bond is somewhat perturbed. The same research group employed the benzyl elimination approach to access a range of bis-alkyl (3.209, 3.210), bis-aryl (3.211), and (alky)(aryl)-silylene (3.212) complexes of molybdenum hydrides, as well as (aryl)(hydrido)silylene complexes 3.213, 3.214, 3.215, exhibiting varying degrees of Mo–H⋯Si interactions.^149^ Similar chemistry was achieved using a C–H activated Cp* tungsten complex (Scheme 47). Addition of silane leads to protonation and regeneration of the Cp* ligand, resulting in the formation of base-free silylene complexes 3.216 and 3.217.^150^

**Scheme 48:**
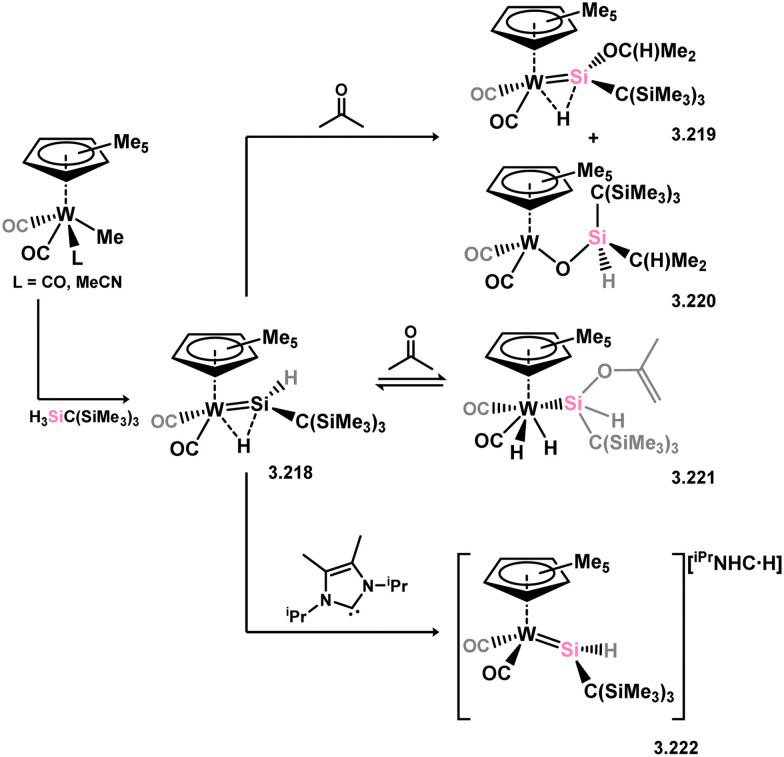
Synthesis of a tungsten complex bearing a hydride-bridged acyclic hydrido-silylene ligand, and its reactivity.

An alternative route to sterically encumbered (alkyl)(hydrido)silylene tungsten complexes was subsequently reported. Here, the direct reaction of Cp-bound tungsten methyl complexes (*i.e.* [CpW(CO)_2_Me L]; L = CO, MeCN) with monoalkylsilanes led to methyl elimination and H-migration to form 3.218 (Scheme 48).^151^ Addition of acetone to 3.218 triggers various reactive processes, including the insertion of the C=O bond into the Si–H bond, forming 3.219, and C=O bond metathesis, forming 3.220. The reversible addition of one C–H bond of acetone is also observed, forming 3.221. Furthermore, it was later discovered that the addition of ^iPr^NHC (^iPr^NHC = [{(Me)CN(^i^Pr)}_2_C:]) to 3.218 results in hydride abstraction from this complex, forming anionic 3.222 with a contracted Si–W bond length, indicative of the “formal” silylene character (*i.e.* without bridging hydride interaction).^152,153^

**Scheme 49:**
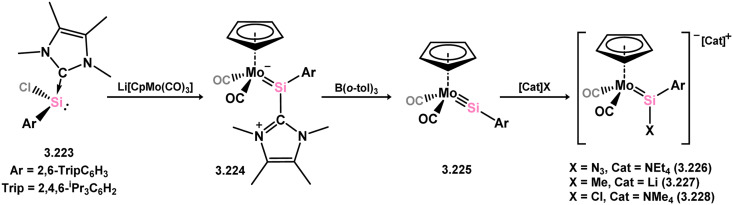
Synthesis of molybdenum complex bearing acyclic silylene ligands, *via* salt metatheses.

**Scheme 50:**
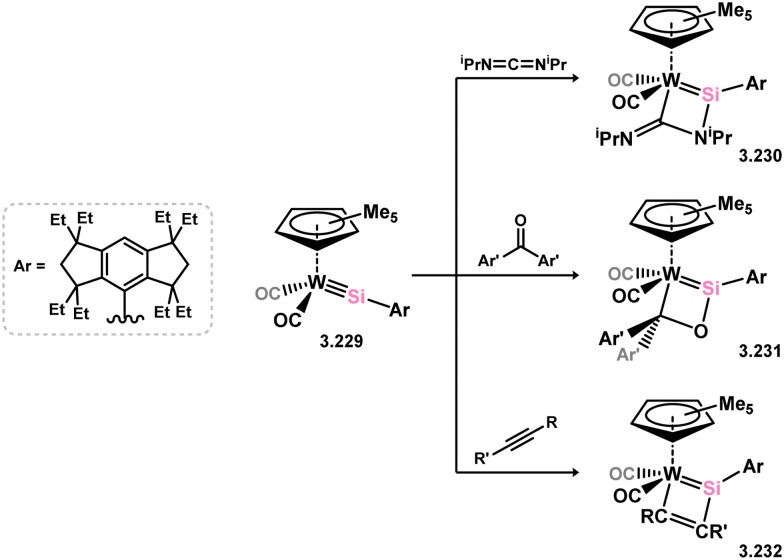
Synthesis of a tungsten complexes bearing acyclic silylene ligands, *via* cycloaddition of a tungsten-silylidyne complex.

*En route* to a stable molybdenum silylidyne complex, the NHC-stabilised (aryl)(chloro)silylene 3.223 was reacted with the anionic molybdenum salt Li[CpMo(CO)_3_], resulting in metathesis and the formation of 3.224; the most likely resonance form features a positively charged carbene (*i.e.* imidzolium) ligand, and an anionic molybdenum center. As such. 3.224 represents a formal silylene complex (Scheme 49).^154^ Subsequent NHC abstraction led to the first example of a molybdenum silylidyne complex, 3.225. Addition of nucleophiles to this species generally resulted in addition at Si, yielding a range of novel molybdenum silylene complexes, such as 3.226, 3.227, and 3.228.^155^ This indicates the electrophilic nature of the silicon center in triply-bonded M–Si species. The [2+2] cycloaddition of carbodimides and ketones to metal silylidyne complexes also leads to metal silylene complexes, forming 4-membered metallacycles with metal–silicon double bonds. Complex 3.229 exhibits such reactivity, giving rise to 3.230, 3.231, and 3.232, all of which contain a silylene fragment (Scheme 50).^156,157^

**Scheme 51:**
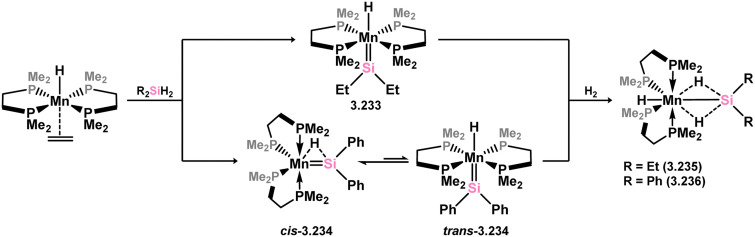
Synthesis of manganese complexes bearing an acyclic silylene ligands, *via* dihydrogen elimination.

Two acyclic silylene complexes of group 7 metals have been identified. They are obtained through the addition of diethyl or diphenyl silane to the alkene-coordinated Mn^I^ hydride complex [(dmpe)_2_MnH(C_2_H_4_)], resulting in the formation of 3.233 and 3.234, accompanied by the elimination of ethane (Scheme 51).^158^ While the diethyl derivative 2.233 exclusively exists as the *trans*-isomer without Mn–H⋯Si interactions, the *cis*-isomer is observed in the case of the diphenyl derivative 3.234. Both complexes react with H_2_ to yield hydride-bridged silyl complexes 3.235 and 3.236.

**Scheme 52:**
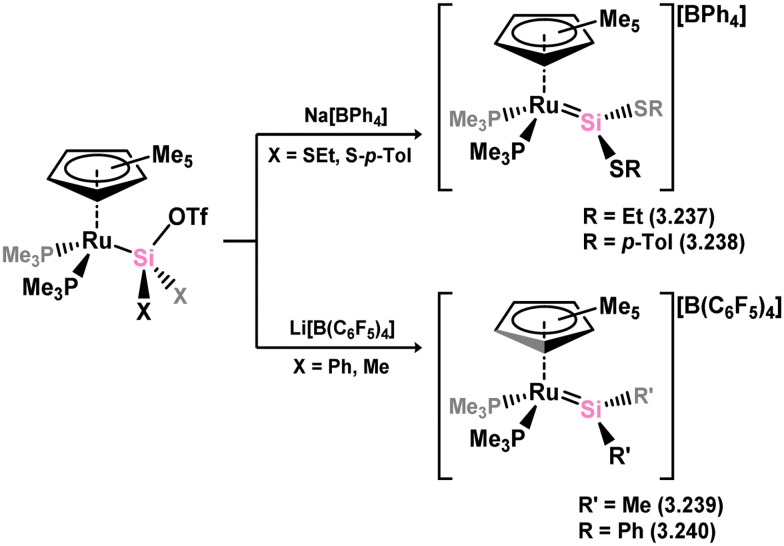
Synthesis of a rutheneium complexes bearing acyclic silylene ligands.

The first base-free silylene complexes were reported in 1990 in the cationic ruthenium species 3.237 and 3.238, synthesised through triflate abstraction from their silyl complexes.^159^ Four years later, silylene complexes without π-donating substituents at Si^II^ were reported (3.239 and 3.240, Scheme 52).^160^ Since then, several group 8 silylene complexes have been documented.

**Scheme 53:**
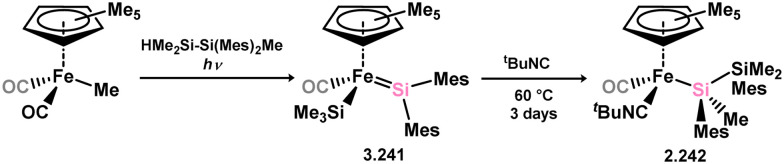
Synthesis of an iron complex bearing an acyclic silylene ligand, *via* σ-metathesis and group migration.

**Scheme 54:**
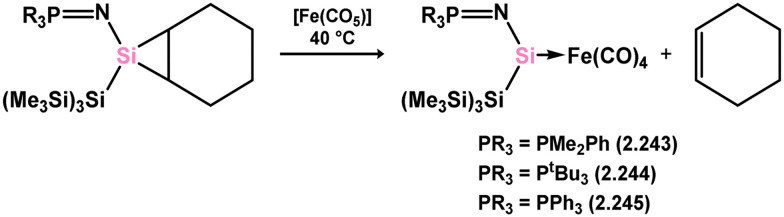
Synthesis of an iron complex bearing an acyclic silylene ligand, *via* cyclohexene elimination.

Iron complexes featuring an acyclic silylene ligand are rather rare. The first, 3.241, was synthesised using a similar approach as tungsten complex 3.206. Irradiation of a mixture of [Cp*FeMe(CO)_2_] and disilane HMe_2_Si–SiMeMes_2_ resulted in the initial loss of methane, followed by Me_3_Si migration.^161^ Upon addition of *tert*-butyl isocyanide, evidence of both 1,2- and 1,3-group migrations in 3.241 was observed, leading to the formation of silyl complex 3.242 (Scheme 53). These findings provide insights into the fluxional binding behavior in such complexes. More recently, a handful of additional Fe complexes were reported, accessed from ‘masked’ (phosphinimide)(silyl)silylenes (Scheme 54). These precursors, akin to earlier reported 3-membered silacycles,^162–165^ can eliminate their cyclohexane unit in a retro-[2+1] reaction, releasing cyclohexene. The resulting silylenes are trapped by additional substrates, *e.g.* [Fe(CO)_4_].^166^ In this manner, complexes 3.243, 3.244, and 3.245 were isolated.

**Scheme 55:**
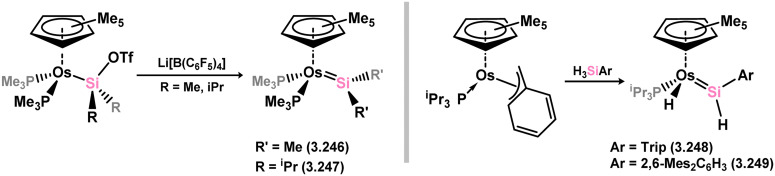
Synthesis of osmium complexes bearing acyclic silylene ligands, *via* salt elimination, and σ-metathesis and hydride migration.

Osmium complexes with related silylene ligands were accessed through initial dealkylation reactions of Os^II^ alkyls. Specifically, the formation of bis(alkyl)silylene complexes 3.246 and 3.247 involved this initial dealkylation reaction, followed by the conversion of (chloro)silyl complex [Cp*Os(PMe_3_)_2_(SiClR_2_)] to the corresponding triflate, and subsequent triflate abstraction (Scheme 55).^167^ The direct dealkylation of Os^II^ benzyl species [Cp*Os(P^i^Pr_3_)(Bz)] with aryl silanes, followed by spontaneous hydride migration, led to the formation of (hydrido)(aryl)silylene complexes 3.248 and 3.249.^168^

**Scheme 56:**
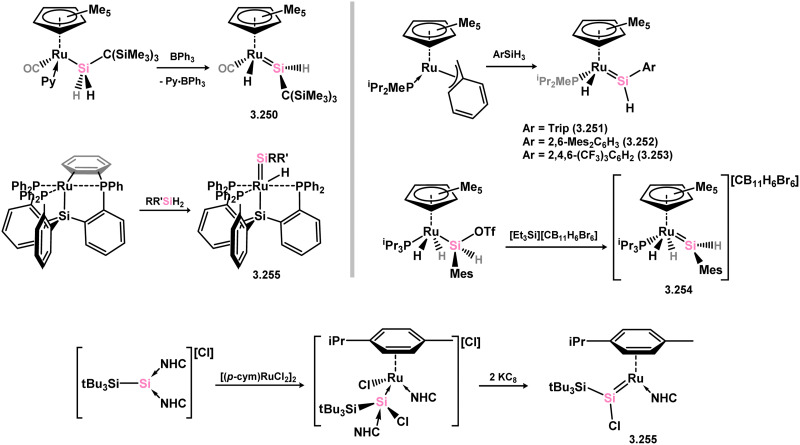
Synthesis of numerous ruthenium complexes bearing acyclic silylene ligands.

Further examples of ruthenium–silylene complexes have been synthesised through alkyl abstraction and hydride/group migration methods.^169–171^ Specifically, examples of (alkyl)(hydrido)- (3.250) and (aryl)(hydrido)-silylene (3.251, 3.252, 3.253, and 3.254) complexes having been accessed utilising a [Cp*Ru] moiety, as well as the unique scorpionate complex 3.255,^172^ where silane addition leads to H-transfer to one activated Ph group of the ligand (Scheme 56). Additionally, the (chloro)(silyl)silylene complex 3.256 was prepared by initially combining the cationic NHC-stabilised silylene [(IMe)_2_(^*t*^Bu_3_Si)Si]Cl with arene-coordinated [{RuCl_2_(p-cym)}_2_] (*p*-cym = 1-Me-4-^i^PrC_6_H_4_), followed by reduction with KC_8_ (Scheme 56).^173^

**Scheme 57:**
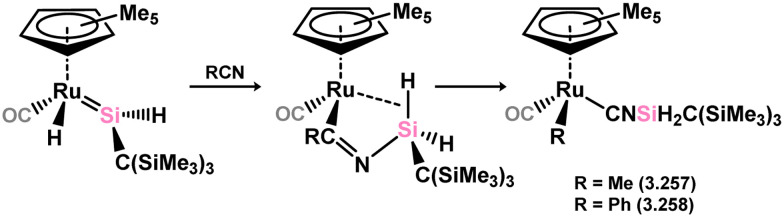
Reactivity of an acyclic–silylene ruthenium complex torwards organonitriles.

**Scheme 58:**
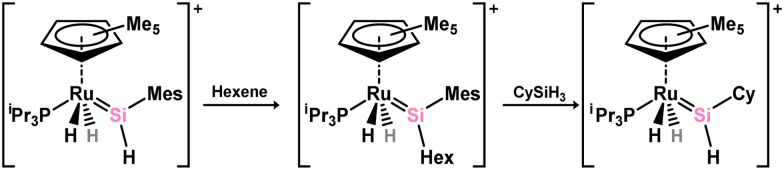
Insertion chemistry of an acyclic–(hydrido)silylene ruthium complex towards alkenes, and subsequent metathesis chemsitry with a silane.

Further investigations into the chemistry of several ruthenium–silylene complexes also reveal intriguing points. The (hydrido)silylene complex 3.250 demonstrated reactivity with nitriles, whereby insertion into the Ru–Si double bond and subsequent bond migration processes result in the formation of silyl–isocyanide complexes 3.257 and 3.258 (Scheme 57).^169^ In a more practical context, the closely related hydrido complex 3.254 exhibited effective catalytic activity in the hydrosilylation of alkenes, for which the postulated mechanism involves alkene insertion into the Si–H bond of the silylene ligand (Scheme 58).^171^ This previously unobserved mechanism highlights the potential significance of silylene–metal complexes as intermediates in industrially applicable hydrosilylation catalyses.

**Scheme 59:**
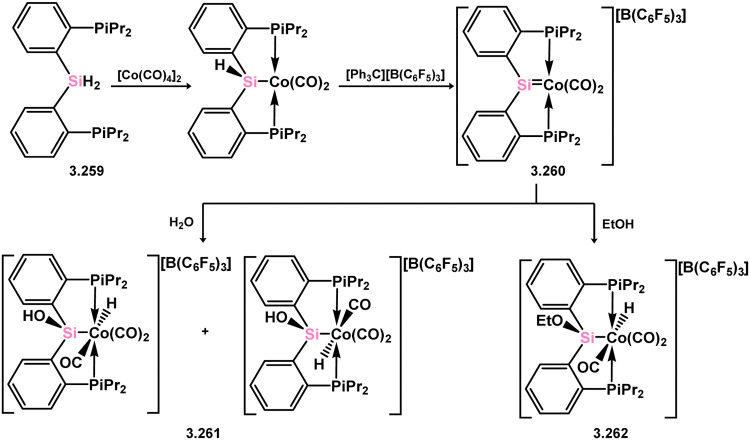
Synthesis and addition chemistry of a cobalt complex bearing an acyclic silylene ligand.

Among the group 9 elements, iridium dominates the field of base-free acyclic silylene chemistry. These complexes, besides a single scorpionate-silylene cobalt example with chelating phosphine arms (Scheme 59), represent the only stable and structurally characterised examples. The formation of the aforementioned cobalt complex involved the combination of phosphine-functionalised bis(aryl)silane 3.259 with [Co_2_(CO)_8_], leading to the loss of H_2_ and CO. Subsequent hydride abstraction from the resulting silyl complex yielded the cationic cobalt silylene complex 3.260.^174^ Notably, the Si^II^ center in this species exhibited Lewis acidity, enabling the binding of nucleophiles and facilitating synergistic bond activation across the Si=Co bond. Substrate coordination (EtOH, H_2_O) at silicon resulted in the formation of cationic silyl complexes 3.261 and 3.262.

**Scheme 60:**
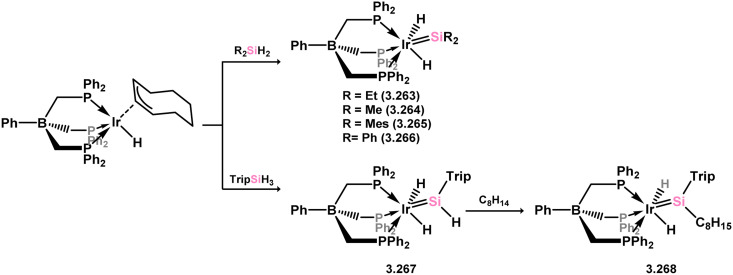
Synthesis of iridium complexes bearing acyclic bis(organyl)- and hydrido-silylene ligands, and the insertion chemistry of the latter towards alkenes.

**Scheme 61:**
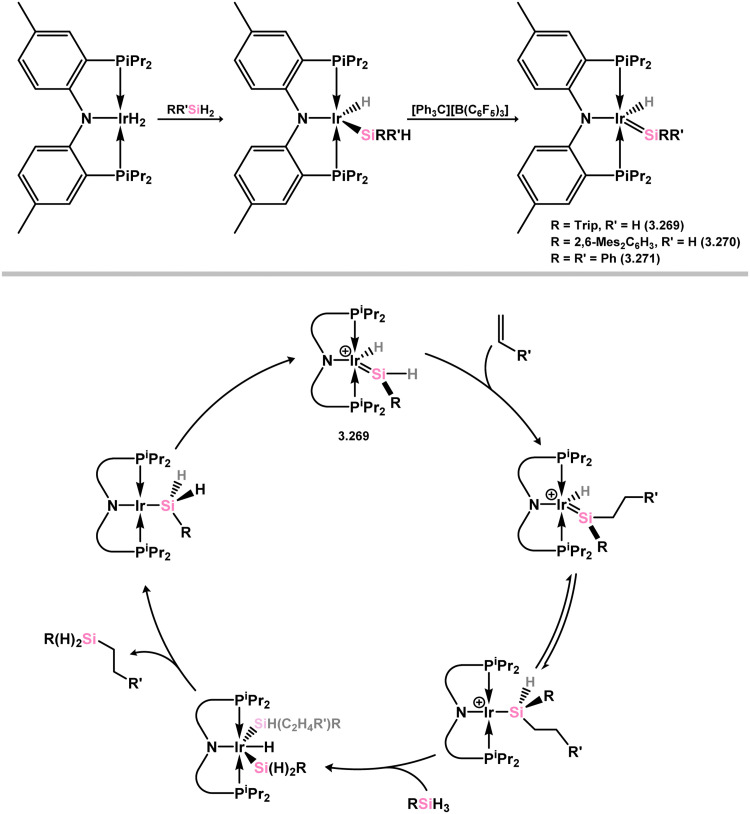
Synthesis of osmium complexes bearing acyclic silylene ligands, *via* salt elimination, and σ-metathesis and hydride migration (above), the one possible alkene hydrosilylation mechanism which involves the Ir and Si centres in 3.269 (below).

The initial example of an acyclic–silylene iridium species involved the deallylation of a tris(phosphino)phenyl borate-stabilised Ir^III^ allyl complex with bis(organyl)silanes, accompanied by Si–H bond migration, leading to alkyl- (3.263 and 3.264) and aryl-silylene (3.265 and 3.266) complexes (Scheme 60).^175,176^ It was also demonstrated that employing TripSiH_3_ as the silane (Trip = 2,4,6-^i^Pr_3_C_6_H_2_) allowed for the synthesis of the (aryl)(hydrido)silylene derivative 3.267, which undergoes alkene insertion at the Si–H bond, akin to the earlier described catalytically active ruthenium complex 3.254, in forming (alkyl)(aryl)silylene complex 3.268. Subsequently, a range of iridium silylene complexes, 3.269, 3.270, and 3.271, were accessible *via* hydride abstraction from iridium silyl complexes (Scheme 61). The hydride-substituted silylene complexes 3.269 and 3.271 exhibited catalytic activity in alkene hydrosilylation, suggesting a second sphere mechanism where alkene insertion into the Si–H bond plays a pivotal role. One such postulated mechanism is outlined in Scheme 61.^177^

**Scheme 62:**
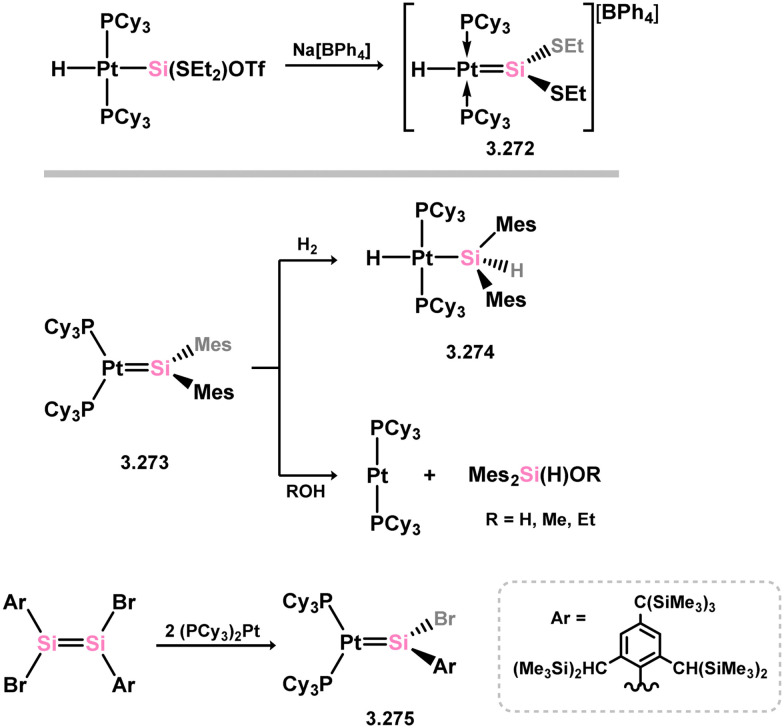
Synthesis of platinum complexes bearing acylic silylene ligands.

One of the earliest reported instances of a TM–silylene complex was the platinum species 3.272, featuring the bis(thionyl)silylene ligand, and was accessed through triflate abstraction from its silyl complex (Scheme 62).^52^ Despite this initial discovery, only a limited number of well-defined examples of acyclic–silylene complexes involving group 10 metals have been reported. Subsequent to the 1993 publication, the neutral platinum silylene complex 3.273 was reported, generated through the *in situ* formation of bis(mesityl)silylene in the presence of [(R_3_P)_2_Pt] (R = ^i^Pr, Cy).^178^ The resulting PCy_3_-stabilised complex exhibited reactivity at the Pt=Si bond, leading to H_2_ cleavage and the formation of silyl complex 3.274, as well as alcohol cleavage to yield Pt^0^ species and alkoxy silanes. A closely related (aryl)(bromo)silylene complex of the [(Cy_3_P)_2_Pt] fragment (3.275) was also obtained *via* the addition of a stable 1,2-diaryl-1,2-dibromo disilene to [(Cy_3_P)_2_Pt].^179^ However, further investigations into the reactivity of this species were not pursued.

**Scheme 63:**
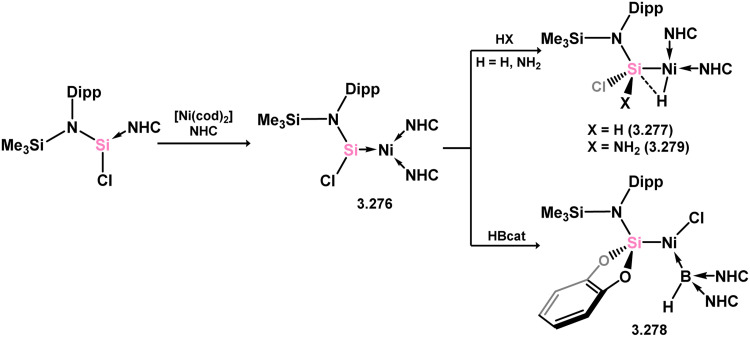
Synthesis and small-molecule activation chemistry of an acyclic–silylene nickel(0) complex.

**Scheme 64:**
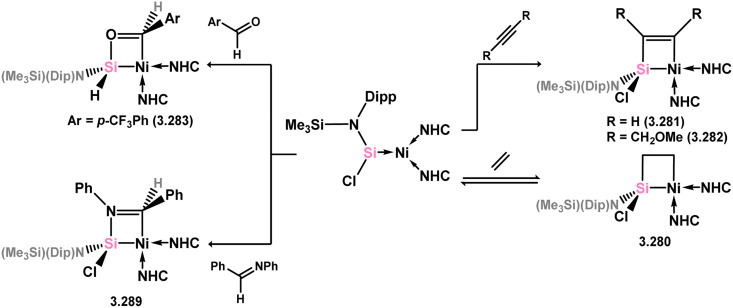
Cycloaddition chemistry of an acyclic–silylene nickel(0) complex.

**Scheme 65:**
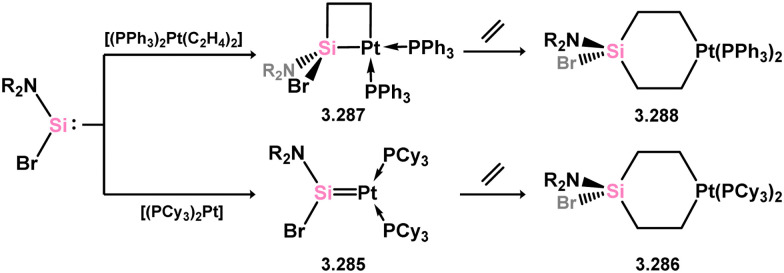
Cycloaddition chemistry of an acyclic–silylene platinum(0) complex.

The acyclic silylene Ni^0^ complex 3.276 was subsequently reported (Scheme 63), synthesised through the combination of NHC-stabilised (amido)(chloro)silylene [{(Dip)(Me_3_Si)N}(Cl)Si·^iPr^NHC] with [Ni(cod)_2_] in the presence of an additional equivalent of ^iPr^NHC.^180^ The resulting 16-electron Ni^0^ complex displayed high reactivity in synergistic bond activation processes. Notably, it demonstrated the capacity to cleave H_2_, similar to platinum complex 3.273, resulting in the formation of silyl complex 3.277. More remarkably, 3.276 cleaves the catechol ligand upon reaction with catechol borane, leading to the formation of the unique Ni^II^ hydroborylene complex 3.278 (Scheme 73). Further studies demonstrated that the chloride ligand in 3.276 could be exchanged through salt-metathesis, yielding a “half parent” bis(amido) silylene complex and various sila-phosphene, -arsene, -phosphinidene, and -arsinidene species (*vide infra*).^181^ Additionally, it was shown that 3.276 could activate ammonia across the Si=Ni bond, resulting in the formation of 3.279, a rare reaction for TM species. Furthermore, this complex also demonstrated the cycloaddition of alkenes (3.280), alkynes (3.281 and 3.282), aldehydes (3.283), and imines (3.284), in all cases forming novel metallacyclic complexes (Scheme 64).^49^ Notably, the reaction with ethylene was found to be reversible. Related chemistry has recently been reported for (amido)(bromo)silylene-Pt^0^ complex 3.285, synthesised *via* the addition of the free (amido)(bromo)silylene [(Cy_3_P)_2_Pt]. This complex reacts with an excess of ethylene in forming the [2+2+2] product 3.286. Alternatively, the free silylene also reacts with [(Ph_3_P)_2_Pt(C_2_H_4_)_2_] in generating the formal [2+2] reaction product, 3.287, which reacts with one addition equivalent of ethylene to form 3.288. Here, ethylene insertion chemistry is not reversible, but, overall, both mono- and bis-ethylene [2+2] cycloadditions are observed at the Si–Pt interface (Scheme 65).^182^

**Scheme 66:**
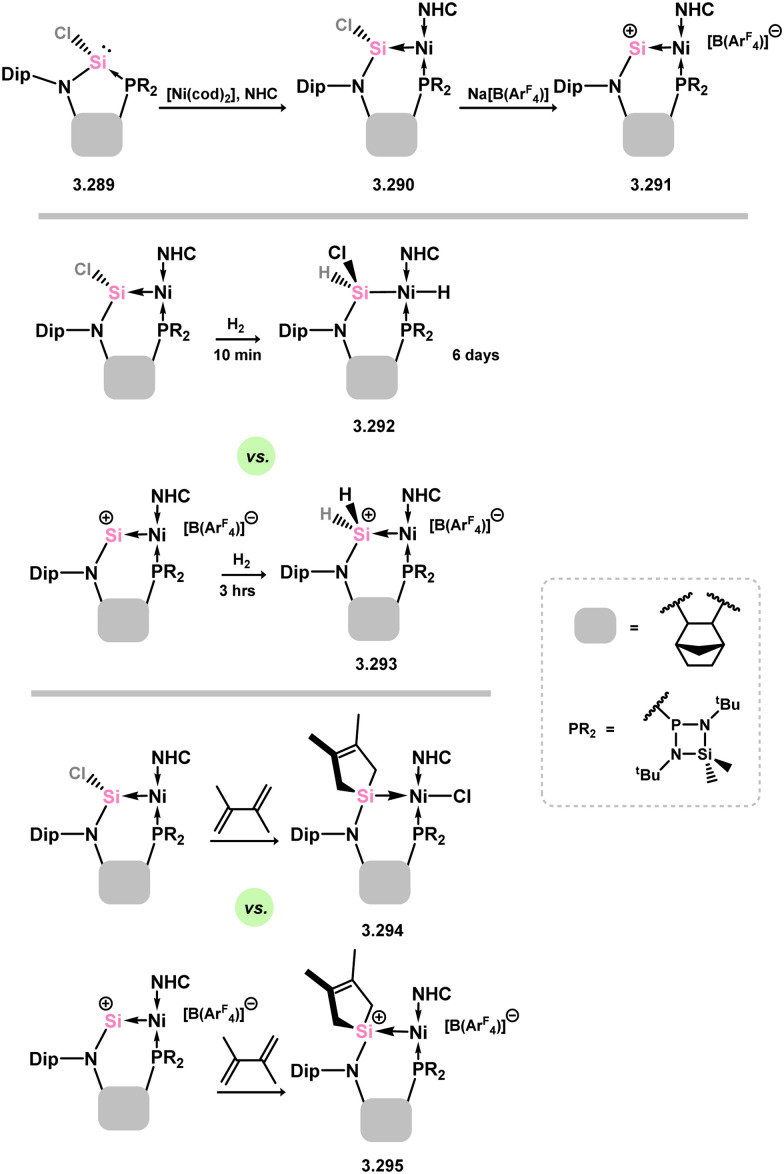
Synthesis and reactivity of neutral and cationic acyclic–silylene nickel(0) featuring a T-shaped nickel centre.

In all silylene systems discussed to this point, the TM centre has borne a ‘classical’ geometry. Recently, Kato and co-workers reported that (amido)(chloro)silylene ligand 3.289 reacts with [Ni(cod)_2_], in the presence of an additional NHC, to form a chelating silylene-ligated Ni^0^ complex 3.290. Though structurally very similar to the ‘non-chelating’ (amido)(chloro)silylene complex 3.276 described above, by being built into a chelating ligand scaffold the Lewis acidic character of the Si^II^ centre becomes more apparent, leading to Z-type ligand behaviour (Scheme 66).^183^ The Ni^0^ centre thus behaves as an L-type ligand towards silicon, this electronic situation borne out by the T-shaped geometry at nickel. Prior to that report, only one T-shaped Ni^0^ complex had been described, featuring the [GeCl_2_] ligand (*vide infra*). Following the initial publication of neutral 3.290, the cationic derivative 3.291 was also reported, accessed through chloride abstraction from 3.290 with Na[BAr_4_^F^].^184^ Cationic 3.291 also holds a Z-type silylene centre, and thus a T-shaped geometry at nickel. Both described complexes are highly reactive, but show rather different characteristics. Neutral complex 3.290 readily activates H_2_, leading to the 1,2-dihydride complex 3.292 through ‘dual-centred’ activation. In contrast, cationic 3.291 behaves akin to a metallosilylene, with H_2_ oxidative addition only at the Si-centre, in forming 3.293. A similar case is observed for MeOTf, whilst the unsaturated species diphenylacetylene and 2,3-dimethyl-1,3-butadiene undergo [2+1] and [4+1] cycloadditions at the silicon centre (*e.g.*3.294 and 3.295, Scheme 66), respectively, in both neutral and cationic systems. This opens the question as to whether cationic 3.291 is better described as a cationic Ni^II^ metallosilylene, which may become more clear through future studies.

To date, no base-free acyclic silylene complexes of group 11 or 12 metals have been reported.

#### 3.1.4. Chelating bis(NHSi) complexes

**Fig. 7:**
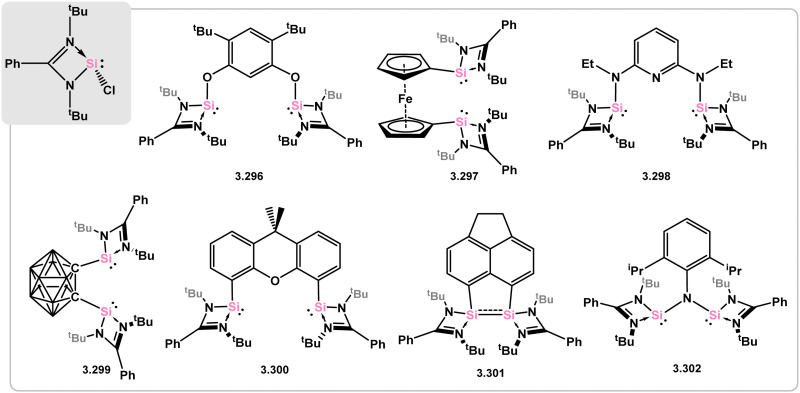
A variety of the known chelating bis(silylene) ligands which are known, utilising the Roesky silylene.

The highly efficient synthesis of the amidinate-stabilised N-heterocyclic chloro silylene 3.1 has since sparked numerous investigations involving salt-metathesis of the Si–Cl bond in this compound, which are generally conveinient, high-yielding processes. As a result, the Driess group has pioneered the development of chelating bis(silylenes), with various examples shown in Fig. 7 denoted as 3.296, 3.297, 3.298, 3.299, 3.300, 3.301, and 3.302. The various potential backbone structures shown here gives rise to a family of chelating ligands possessing different, tunable bite-angles and electronic properties. Several reviews on these compounds have emphasised their ability to act as strong and stable donor ligands in catalytic systems.^61,185^ Additionally, in a few cases, they have been found to actively participate in bond-breaking and bond-forming reactions within a catalytic context. This suggests that this area of research holds significant potential for future development.

**Scheme 67:**
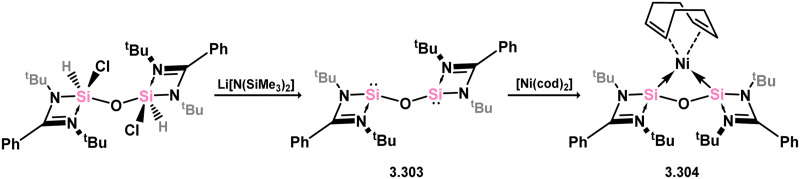
Synthesis of an oxo-bridged bis(silylene) ligand, and its coorindation to nickel(0).

**Scheme 68:**
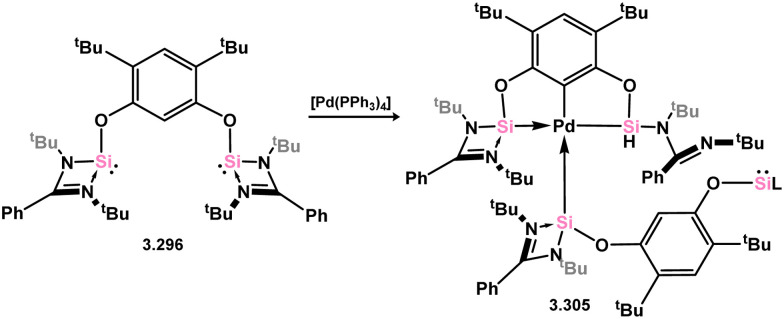
Coordination of a resorcinol-based bis(silylene) ligand to palladium(ii).

The first instance of a chelating bis(silylene) ligand derived from silylene 3.1 did not involve the chloro–silylene itself. Instead, it was synthesised by dehydrochlorination of its disiloxane derivative with Li[N(SiMe_3_)_2_], resulting in the formation of “disilylenoxane” 3.303. This compound was found to readily act as a chelating ligand toward Ni^0^ when reacted with [Ni(cod)_2_], leading to the formation of complex 3.304 (Scheme 67).^186^ Shortly after this initial discovery, it was demonstrated that chloro–silylene 3.1 could be used to easily access chelating bis(silylene) compounds. When reacted with dilithio-resorcinolate, compound 3.296 is efficiently formed. Furthermore, the reaction of this chelating ligand with [Pd(PPh_3_)_4_] resulted in the insertion of the metal into the central aryl C–H bond, followed by hydrogen migration to one of the silylene centers, ultimately generating Pd^II^ complex 3.305. This complex was further stabilised by one silylene center from an additional equivalent of 3.296 (Scheme 68).^187^

**Scheme 69:**
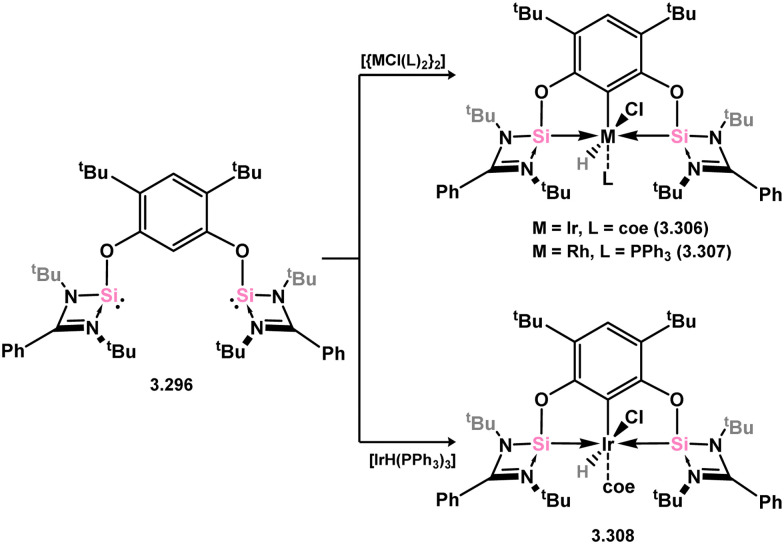
Synthesis of iridium and rhodium complexes bearing a resorcinol-derived bis(silylene) ligand.

**Scheme 70:**
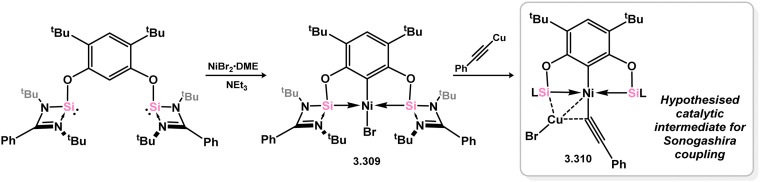
Reactivity of a resorcinol-derived bis(silylene) ligand towards nickel(ii) bromide (inset: Identified bimetallic intermediate in the catalytic Sonogashira coupling).

**Scheme 71:**
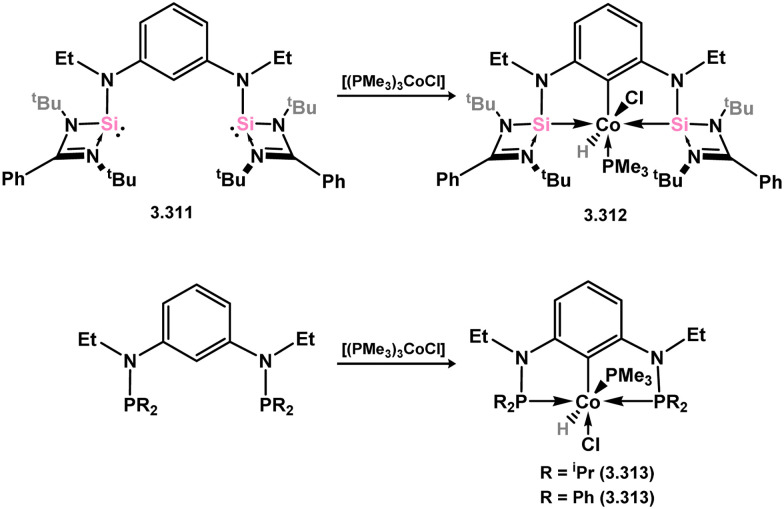
Synthesis of a cobalt complex utilising a *m*-diaminophenyl-derived bis(silylene) ligand, and corresponding phosphine complexes.

The methodology described above was also expanded to include complexes of Ir and Rh (Scheme 69), as well as complexes incorporating closely related chelating Ge^II^ (*vide infra*) and phosphine ligands featuring the resourcinol backbone.^188^ In all cases, no hydrogen migration was observed, thereby preserving the E^II^ character (E = Si, Ge) of the donor centers in the ligands (for Si: 3.306, 3.307, and 3.308). These complexes were found to be highly efficient catalysts for the dehydrogenative borylation of arenes using pinacol borane. This provided the initial evidence that chelating silylene (and germylene) ligands exhibit significantly stronger σ-donating capabilities compared to their phosphine counterparts. The same ligands derived from resorcinol were employed in Ni-catalysed Sonogashira cross-coupling reactions, utilising Ni^II^ bis(silylene) complex 3.309 (Scheme 70).^189^ It is worthy of note that the heterobimetallic complex 3.310 was isolated through introduction of a copper acetylide to 3.309, suggesting its crucial role as an intermediate in the catalytic reaction mechanism. More recently, the *m*-phenylenediamine derivative, 3.311, was reported, and Co^III^ monohydride complexes accessed through oxidative addition of Co^I^ into the C–H bond of the central arene (3.312, Scheme 71).^190^ Though the bis(silylene) ligated complex was an active catalyst for alkene hydrosilylation, it was in fact less active in this chemistry than the related bis(phosphine) ligated complexes 3.313 and 3.314.

**Scheme 72:**
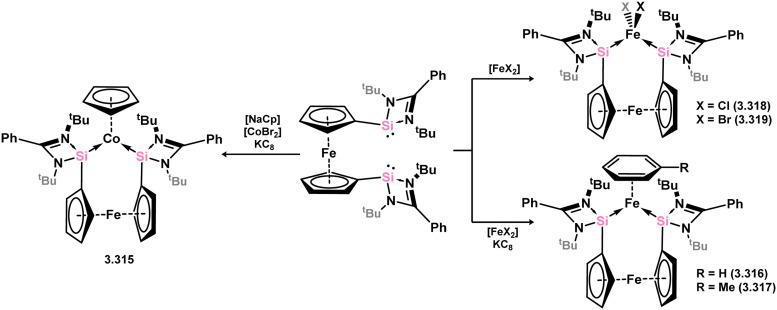
Formation of iron(ii), iron(0), and cobalt(i) complexes bearing a ferrocene-derived bis(silylene) ligand.

The ferrocene-bridged bis-silylene 3.297 was successfully synthesised by reacting di(lithio)ferrocene with chloro–silylene 3.1. In the initial publication on this ligand, it was demonstrated that the reaction with *in situ* generated [CpCo] resulted in the formation of the Co^I^ complex 3.315 (Scheme 72).^191^ This complex was utilised as a catalyst for the cyclo-trimerisation of phenylacetylene and acetonitrile, expanding the scope of transformations achievable with this ligand class. Subsequently, the same bis-silylene ligand was employed to stabilise novel Fe^0^ arene complexes 3.316 and 3.317, as well as Fe^II^ dihalide complexes 3.318 and 3.319. The Fe^II^ complex 3.316 proved to be an effective catalyst for the hydrogenation of ketones.^192^

**Scheme 73:**
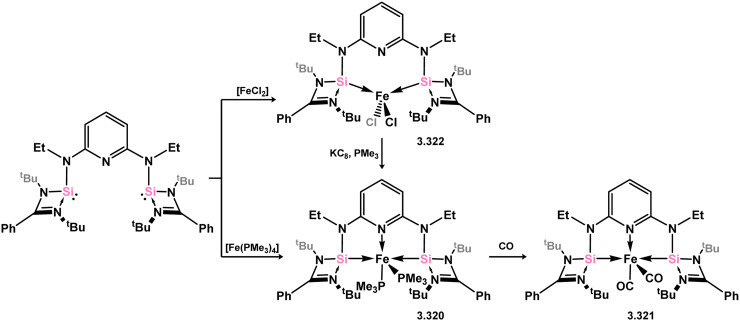
Formaition of iron(ii) and iron(0) complexes bearing a bis(amino)pyridine-derived bis(silylene) ligand.

**Scheme 74:**
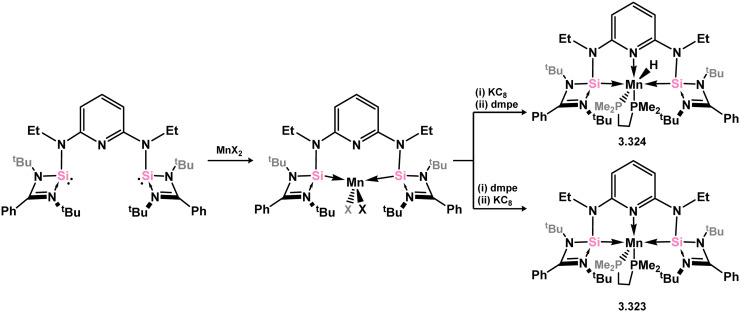
Formaition of managnese(ii), manganese(i), and manganese(0) complexes bearing a bis(amino)pyridine-derived bis(silylene) ligand.

The pyridine-derived pincer ligand 3.298, which is closely related to the resorcinol derivative 2.396, can be readily synthesised *via* a salt-metathesis reaction involving chloro–silylene 3.1. In the initial publication regarding Fe^II^ and Fe^0^ complexes, it was observed that the coordination of the pyridine moiety to iron depended on the oxidation state of the metal center. The ligand exhibited tridentate coordination in Fe^0^ complexes 3.320 and 3.321, while no N → Fe donation was observed in Fe^II^ complex 3.322 (Scheme 73).^193^ Bis(trimethylphosphine) complex 3.320 served as a catalyst for the hydrosilylation of ketones, with lower catalyst loadings required compared to ferrocene-derived bis-silylene complex 3.316 for comparable transformations (*vide infra*). Pyridine-supported bis(silylene) 2.298 was also later shown to stabilised Mn^0^ in complex 3.323, as well as Mn^I^ species 3.324, carbonyl-free examples of which are rare. The former open-shell species was shown to be an active catalyst for the selective 1,2-hydroboration of pyridines (Scheme 74).^194^

**Scheme 75:**
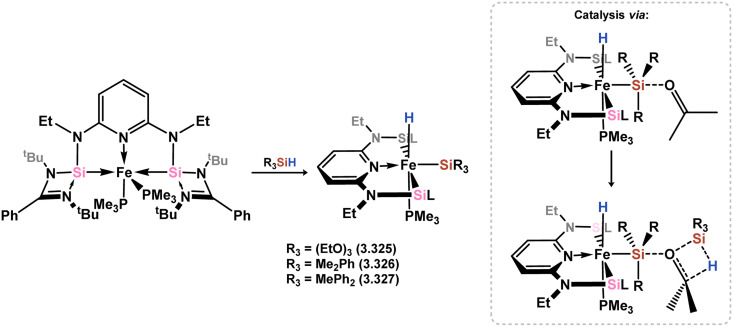
The reactivity of an iron(0) bis(silylene) complex towards silanes, and proposed intermediates in the catalytic hydrosilylation of ketones.

A subsequent publication revealed that the Fe^0^ complex 3.320 undergoes oxidative addition of silanes to yield octahedral complexes 3.325, 3.326, and 3.327, which was proposed as the initial step in hydrosilylation catalysis (Scheme 75).^195^ Further investigations using density functional theory (DFT) calculations indicated that a “peripheral” mechanism, where the incoming ketone attacks the silyl-Si center followed by the formation of a 4-membered transition complex at the activated ketone with a second equivalent of silane, was energetically favorable for hydrosilylation.

**Scheme 76:**
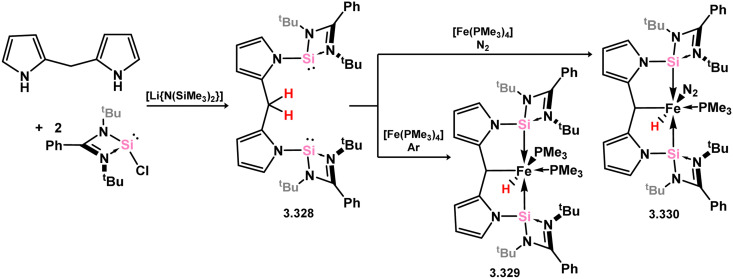
Synthesis and iron complexation of a bis(pyrrole)-derived bis(silylene).

**Scheme 77:**
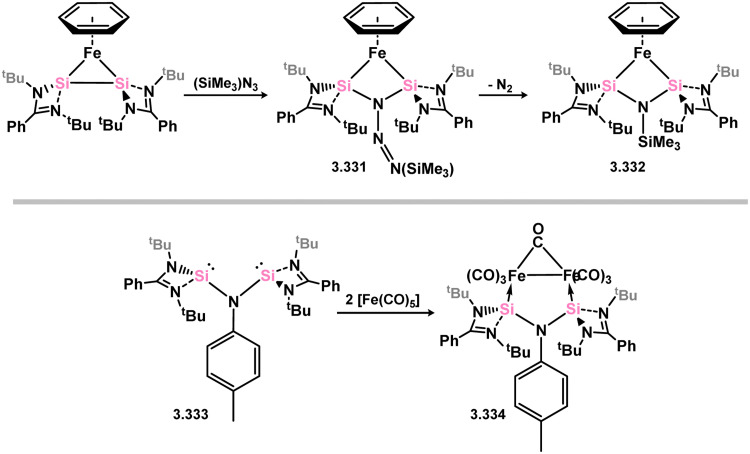
Iron complexes of RN-appended bis(silylene) ligands.

More recently, further examples of chelating bis(silylene)-complexed iron species have been reported. The first involved the development of a novel pyrollyl-derived bis(silylene) ligand, 3.328, which reacts with [Fe(PMe_3_)_4_] to yield hydrido-Fe^II^ complex 3.329. This species was shown to undergo PMe_3_-N_2_ exchange (*viz.*3.330), and as such was also investigated for the catalytic silylation of N_2_ (Scheme 76).^196^ Here, up to 74 turnovers could be observed, being comparable to the relatively small number of molecular catalysts which can achieve this reaction.^95^ An additional example of an Fe^0^ complex has also been reported, accessed rather uniquely *via* small-molecule activation by a ‘disilyne’ complex of iron (Scheme 77). Addition of Me_3_SiN_3_ leads to scission of the Si–Si bond, and formation of azide-bridged 3.331. This complex eliminates N_2_, finally forming [(Me_3_Si)N]-bridged 3.332,^197^ the bis(silylene) ligand backbone of which is not dissimilar to that recently reported by Driess and co-workers.^198^ Indeed, it was recently reported by Roesky and co-workers that this bis(silyenyl)aniline (*viz.*3.333) reacts with [Fe(CO)_5_].^198,199^ Given the tight bite angle in this bis(silylene) ligand, the resulting complex (*viz.*3.334) contains the [Fe_2_(CO)_7_] unit, featuring a formal Fe–Fe interaction.

**Scheme 78:**
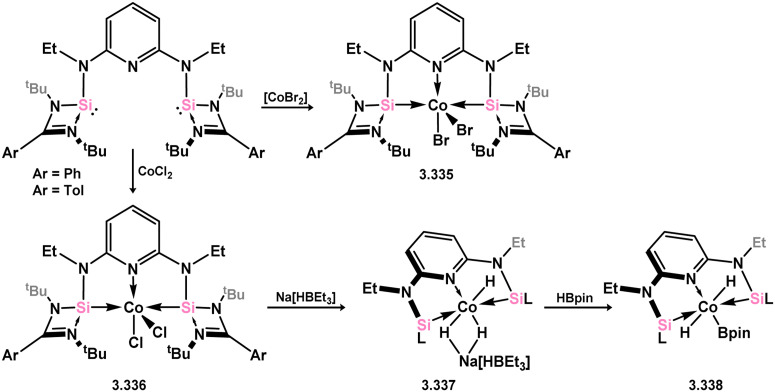
Formaition of cobalt(ii) complexes bearing bis(amino)pyridine-derived bis(silylene) ligands.

Cobalt complexes of pyridine-derived bis-silylene ligand 3.298 have been developed and successfully applied in the C–H borylation of arenes and heterocycles. In the initial report, the cobalt dibromide complex 3.335 was utilised as the catalyst, and an additional hydride source, Na[BEt_3_H], was employed to generate the active cobalt hydride catalyst *in situ* (Scheme 78).^200^ A subsequent publication demonstrated that the dichloro-complex 3.336 could be utilised for the generation of stable hydride complex 3.337, while the (hydrido)(boryl)cobalt complex 3.338 could be subsequently generated through reaction with HBpin.^201^ A detailed study was conducted to explore the potential reaction mechanism, which goes beyond the scope of this review. However, it was proposed that a Co^I^–Co^III^ redox cycle may be involved, especially as no evidence of non-innocent ligand behavior was observed.

**Scheme 79:**
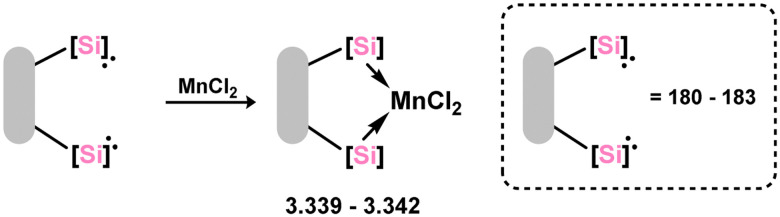
Synthesis of known manganese(ii) dichloride complexes of bis(silylene) ligands.

The carborane-derived bis-silylene ligand 3.299, similar to examples described earlier, was synthesised through a straightforward salt-metathesis reaction and was utilised in the nickel-catalysed Buchwald-Hartwig amination of halo-arenes. The high donor strength of the ligand, imparted by the electron-rich carborane moiety, contributed to impressive reaction rates. Manganese dichloride complexes of this bis-silylene ligand, as well as the ligands discussed above (*viz.*3.339, 3.340, 3.341, and 3.342, Scheme 79), were also reported and used in the transfer semi-hydrogenation of alkynes to *E*-alkenes.^202^

**Scheme 80:**
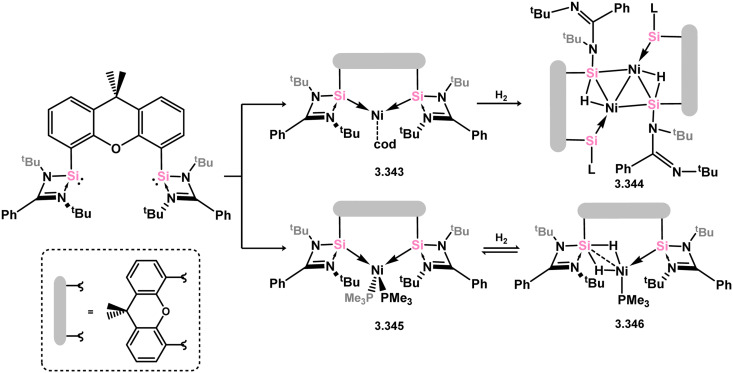
Synthesis of nickel(0) complexes supported by a xanthene-derived bis(silylene) ligand, and their reactivity towards H_2_.

Utilising novel xanthene-derived ligand 3.300, Ni^0^ complexes could be readily accessed which were shown to be catalytically active in alkene hydrogenation.^203^ The wide bite-angle of this ligand resulted in remarkable catalytic activity, similar to the well-known XantPhos ligand.^204,205^ In this case, the Ni^0^ complex 3.343 was synthesised and shown to catalyse the hydrogenation of unactivated alkenes, including the challenging tetramethylethylene, at ambient temperature and 1 bar H_2_. It was suggested that the ligand exhibits non-innocent behavior, with the 3.343 forming the silyl complex 3.344 upon reaction with H_2_. Notably, the related bis(trimethylphosphine) complex 3.345 undergoes *reversible* reaction with H_2_, leading to the mixed-valence complex 3.346, incorporating one silyl and one silylene center (Scheme 80).

**Scheme 81:**
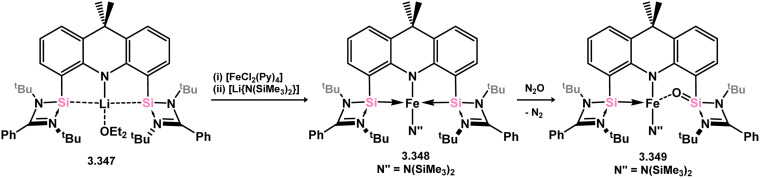
Synthesis of iron(ii) complexes supported by and anionic 9,9-dimethylacridine-derived bis(silylene) ligand, and subsequent oxidation of one silylene centre.

As an adjunct to the established xanthene derived bis(silylene), Mo and co-workers have developed the 9,10-dihydroacridine derived bis(silylene) 3.347, which was utilised in the formation of Fe^II^ complex 3.348 (Scheme 81).^206^ This complex was shown to undergo oxidation with N_2_O, yielding 3.349, referred to as an iron-silanone complex, with one oxygen atom bridging the Fe and a single Si centre. Further, 3.348 was found to be an active catalyst for the reduction of N_2_O with HBpin, in the formation of N_2_, H_2_, and (pinB)_2_O.

**Scheme 82:**
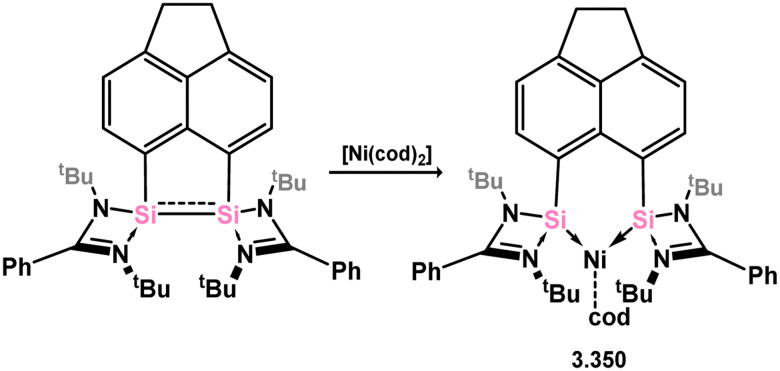
Formation of a bis(silylene) nickel(0) complex through the addition of a acenapthene-derived disilene to [Ni(cod)_2_].

**Scheme 83:**
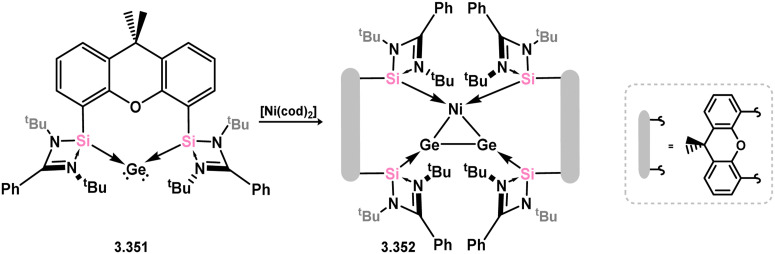
Formation of a unique [NiGe_2_] moiety through the addition of a bis(silylene)-supported germylone to [Ni(cod)_2_].

**Scheme 84:**
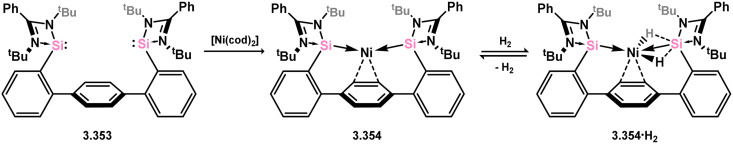
Formation of a nickel(0) complex of a terphenyl-derived bis(silylene) ligand.

Three additional examples of Ni^0^ complexes incorporating chelating bis-silylene ligands have been reported. The first, namely acenaphthene-derivived 3.350, is obtained through the addition of disilene 3.301 to [Ni(cod)_2_], resulting in insertion into the Si–Si bond (Scheme 82).^207^ The second example was obtained through reaction of the bis(silylene)-stabilised germylone 3.351 with [Ni(cod)_2_], leading to a unique complex in which two bis-silylene ligands stabilise a [Ge_2_Ni] three-membered ring (*viz.*3.352, Scheme 83).^208^ Most recently, the terphenyl bis(silylene) ligand 3.353 was developed, and its Ni^0^ complex accessed.^209^ Here, the central aryl ring of the ligand backbone acts as an additional ligand towards nickel (Scheme 84). Complex 3.354 was employed in the catalytic hydrogenation of alkenes, with reversible H_2_ activation shown to occur at one Ni–Si interface in the formation of 3.354·H_2_.

**Scheme 85:**
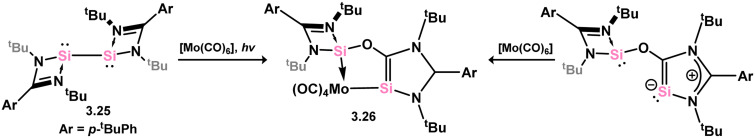
The addition of an Si–Si bonded bis(silylene) to [Mo(CO)_6_], leading to CO-activation and Mo-chelation.

Finally, the only reported group 6 complex bearing a bis(silylene) ligand was recently reported by Jones and co-workers, through the reaction of bis(silylene) 3.25 with [Mo(CO)_6_], under blue light irradiation, forming 3.26.^82^ The ‘free’ bis(silylene) could also be accessed by activation of gaseous CO, which subsequently reacts with [Mo(CO)_6_] (Scheme 85).

The diverse range of complexes obtained using chelating bis-silylene ligands, which includes both chemical curiosities and active catalysts, suggests that this area of research will continue to flourish in the coming years. This should further enhance our understanding of bonding in these complexes and enable the development of tailored catalysts for key catalytic processes. Particularly noteworthy are complexes where the low-valent silicon centre exhibits non-innocent behaviour, a concept that is just beginning to be explored in the broader context of tetrylene ligation.

### 3.2. Germylene – transition metal chemistry

#### 3.2.1. N-heterocyclic germylenes

This area of tetrylene chemistry is not yet so deeply explored as that for silicon. Still, as already mentioned germylene ligands hold significant promise as effective activating ligands in molecular catalytic systems.^62^ It should also be expected that germylene ligands are more readily accessible, given that divalent germanium precursors are readily available (*e.g.* [dioxane·GeCl_2_]).

##### 4-Membered

**Scheme 86:**
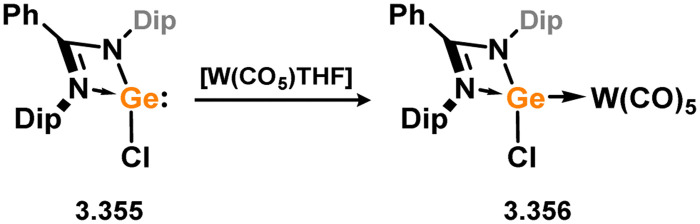
Synthesis of a tungsten complex bearing an (amidinato)(chloro)germylene ligand.

**Scheme 87:**
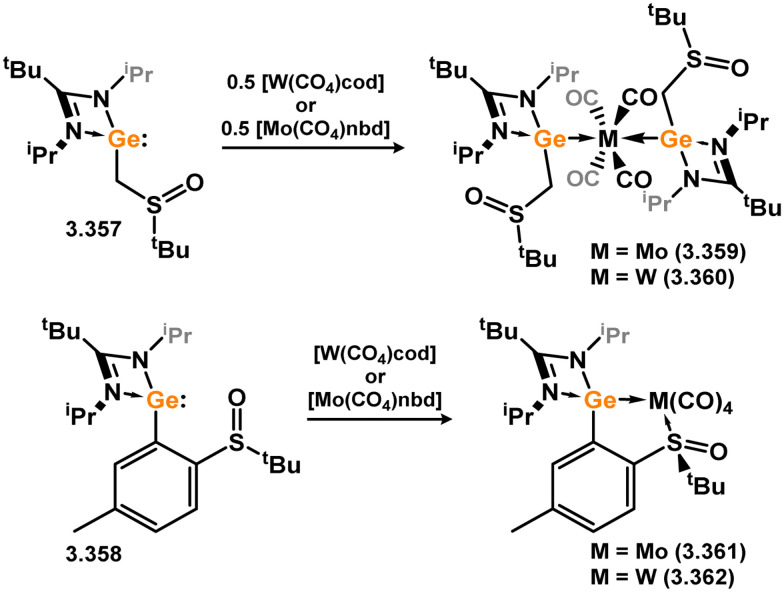
Synthesis of a tungsten and molybdenum complexes bearing sulphoxide-functionalised amidinato-germylene ligands.

As yet, no 4-membered NHGes are known for groups 3–5. The first investigation into related group 6 chemistry involved germylene 3.355, which bears a considerably bulkier amidinate ligand when compared to the commonly utilised Roesky silylene. This attests to the larger ionic radius of Ge^II^, relative to Si^II^. The tungsten complex of germylene 3.356 could be readily accessed in the THF displacement reaction [THF·W(CO)_5_] (Scheme 86).^210^ Later, sulphoxide-appended derivatives of 4-membered NHGes (*viz.*3.357 and 3.358) were developed,^211,212^ and their coordination chemistry towards [M(CO)_4_] fragments explored (3.359, 3.360, 3.361, and 3.362, Scheme 87). Here, chelating ligand motifs were targeted, whereby both germylene and sulphoxide fragments would bind the metal centre. This was found to be possible utilising the aryl-appended system 3.358.

**Scheme 88:**
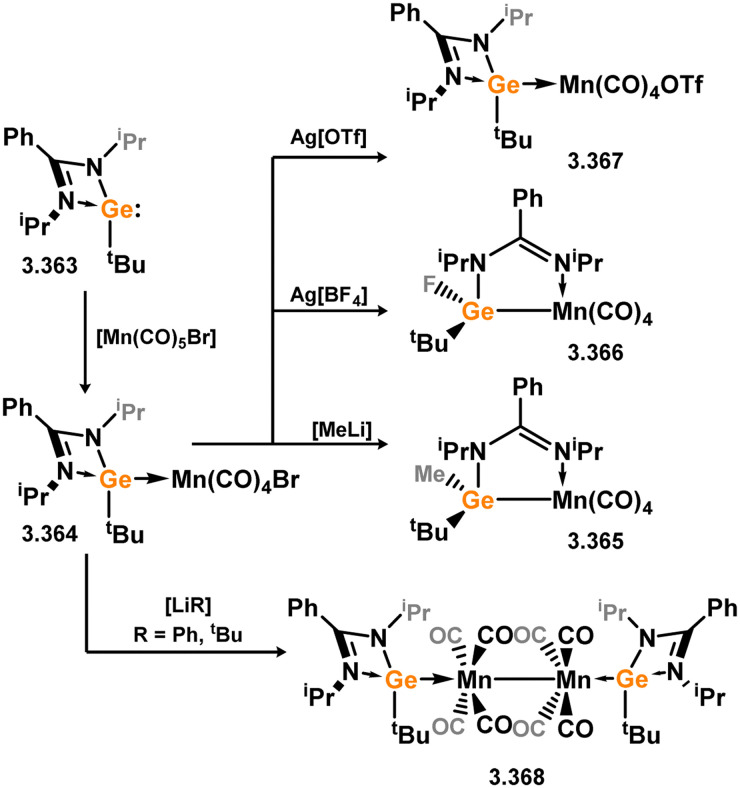
Synthesis and further modification of a manganese complex bearing an (amidinato)(alkyl)germylene ligand.

There are also a small handful of complexes for the group 7 metals, largely reported by the group of Cabeza and García-Álvarez. Using their developed germylene 3.363, the [Mn(CO)_4_Br] complex 3.364 was readily accessed through reaction with [Mn(CO)_5_Br] (Scheme 88).^213^ Bromide exchange at Mn interestingly led to the formation of formal germyl complexes, through group migration to Ge, in formation of methyl- and fluoro-germyl complexes 3.365 and 3.366. Exchange with the triflate group generated stable Mn^I^-triflate complex 3.367, the Ge^II^ centre maintaining its tetrylene form. It was later shown that similar bromide-exchange with Ph or ^*t*^Bu groups rather led to C–C coupling in reductive elimination of those groups, generating the dimanganeses octacarbonyl fragment [Mn_2_(CO)_8_], stabilised by two germylene ligands (*viz.*3.368, Scheme 88).^214^ This could also be accessed *via* reaction of the free germylene with [Mn_2_(CO)_10_]. In the same submission, it was also shown that one CO-ligand could also be exchanged by PMe_3_ or ^*t*^BuNC.

**Scheme 89:**
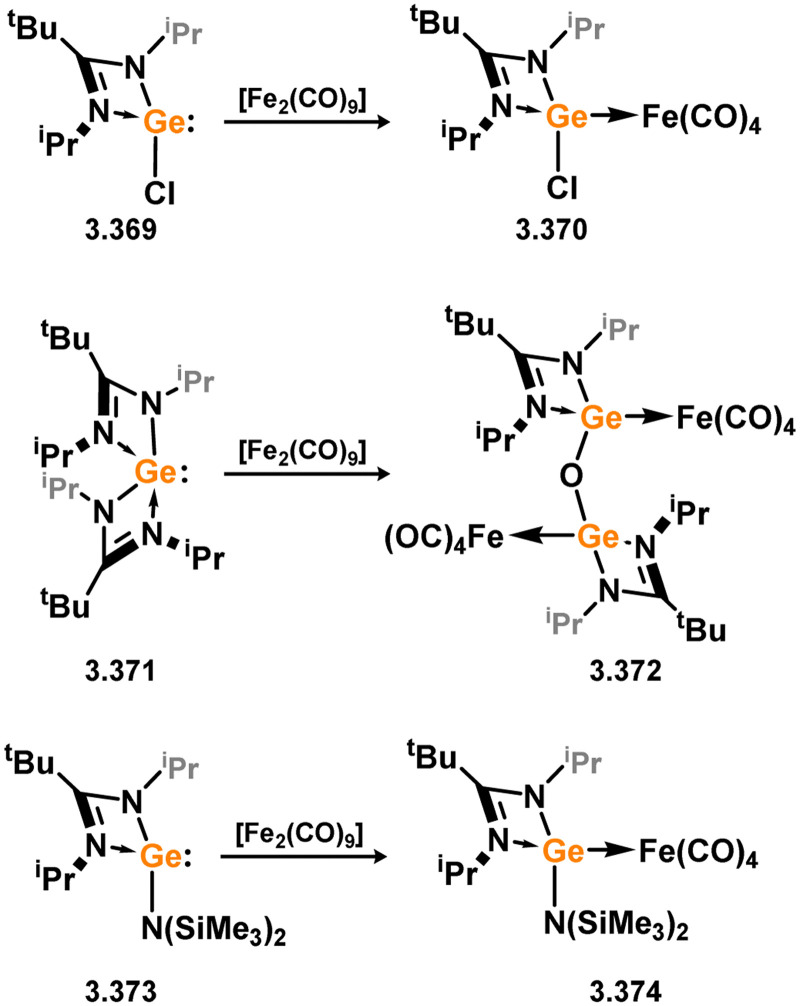
Access to a range of iron(0) carbonyl complexes bearing amidinato-germylene ligands.

**Scheme 90:**
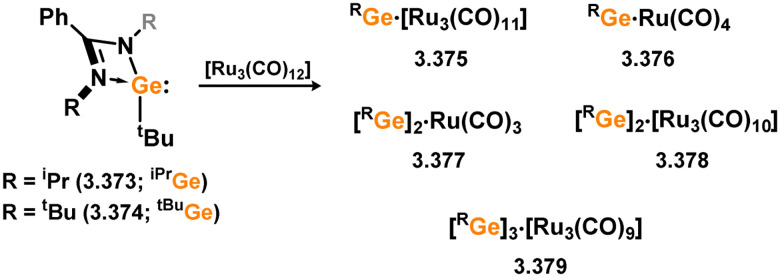
Synthesis of differing ruthenium(0) carbonyl complexes bearing (amidinato)(alkyl)germylene ligands.

**Scheme 91:**
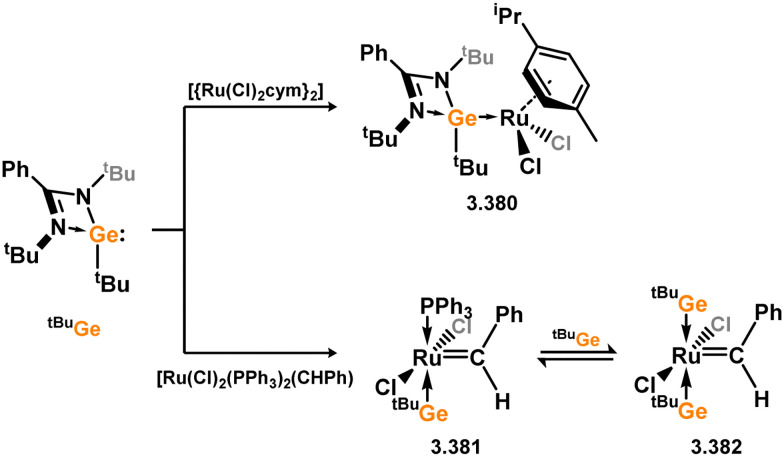
Synthesis of varying ruthenium complexes bearing an (amidinato)(alkyl)germylene ligand.

The first example of group 8 NHGe complexes was reported by Cabeza and García-Álvarez, through addition of their chloro-germylene 3.369 to [Fe_2_(CO)_9_], in the loss of [Fe(CO)_5_] forming 3.370 (Scheme 89).^215^ It was also shown that the bis(amidinato)germylene 3.371 reacts with the same iron(0) precursor to form the bis(germylene) complex 3.372, thought to proceed *via* the reaction of an intermediary [Fe(CO)_4_] adduct of 3.371 with adventitious moisture. The same group later reported related chemistry for the ruthenium trimer, [Ru_3_(CO)_12_], involving the two (alkyl)(amidinato)germylenes 3.373 and 3.374 (Scheme 90).^216^ Here, a selection of mono-, bis-, and tris-metallic species were observed and isolated (*viz.*3.375, 3.376, 3.377, 3.378, and 3.379). Further Ru species were accessed in subsequent studies, in the addition of (alkyl)(amidinato)germylenes to [(cym)RuCl_2_]_2_ (cym = 1-Me-4-^i^PrC_6_H_4_) and [(Cy_3_P)_2_Cl_2_RuC(H)Ph], leading to dimer scission (*viz.*3.380) or phosphine-exchange (*viz.*3.381 and 3.382; Scheme 91).^217,218^ The former species 3.380 was utilised in catalytic transfer hydrogenation, amination of alcohols, and the full deuteration of aromatic aldehydes, demonstrating the utility of germylenes as ligands. In addition, in reactions of germylenes with [(Cy_3_P)_2_Cl_2_RuC(H)Ph] both mono- and bis-ligation was observed, with the equilibrium being dependant on the alkyl group at the Ge centre.

**Scheme 92:**
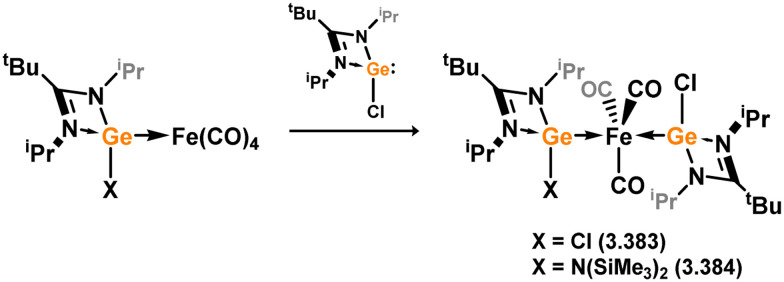
Synthesis of unsymmetrical bis(amidinato-germylene) iron(0) complexes.

**Scheme 93:**
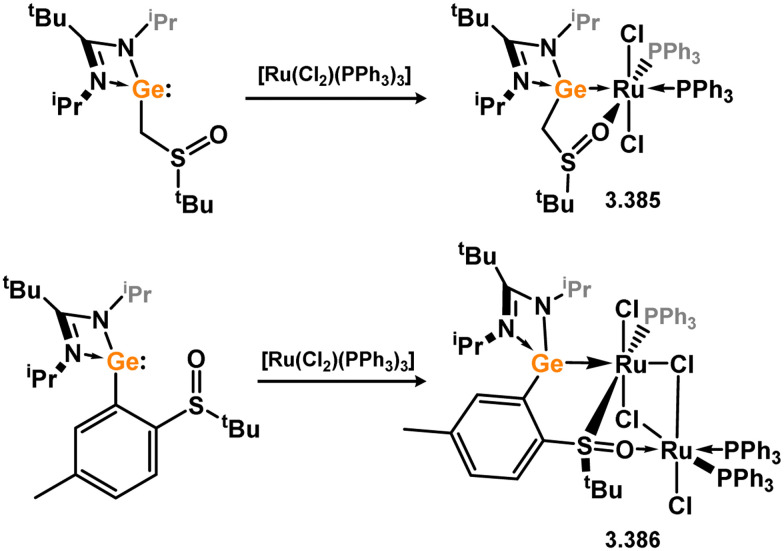
Synthesis of a ruthenium complexes bearing sulphoxide-functionalised amidinato-germylene ligands.

Above described complexes 3.370 and 3.74 have been developed as well-defined molecular precursors for [GeFe] nanoparticles.^219^ These species were shown to decompose under mild conditions (200 and 100 °C, respectively) in the presence of additional coordinating ligands, to form the targeted nanoparticles. Notably the latter bulky amido system, which decomposes at much lower temperatures, was shown to lead to a narrower size distribution in formed nanoparticles, giving insights into the controllability of such processes. The bis(germylene) complexes 3.383 and 3.384 were later introduced by the same group, and shown to achieve related nanoparticle formation, albeit under more forcing conditions of 300 °C (Scheme 92).^91^ The same group has also reported Ru complexes of their earlier described sulphoxide-functionalised germylene ligands, 3.385 and 3.386 (Scheme 93).^211,212^ Here, both mono- and bimetallic Ru complexes could be realised, depending on the nature of the linker moiety in the ligand systems. These are rather rare examples of non-symmetrical chelating ligands which incorporate a germylene centre, which are presently becoming considerably more popular.

**Scheme 94:**
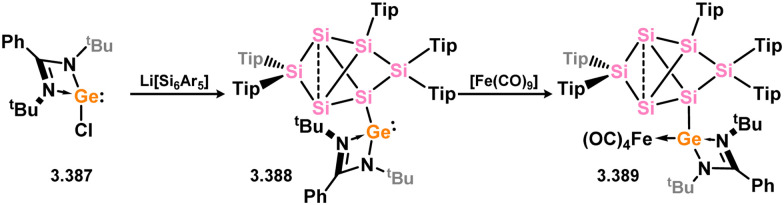
Functionalisation of an Si_6_ cluster with an amidinato-germylene ligand, and its coordination to an iron(0) fragment.

**Scheme 95:**
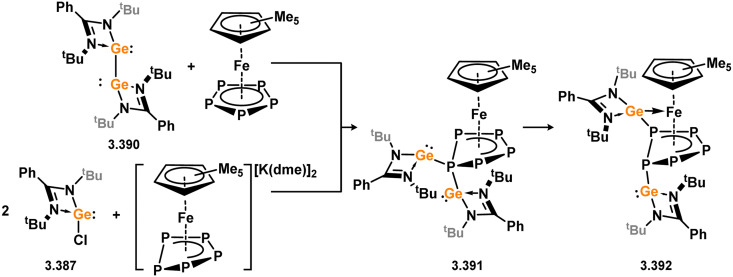
Functionalisation of a P_5_-ferrocenyl derivative with a Ge–Ge bonded bis(germylene), leading a formal iron germylene complex.

**Scheme 96:**
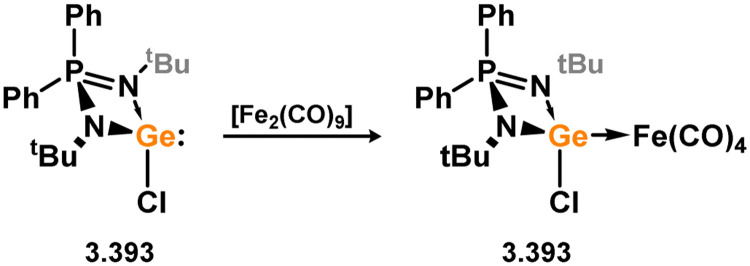
Coordination of an iminophosphonamido-germylene to an iron(0) fragment.

Examples of more exotically functionalised 4-membered NHGes have been reported, featuring a Si_6_ siliconoid cluster (Scheme 94), and a P_5_ ring system (Scheme 95).^220,221^ The former germylene ligand system was accessed through the salt-metathesis of the chloro-germylene 3.387 with the [R_5_Si_6_]Li, as has been described for the closely related Roseky silylene, forming 3.388. Again, as per the functionalised silylene systems, addition of [Fe_2_(CO)_9_] led to complexation of the [Fe(CO)_4_] at Ge (3.389). The described P_5_ system was accessed either through addition of the bis(germylene) 3.390 to [Cp*FeP_5_], or the chlorogermylene 3.387 to the reduced [Cp*FeP_5_][K(dme)]_2_. In both cases, initially the two germylene centres bind one P-centre (*viz.*3.391). A subsequent 1,2-migration of a single germylene ligand leads to complex 3.392, in which one germylene binds the Fe centre. A final example of an iron-complex featuring a 4-membered NHGe is known, utilising the unique iminophosphonamide ligand 3.393, which as with many examples already described, reacts with [Fe_2_(CO)_9_] in the elimination of [Fe(CO)_5_] and complexation of the [Fe(CO)_4_] fragment (3.394, Scheme 96).^222^

**Scheme 97:**
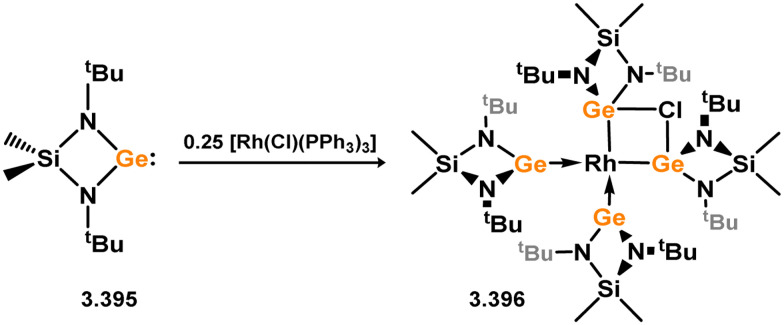
Reactivity of a 4-membered NHGe towards a ruthenium(i) complex.

**Scheme 98:**
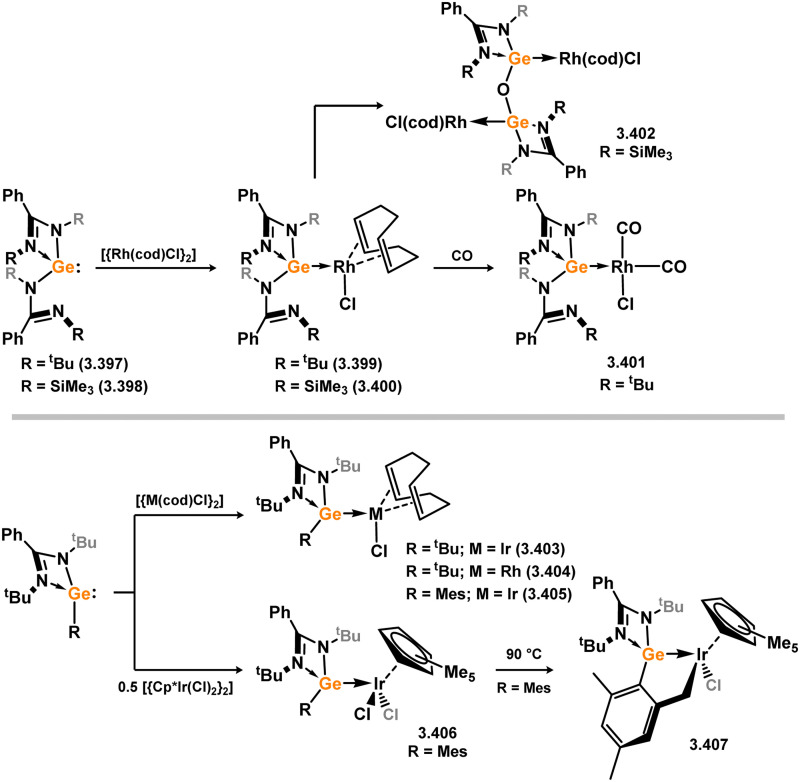
Reactivity of various amidinato-germylenes towards iridium and ruthenium species.

The initial example of a group 9 complex featuring a 4-membered NHGe did not employ an amidinate-stabilised germylene, but rather the cyclic bis(amido)germylene 3.395 (Scheme 97).^223^ Four equiv. of this ligand react with [Rh(PPh_3_)_3_Cl], in displacing all three PPh_3_ ligands, and forming 3.396 in which the Rh–Cl bond is activated, the Cl^−^ ligand now bridging two Ge centres. Castel *and* co-workers later reported on several Rh complexes featuring the bis(amidinato)germylenes 3.397 and 3.398.^224^ These were accessed through initial addition of the germylene ligands to [{Rh(cod)Cl}_2_], in forming 3.399 and 3.400, with a subsequent CO complex generated through cod-exchange with CO gas, yielding 3.401, and presumably the presence of adventitious moisture leading to 3.402 (Scheme 98). Cabeza, Àlvarez-García and co-workers have also reported both Rh and Ir complexes (*viz.*3.403 and 3.404), which were accessed in a similar manner (*i.e.* utilising [{M(cod)Cl}_2_]).^217^ The closely related mesityl-substituted germylene undergoes similar reactivity, forming 3.405, in addition to ready complexation with the [CpIrCl_2_] fragment, forming 3.406. This latter species undergoes C–H activation of one Me group of the mesityl ligand upon heating at 90 °C, in forming complex 3.407.^225^ This reactivity is reminiscent of C–H activation processes in the Si congeners of these species (*vide supra*) which occurs under much more mild conditions, giving some indications as to the potential greater stability of germylene complexes, which may be important in catalytic systems.

**Scheme 99:**
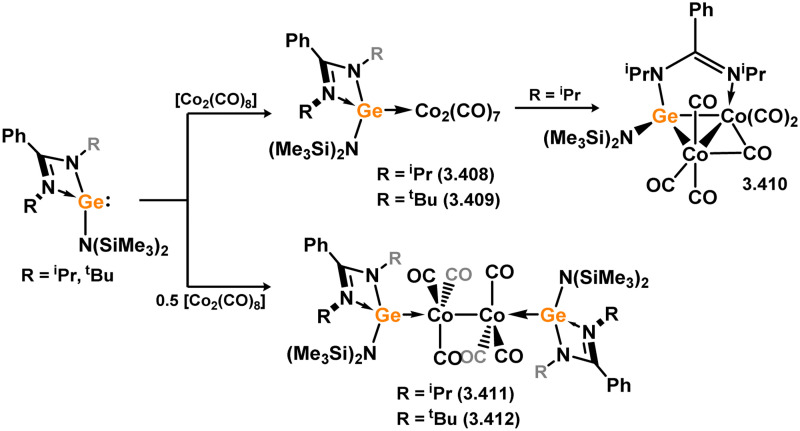
Formation of [Co_2_] complexes supported by amidinato-germylene ligands.

**Scheme 100:**
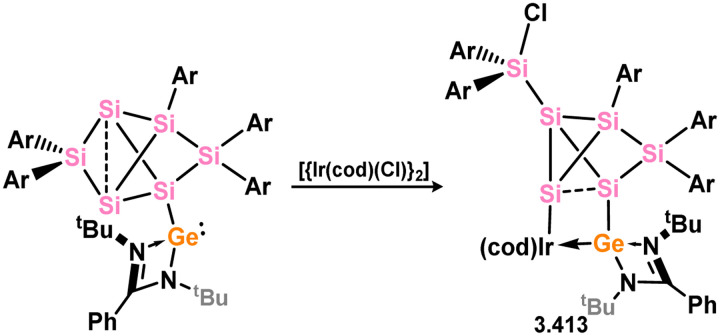
Reactivity of an Si_6_-bound amidinato-germylene towards an iridium(i) species.

A small number of bimetallic cobalt complexes are known, accessed *via* dimeric [Co_2_(CO)_8_], and incorporating either one or two germylene ligands (3.408, 3.409, 3.410, 3.411, and 3.412, Scheme 99),^226^ with 3.410 demonstrating that the amidinate ligand can bridge the Ge and Co centres, giving a further example of fluctional binding of this ligand class. The siliconoid-functionalised germylene 3.387 has also been shown to react with [(cod)IrCl]_2_, leading to activation of the Ir–Cl bond at the Si-cluster, and chelation by the germylene ligand in forming 3.413 (Scheme 100).^102^

**Scheme 101:**
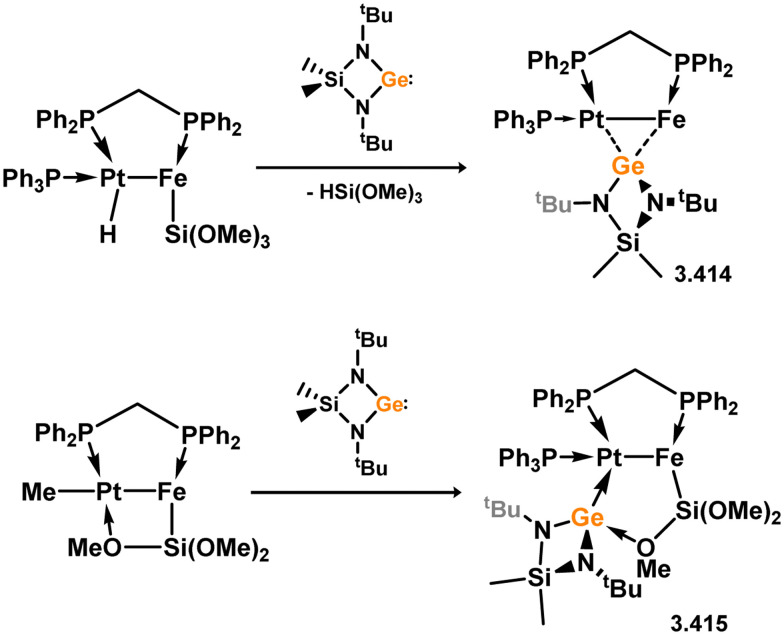
Reactivity of a 4-membered NHGe towards a bimetallic [FePt] complex.

The seminal examples of group 10 elements bonded by 4-membered NHGes again utilised cyclic bis(amido)germylene 3.395, and the heterobimetallic [FePt] compound [(CO)_3_{(MeO)_3_Si}Fe(μ-dppm)Pt(H)(PPh_3_)] (Scheme 101).^227^ This led to the elimination of (MeO)_3_SiH, with the germylene bridging the Fe and Pt centres in 3.414, though this was not crystallographically confirmed and rather assigned by multi-nuclear NMR spectroscopic studies. Reaction with the related [(CO)_3_{(MeO)_3_Si}Fe(μ-dppm)Pt(Me)], in which the Pt centre is intramolecularly stabilised by one OMe group, leads to insertion of the germylene into this Pt⋯O interaction, in the formation of the novel base-stabilised germylene–Pt complex 3.415, which could be structurally characterised.

**Scheme 102:**
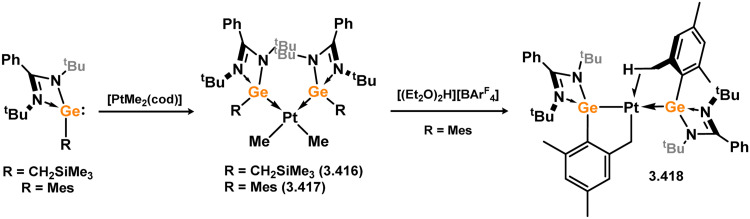
Formation of a platinum(ii) complexes bearing an amidinato-germylene, and subsequent C–H activation of one ligand.

Cabeza, García-Àlvarez and co-workers have also shown that their alkyl and mesityl substituted germylenes undergo (cod)-exchange reactions with [(cod)PtMe_2_], giving bis(germylene) complexes 3.416 and 3.417. Akin to the previously discussed silylene congener (*vide supra*), mesityl-substituted 3.417 reacts with [(Et_2_O)_2_H][BAr^F^_4_] to yield C–H activation product 3.418 (Scheme 102).^228^

**Scheme 103:**
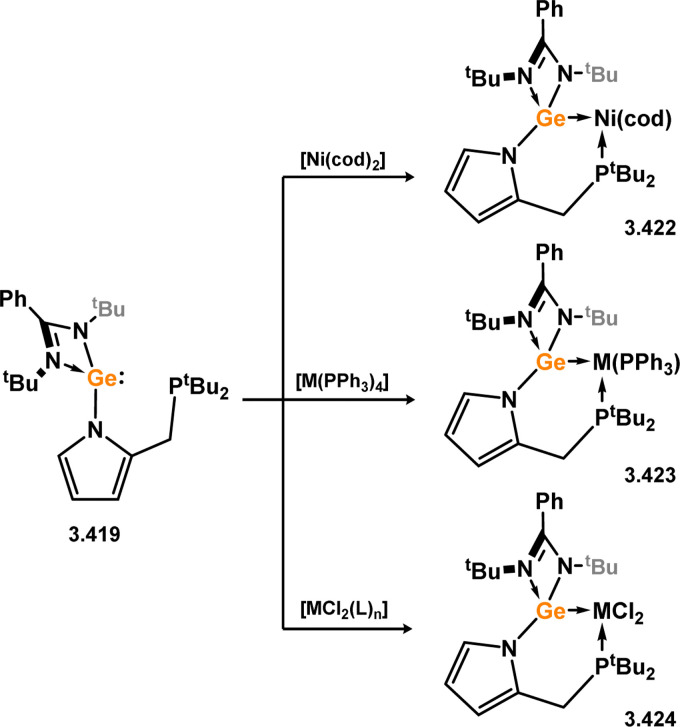
Reactivity of a chelating phosphine-functionalised amidinato-germylene towards group 10 metal fragments.

**Scheme 104:**
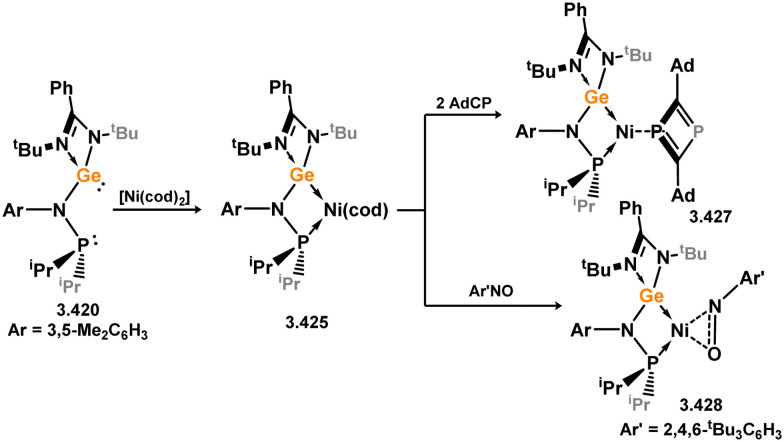
Reactivity of a chelating phosphine-functionalised amidinato-germylene towards nickel(0), and subsequent reactivity.

**Scheme 105:**
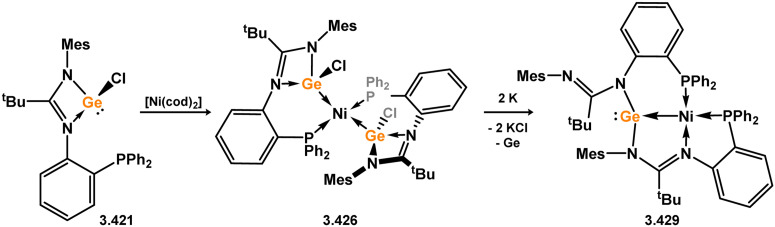
Formation of a Z-type germylene ligand through reduction of a bis(amidinato-germylene) complex.

**Scheme 106:**
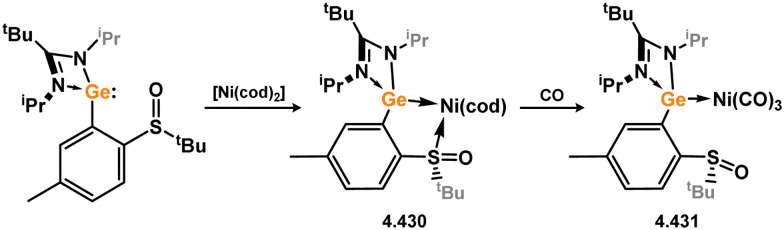
Reactivity of a sulphoxide-functionalised amidinato-germylene towards nickel(0).

Three examples of phosphine-functionalised (amidinato)germylenes have been reported (3.419, 3.420, and 3.421), for which group 10 complexes are known (*viz.*3.422, 3.423, 3.424, 3.425, and 3.426; Scheme 103–106), all generated through direct combination with M^0^ or M^II^ species.^229–231^ The further chemistry of Ni^0^-complexed (phospha-aniline)germylene 3.425 demonstrated exclusive reactivity at nickel, in phosphalkyne dimerisation, and ArNO coordination (3.427 and 3.428, respectively; Scheme 104). Of particular note is the further chemistry of 3.426: reduction of this complex leads to elimination of elemental germanium, and formation of a novel germylene–Ni^0^ complex in which the germylene now behaves as a Z-type ligand, receiving donation from the Ni centre in complex 3.429 (Scheme 105).^229^ The earlier described sulphoxide-functionalised germylene ligand 3.358 has also been shown to react with [Ni(cod)_2_], in the formation of the ligand-chelated Ni^0^ system 3.430, which is converted to the corresponding ‘un-chelated’ carbonyl complex 3.431 when reacted with CO.^212^

**Scheme 107:**
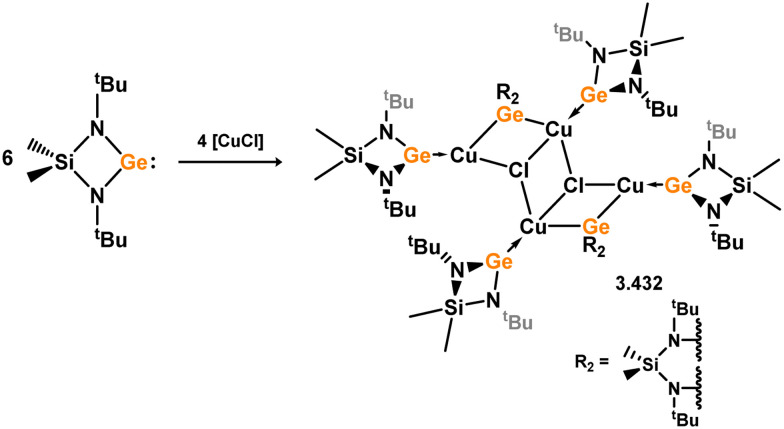
Reactivity of a 4-membered NHGe towards copper(i).

**Scheme 108:**
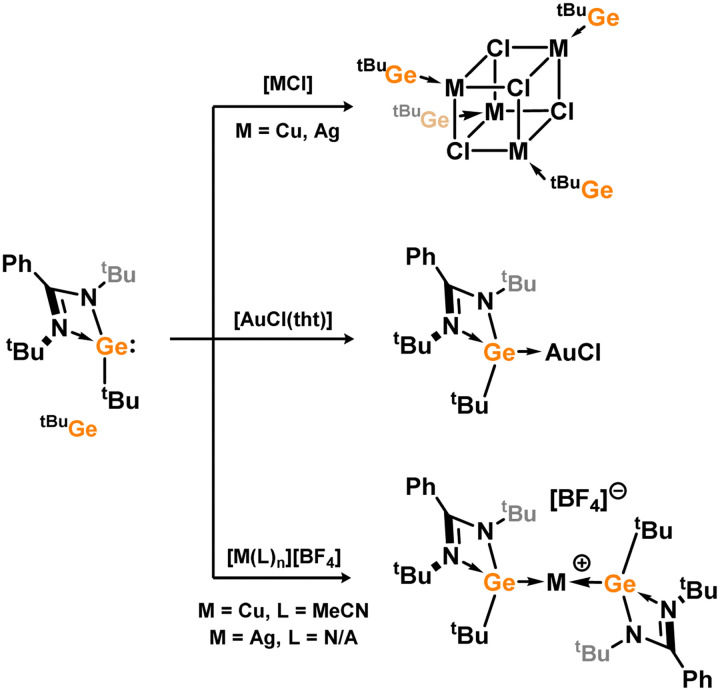
Reactivity of an (amidinato)(alkyl)germylene towards various coinage metal fragments.

**Scheme 109:**
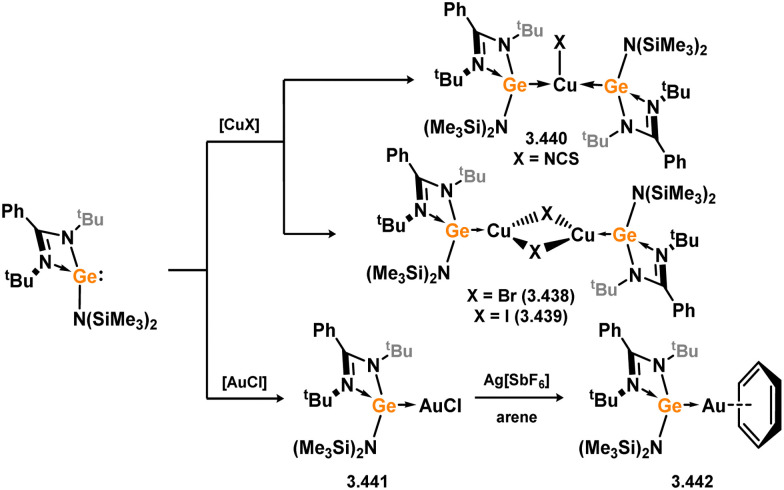
Chelating NHSi (and NHGe) ligands in the synthesis of well-defined Ni(ii) catalytic complexes.

The bis(amido) NHGe 3.395 has been shown to react with [CuCl] in the formation of the small cluster complex, featuring 6 germylene ligands (4 terminal, two bridging), and 4 [CuCl] fragments (3.432, Scheme 107).^232^ The group of Cabeza and García-Àlvarez have reported on Cu, Ag, and Au halide complexes featuring their (alkyl)(amidinato)germylene 3.374, through straight-forward addition of the ligand to the free halides (*viz.*3.433, 3.434, and 3.435, Scheme 108). For Cu and Ag, reaction with metal tetrafluoroborate salts led to the formation of pseudo-two-coordinate coinage metal complexes 3.436 and 3.437.^233^ The group of Kahn has also reported a handful of coinage metal complexes utilising a bulky amide functionalised germylene (Scheme 109). Here, copper halide (3.438 and 3.439), copper thiocyanate (3.440), gold chloride (3.441), and arene-stabilised gold cation (3.442) complexes have been reported.^234,235^ The utility of latter complexes in ‘click’ chemistry is noteworthy here, which again demonstrates application of these readily accessible heavier group 14 ligands in catalysis.

**Scheme 110:**
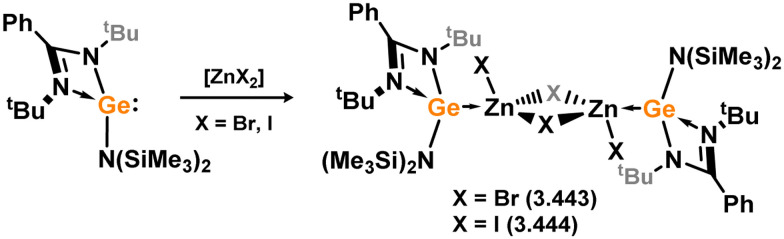
Synthesis of zinc complexes bearing an amidinato-germylene.

Only two complex are known for group 12, both of which employ the above described bulky amide-substituted germylene, which reacts with ZnX_2_ (X = Br, I) to yield dimeric adducts 3.443 and 3.444 (Scheme 110).^236^

##### 5-Membered

**Fig. 8:**
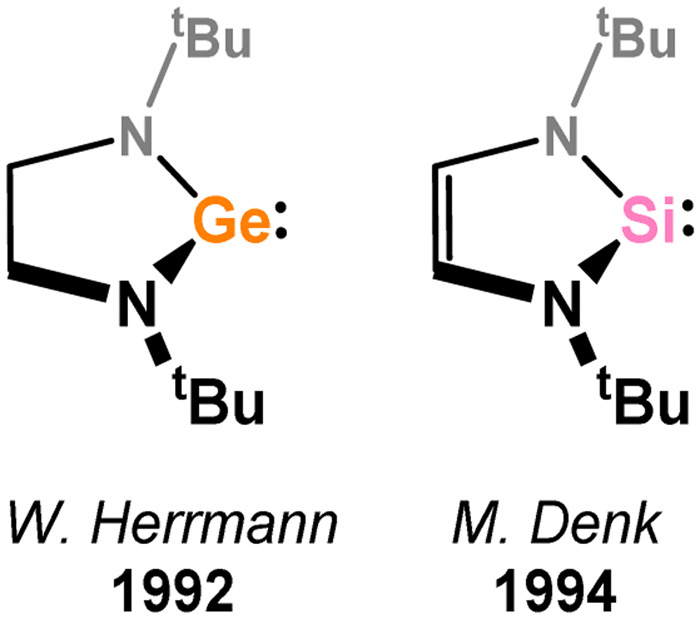
The first reported example of a 5-membered NHGe, compared to its silicon congener.

The first example of a 5-membered NHGe was in fact reported prior to the initial example of the silicon derivative, in 1992 from the group of Hermann.^237^ It is perhaps not surprising, though, that this study involved Denk, who later reported the first NHSi in 1995 (Fig. 8).^58^ Since the first report of an NHGe their numbers have grown to surpass that of NHSis, presumably owing to the ready accessibility of the Ge^II^ oxidation state (*viz.* [dioxane·GeCl_2_]).

**Scheme 111:**
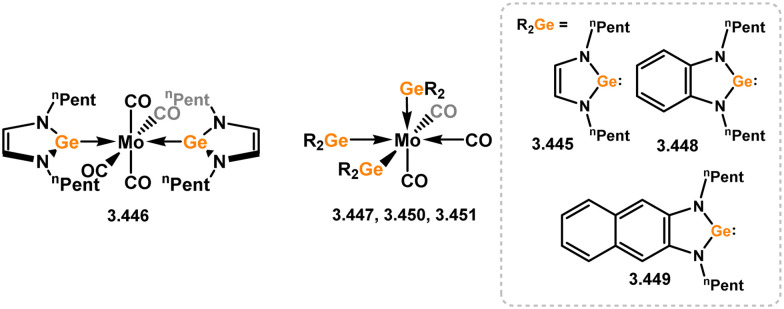
Formation of various molybdenum(0) complexes bearing 5-membered NHGe ligands.

**Scheme 112:**
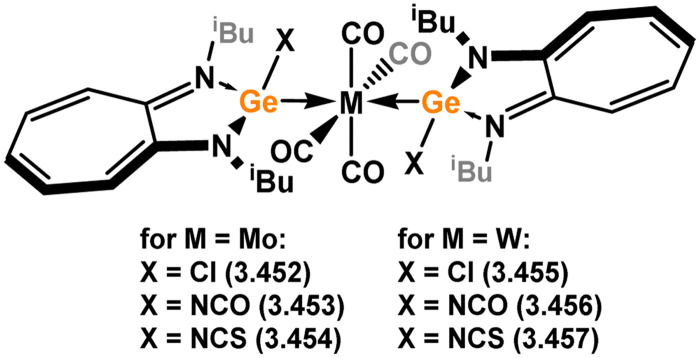
Synthesis of molybdenum and tungsten complexes bearing tropiminoato–germylene ligands.

No 5-membered NHGe complexes are known for groups 3–5. The first complexes for group 6 were forthcoming in 2002, utilising the *n*-pentyl substituents at N (*viz.*3.445 in Scheme 111). Reaction of two equiv. with [Mo(CO)_4_(NCEt)_2_] led to mixtures of bis- and tris-germylene complexes 3.446 and 3.447 (Scheme 111).^238^ The phenylenediamine and napthalenediamine-derived germylenes 3.448 and 3.449 were later shown to react in a 3 : 1 ratio with [(η^3^-CHT)Mo(CO)_3_] (CHT = cycloheptatriene), forming octahedral tris(germylene)molybdenum complexes 3.450 and 3.451.^239^ Monoanionic tropiminoate ligands have also been employed in generating 5-membered NHGes, initially the (chloro)germylene, from which the chloride can be exchanged for a range of *pseudo*-halides. From these, a range of bis(germylene) tungsten and molybdenum complexes have been accessed through direct addition of the ligands to [M(CO)_4_(cod)] (*viz.*3.452, 3.453, 3.454, 3.455, 3.456, and 3.457; Scheme 112).^240^ In all cases, formed complexes are octahedral.

**Scheme 113:**
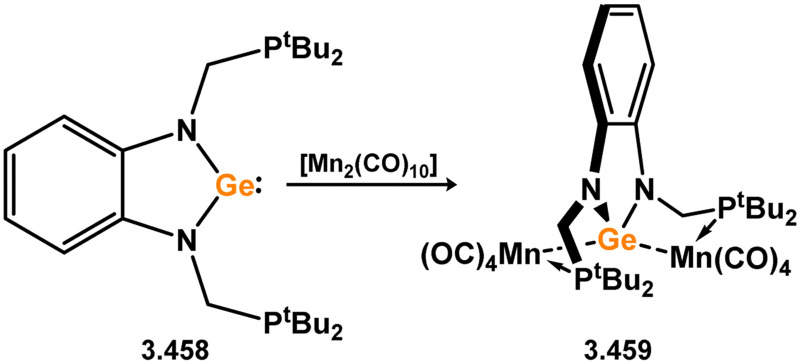
Reaction of a manganese(0) carbonyl complex towards a chelating phosphine-functionalised 5-membered NHGe ligand.

A single example of a group 7 complex featuring a 5-membered NHGe is reported, utilising phosphine-appended NHGe 3.458. The ligand reacts with [Mn_2_(CO)_10_] at 110 °C in elimination of two equiv. CO, and formation of bimetallic manganese complex 3.459 (Scheme 113).^241^

**Scheme 114:**
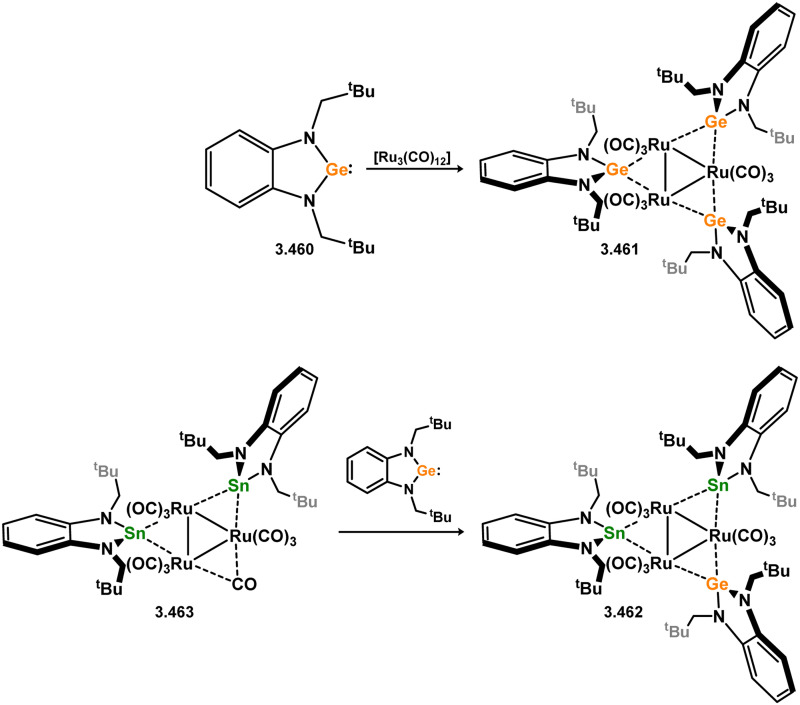
Formation of a ruthenium(0) trimer supported by a 5-membered NHGe ligand.

**Scheme 115:**
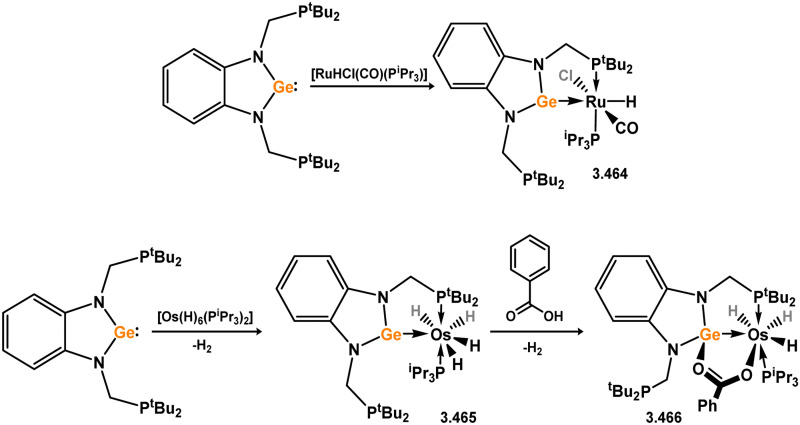
Reactions of ruthenium and osmium species towards phosphine-functionalised 5-membered NHGe ligands.

Numerous complexes featuring group 8 metals stabilised by 5-membered NHGes have been reported. In an initial report, it was shown that germylene 3.460 reacts with [Ru_3_(CO)_12_] at 100 °C to yield the trimeric ruthenium complex 3.461 (Scheme 114).^242^ The same group later showed that the mixed stannylene-germylene trimeric ruthenium complex 3.462 can be accessed *via* a similar route, employing the bis(stannylene) complex 3.463.^243^ The phosphine-appended NHGe already described (*viz.*3.458) was shown to react with [RuHCl(CO)(P^i^Pr_3_)_2_], forming complex 3.464 in one phosphine arm chelates the Ru centre, and the germylene centre remains as a classical σ-donor (*i.e.* no insertion into the Ru–Cl/H bonds is observed; Scheme 115).^241^ The same phosphine-appended NHGe ligand was subsequently reported to undergo a unique reactivity with [OsH_6_(^i^Pr_3_P)_2_], in generating complex 3.465. Here, one phosphine arm is cleaved, in loss of HP^*t*^Bu_2_. This species was found to be an active catalyst for the dehydrogenation of formic acid. Importantly, 3.465 reacts with carboxylic acids to form carboxylate-bridged species 3.466, giving some evidence that the germylene centre's non-innocence is key in the reaction mechanism.^244^

**Scheme 116:**
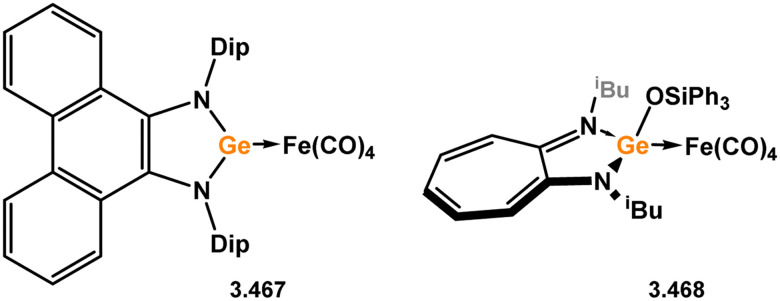
Iron(0) complexes bearing 5-membered NHGe ligands.

Related to the above described NHGes, 9,10-phenanthrendiimine derived NHGe was recently reported, and shown to form a stable complex with [Fe(CO)_4_] in reaction with [Fe(CO)_9_] (3.467, Scheme 116).^245^ Interestingly, the same carbonyl displacement reaction was not possible with the tin congener. Extending this NHGe chemistry to the tropiminoate systems, the (siloxy)(tropiminoate)germylene was shown to form a stable complex with [Fe(CO)_4_] in 3.468, whilst this was not possible with the closely related thiosilyl germylene.^246^

**Scheme 117:**
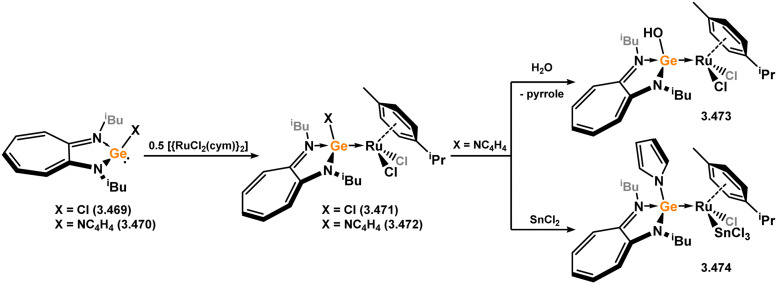
Ruthenium complexes bearing tropiminoato–germylene ligands.

In a subsequent study, it was shown that tropiminoato–germylene ligands 3.469 and 3.470 react with [RuCl_2_(*p*-cumene)]_2_ to yield complexes 3.471 and 3.472 through dimer cleavage (Scheme 117). The pyrrolidine-functionalised system reacts with water under metathesis of the Ge–N bond, in forming hydroxyl-germylene complex 3.473, whilst reaction with [SnCl_2_] led exclusively to insertion into one Ru–Cl bond (*viz.*3.474).^247^

**Scheme 118:**
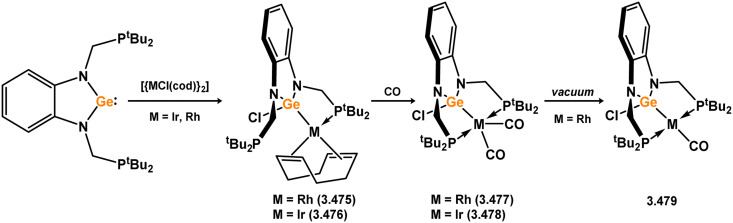
Reactivity of a phosphine-functionalised 5-membered NHGe ligand towards iridium and rhodium species.

**Scheme 119:**
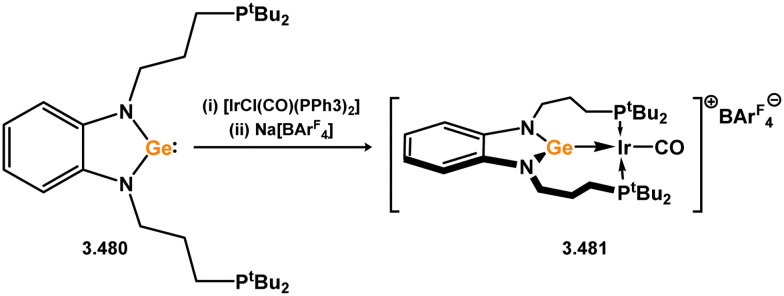
Formation of a formal 5-memebered NHGe complex of iridium, employing extended phosphine chelating arms.

**Scheme 120:**
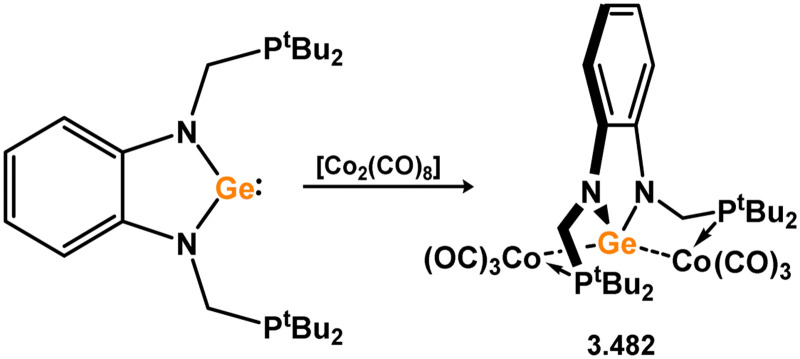
Reactivity of a phosphine-functionalised 5-membered NHGe ligand a cobalt(0) carbonyl species.

Formation of group 9 metal complexes bearing NHGes has proved more challenging to access due to the insertion of the Ge centre into M–Cl bonds (M = Rh, Ir) when a free NHGe is reacted with [MCl(cod)_2_] (Scheme 118).^241,248^ For both rhodium and iridium, reaction of these precursors with pincer germylene 3.460 generates (chloro)(bisamido)germyl complexes, 3.475 and 3.476. Reaction with gaseous CO leads to exchange of the cod ligand by two equiv. CO (*viz.*3.477 and 3.478), with the rhodium derivative eliminating one CO when placed under vacuum, forming 3.479. It was very recently shown by Campos and co-workers that the related NHGe 3.480, with extended phosphine tethers (*i.e.* C_3_H_6_ in place of CH_2_) led to similar M–Cl insertion chemistry in reaction with [IrCl(CO)(PPh_3_)_2_] (Scheme 119).^249^ However, chloride abstraction from this species leads to planarisation at Ge, in forming a classic σ-donating germylene ligand in 3.481. As per the NHGe–Os complex 3.465 already described, NHGe–Ir complex 3.481 is an active catalyst for the dehydrogenation of formic acid. One example of a cobalt complex bearing an NHGe is known, namely 3.482, which was accessed by the addition of phosphine appended ligand 3.460 to [Co_2_(CO)_8_] (Scheme 120).^248^3.482 is a bimetallic system, in which two [Co(CO)_3_] fragments are coordinated at Ge.

**Scheme 121:**
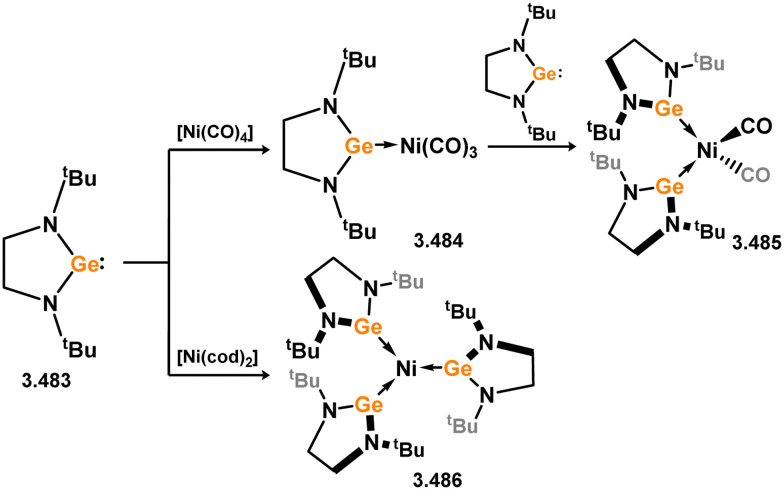
Formation of nickel(0) complexes bearing 5-membered NHGe ligands.

**Scheme 122:**
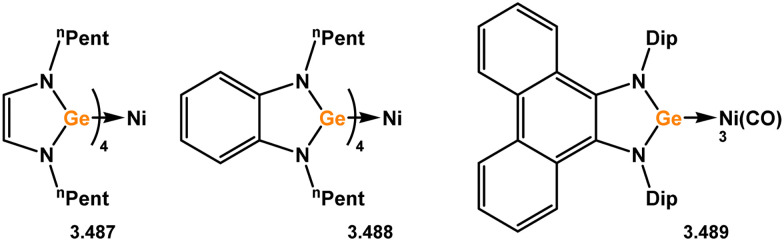
Additional examples of nickel(0) complexes featuring 5-membered NHGe ligands.

**Scheme 123:**
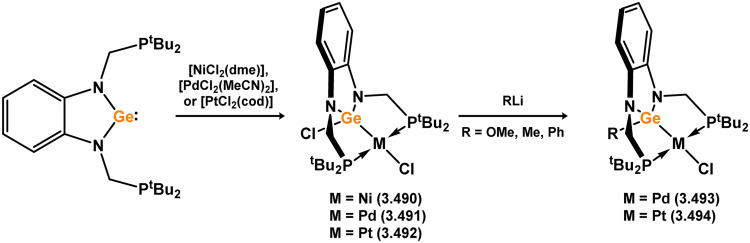
Reactivity of a phosphine-functionalised 5-membered NHGe ligand towards group 10 metal(ii) halide complexes.

**Scheme 124:**
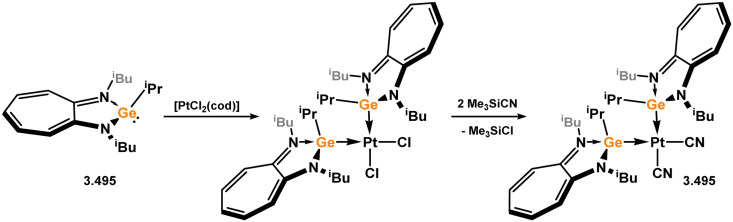
Formation of tropiminoato–germylene complexes of platinum(ii).

The first example of a group 10 NHGe complex accompanied the initial report of this ligand class, in 1992.^237^ There, it was shown that one, two, or three equiv. of 3.483 can complex Ni^0^, through modification of either stoichiometry or precursor, in generating 3.484, 3.485 and 3.486 (Scheme 121). Since that time, a number of group 10 NHGe complexes have been forthcoming. A handful of examples akin to those reported by Hermann and co-workers are now known, utilising *n*-pentyl functionalised germylenes 3.445 and 3.448, as well as the 9,10-phenanthrendiimine-derived system, in forming 3.487, 3.488, and 3.489 (Scheme 122).^239,245,250^ In all cases, these complexes were accessed through direct addition of the ligands to Ni^0^ precursors. It's worth noting here that the reduced steric hinderance of *n*-pentyl ligands 3.445 and 3.448, relative to the earlier reported *t*-butyl derivative, allows for the formation of tetra-NHGe complexes. As per the earlier described complexation of Rh–Cl and Ir–Cl fragments with phosphine appended NHGe ligand 3.460, reaction of this system with group 10 MCl_2_ precursors also leads exclusively to (chloro)(bisamido)germyl complexes (*viz.*3.490, 3.491, and 3.492, Scheme 123).^248,251^ It was also shown that these species undergo nucleophilic metathesis reactions selectively at the Ge–Cl fragment (*i.e.* in 3.493 and 3.494),^251^ which gives potentially important synthetic information for the future development of such systems. The tropiminoate NHGe 3.495 has also been employed in group 10 complexation chemistry, in forming [Pd(CN)_2_] complex 3.496, through initial formation of the [PtCl_2_] complex through a direct ligation, followed by Cl/CN exchange on reaction with Me_3_SiCN (Scheme 124).^252^ This complex was utilised in the catalytic cyanosilylation of carbonyl compounds.

**Scheme 125:**
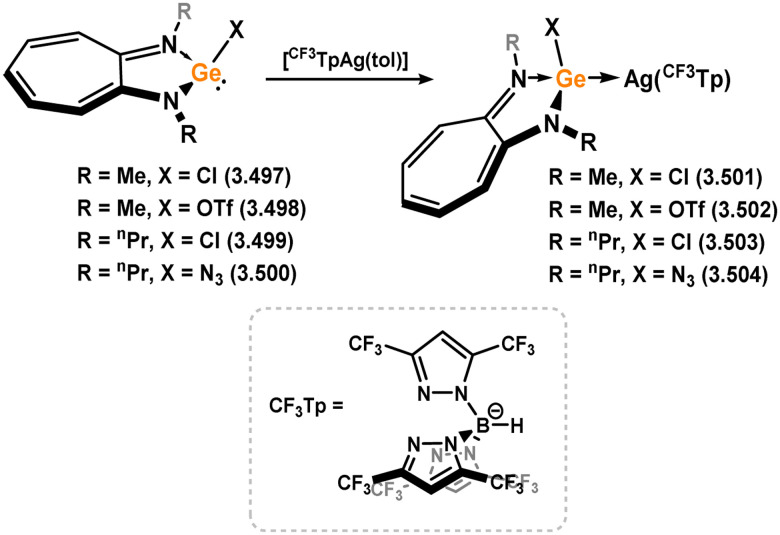
Formation of tropiminoato–germylene complexes of silver(i).

**Scheme 126:**
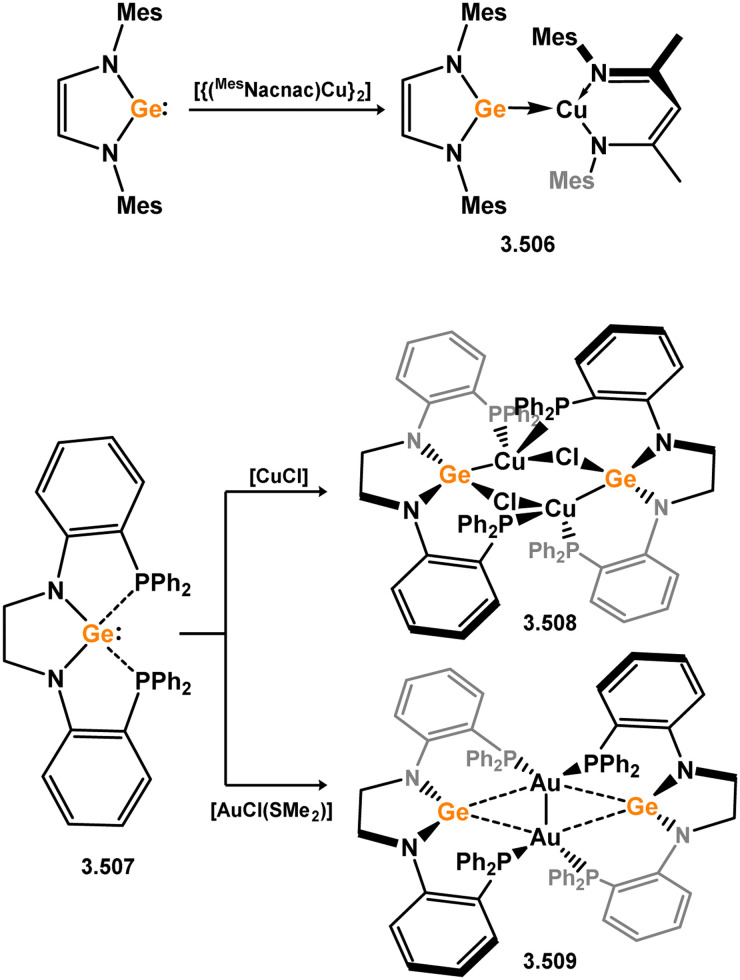
Reactivity of 5-memebered NHGe ligands towards coinage metal species.

The initial examples of coinage metal complexes bearing NHGe ligands were reported by the group of Dias, employing the tropiminoate ligands developed by that group. These involved Ge-halo, triflic, and azido ligands 3.497, 3.498, 3.499, and 3.500, and generally a tris(pyrazolyl)borate ligated silver(i) centre, forming compexes 3.501, 3.502, 3.503, and 3.504, respectively (Scheme 125).^253–255^ Nagendran and co-workers later extended this to copper(i) systems,^256^ employing chelating bis(NHGe) systems which will be discussed subsequently. One example of a ‘classic’ 5-membered NHGe complex of a copper(i) Nacnac system has been reported, again access through direct addition of the NHGe ligand to the dimeric Cu^I^ complex 3.505, forming 3.506 (Scheme 126). More recently, Goicoechea and co-workers reported a novel phosphine appended NHGe (*viz.*3.507, Scheme 126) which was shown to undergo interesting coordination chemistry with group 11 metal(i) chlorides, forming two examples of polynuclear complexes bearing two chelating germylene ligands (*viz.*3.508 and 3.509).^257^

**Scheme 127:**
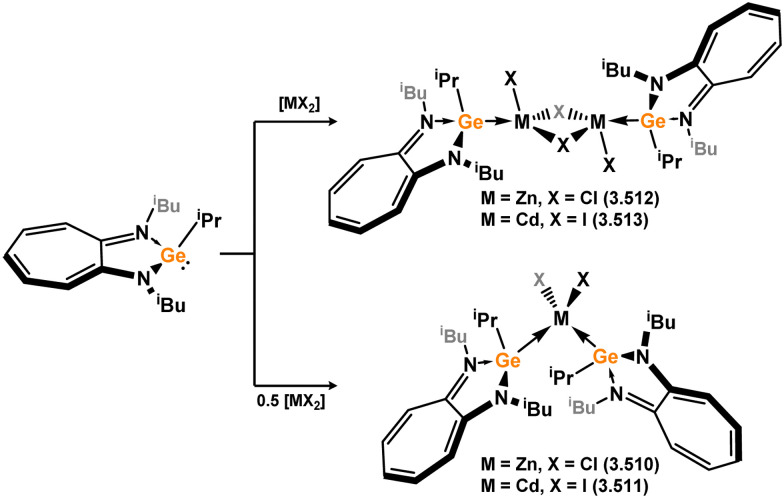
Formation of tropiminoato–germylene complexes of group 12 metals.

All reported 5-membered NHGe complexes of group 12 metals feature tropiminoate ligands, and are reported by the group of Nagendran (Scheme 127).^258,259^ Exclusively zinc and cadmium halide complexes are known, whereby addition of NHGe 3.495 to the metal halides leads to complexation; 2 : 1 reaction leads to doubly-NHGe coordinated species (*viz.*3.510 and 3.511), whilst 1 : 1 reaction leads to bimetallic halide bridged dimers (*viz.*3.512 and 3.513).

##### 6-Membered

Again, due to greater stability of Ge^II^ relative to Si^II^, there are considerably more 6-membered NHGes known when compared with the silicon counterparts. These are largely based upon the Nacnac ligand scaffold, although a good number of complexes are reported which feature phosphine appended cyclic bis(pyrollyl)germylenes.

**Scheme 128:**
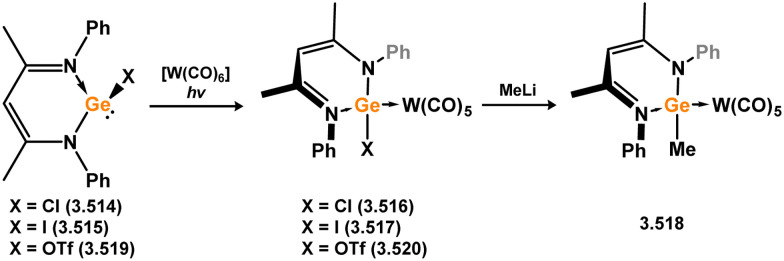
Reactivity of 6-membered Nacnac-germylene ligands towards tungsten(0).

No 6-membered NHGes are known for groups 3–5. The first example of a group 6 species employed the simple phenyl Nacnac-stabilised (halo)germylenes 3.514 and 3.515, which reacted with [W(CO)_6_] under irradiation in forming [W(CO)_5_] complexes 3.516 and 3.517 (Scheme 128).^260^ These were shown to undergo salt-metathesis with MeLi at the Ge–X moiety (X = Cl, I), generating (methyl)germylene complex 3.518. The same group later reported the closely related (triflic)germylene 3.519, from which the [W(CO)_5_] complex 3.520 can also be accessed.^261^

**Scheme 129:**
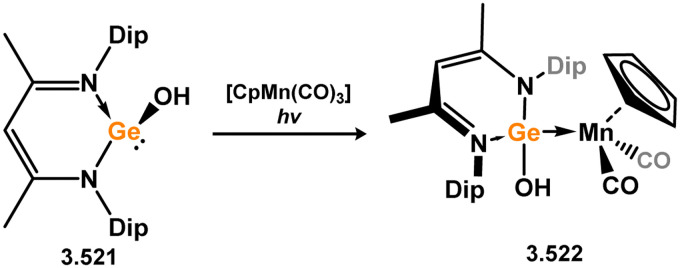
Formation of a manganese(i) complex bearing a 6-membered Nacnac-germylene ligand.

Regarding group 7, Dip-substituted Nacnac germylene 3.521 was shown to react with [Mn(Cp)(CO)_3_] under irradiation, to form complex 3.522 (Scheme 129).^262^

**Scheme 130:**
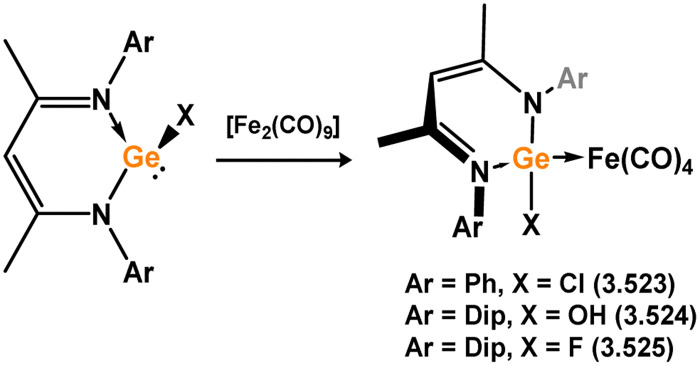
Formation of iron(0) complexes bearing 6-membered Nacnac-germylene ligands.

**Scheme 131:**
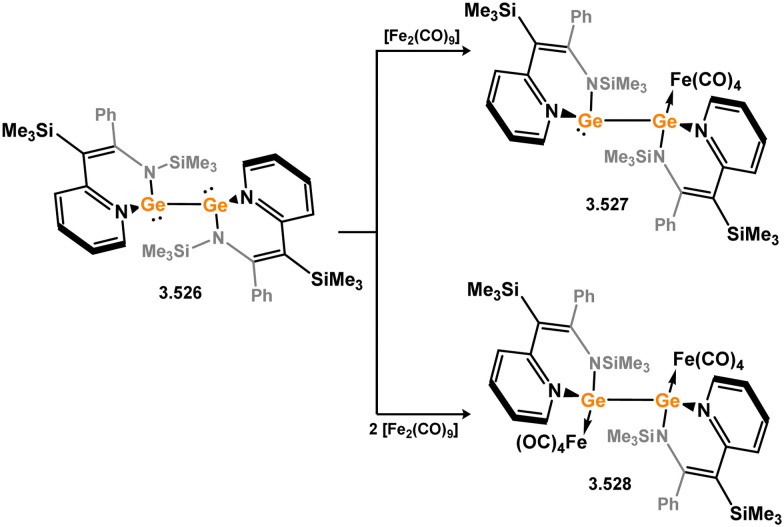
Reactivity of a Ge–Ge bonded 6-membered bis(germylene) ligand towards iron(0).

**Scheme 132:**
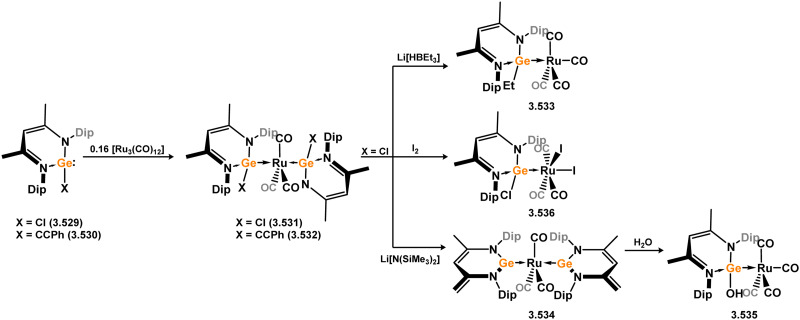
Formation of ruthenium(0) complexes bearing 6-membered Nacnac-germylene ligands, and subsequent reactivity.

The first example of a group 8 6-membered NHGe complex, 3.523, was reported alongside the aforementioned [W(CO)_5_] complex 3.516. This bears the [Fe(CO)_4_] moiety, and the Ph functionalised chloro germylene 3.514 (Scheme 130).^260^ Similarly, complex 3.524, also an adduct of [Fe(CO)_4_], was reported alongside manganese complex 3.522,^262^ followed later by 3.525,^263^ featuring hydroxy- and fluoro-germylenes respectively. All complexes described were accessed through the addition of the free germylene ligands to [Fe(CO)_9_], in elimination of [Fe(CO)_5_]. Further [Fe(CO)_4_] complexes utilising the bis(germylene) 3.526, which can be considered as a dimer of two 6-membered germylenes, have also been accessed through addition of this dimer to either one or two equiv. [Fe(CO)_9_], leading to mono (3.527) or bis (3.528) adducts, respectively (Scheme 131).^264^ A series of Ru complexes have also been reported by Zhu, Wen and co-workers.^265^ These primarily utilised the (chloro)germylene 3.529, in addition to the acetylide derivative 3.530, which each react with [Ru_3_(CO)_12_] in a 6 : 1 ratio to yield bis(germylene) [Ru(CO)_3_] complexes 3.531 and 3.532 (Scheme 132). Utilising complex 3.532, the chloride ligand undergoes salt-metathesis reactivity to form (ethyl)germylene complex 3.533, and reacts with the strong base Li[N(SiMe_3_)_2_] in generating the deprotonated Nacnac germylene complex 3.534, in which the germylene ligands are akin to the 6-membered Si^II^ system reported by Driess and co-workers, where the Nacnac system is now dianionic. This complex reacts with H_2_O *via* addition across the unsaturated ligand, re-forming the initial Nacnac ligand in (hydroxy)germylene complex 3.535. Finally, (chloro)germylene complex 3.532 reacts with I_2_ exclusively at Ru, forming di(iodo)ruthenium(ii) complex 3.536. This particular study thus begins to demonstrate the potential non-innocent behaviour of tetrylene ligands in the coordination sphere of a transition metal.

**Scheme 133:**
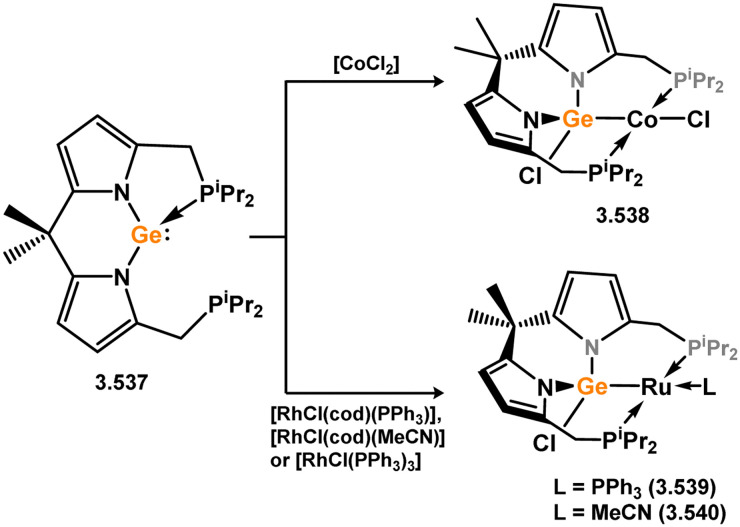
Reactivity of a phosphine-functionalised 6-membered germylene ligand towards cobalt(ii) and ruthenium(i) halide species.

**Scheme 134:**
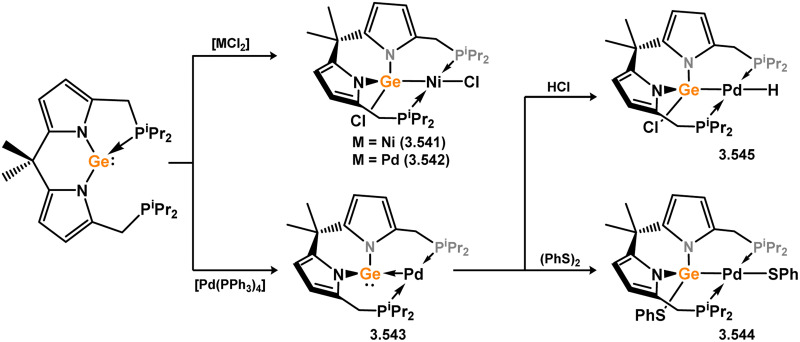
Reactivity of a phosphine-functionalised 6-membered germylene ligand towards group 10 metal species, and subsequent reactivity of a T-shaped palladium(0) complex.

**Scheme 135:**
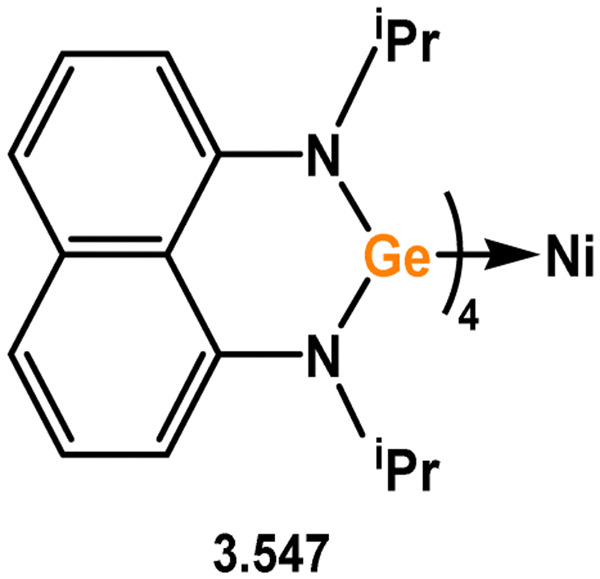
A nickel(0) complex bearing a 1,8-bisaminnaphthalene-derived 6-membered germylene ligand.

Group 9 complexes bearing 6-membered NHGes are as yet unknown, although examples of the addition of phosphine-appended germylene 3.537 to Co and Rh halides has been described.^266,267^ In these cases, Ge insertion in the M–Cl bond of the [CoCl_2_] or [RhCl(L)] (L = PPh_3_, MeCN) fragments is observed, forming 3.538, 3.539, and 3.540 (Scheme 133). Similar chemistry has also been reported by the same group for [NiCl_2_] and [PdCl_2_], leading to complexes 3.541 and 3.542 (Scheme 134).^266,268^ However, Pd^0^ germylene complex 3.543 could also be accessed. Notably, this complex demonstrated dual-centred bond activation chemistry, in the scission of both PhSSPh and HCl, leading to 1,2-addition products 3.544 and 3.545.^268^ Earlier, the 1,8-naphthelenediamine derived germylene 3.546 was shown to form a four-fold complex when reacted with [Ni(cod)_2_] (*viz.*3.547, Scheme 135).^269^

**Scheme 136:**
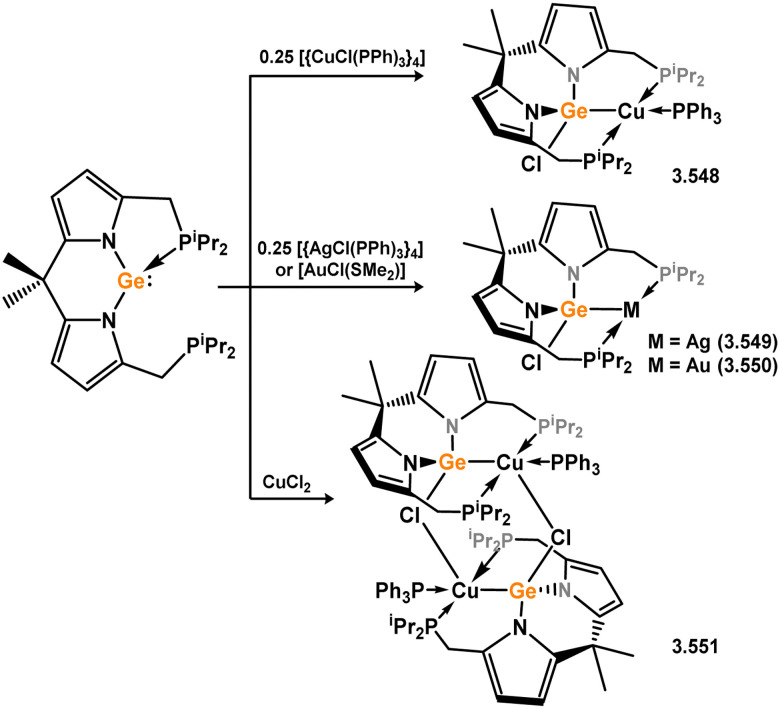
Reactivity of a phosphine-functionalised 6-membered germylene ligand towards coinage metal species.

As with the above described reactions of phosphine-appended germylene 3.537 with various TM chlorides, the same ligand reacts with MCl (M = Cu, Ag, Au) and [CuCl_2_] through insertion of the Ge centre into the M–Cl bonds. In the former species, monomeric complexes 3.548, 3.549, and 3.550 are formed,^270^ whilst reaction with [CuCl_2_] leads to chloride-bridged 3.551, which features a [Ge_2_Cu_2_Cl_2_] ring (Scheme 136).^266^

**Scheme 137:**
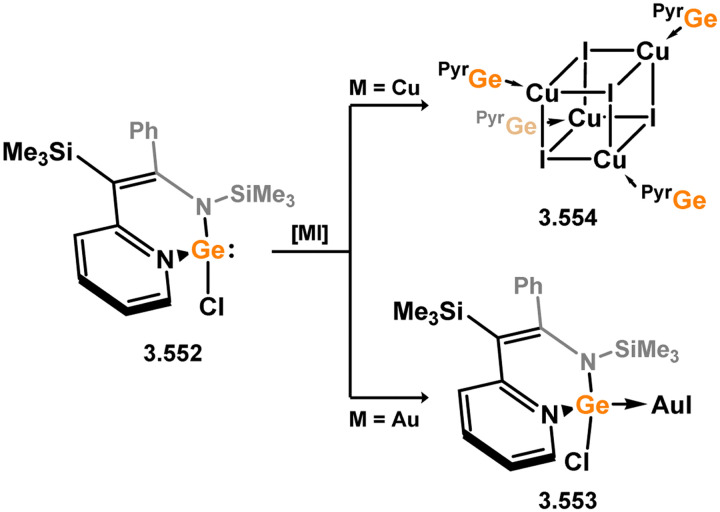
Reactivity of a pyridyl-1-azaallyl-derived 6-membered germylene ligand towards coinage metal species.

**Scheme 138:**
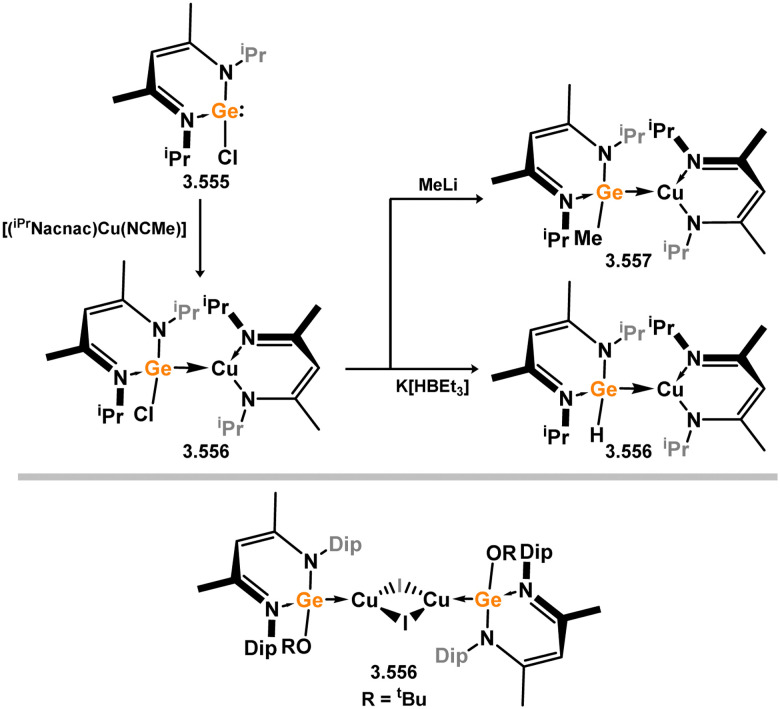
The formation and further reactivity of copper(i) complexes bearing Nacnac-germylene ligands.

Pyridyl-1-azaallyl stabilised (chloro)germylene 3.552 has also been employed in the coordination of coinage metal halides, leading to terminal [AuI] complex 3.553, and cubic [CuCl] complex 3.554 (Scheme 137).^271^ A number of Nacnac stabilised germylene-coinage metal complexes are also known (Scheme 138). The first example was a direct adduct of (chloro)germylene 3.555 with the corresponding Nacnac stabilised Cu^I^ complex.^272^ Here, the chloride substituent at germanium was shown to readily undergo salt-metathesis reactions in forming (hydrido)- and (methyl)germylene complexes 3.556 and 3.557. One alkoxide-funtionalised Nacnac-germylene was also shown to complex [Cu_2_I_2_], in 3.558.^273^

**Scheme 139:**
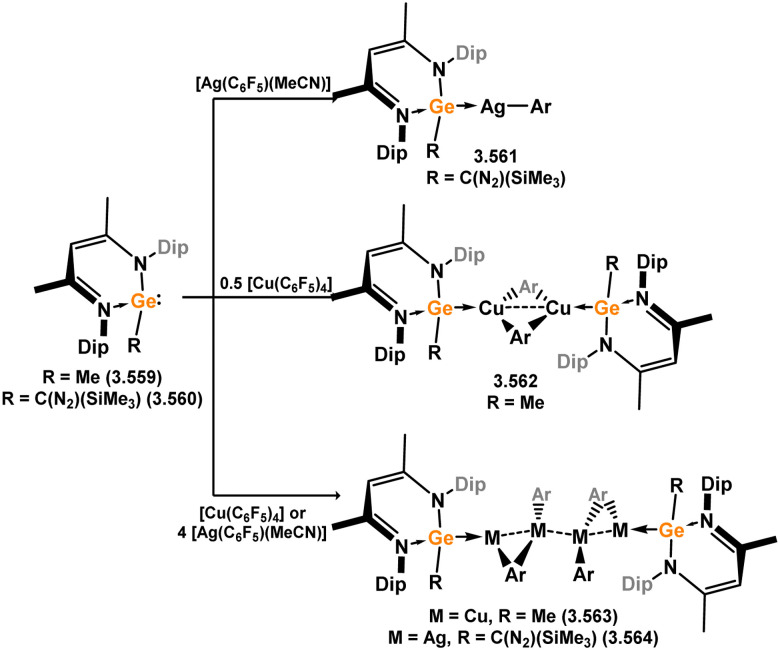
Coinage metal complexes bearing Nacnac-germylene ligands, whereby dimeric and tetrameric metal chains are formed.

**Scheme 140:**
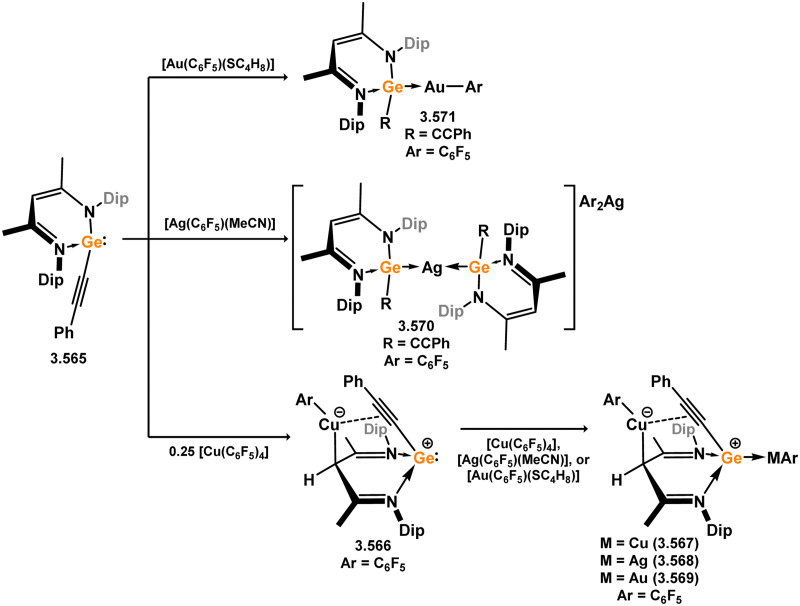
Coinage metal complexes of (acetylide)(Nacnac)germylene ligands, allowing for a unique binding motif within the Nacnac ligand backbone.

Methyl- and diazomethyl-functionalised germylenes (3.559 and 3.560, respectively) form adducts with [M(C_6_F_5_] fragments, leading to terminal (M = Ag; 3.561), dimeric (M = Cu; 3.562), and tetrametallic M chains (M = Cu (3.563), Ag (3.564)), dependant on the stoichiometry of the reaction (Scheme 139).^274^ As an interesting extension to that work, it was later shown that (alkynyl)germylene 3.565 reacts with [Cu(C_6_F_5_)] under ligand oxidation, through coordination of Cu to the alkyne and Nacnac moieties, rather than at Ge (3.566, Scheme 140).^275^ Addition of further [M(C_6_F_5_)] species (M = Cu (3.567), Ag (3.568), Au (3.569)) led to coordination of this second equivalent at Ge, so generating homo- and hetero-bimetallic coinage metal complexes. Reaction of the germylene ligand with [M(C_6_F_5_)] (M = Ag, Au) did not lead to the same ligand activation product as observed for Cu, but rather to terminal coordination, forming cationic bis(germylene)silver complex 3.570 and neutral mono(germylene)gold complex 3.571.

**Scheme 141:**
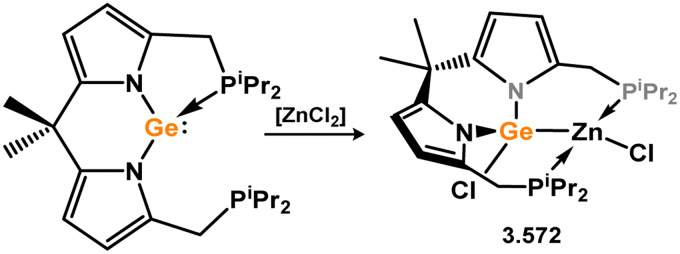
Reactivity of a phosphine-functionalise 6-membered germylene ligand towards zinc(ii) chloride.

No formal 6-membered germylene complexes of group 12 metals are known, though already described M–Cl insertion chemistry is known for the phosphine appended germylene 3.537 and ZnCl_2_, in the synthesis of complex 3.572 (Scheme 141).^266^

#### 3.2.2. Further cyclic germylene systems

**Scheme 142:**
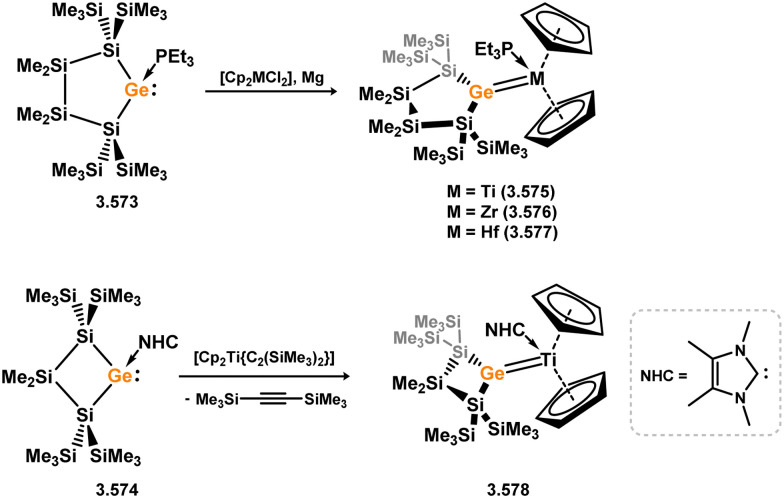
Synthesis of group 4 complexes stabilised by cyclic 4- and 5-membered bis(silyl)germylenes.

In addition to the described NHGe ligated TM complexes, a small number of additional cyclic germylene complexes are known, being bis(silyl) or bis(alkyl) derivatives. The first examples are analogues to their congeneric earlier reported Sn and Pb systems (*vide infra*), namely 4- and 5-membered cyclic bis(silyl) germylene complexes of Ti, Zr, and Hf (Scheme 142).^276^ These were synthesised either *via* the *in situ* reduction of [Cp_2_MCl_2_] with Mg, in the presence phosphine-stabilised germylene 3.573 (for 5-membered derivatives), or through the addition of NHC-stabilised 4-membered germylene 3.574 to the Ti-acetylene adduct [Cp_2_Ti{C_2_(SiMe_3_)_2_}], in forming complexes 5-membered NHGe complexes 3.575, 3.576, and 3.577, and 4-membered NHGe complex 3.578.

**Scheme 143:**
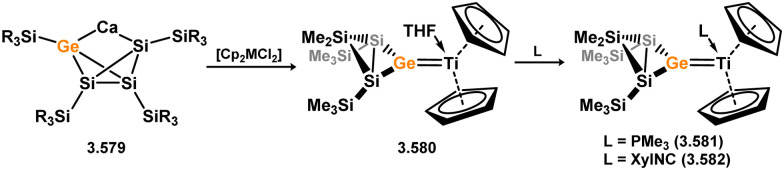
Synthesis of bicyclic bis(silyl)germylene complexes of titanium, which are described as Schrock germylene complexes.

Later, it was shown by Lee, Sekiguchi and co-workers that the bicyclic dianion 3.579 reacts with [Cp_2_TiCl_2_] in forming the bicyclic-germylene adduct of [Cp_2_Ti], 3.580, as its THF adduct. This species could be crystallised as their PMe_3_ (3.581) and XylNC (3.582) adducts, revealing a short Ge–Ti bond marked as a formal double bond (Scheme 143).^277^ Moreover, this linkage is negatively polarised towards Ge, in contrast to the vast majority of tetrylene complexes discussed in this review, and as such is best described as a Schrock germylene complex. The same publication reported the W and Mo derivatives, though no structural data was provided.

**Scheme 144:**
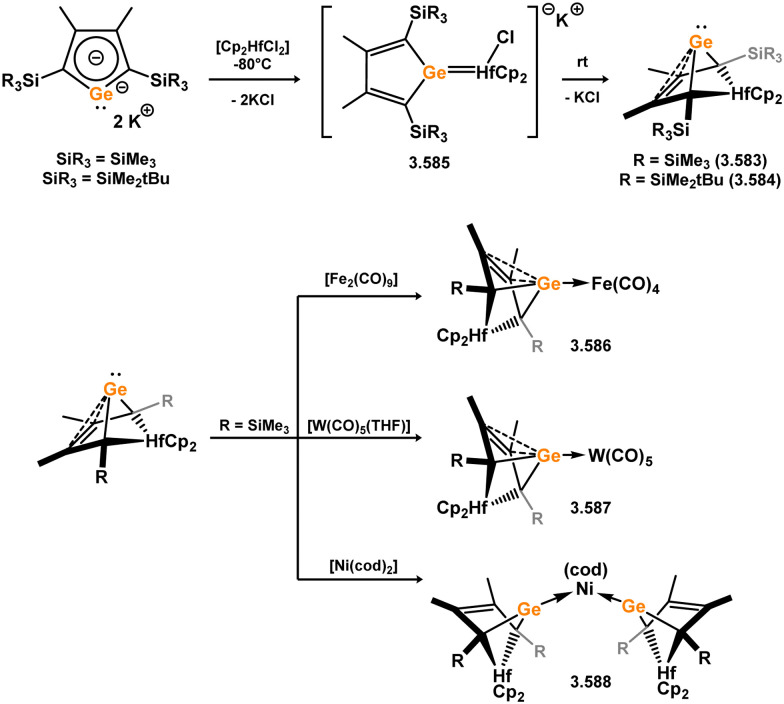
Transient formation of an unsaturated 5-membered germylene-haffnium complex, rearrangement to a bicyclo[2.1.1.]hexene germylene complex, and its utility in the formation of heterometallic species.

Later, the hafnium-based bicyclo[2.1.1.]hexene germylene (BCHGe) systems 3.583 and 3.584 were reported, accessed *via* the salt-metathesis of the corresponding dipotassium germole ligands with [Cp_2_HfCl_2_], which proceeds through the anionic germylene complex 3.585 (Scheme 144).^278,279^ These BCHGe ligands are stabilised through a significant interaction with the unsaturated C–C backbone.^279^ Nevertheless, they were shown to react with TM fragments as typical Lewis basic ligands, as shown through the addition of 3.583 to [Fe_2_(CO)_9_] and [W(CO)_5_(THF)]. In these species (*viz.*3.586 and 3.587) the interaction of the Ge centre with the C=C π-system is maintained, indicating negligible M → Ge (M = Fe, W) back-bonding. In contrast, reaction with [Ni(cod)_2_] led to an equilibrium mixture of 3.583 and bis(germylene) complex 3.588, in which the Ge⋯C=C interaction is cleaved. This is indicative of a greater Ni → Ge back-donation when compared to the above complexes, which competes with the described π-interaction. As such, isolated complex 3.586 is not stable in solution, rapidly decomposing upon dissolution.

**Scheme 145:**
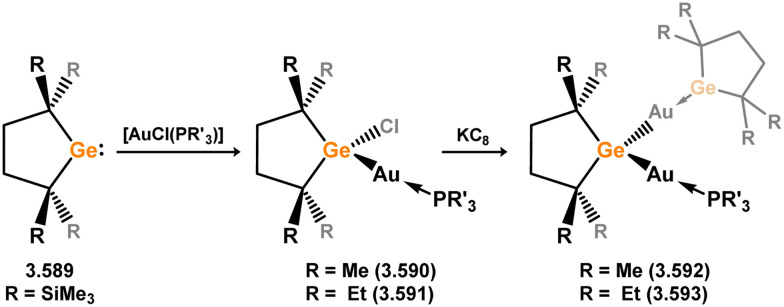
Formation of mono- and bis-gold complexes utilising a cylic bis(alkyl)germylene ligand.

Finally, Kira and co-workers reported the synthesis of a cyclicalkylgermylene-stabilised di-goldgermane complexes, generated through the initial insertion of bis(alkyl)germylene 3.589 into the Au–Cl bond of [AuCl(PR_3_)] (R = Me (3.590), R = Et (3.591)), followed by KC_8_ reduction forming 3.592 and 3.593 (Scheme 145).^280^ The core of these species can be described as a dimetallated germane, with halogen-like Au centres which bear a degree of negative charge localisation, each supported by a dative ligand (*i.e.* germylene or phosphine).

#### 3.2.3. Acyclic germylene TM complexes

In contrast to cyclic-germylene complexes, which typically employ stable germylenes in addition reactions to a metal species, the synthesis of acyclic–germylene transition metal complexes has been approached *via* numerous different routes (*e.g.* addition to TM–Ge triple bonds, activation of germanes, elimination from germyl complexes, *etc.*). It is also generally true that acyclic derivatives have historically been accessed with a focus on the fundamental electronic nature of the M–Ge bond, *e.g.* exploring Schrock *vs.* Fischer type character, and an amplified reactive capacity of the Ge centre, leading towards metal–ligand cooperativity.^27,281^ Acyclic–germylene complexes are more rare than cyclic systems, largely owing to challenges in their isolation. Still, the first example of an acyclic system was reported by Jutzi and co-workers in 1976, demonstrating the long standing interest in these fascinating complexes.^282^

**Scheme 146:**
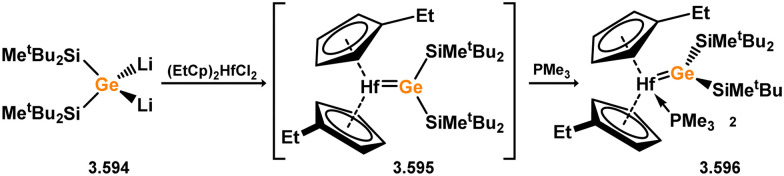
Synthesis of an acyclic bis(silyl)germylene haffnium complex.

No such complexes are known for group 3. Regarding group 4, addition of the dilithiogermanide 3.594 to a hafnocene dichloride derivative led to Hf complex 3.595 (Scheme 146).^283^ Given the instability of this initially formed product above −50 °C, the germylene complex is additionally stabilised by PMe_3_, which binds at Hf, forming 3.596. Complex 3.595 bears a formal Hf–Ge double bond, reflected clearly by the calculated frontier orbitals for this species which represent π- and π*-bonds for the HOMO and LUMO, respectively. 3.595 was assigned as a Schrock-type germylene complex, on the basis of the highly polarised Ge–Hf bond, with partial charges on Ge and Hf of −0.32 and +0.74, respectively.

**Scheme 147:**
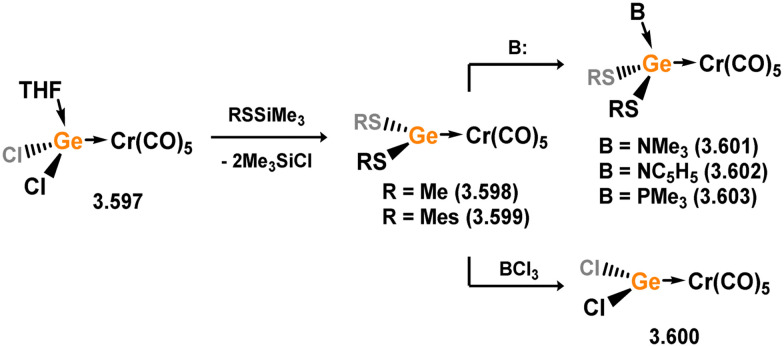
Early examples of chromium complexes bearing acyclic germylene ligands.

**Scheme 148:**
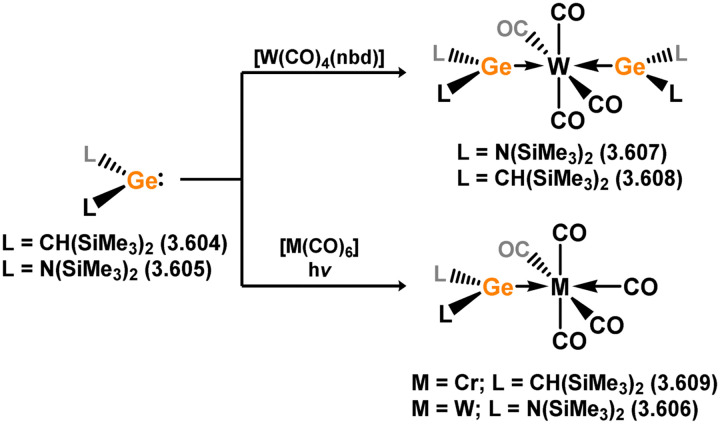
Formation of chromium(0) and tungsten(0) complexes bearing acyclic germylene ligands.

Numerous examples of acyclic–germylene complexes of the group 6 metals are known, the first reported by Jutzi and co-workers in 1976, with the first structurally characterised example appearing the following year, reported by Lappert and co-workers.^51,282^ We note that the X-ray crystal structure of the Jutzi example was reported two years later.^284^ The former report first involved the synthesis of the base-stabilised [GeCl_2_] adduct of [Cr(CO)_5_] (*viz.*3.597, Scheme 147),^285^ which reacts with thiosilanes RSSiMe_3_ (R = Me, Mes) to yield di(thio)germylene complexes 3.598 and 3.599.^282^ These systems also further react with BCl_3_ in thiol-chloride exchange, forming base-free dichlorogermylene complex 3.600. Later, alongside the crystallographically determined structure of 3.599, the same group reported that addition of bases to the germylene complexes led to binding at Ge in forming complexes 3.601, 3.602, and 3.603.^284^ This has implications in cooperative bond activation *via* ligand centred nucleophile binding. Soon after, Lappert and co-workers demonstrated the coordination ability of their earlier reported acylic bis(alkyl)- and bis(amido)germylenes (3.604 and 3.605, respectively; Scheme 148).^51^ Bis(amido) system 3.605 was shown to undergo mono-carbonyl substitution with [W(CO)_6_] under irradiation, yielding 3.606, whilst reaction with [W(CO)_4_(norbornadiene)] formed bis-germylene complex 3.607. For bis(alkyl) germylene 3.604, the corresponding bis-germylene was also reported (*viz.*3.608), in addition to the [Co(CO)_5_] complex 3.609, generated by addition of 3.604 to [Co(CO)_6_] under irradiation.

**Scheme 149:**
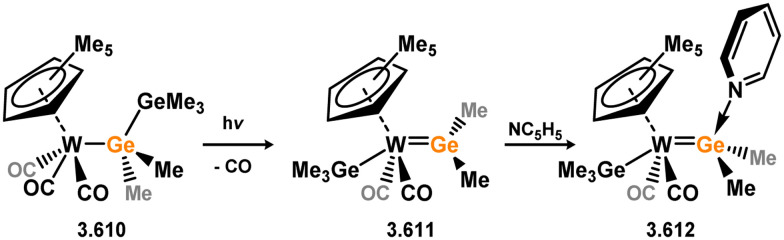
Formation and coordinative behaviour of dimethylgermylene in the coordination sphere of tungsten.

**Scheme 150:**
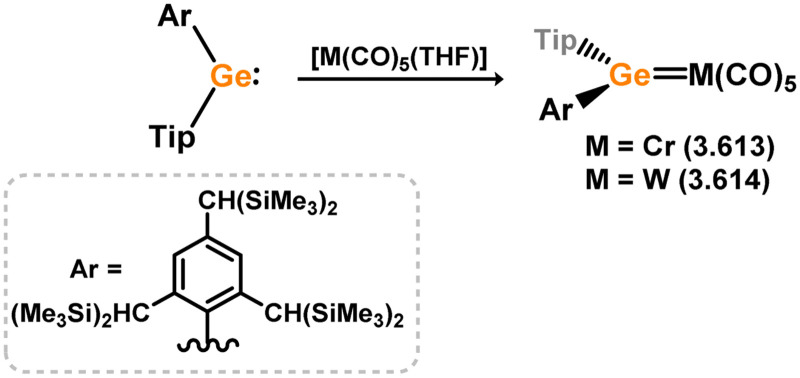
Formation of bis(aryl)germylene complexes of tungsten and chromium. Tip = 2,4,6-^i^Pr_3_-C_6_H_2_.

**Scheme 151:**
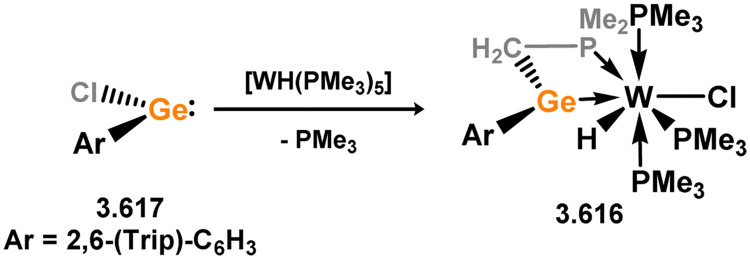
Formation of a phosphine-appended germylene complex of tungsten.

**Scheme 152:**
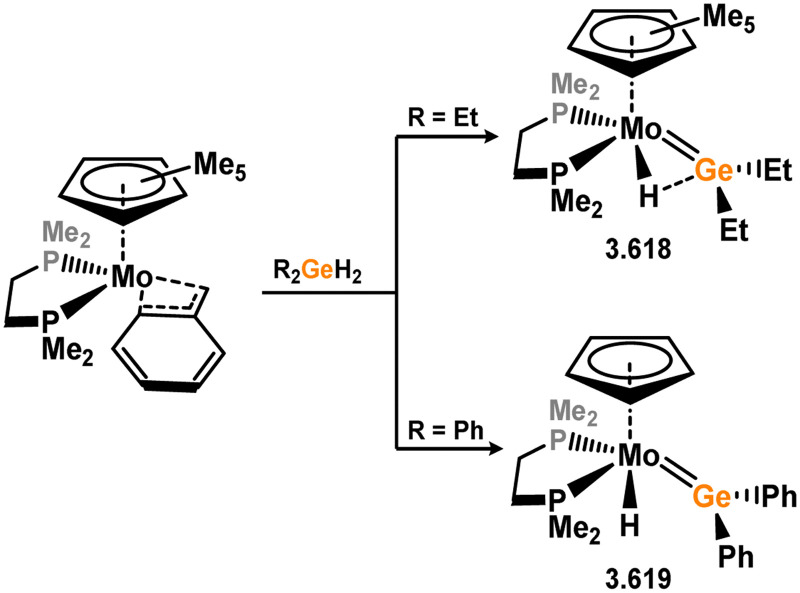
Formation of bis(organyl)germylene complexes of molybdenum through dehydrogenation of a germanes.

Much later, further examples of group 6 complexes were reported. In 1993, it was demonstrated that terminal digermyl-tungsten complex 3.610 undergoes germyl migration upon irradiation, forming (germyl)(germylene)tungsten complex 3.611, with a terminal dimethylgermylene ligand (Scheme 149).^286^ This approach to such a species is of significant importance, given that [Me_2_Ge:], the smallest alkyl germylene, is not stable in its own right. Notably, Ogino and co-workers reported spectroscopic evidence for such a process some years earlier, in the irradiation of [CpFe(CO)_2_(Ge_2_Me_3_)] and derivatives.^287^ The electrophilicity of the germanium centre in 3.611, as per examples from Jutzi, was also demonstrated through the addition of pyridine, which selectively coordinates at Ge (*viz.*3.612). Soon after, the first examples of base-free bis(aryl)germylene TM complexes were reported (*viz.*3.613 and 3.614), through addition of the isolable bis(aryl)germylene 3.615 to [M(CO)_5_·THF] (M = W, Cr; Scheme 150).^288^*En route* to a germylidyne complex (*vide infra*), Filippou and co-workers reported the synthesis of phosphine-appended complex 3.616, through addition of (aryl)(chloro)germylene 3.617 to hexakis-trimethylphosphino tungsten (Scheme 151). Here, the Ge–Cl bond is activated, in addition to the Ge centre inserting into the W–C bond of an activated PMe_3_ ligand.^289^ Acyclic bis(alkyl)germylene and bis(aryl)germylene complexes of molybdenum were reported by Tilley, accessed through the activation of germanes by their previously reported benzyl molybdenum(ii) complex [Cp*Mo(pmpe)(CH_2_Ph)] (dmpe = 1,2-(PMe_2_)C_2_H_4_; Scheme 152).^290^ An initial Ge–H bond activation leads to the elimination of toluene, with subsequent hydride migration from Ge to Mo generating target bis(alkyl)- and bis(aryl)-germylene complexes 3.618 and 3.619, respectively. Although one might assume that bis(aryl) system 3.619 bears a more electrophilic Ge centre, only the bis(alkyl) system features a bridging Mo–H⋯Ge interaction. This chemistry is particularly reminiscent of silane/silylene chemistry from the same group, which has implications in hydrosilylation catalysis which may proceed *via* TM silylene complexes.

**Scheme 153:**
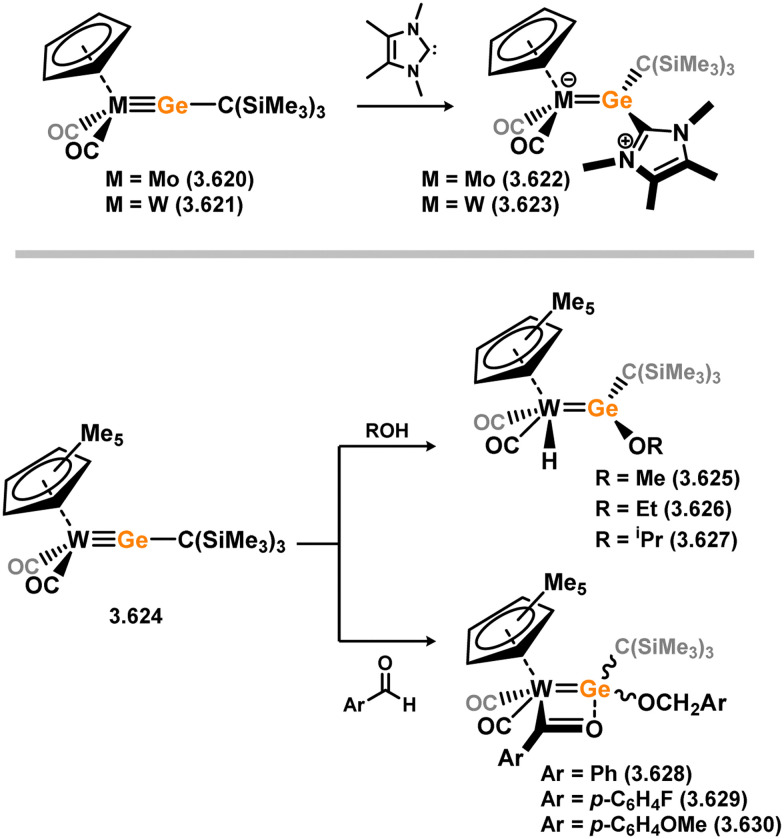
Formation of acyclic germylene complexes of molybdenum and tungsten through addition reactions to germylidyne complexes.

Three further examples of acyclic–germylene complexes of group 6 metals are known, accessed *via* metal germylidyne complexes which are discussed subsequently (Scheme 153). First, Filippou and co-workers reported that Mo and W germylidyne complexes 3.620 and 3.621 react with the small NHC [(MeCNMe)_2_C:], with binding exclusively at Ge.^291^ This leads to a formal imidazolium salt, with a negative charge residing at Mo/W, and a formal Ge–C bond. As such, products 3.622 (Mo) and 3.623 (W) are best described as germylene complexes, borne out by the considerable lengthening of their Ge–M bonds (*i.e.* Δ*d*_M–Ge_ for 3.622 : 0.1199 Å; for 3.623 : 0.1173 Å). Finally, the closely related Cp*-tungsten germylidyne complex 3.624, reported by Hashimoto and Tobita and co-workers, reacts with both alcohols and aldehydes in the formation of germylene complexes.^292^ In the former reactions, 1,2-addition across the W–Ge triple bond is observed, with the alkoxide moiety bound at Ge indicative of the electrophilicity of this centre, forming novel germylene species 3.625, 3.626, and 3.627. The situation is somewhat more complex for aldehydes; two equivalents react, the first in C–H activation across the W–Ge bond in forming an (alkyl)(hydrido)germylene complex, and the second through insertion into the newly formed Ge–H moiety. This was observed for a variety of aryl aldehydes, yielding compounds 3.628, 3.629, and 3.630, in all cases forming chiral Ge centres, though none demonstrating notable enantioselectivity.

**Scheme 154:**
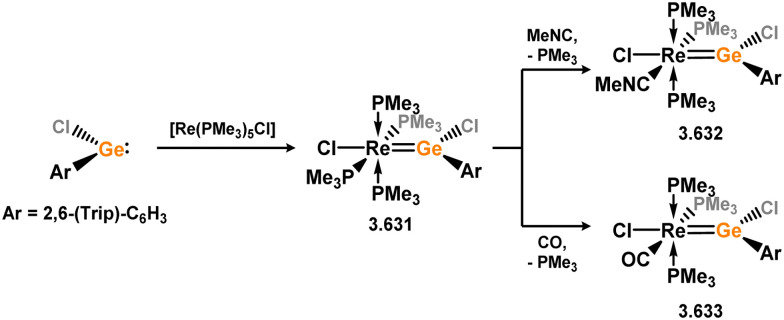
Formation of rhenium complexes bearing acyclic germylene ligands.

Three true examples of group 7 TM complexes bearing acyclic germylenes are known, namely Re complexes 3.631, 3.632, and 3.633, the initial complex accessed through the direct addition of (aryl)(chloro)germylene 3.617 to [ReCl(PMe_3_)_5_] (Scheme 154).^293^ Subsequently this species loses an additional PMe_3_ ligand in forming a germylidyne complex (*vide infra*), which reacts with CO or MeNC in the regeneration of germylene complexes, 3.632 and 3.633.

**Scheme 155:**
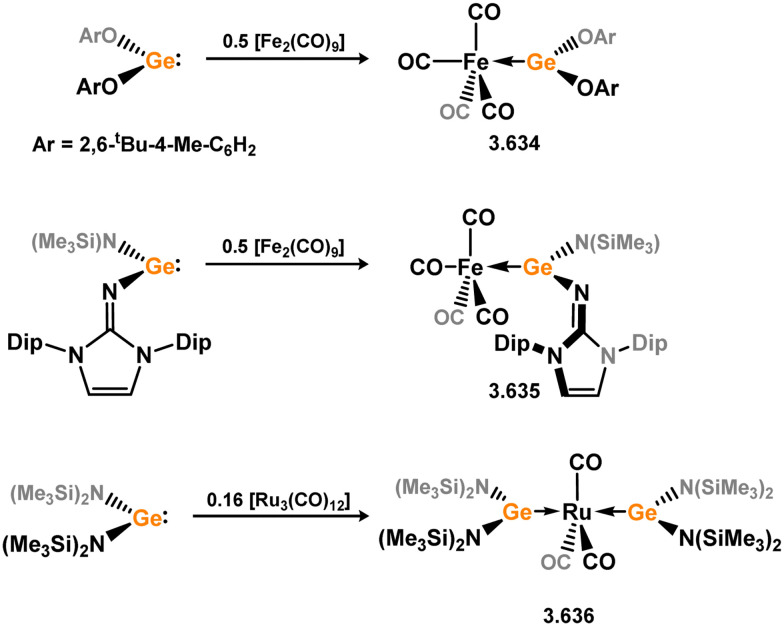
Generation of iron(0) and ruthenium(0) complexes bearing acyclic germylene ligands.

A number of Fe and Ru complexes with acyclic germylene ligands have been reported (Scheme 155), accessed either *via* direct germylene addition to Fe species, or through Ge–H activation of germanes. For the former, the [Fe(CO)_4_] complexes 3.634 and 3.635 are known, the initial example reported by Lappert and co-workers as early as 1986, as well as a bis(germylene) [Ru(CO)_3_] complex reported by Cabeza, García-Álvarez and co-workers (*viz.*3.636).^242,294,295^

**Scheme 156:**
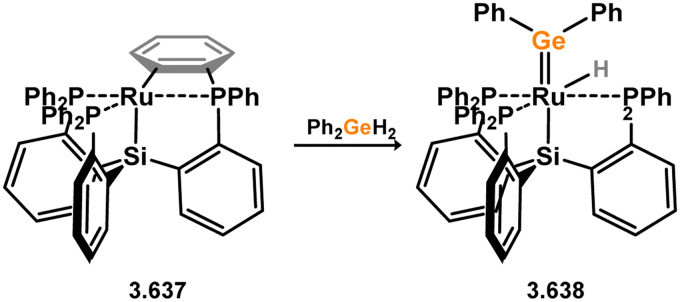
Formation of a diphenylgermylene complex of ruthenium *via* germane dehydrogenation.

**Scheme 157:**
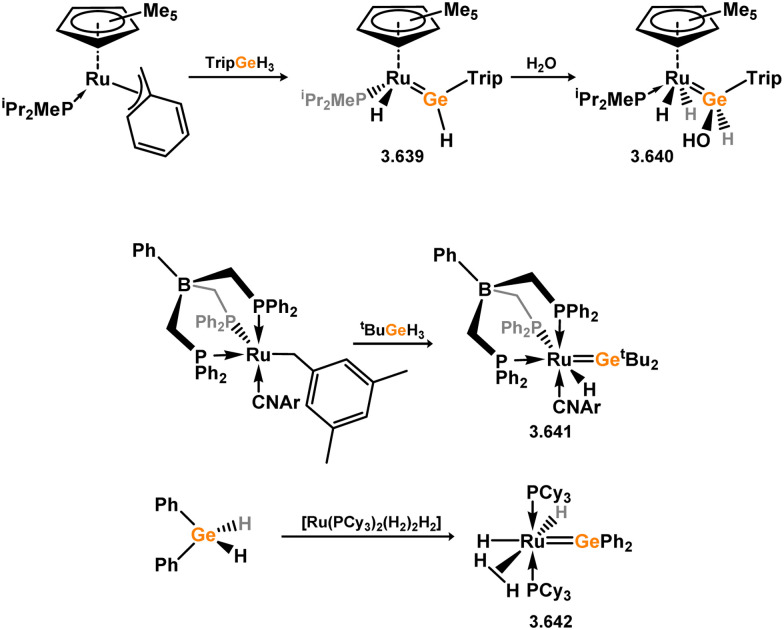
Further examples of ruthenium germylene complex formation through H_2_ extrusion.

The first observation of terminal germylene formation through germane activation was reported by Peters and co-workers, in the addition of Ph_2_GeH_2_ to Ru complex 3.637, in which one Ph group of the ligand is activated. Addition of the germane reprotonates this ligand moiety, with the second Ge–H fragment transferred to the Ru centre in forming germylene complex 3.638 (Scheme 156).^172^ In a similar methodology, Tilley and co-workers soon after reported that the addition of (Trip)GeH_3_ to [CpRu(PMe^i^Pr_2_)(CH_2_Ph)], which led to terminal (aryl)(hydrido)germylene complex 3.639, again through protonation, in this case of the benzyl fragment, and hydride migration to Ru (Scheme 157).^170^ This is also akin to earlier described silylene chemistry reported in the same submission, which has implications in alkene hydrosilylation catalysis.^59^ As with earlier examples, the addition of H_2_O to germylene complex 3.639 leads to addition across with Ru–Ge bond, with hydroxyl group at Ge, forming 3.640. This further indicates the Lewis acidity of such germylene ligands, and feeds into potential applications in cooperativity. Later, Tilley reported a further example of a hydrido germylene complex, 3.641, which forms on addition of alkyl germane ^*t*^BuGeH_3_ to [{PhB(CH_2_PPh_2_)_3_}Ru(CNAr)(Mes)].^296^ A final example of a Ru system has been reported, generated *via* addition of Ph_2_GeH_2_ to Ru–hydrogen complex [Ru(PCy_3_)_2_(H_2_)_2_(H)_2_], leading to bis(phenyl)germylene complex 3.642, demonstrating that bulky aryl ligands aren’t a requirement for the synthesis of such systems.^297^

**Scheme 158:**
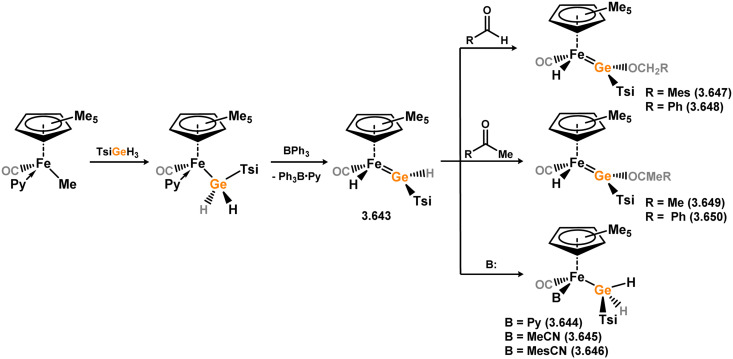
Formation of an iron complexes bearing an (aryl)(hydrido)germylene, and reactivity towards insertion and base coordination.

**Scheme 159:**
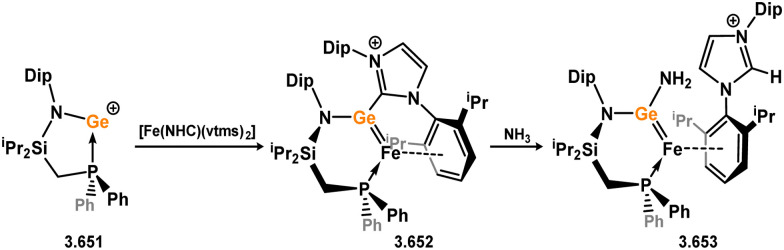
Synthesis of a cationic germylene iron(0) complex, and its cooperative activation of ammonia.

Moving to iron, Tobita and co-workers extended the described methodologies to this lighter metal in accessing hydrido germylene complex 3.643, through deprotonation of the bulky alkyl germane [(Me_3_Si)_3_C]GeH_3_ with methyl iron(ii) complex [Cp*Fe(CO)(Py)Me] (Py = pyridine; Scheme 158).^298^ Addition of ligands at Fe led to migration of the Fe–H fragment back to Ge, forming germyl complex 3.644, 3.645, and 3.646, whilst addition of aldehydes and ketones led to insertion into the Ge–H fragment (*viz*3.647, 3.648, 3.649, and 3.650). Most recently, our group has demonstrated ready access to carbonyl- and Cp-free iron(0) complexes (Scheme 159). The addition of cationic germylene 3.651 to [IPr·Fe·{η^2^-(vtms)}_2_] leads to alkene elimination and NHC migration to Ge, in the formation of 3.652. This is best described as a 2-coordinate acyclic germylene complex, given that the cationic charge likely resides on the NHC ligand (*i.e.* as the imidazolium), which features a formal Ge–Fe double bond.^299^ Despite this, addition of NH_3_ to 3.652 leads exclusively to binding at Ge, and proton transfer the carbene–carbon, generating bis(amido)germylene complex 3.653.

**Scheme 160:**
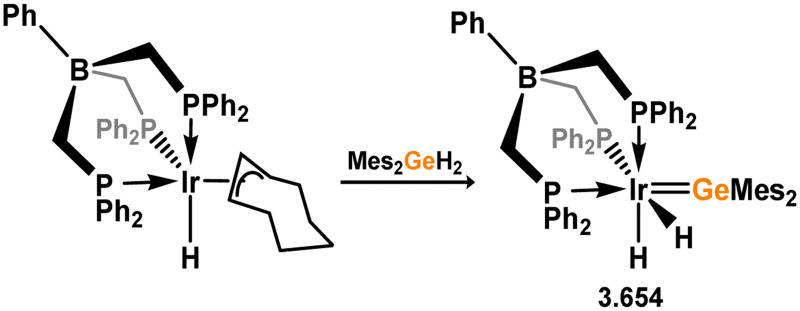
Formation of a bis(aryl)germylene iridium complex *via* H_2_ extrusion.

**Scheme 161:**
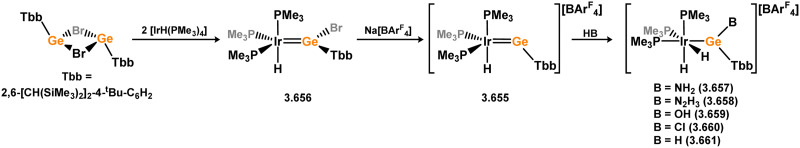
Formation of neutral and cationic iridium(i) complexes bearing gerlymene ligands, and the cooperative activation of numerous protic species across the Ir–Ge bond.

As per above described Ru chemistry, Tilley and co-workers have shown that addition of diaryl germane to allyl iridum complex [{PhB(CH_2_PPh_2_)_3_}IrH(C_8_H_13_)] leads to the formation of germylene species 3.654 (Scheme 160).^176^ Whilst that reaction is high yielding, it is interesting to note that Lappert's attempt to generate an iridium germylene complex some years earlier led to C-H activation of the ligand, when utilising the [(Me_3_Si)_2_N]^−^ scaffold.^300^ Closely related Ir germylene complexes were recently described by Wesemann and co-workers, *via* the cooperative activation of a range or protic small-molecules (NH_3_, N_2_H_4_, H_2_O, H_2_, HCl) in *pseuso*-germylyne complex 3.655, formed through bromide abstraction from (aryl)(bromo)germylene complex 3.656 (Scheme 161). We describe complex 3.655 as such due to the 2-coordiante Ge-centre, its bent geometry, and positive charge localisation at Ge (*i.e.* this is not a ‘true’ germylidyne, nor a metallogermylene). In all cases, the nucleophile binds at Ge, leading the proton transfer to the Ir centre, in the formation of 3.657, 3.658, 3.659, 3.660, and 3.661.^301^

**Scheme 162:**
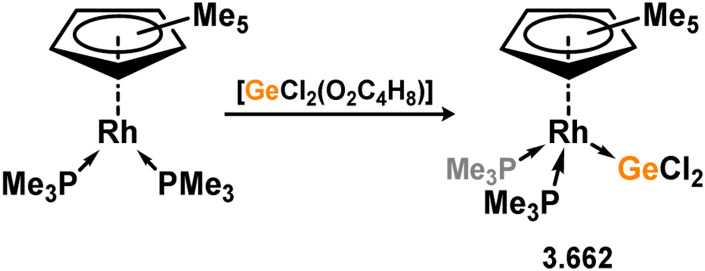
Germanium(ii) chloride behaving as a Z-type ligand in the coordination sphere of rhodium.

**Scheme 163:**
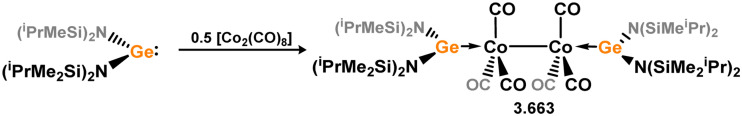
Formation of a dimeric cobalt(0) complex bearing two acyclic bis(amido)germylene ligands.

**Scheme 164:**
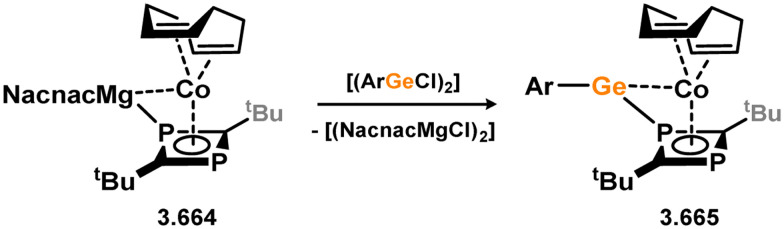
Formation of a cobalt complex bearing a diphosphacyclobutadiene-appended acyclic gerlymene ligand.

A single example of a rhodium complex bearing an acyclic germylene ligand is known, that is [GeCl_2_] complex 3.662, simply accessed through addition of [dioxane·GeCl_2_] to [Cp*Rh(PMe_3_)_2_], where interestingly the [GeCl_2_] moiety behaves as a Z-type ligand, receiving electron density from Rh (Scheme 162).^302^ Two examples of related cobalt complexes are known. The first involves the direct addition of bis(amido)germylene 3.605 to [Co_2_(CO)_8_], leading to end-on coordination of the germylene ligands at each Co centre (3.663, Scheme 163).^303^ The second such example sits on the boundary between a formal tetrylene Co complex, and a metallotetrylene, but it is nevertheless described here. Addition of (aryl)(chloro)germylene 3.617 to the magnesium cobaltate 3.664 leads to metathesis in the formation of 3.665, in which the germanium centre forms a close contact with on P-atom diphosphacyclobutadiene ligand, and a long Ge–Co contact is observed (Scheme 164).^304^ It is likely, then, that the Ge^II^ centre in this species is formally ligated by the aryl and diphosphacyclobutadiene ligands, and forms a dative interaction with the Co centre.

**Scheme 165:**
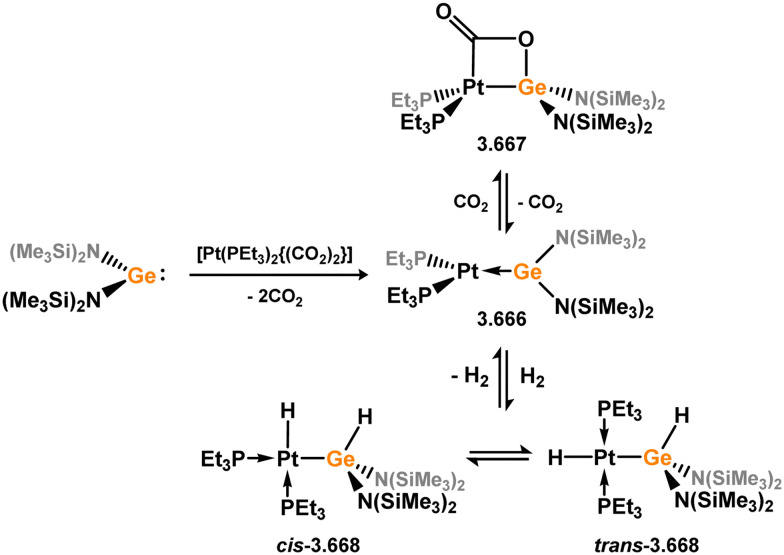
Synthesis and reactivity of an acyclic bis(amido)germylene–platinum(0) complex.

**Scheme 166:**
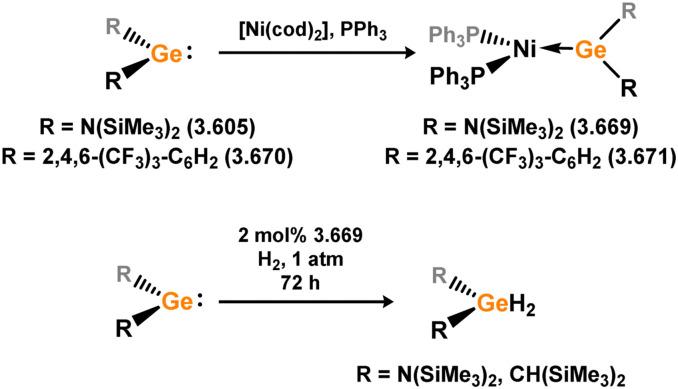
Synthesis and reactivity of various acyclic germylene–nickel(0) complexes.

Given the ready accessibility of M^0^ species of the group 10 metals, a considerable number of acyclic germylene complexes are known for these elements. This is made more interesting but the combined capacities of germylenes to behave as Lewis acids (*i.e.* Z-type ligands), and the group 10 metals to behave as Lewis bases (*i.e.* L-type ligands). Initial reports regarding this compound class came from the group of Holl, with a series of three publications giving key insights into both the bonding and reactivity of such species (Scheme 165). In the first submission, the Pt^0^ germylene complex 3.666 was described, bearing the bis(amido)germylene developed by Lappert, 3.605.^47^ This remarkable species, accessed through CO_2_ elimination from a Pt^II^ oxalate, demonstrated both the reversible [2+2] cycloaddition of CO_2_ and reversible H_2_ addition across the Ge–Pt bond (*viz.*3.667 and 3.668). This was an early demonstration of the cooperative bond activation capacities of such heteroelemental bonds, and remains a central point of investigation in the implementation of low-valent p-block ligands to this day. The described [2+2] cycloaddition chemistry was later extended to arylnitroso species.^305^ Subsequently, the same group showed that the closely related Ni^0^ complex (*viz.*3.669) can be readily accessed, and behaves as an efficient catalyst for the hydrogenation of the germylenes 3.604 and 3.605.^306^ It's important to note here that addition of H_2_ to isolated Ni complex 3.669 did not lead to an isolable H_2_ activation product, highlighting both the differences in reactivity between Ni and the heavier group 10 metals, as well as the importance of ligand design in developing such chemistry for the more abundant base metals. In their third submission on the topic, Holl and co-workers extended their Ni^0^ system to the (fluroaryl)germylene 3.670, in the synthesis of complex 3.671 (Scheme 166).^307^ This report did not describe further reactivity studies, but rather focused on the electronic structure and bonding in this species when compared with more electron rich germylene systems. The key finding was a significant degree of π-character in the Ge–Ni bond, with the strongly electron withdrawing nature of the [2,4,6-(CF_3_)_3_C_6_H_2_] increasing Ni → Ge back-bonding relative to the bis(amido) derivative 3.669. This bonding nature is additionally borne out by the planarity of the Ge centre in these systems.

**Scheme 167:**
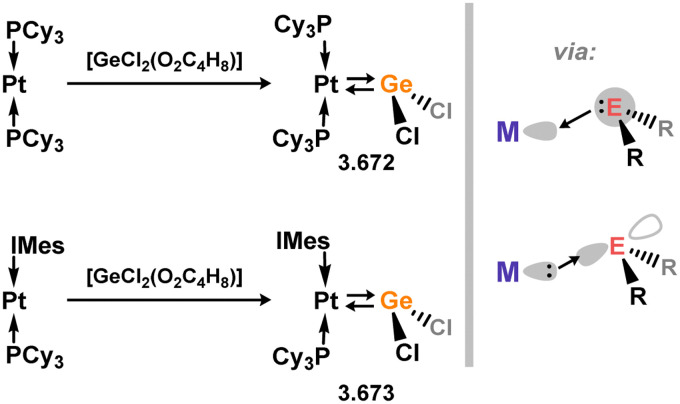
Dichlorogermylene complexes of platinum(0), and the suggested double-σ-bonding model.

**Scheme 168:**
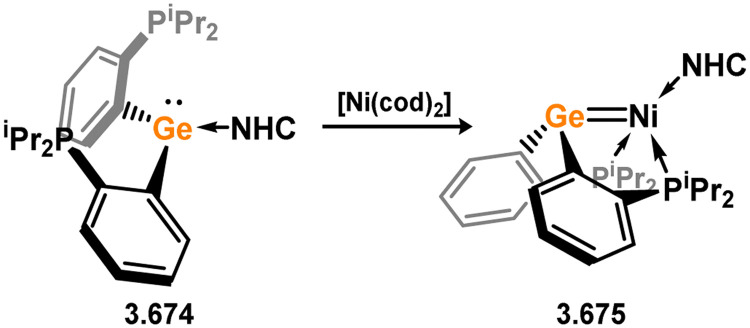
Formation of an acyclic–germylene nickel(0) complex, featuring a Lewis basic germanium centre.

It was later shown by a number of groups that acyclic–germylene complexes of group 10 metals can feature pyramidalised Ge centres, which is indicative of a predominant M → Ge donation, and little or no ‘classic’ L-type ligand behaviour of the germylene. This bonding motif is also thought be brought about by ‘double-σ’ bonding, with simultaneous σ-donation from M → Ge and Ge → M,^308^ reminiscent of the bonding model leading to *trans*-bending in the heavier alkene analogues.^55^ The first such example for Ge was reported by Braunschweig and co-workers, who showed that both [(Cy_3_P)_2_Pt] and [(Cy_3_P)(IMes)Pt] react with [dioxane·GeCl_2_], to yield germylene complexes 3.672 and 3.673 in which the Ge centre is paramidalised (Scheme 167).^309^ Similarly, it was later shown that the phosphine-functionalised bis(aryl)germylene 3.674 reacts with [Ni(cod)_2_] in the formation of 3.675, with a pyramidalised Ge centre (Scheme 168).^310^ Here, accompanying computational analysis located both Ge → Ni and Ni → Ge σ-bonding, forming the so-called σ-double-bond.

**Scheme 169:**
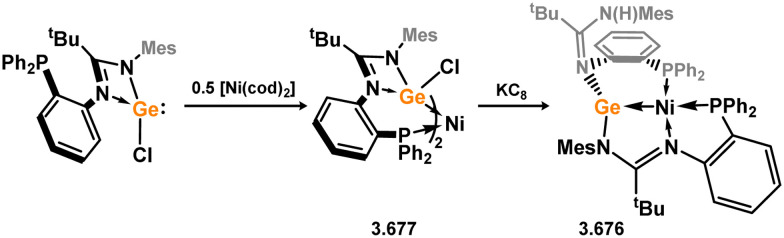
Nickel complexation of a chelating amidinato-germylene, leading a Z-type acyclic–germylene complex.

**Scheme 170:**
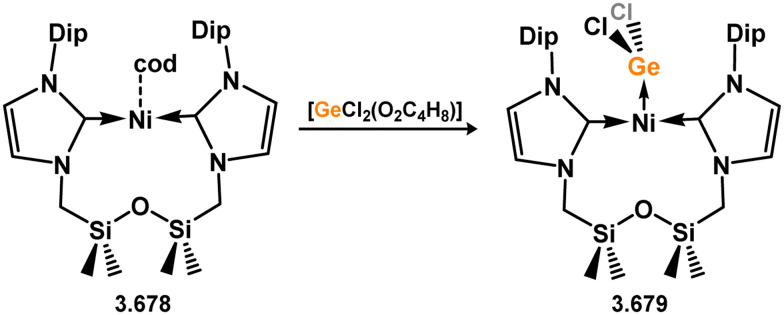
Formation of a T-shaped geometry at nickel(0), driven by the Lewis acidic nature of a dichlorogermylene ligand.

In contrast, complex 3.676, synthesised through the reduction of a bis(chloro-germylene) Ni^0^ complex 3.677, also bears a pyramidalised Ge centre, but rather with a Z-type ligand character (Scheme 169).^229^ That is, the Ni^0^ centre in this species is Lewis basic, and is thus an L-type donor towards Ge. This bonding character is particularly rare for Ni^0^, and was first formally demonstrated by Figueora and co-workers in 2011.^311^ More recently, T-shaped Ni^0^ species featuring Z-type germylene ligands have been realised, in which this geometry at Ni^0^ is strongly indicative of Lewis basic character, as per numerous known Lewis basic Pd and Pt systems.^312–314^ The first such example, reported by Roesler and co-workers, utilises the chelating bis(NHC) stabilised [Ni(cod)] species 3.678, which reacts with [dioxane·GeCl_2_] in forming the Ni-T-shaped dichlorogermylene complex 3.679 (Scheme 170).^250^ Here, the Ge-centred lone pair of electrons was found by computational analysis to not partake in a bonding interaction with Ni, with the only distinct bonding orbitals resembling Ni → Ge donation.

**Scheme 171:**
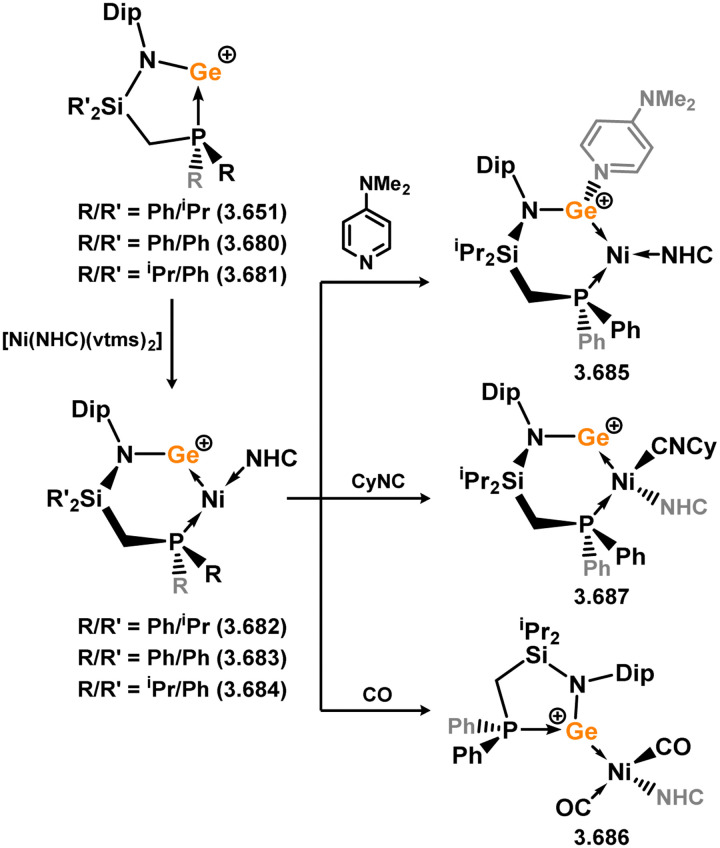
Formation and reactivity of a T-shaped nickel(0) complex, enforced by both geometric constraint and high Lewis acidity of the germylene ligand.

**Scheme 172:**
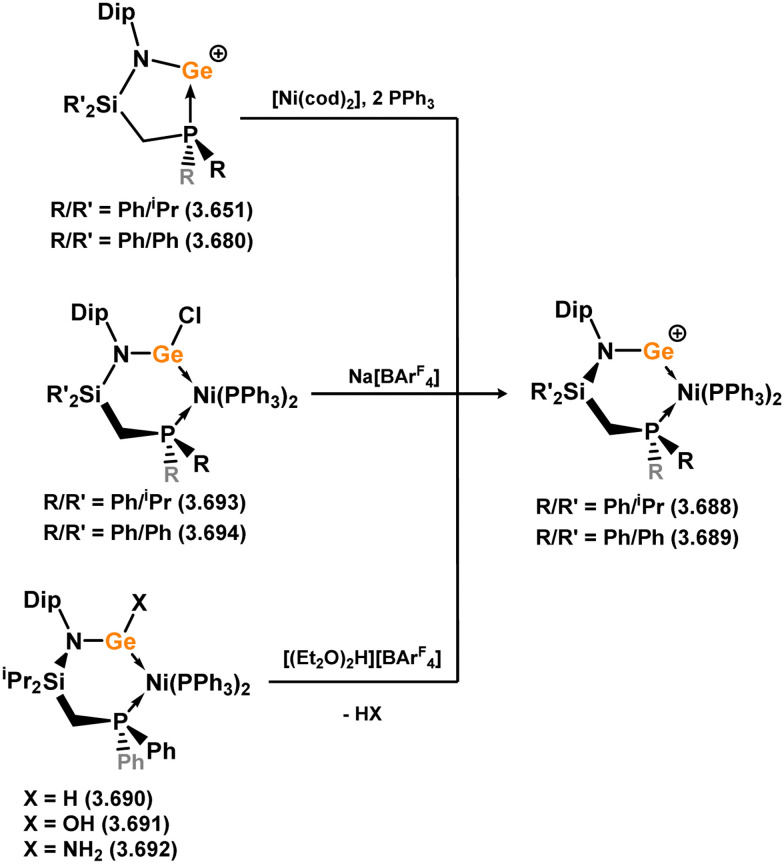
Formation of cationic germylene complexes of nickel(0) *via* various routes.

In our own work, we recently showed that chelating cationic germylene ligands also drive a similar bonding mode in complexation with Ni^0^ (Scheme 171).^315^ Addition of phosphine-chelated cationic germylenes 3.651, 3.680, and 3.681 to [IPr·Ni·{η^2^-(vtms)}_2_] gave ready access to complexes 3.682, 3.683, and 3.684 through alkene displacement. All complexes were found by structural analysis to feature a T-shaped geometry at Ni^0^. This was shown to be highly dependent on the Lewis acidity of the Ge centres. Addition of *N*-bases to these systems led to site-selective binding at Ge, *e.g.* in DMAP adduct 3.685. Typical π-acceptor ligands (CO (3.686), CyNC (3.687)) were shown to bind at Ni^0^, forming tetryhedral complexes. In the former systems, the now more electron rich germylene ligands switch the geometry at Ni from T-shaped to trigonal planar. This thus demonstrates a switch from Z-type to L-type ligand character for Ge^II^, with clear structural effects resulting from this change. Moreover, all described T-shaped complexes were shown to be active catalysis for the hydrogenation of alkenes, whist trigonal planar complexes were not, opening up a new vista for tetrylene ligands in switchable catalysis. The closely related 18-electron Ni^0^ species 3.688 and 3.689, also bearing our developed phosphine-funcationalised cationic germylene ligands are also potent in catalysis, in this case for the hydrosilylation of alkenes.^316^ These complexes were shown to be accessible by differing routes (Scheme 172): (i) the free cationic germylenes can be directly combined with a mixture of [Ni(cod)_2_] and PPh_3_; (ii) the hydride, hydroxide, or amide complexes 3.690, 3.691, and 3.692 can also be treated with the oxonium species [(Et_2_O)_2_H][BAr^F^_4_], in the elimination of H_2_, H_2_O, or NH_3_, respectively; or (iii) the chloride ligand can be abstracted from chloro-germylene complexes 3.693 and 3.694.

**Scheme 173:**
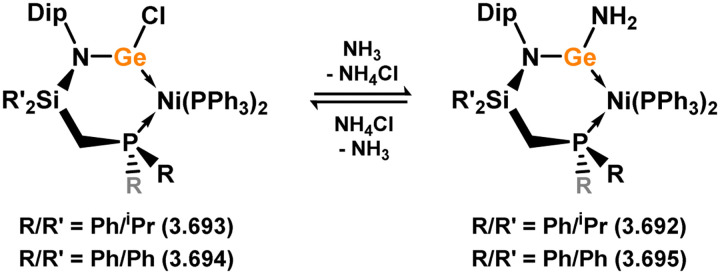
Germylene-centred reversible ammonia activation in an acyclic–germylene nickel(0) complex.

In addition, the neutral chlorogermylene complex 3.693 and 3.694 undergoes non-redoxactive σ-metathesis of the Ge–Cl bond with the N–H bond in ammonia, a reaction in which the added ammonia simultaneously acts as a base in abstraction of HCl, forming bis(amido)germylene complexes 3.692 and 3.695 (Scheme 173).^317^ This reaction is reversible, highlighting the potential for such germylene ligands to behave as bifunctional ligands which can bind a metal centre, whilst also reacting as a Lewis acidic binding site.

**Scheme 174:**
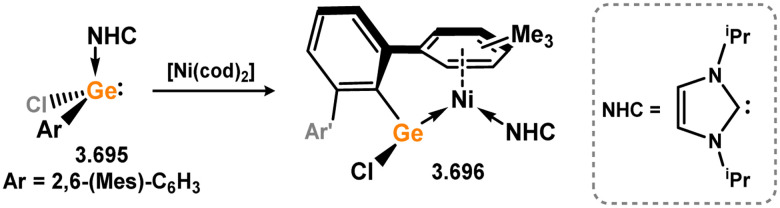
Formation of an acyclic–germylene complex of nickel(0) through Ge–to–Ni NHC migration.

It was additionally recently shown by Campos and co-workers that the NHC-bound (aryl)(chloro)germylene complex 3.696 reacts with [Ni(cod)_2_], leading to NHC-migration to and arene coordination at Ni, in complex 3.697 (Scheme 174).^318^

**Scheme 175:**
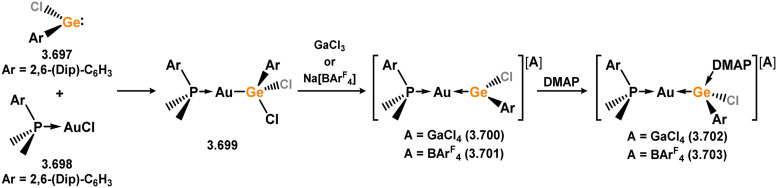
Generation of cationic gold(i) complexes bearing (aryl)(chloro)germylene ligands, and their Ge-centred Lewis base binding chemistry.

Gold complexes which may be described as cationic germylene complexes have been reported by Campos and co-workers, as the only such species for the coinage metals. These were generated by first addition of (aryl)(chlor)germylene 3.698 to bulky phosphino-gold chloride 3.699, leading to germyl-gold complex 3.700*via* gold-to-germanium chloride migration (Scheme 175). A simple chloride-abstraction from these (chloro-germyl)gold(i) complexes led to the target cationic complex 3.701 and 3.702.^319^ The Lewis acidic nature of the Ge centre these species was demonstrated by Ge-centre DMAP binding, leading to adducts 3.703 and 3.704.

#### 3.3.4. Chelating bis(germylene) systems

**Scheme 176:**
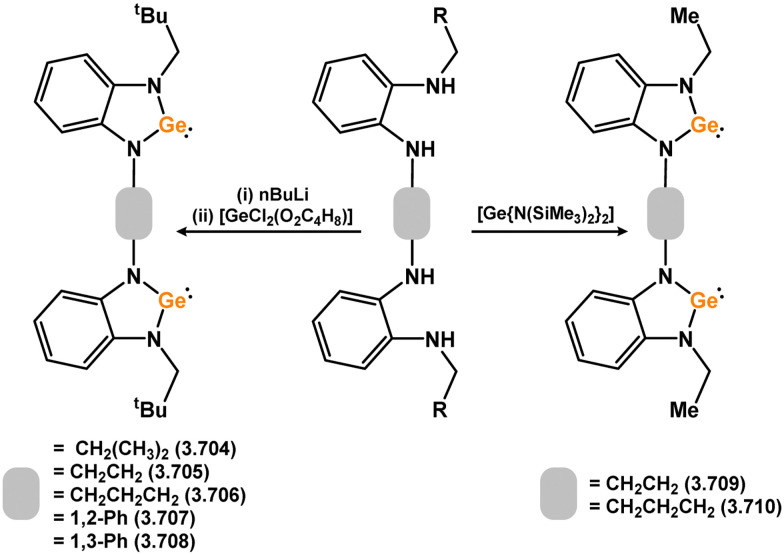
Synthesis of chelating bis(germylene) ligands featuring 5-membered NHGe moieties.

**Scheme 177:**
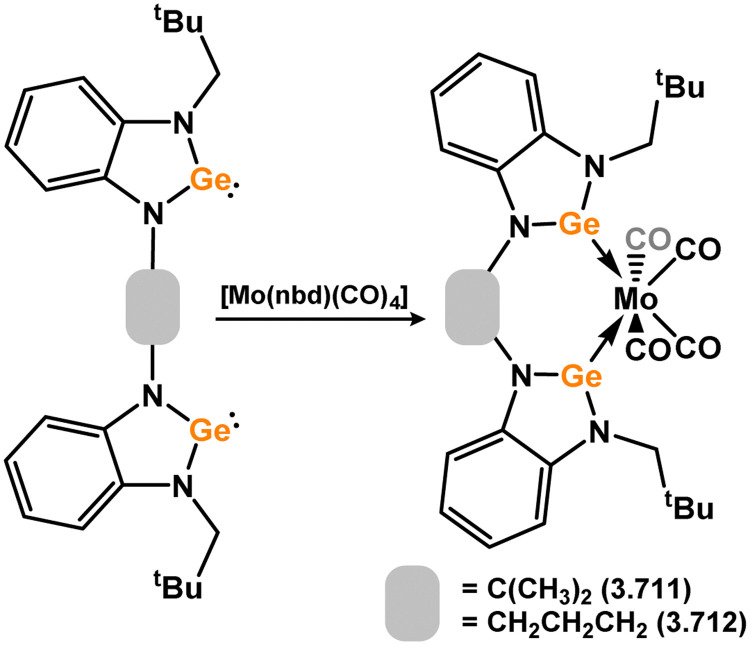
Formation of molybdenum complexes of chelating bis(NHGe) ligands.

Though chelating bis(silylene) ligands have seen considerable attention throughout the past 10–15 years, the same is not true for germylene derivatives, and particularly not so in the field of transition metal coordination chemistry. Early examples were reported by Hahn and co-workers, extending the ‘classical’ West-type scaffold to a number of chelating systems. This involved the generation of a number of novel tetraamines, through a lengthy synthetic protocol, followed by reaction with [{(Me_3_Si)_2_N}_2_Ge] or ^*n*^BuLi/GeCl_2_, leading to *N*-^*n*^Pent bis(germylene) ligands 3.705, 3.706, 3.707, 3.708, and 3.709, in addition to *N*-Et bis(germylene) ligands 3.710 and 3.711 (Scheme 176).^320^ Of these, two were utilised in forming complexes with [Mo(CO)_4_], through reaction with [Mo(nbd)(CO)_4_], forming the desired chelated complexes 3.712 and 3.713 (Scheme 177).^320,321^

**Scheme 178:**
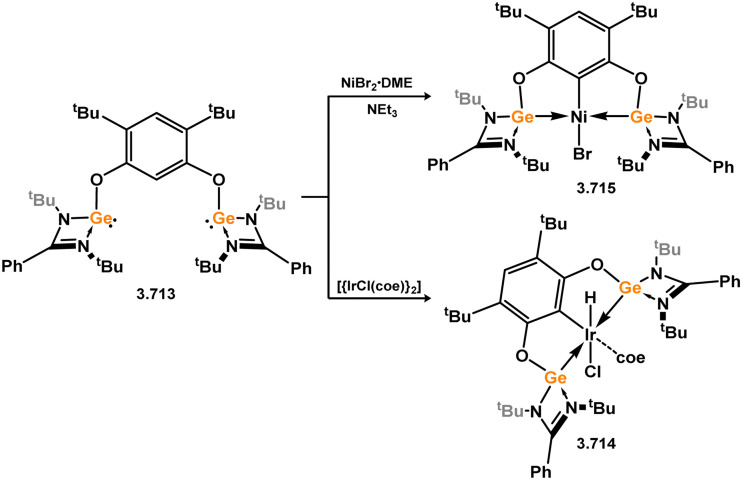
Formation of iridium and nickel complexes bearing a resorcinol-derived chelating bis(germylene) ligand.

**Scheme 179:**
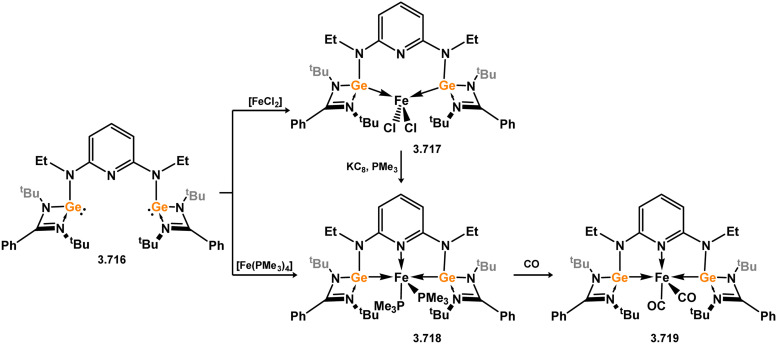
Formation of iron(0) and iron(ii) complexes bearing a bis(amino)pyridine-derived chelating bis(germylene) ligand.

Some years later, Driess and co-workers reported the bis(germylene) ligand 3.714 built upon the 4,6-di-*tert*-butylresorcinol scaffold, analogous to the earlier described Si system 3.296. This germylene ligand was initially utilised in generating iridium complex 3.715, and the catalytic borylation of arenes studied, the reaction rate for the germylene system being slightly slower than that of the silicon derivative (Scheme 178).^188^ Later, the same ligand system was utilised in forming Ni complex 3.716, which was employed in the Sonogashira cross-coupling reaction.^189^ Here, the intermediary copper acetelides were shown to form a bimetallic complex with the catalyst, supported by coordination to one Ge^II^ centre. This was also achieved with the earlier described silylene complex 3.310. This incites that the tetrylene centre is these systems may play a non-innocent role in catalysis, allowing close spatial arrangement of substrates. Further extending the library of chelating bis(germylenes) which utilise the amidinato ligand at Ge^II^, the bis(amino) pyridine-derived ligand 3.717 was developed, reported along-side the silicon analogue 3.311. The bis(germylene), as for the Si system, was employed in accessing complexes with [FeCl_2_], [Fe(PMe_3_)_2_], and [Fe(CO)_2_], though catalytic efficacy was not described (*viz.*3.718, 3.719, and 3.720; Scheme 179).^193^

The above described systems represent the only well-defined transition metal complexes of chelating bis(germylenes). Though these are few, additional interest in bis(germylene) ligands may lead to an expansion of this number.^322^

### 3.3. Stannylene – transition metal chemistry

#### 3.3.1. N-heterocyclic stannylenes

##### 4-Membered

**Scheme 180:**
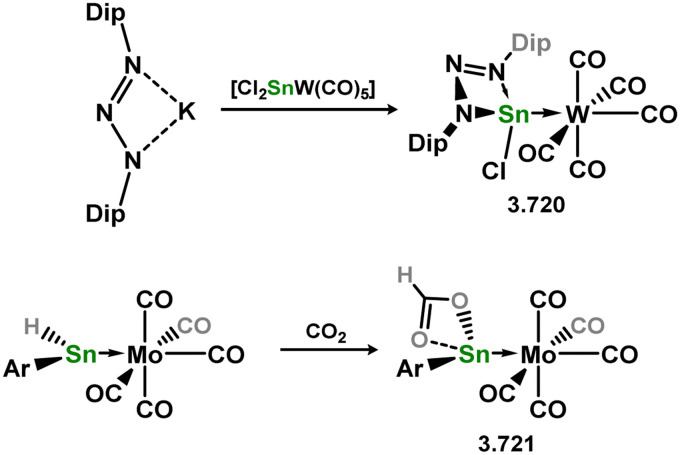
Generation of 4-memebered heterocyclic stannylenes through reactivity of pre-formed stannylenes with small-molecules.

**Scheme 181:**
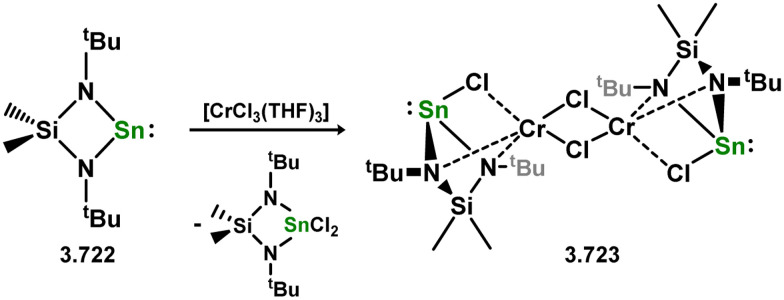
Reactivity of a 4-membered NHSn towards chromium(iii) chloride.

Reports regarding the transition metal coordination chemistry of 4-membered N-heterocyclic stannylenes (NHSns) are considerably more rare than their Si and Ge counterparts, even though *e.g.* amidinate stabilised stannylenes have been well reported.^323–327^ This may be due to the lessened propensity of the Sn^II^ lone pair to partake in bonding interactions, in relation to the inert pair effect on descending the low-valent main group elements. As such, no complexes in this class are known for group 3–5. Two examples of group 6 complexes are known (Scheme 180). The first features a bis(aryl)triazenide-stabilised chloro-stannylene, which was accessed through addition of the potassium salt of the ligand to [(THF)_2_SnCl_2_·W(CO)_5_], forming 3.721 through salt-metathesis.^328^ The second was accessed through insertion of CO_2_ into the Sn–H bond of a hydrido-stannylene complex of [Mo(CO)_5_], forming the formate-bound stannylene complex 3.722, in which the 4-membered ring is formed due to the *κ*^2^-coordination mode of the [HCO_2_]^−^ ligand at Sn^II^.^329^ Additionally, the reaction of NHSn 3.723, bearing the dianionic bis(amido)ligand [Me_2_Si(^*t*^BuN)_2_]^2−^ with CrCl_3_ leads to reduction of the transition metal, generating the corresponding dichlorostannane as a by-product. The formed CrCl_2_ undergoes further Cr–Cl bond activation by NHSn 3.722, forming complex 3.724 (Scheme 181).^232^

**Scheme 182:**
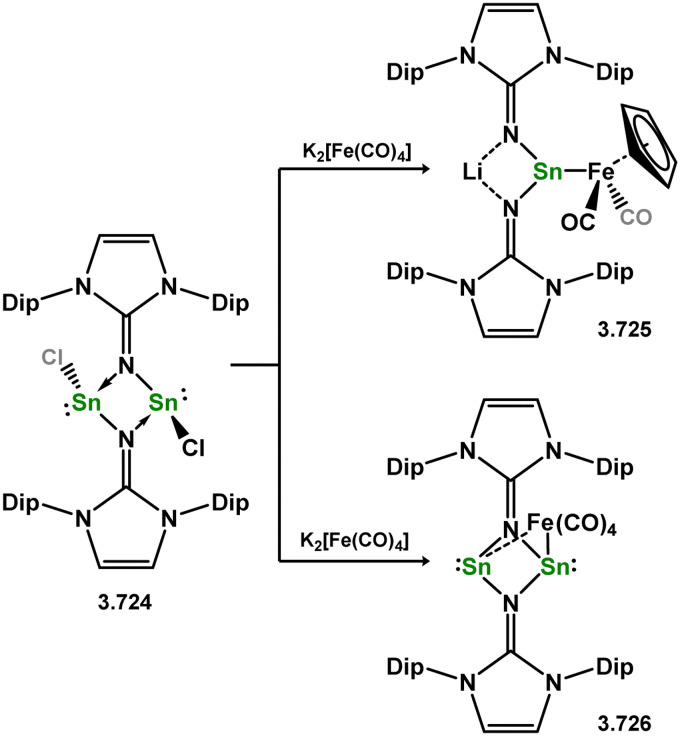
A unique lithium-bridged 4-membered NHSn, and its iron complex.

Inoue and co-workers reported the only example of a group 8 metal complex of a 4-membered NHSn, utilising the bis-N-heterocyclicimine (NHI) stabilised stannylene 3.725, which reacts with [CpFe(CO)_2_]Li in the formation of 3.726, in which a 4-membered [SnN_2_Li] ring is formed (Scheme 182).^330^ Notably, the mixed stannylene-stannylone [Fe(CO)_4_] complex 3.727 was also accessed, achievable only due to the unique bridging nature of the employed NHI ligands.

**Scheme 183:**
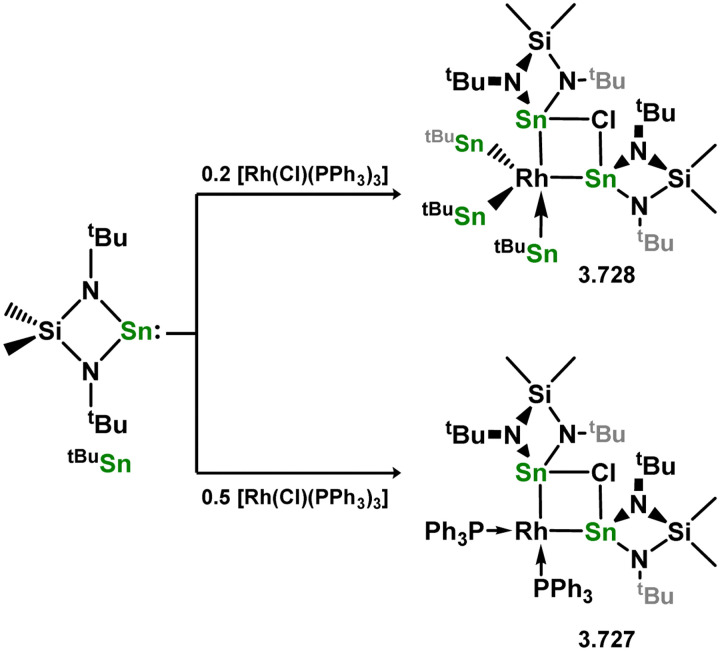
Reactivity of a 4-membered NHSn towards rhodium(i) chloride.

One example of a group 9 complex bearing 4-membered NHSn ligands is known, namely 3.728, which was accessed *via* the addition of 5 equiv. of the two coordinate stannylene 3.722 to [RhCl(PPh_3_)_3_], leading to exchange of all PPh_3_ ligands, and Rh–Cl bond activation (Scheme 183), as was earlier described for the Ge congener (*viz.*3.396).^331^ Addition of just two equiv. of 3.722 leads to complex 3.729 in which the Rh–Cl bond is activated, the Cl^−^ ligand now bridging the two Sn centres.

**Scheme 184:**
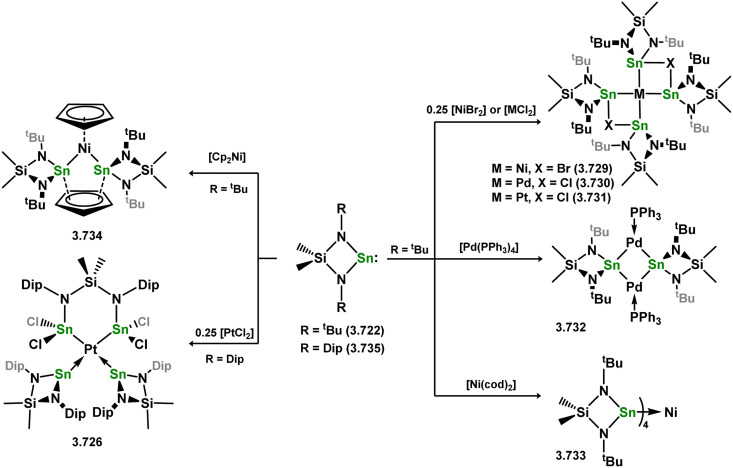
Reactivity of 4-membered NHSn ligands towards group 10 metal species.

Similar metal halide activation was reported by the same group, when reacting NHSn 3.715 with MX_2_ species (Scheme 184; M = Ni, X = Br; M = Pd, Pt, X = Cl). Addition of 4 equiv. leads to the formation of what would be formally described as bis(stannyl)-bis(stannylene) complexes, though the halide ligands each bridge two Sn centres in all cases (*viz.*3.730, 3.731, and 3.732).^223^ In the reporting manuscript, it is described that the ^1^H NMR spectra for these compounds indicates a single ligand environment, so a rapid exchange processes is occurring on the NMR time scale. Addition of 1.5 equiv. of the same ligand to [Pd(PPh_3_)_4_] leads to the dimeric complex 3.733, with three NHSn ligands bridging the two Pd centres, whilst the tetra(stannylene)nickel(0) complex 3.734 is readily accessed through the addition of 3.722 to [Ni(cod)_2_].^332^ In an additional publication, the described insertion chemistry was also extended to [Cp_2_Ni], whereby the NHSn inserts into the Ni–Cp bond, generating Cp-bridged bis(stannyl)nickel complex 3.735.^333^ More recently, Coles and co-workers demonstrated that 4 equiv. of the bulky 4-membered NHSn ligand 3.736 react with PtCl_2_ on heating, forming mixed (stannyl)(stannylene)-Pt complex 3.737, though the room temperature addition of the same ligand to [PtCl_2_(PPh_3_)_2_] or [PtCl_2_(cod)] leads insertion into the Pt–Cl bond of these species.^334^

**Scheme 185:**
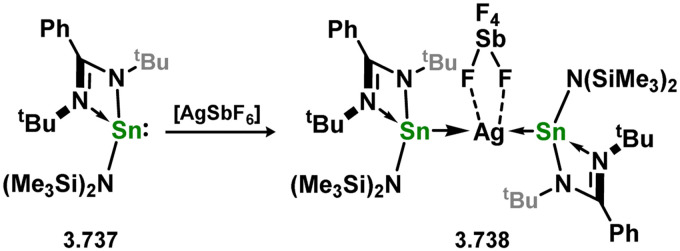
Reaction of an amidinato-stannylene ligand towards silver(i).

One formal stannylene complex of a group 11 metal is reported through the reaction of amidinato-stannylene 3.738 with Ag[SbF_6_], forming the Ag^+^ complex 3.739,^108^ in which two stannylene ligands coordinate a single silver centre (Scheme 185).

No 4-membered NHSn complexes of the group 12 metals are reported.

##### 5-Membered

**Scheme 186:**
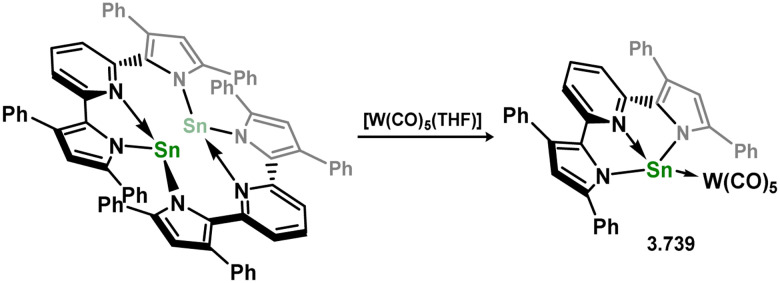
Formation of a bis(pyrrolodyl)stannylene complex of tungsten. There is no Sn–Sn interaction observed in the bis(stannylene) starting material.

**Scheme 187:**
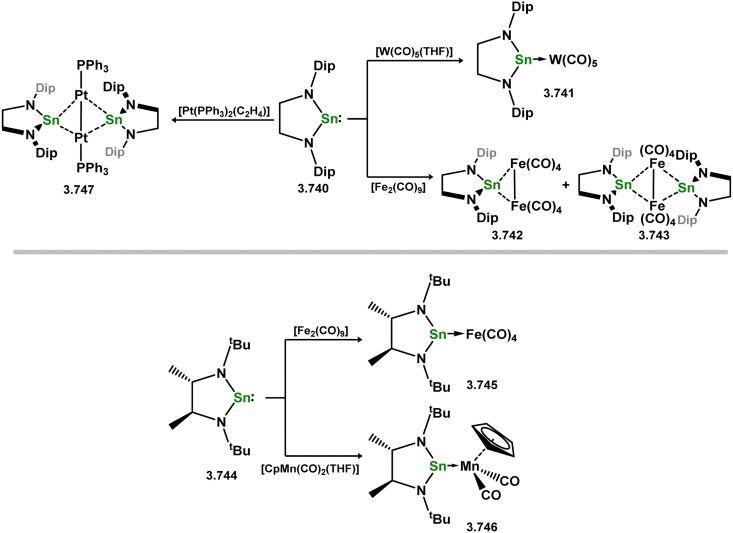
Reactivity of 5-membered NHSn ligands towards various transition metal species.

Complexes bearing this ligand class are similarly uncommon relative to their lighter counterparts. None are known for groups 3–5. A number of species which can be loosely described as 5-membered stannylene group 6 TM complexes are known (*e.g.* stabilised by 2,2′-Bipyridine and 1,10-Phenanthroline systems,^335^ Schiff-base ligands,^336^ or as bicyclic 10-membered systems.^337^ Of these, only the latter are ‘true’ stannylenes, which demonstrated classical Lewis basic coordination to a group 6 metal fragment, in 3.740 (Scheme 186). In addition to this example, the stable 5-membered NHSn 3.741,^338^ isostructural to broadly applied saturated NHCs, has also been shown to react with [(CO)_5_W·THF] in the formation of adduct 3.742 (Scheme 187).^339^ Alongside the above report, it was also described that NHSn 3.740 reacts with [Fe_2_(CO)_9_] in forming a mixture of 1 : 1 and 2 : 1 Fe : Sn products, 3.743 and 3.744. An example of a chiral 5-membered NHSn was developed, namely 3.745, which divergently forms the monomeric [Fe(CO)_4_] complex 3.746 under similar reaction conditions. This novel chiral NHSn also reportedly reacts with [CpMn(CO)_2_THF] in the formation of complex 3.747, which was not reported for aryl-substituted NHSn 3.733. It should be noted that, although NHSn 3.737 is indeed chiral, all reported reactions used racemic mixtures of this ligand, leading to only racemic product complexes. Again described in this report, it was shown that aryl-functionalised 3.733 reacts with [(Ph_3_P)_2_Pt(η^2^-C_2_H_4_)] in the formation of dimeric complex 3.748.

**Scheme 188:**
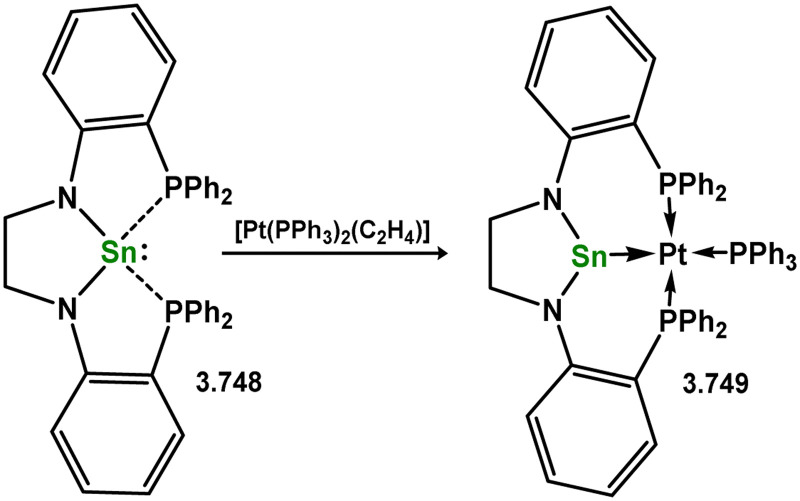
Reaction of phosphine-functionalised 5-membered NHSn ligand towards platinum(0).

**Scheme 189:**
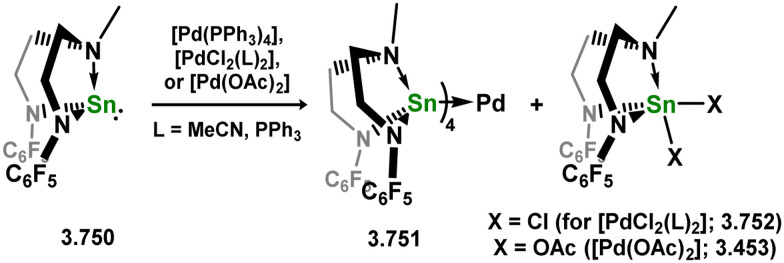
Reaction of an amine-appended bicyclic stannylene ligand towards various palladium species.

A handful of further examples of group 10 complexes featuring 5-membered NHSns are known. The Goicoechea group reported the phosphine-appended stannylene 3.749, the heavier congener of the earlier described germylene 3.507, the former being considerably less tolerant towards transition metal halides than the latter, forming intractable mixtures and elemental tin on reaction, for example, with [MCl] (M = Cu–Au). However, reaction of 3.748 with halide-free [(Ph_3_P)_2_Pt(η^2^-C_2_H_4_)] led to formation of chelation complex 3.750, in loss of ethylene and one Ph_3_P ligand (Scheme 188).^257^ Examples of bicyclic 10-membered NHSn complexes of group 10 metals are also known, featuring discreet 5-membered stannylene rings, and hence will be discussed here (Scheme 189). The first example bears stannylene 3.751, in which the tin centre is stabilised through intramolecular R_3_N → Sn donation. Addition of this ligand to either Pd^0^ (*i.e.* [Pd(PPh_3_)_4_]) or Pd^II^ (*i.e.* [PdCl_2_·(NCMe)_2_], [PdCl_2_·(PPh_3_)_2_], [Pd(OAc)_2_]) sources led in all cases to the generation of tetra-stannylene complex 3.752, as well as chloro- or acetyl-stannanes 3.753 and 3.754 as by-products for respective Pd^II^ reagents.^340^

**Scheme 190:**
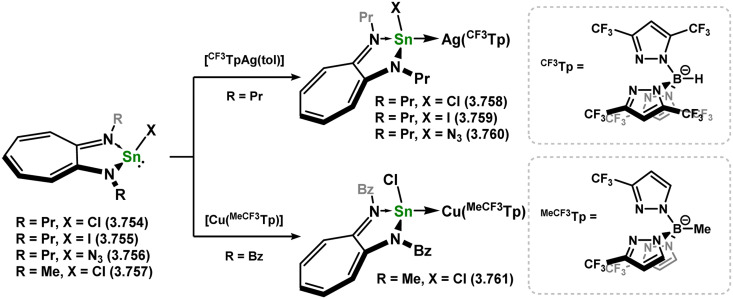
Reactivity of tropiminioato-stannylene ligands towards coinage metal species.

A small number of 5-membered NHSn complexes of group 11 metals are known, all featuring the aminotroponiminate ligand scaffold, and all reported by the group of Dias. These essentially mirror the related germylene complexes discussed earlier. Specifically, ligands 3.755, 3.756, 3.757, and 3.758, bearing halide or azide ligands at Sn^II^, react with trispyrazolylborate copper(i) and silver(i) complexes, forming Sn → M adducts in all cases (*viz.*3.759, 3.760, 3.761, and 3.762, Scheme 190).^254,255,341^

##### 6-Membered

**Scheme 191:**
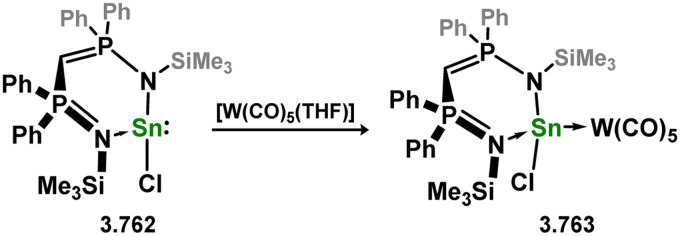
Formation of a tungsten(0) complex of a bis(iminophosphoranyl)methane-derived 6-membered stannylene ligand.

A small number of 6-membered NHSns bearing two-coordinate tin centres (*i.e.* dianionic ligands) are known, with a much greater number of Nacnac derivatives bearing three-coordinate tin. Still, transition metal complexes of this ligand class are sparse, the vast majority being [Fe(CO)_4_] adducts. One example is known for the group 6 elements, featuring the bis(iminophosphoranyl)methane-derived stannylene ligand 3.763 in the [W(CO)_5_] complex 3.764, (Scheme 191).^342^

**Scheme 192:**
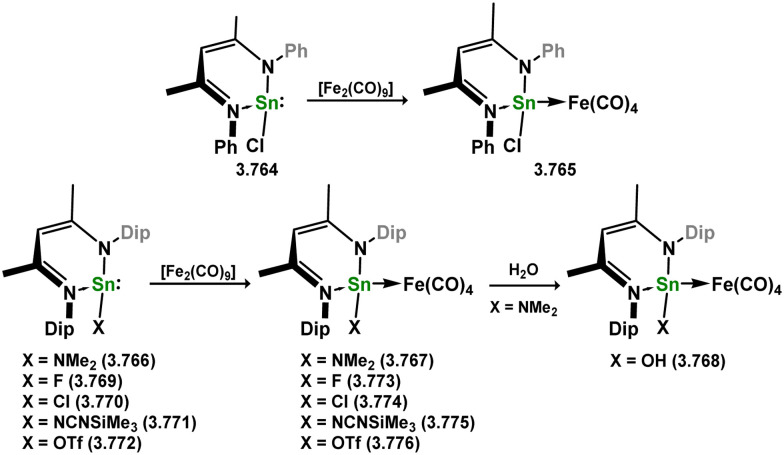
Formation of a range of Nacnac-stannylene ligated iron(0) carbonyl complexes.

**Scheme 193:**
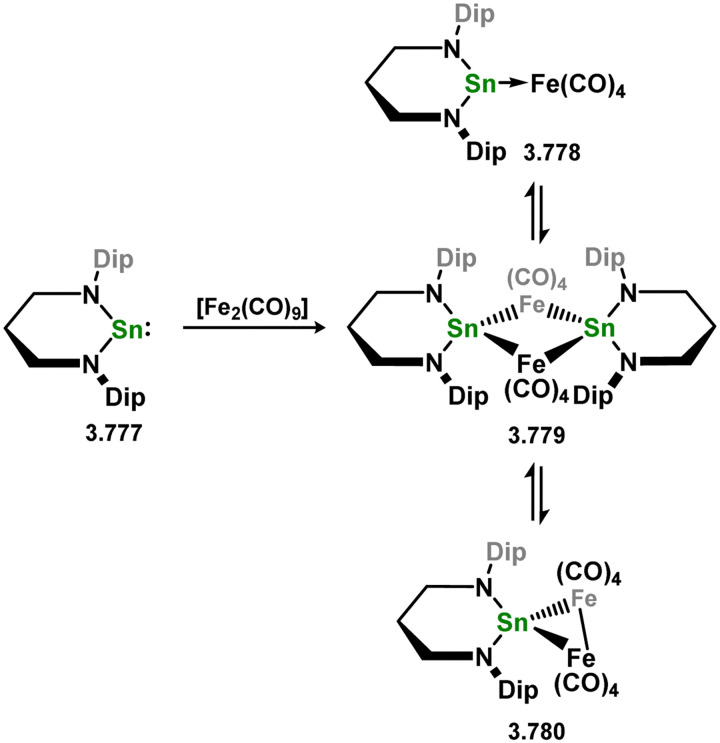
Fluctional behaviour of an iron(0) 6-membered NHSn complex.

The majority of the remaining 6-membered NHSn complexes are iron-centred (Scheme 192). The first involved a Ph-Nacnac stabilised chloro-stannylene ligand 3.765, which reacts with [Fe_2_(CO)_9_] in the formation of 3.766, akin to related chemistry of the Ge^II^ congener.^260^ Roesky and co-workers have reported a number of [Fe(CO)_4_] complexes bearing Dip-Nacnac supported stannylene ligands, with various substituents at tin. Reaction of the Me_2_N-derivative 3.767 with [Fe_2_(CO)_9_] led to the formation of complex 3.768, with undergoes hydrolysis of the Sn–N in the presence of water, forming 3.769.^343^ Similarly, fluoro- (3.770), chloro- (3.771), carbodimido- (3.772), and triflic-stannylene (3.773) ligands readily react with [Fe_2_(CO)_9_] in formation of their [Fe(CO)_4_] adducts 3.774, 3.775, 3.776, and 3.777.^263,344,345^ The two-coordinate stannylene 3.778 also reacts with [Fe_2_(CO)_9_] monomeric and dimeric mono-adducts 3.779 and 3.780, as well as the 2 : 1 Fe : Sn complex 3.781 (Scheme 193).^339^

**Scheme 194:**
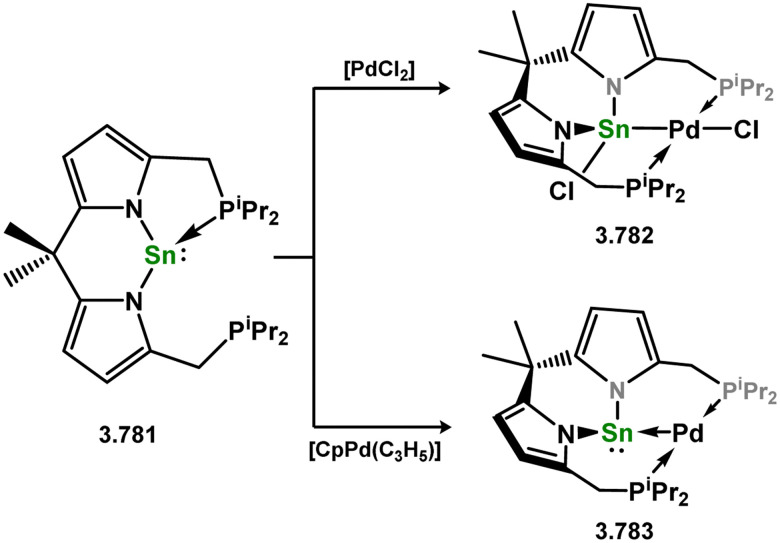
Formation of palladium complexes bearing a phosphine-appended 6-membered NHSn ligand.

Beyond these examples, one further formal 6-membered NHSn transition metal complex is known (Scheme 194). Cabeza and co-workers have recently demonstrated that the reaction of their phosphine appended system 3.782 with [CpPd(C_3_H_5_)] leads to the formation of Pd^0^ complex 3.783, in which the Sn centre behaves as a Z-type ligand, borne out by the T-shaped geometry at Pd and pyramidalization at Sn, as per the earlier described Ge derivative.^346^ The same stannylene ligand reacts with PdCl_2_ through insertion into one Pd–Cl bond, generating stannyl complex 3.784.

#### 3.3.2. Further cyclic stannylene systems

**Scheme 195:**
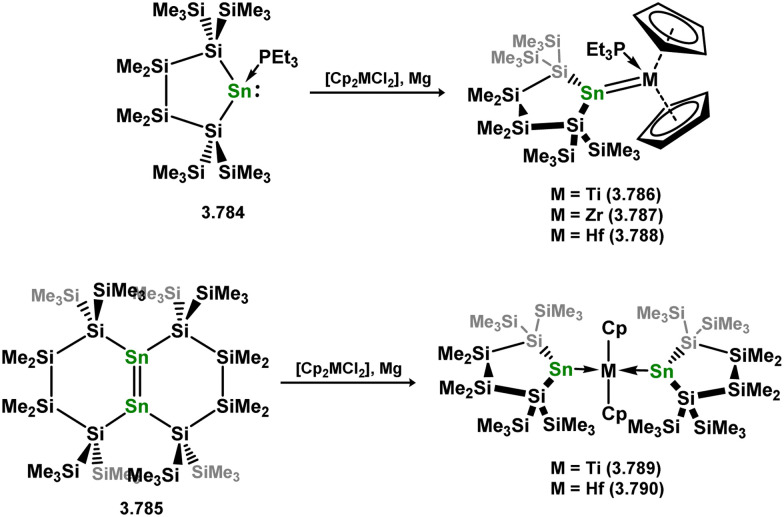
Formation of group 4 complexes bearing cyclic bis(silyl) stannylene ligands.

**Scheme 196:**
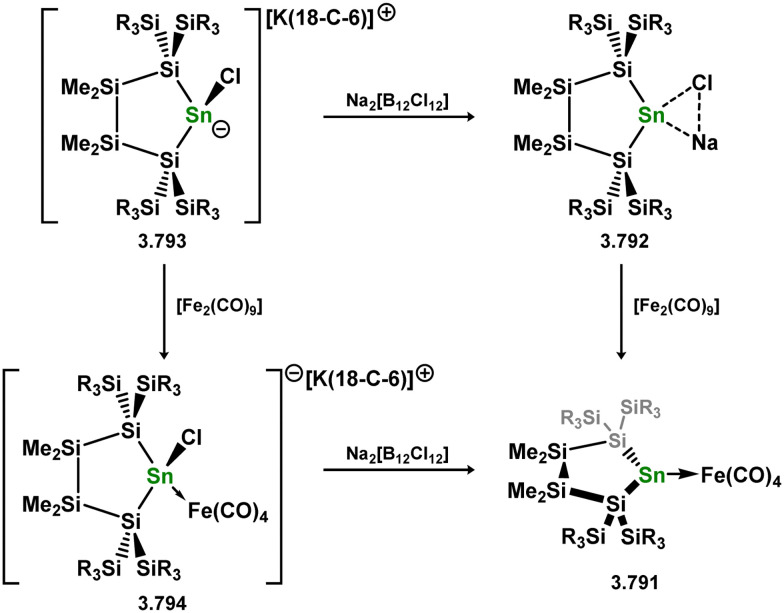
Synthesis of an iron(0) complex bearing a cyclic bis(silyl)stannylene ligand.

In addition to the array of N-heterocyclic stannylene systems which have been discussed, a small number of transition metal complexes featuring further cyclic stannylene ligands have been reported. All such examples employ stannylene derivatives 3.785 and 3.786, in a selection of Ti, Zr, and Hf cyclopentadienyl complexes,^347^ and one [Fe(CO)_4_] complex (Scheme 195).^348^ The former group 4 mono-stannylene complexes 3.787, 3.788, and 3.789 were accessed through the addition of phosphine-stabilised stannylene 3.777 to [Cp_2_MCl_2_] (M = Ti–Hf) in the presence of Mg. The similar reaction utilising the bicyclic distannene 3.778 in place of 3.777 leads instead to the bis(stannylene) complexes 3.790 and 3.791. In contrast, iron complex 3.792 was generated through the reaction of sodium stannyl 3.793 with [Fe_2_(CO)_9_], in the elimination of NaCl and [Fe(CO)_5_] (Scheme 196). Alternatively, the potassium stannyl derivative 3.794 is reacted with [Fe_2_(CO)_9_], leading to the stannyl [Fe(CO)_4_] adduct 3.795, which eliminates NaCl upon K/Na exchange.

#### 3.3.3. Acyclic stannylene complexes

**Scheme 197:**
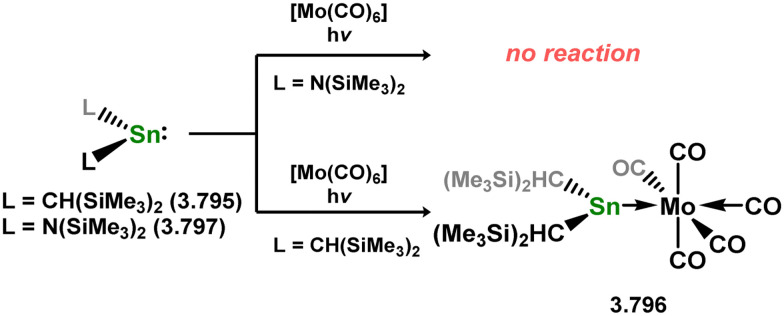
Divergent behaviour of bis(amido)- and bis(alkyl)-stannylene ligands towards tungsten ligation.

**Scheme 198:**
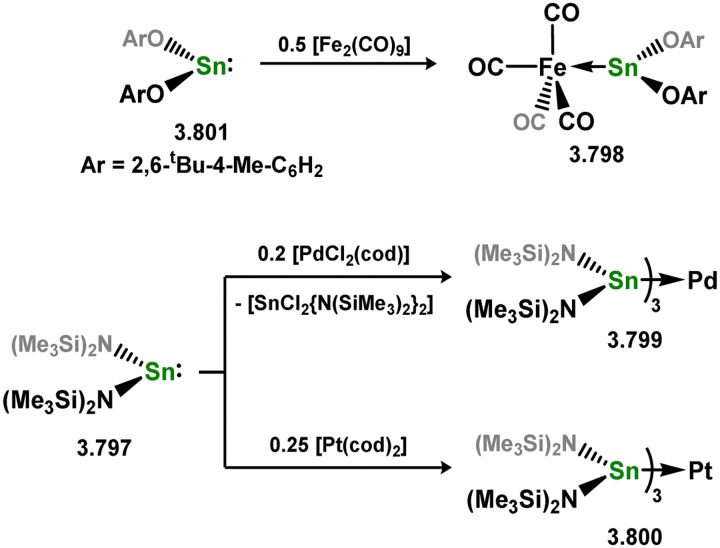
The first examples of crystallographically characterised acyclic stannylene complexes if iron(0), palladium(0), and platinum(0).

**Scheme 199:**
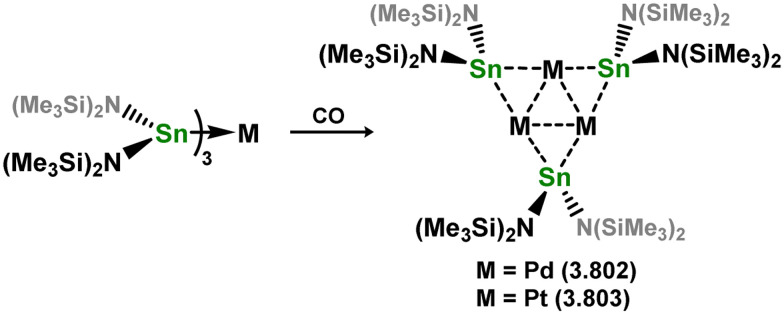
Formation of palladium and platinum trimers supported by acyclic bis(amido)stannylene ligands.

Again in contrast to silylene derivatives, base-free acyclic stannylene-transition metal complexes have generally been accessed through direct addition of isolable stannylene ligands to transition metal fragments, rather than *in situ* generation methods which may otherwise be employed. We note here that utilising push–pull stabilisation even the otherwise polymeric parent stannylene [SnH_2_] can be isolated in the coordination sphere of a TM.^349^ Regarding base-free examples, the first reported example of a transition metal complex of an acyclic stannylene came alongside the first report of bis(alkyl)stannylene 3.796, in 1973, whereby its addition to [Mo(CO)_6_] under UV irradiation led to the formation of 3.797 (Scheme 197).^54^ Although this species was not structurally authenticated, it is perhaps the earliest example of a heavier tetrylene-transition metal complex. Notably, it is also described that the bis(amido)stannylene 3.798 does not react with [Mo(CO)_6_] under the same reaction conditions, attesting to the greater basicity of the bis(alkyl)stannylene system.^350^ It was 12 years later that the first crystallographically characterised derivatives were reported, also by Lappert and co-workers, in iron complex 3.799, and palladium and platinum complexes 3.800 and 3.801, bearing isolable bis(aryloxy)- and bis(amido)-stannylenes (3.802 and 3.797, respectively; Scheme 198).^294,351,352^ The synthetic route for the formation of Pd complex 3.799 is of interest as the stannyene behaves both as the reducing agent and stabilising ligand. Further reaction of group 10 compounds 3.799 and 3.800 with carbon monoxide led to the cluster compounds 3.803 and 3.804, in which three stannylene ligands bridge three M centres (Scheme 199).^353^

**Scheme 200:**
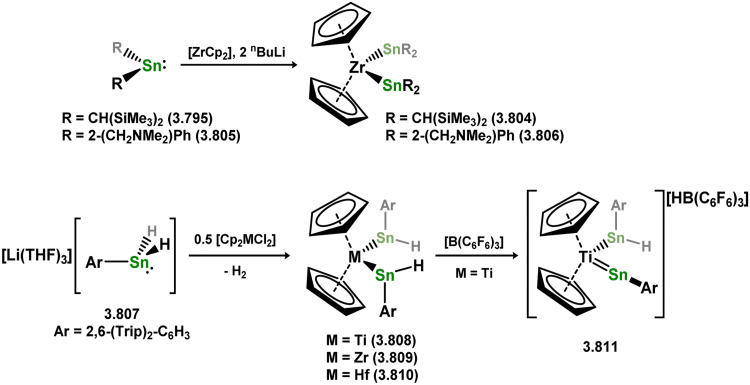
Synthesis of bis(stannylene) complexes of group 4 metallocenes.

No acyclic stannylene complexes of the group 3 elements are known, but a handful of examples have been accessed for the group 4 elements (Scheme 200). The first of these, complex 3.805, was synthesised through the *in situ* generation of zirconocene (*i.e.* [Cp_2_Zr]), which is trapped by two equiv. of Lappert's bis(alkyl)stannylene.^354^3.804 features slightly contracted Zr–Sn interactions when compared with the sum of the covalent radii for these elements, indicative of a degree of Zr → Sn π-backbonding. The amine-functionalised stannylene 3.806 was later utilised in accessing the closely related bis(stannylene) complex 3.807, using a similar synthetic route.^355^ Wesemann and co-workers later reported hydrido-stannylene group 4 complexes, accessed using a different synthetic strategy. Here, hydridostannyl lithium species 3.808 was shown to react with metallocene-dichloride complexes [Cp_2_MCl_2_] (M = Ti–Hf), leading to salt-metathesis and H_2_ elimination, forming bis(stannylene) complexes 3.809, 3.810, and 3.811.^356^ All complexes exhibit low-field shifted Sn–H signals in their ^1^H NMR spectra (12.63–13.27 ppm) when compared with the free hydrido-stannylene (7.87 ppm),^357^ but in keeping with other reported hydrido-stannylenes.^21^ It was also demonstrated that one hydride ligand could be abstracted from titanium complex 3.801, leading to cationic 3.812, with one Ti-*Sn*-L angle now close to linearity indicative of amplified Ti → Sn π-back bonding.

**Scheme 201:**
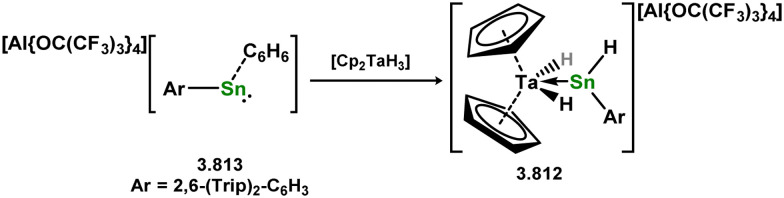
Synthesis of a (aryl)(hydrido)stannylene adduct of tantalum.

The same group later reported the closely related tantalum complex 3.813, being the only representative example of a group 5 acyclic stannylene complex (Scheme 201).^358^ This complex was synthesised *via* the addition of cationic stannylene 3.814 to [CpTaH_3_], leading to hydride migration from Ta to Sn. Here, the observed ^1^H NMR shift for the Sn–H (15.55 ppm) is considerably down field when compared with described group 4 derivatives.

**Scheme 202:**
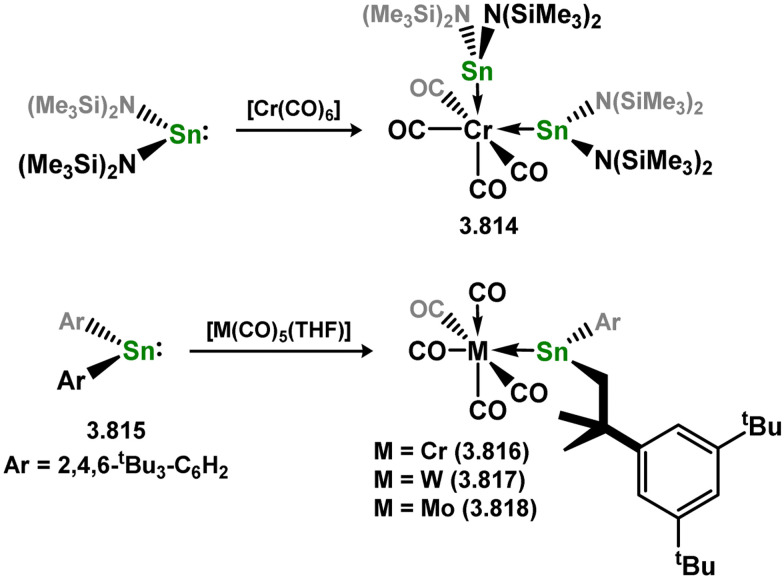
Formation of group 6 complexes bearing acyclic stannylene ligands.

**Scheme 203:**
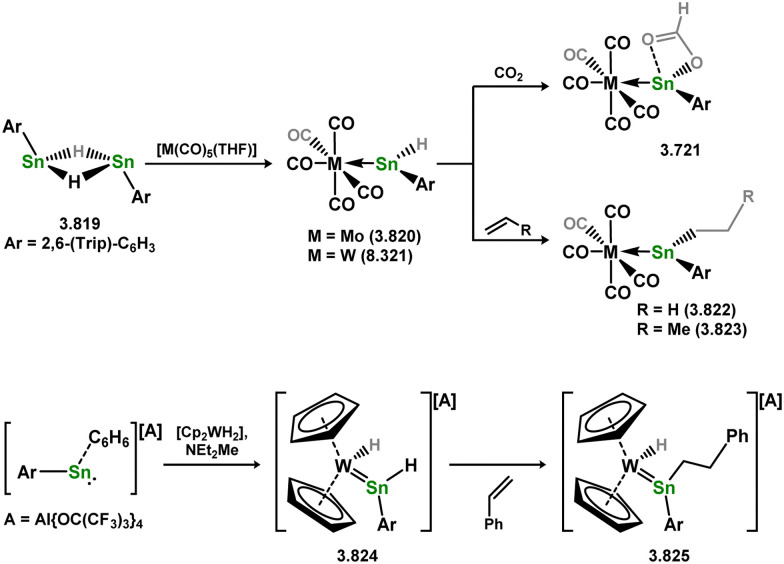
Synthesis of molybdenum and tungsten complexes bearing (aryl)(hydrido)stannylene ligands, and subsequent insertion chemistry of the Sn–H bond.

The first examples of group 6 complexes featuring acyclic stannylenes were reported by Lappert and co-workers, utilising their bis(amido)stannylene ligand 3.797. The addition of two equiv. of this ligand to [Cr(CO)_6_] led to the bis(stannylene) complex 3.815, with stannylene ligands in a *cis*-arrangement (Scheme 202).^359^ Soon after this report, it was shown that bis(aryl)stannylene 3.816 also reacts with the group 6 metal(0) hexacarbonyl complexes. This situation is slightly more complex, however. 3.813 exists as the distannene form, which dissociates in solution leading to H-migration/rearrangement in one [2,4,6-^*t*^BuC_6_H_2_] group, forming an (alkyl)(aryl)stannylene which reacts with [M(CO)_6_] to form complexes 3.817, 3.818, and 3.819.^360,361^ Considerably later, in 2021, Power and co-workers showed that their (aryl)(hydrido)stannylene 3.820 reacts with [Mo(CO)_5_·THF] in forming complex 3.821 (Scheme 203).^329^ This species undergoes CO_2_ insertion into the Sn–H bond under non-forcing conditions forming earlier described 3.714, which was extended to the catalytic hydroboration of CO_2_ with HBpin. The directly analgous hydrido-stannylene tungsten complex (*viz.*3.822) was also accessed through the same synthetic methodology, whereby insertion of alkenes into the Sn–H bond was demonstrated, forming 3.823 and 3.824.^362^ Attempts were made to extend this to alkene hydrosilylation catalysis, but it was found computationally that the barrier to Sn–C σ-metathesis was to great, at more than 70 kcal mol^−1^. A similar (aryl)(hydrido)stannylene complex of tungsten was also reported by Wesemenn and co-workers, 3.825, which was accessed through the addition of stannylene cation 3.813 to the tungsten dihydride species [Cp_2_WH_2_], followed by reaction with EtMe_2_N.^358^ The hydride moiety in 3.823 inserts alkenes in forming 3.826, as per the above described complex reported by Power and co-workers, and can also be abstracted by an NHC in forming a neutral metallostannylene (*vide infra*).

**Scheme 204:**
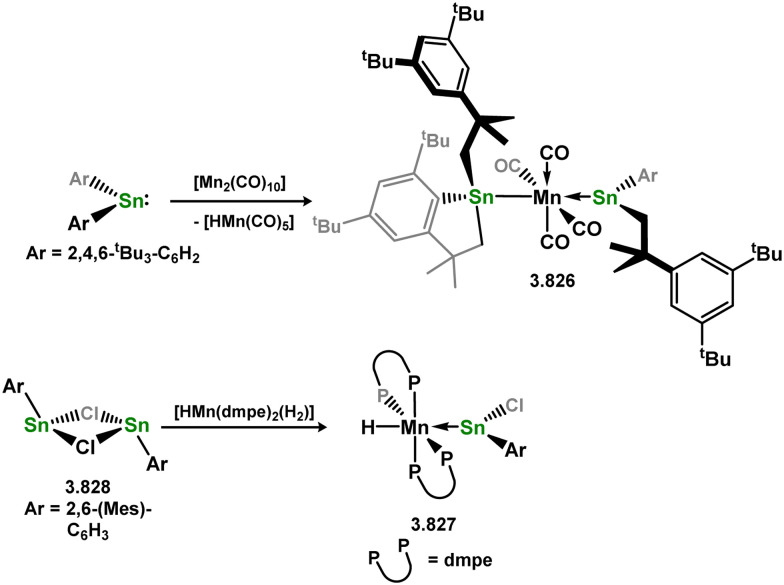
Formation of a acyclic stannylene ligated manganese complexes.

The initial example of a group 7 acyclic stannylene complex involved the rearranged bis(aryl)stannylene 3.815; two equiv. of the isomerised form react with [HMn(CO)_5_], leading to complex 3.827 in which one stannylene ligand binds Mn, and the second undergoes C–H activation, presumably in loss of H_2_, forming a novel stannyl ligand (Scheme 204).^363^ The somewhat related phosphine-stabilised Mn^I^ species 3.828 has also been used in accessing the chloro-stannylene complex 3.829, which was utilised as a precursor to a heavier alkylidyne derivative (*vide infra*).^364^

**Scheme 205:**
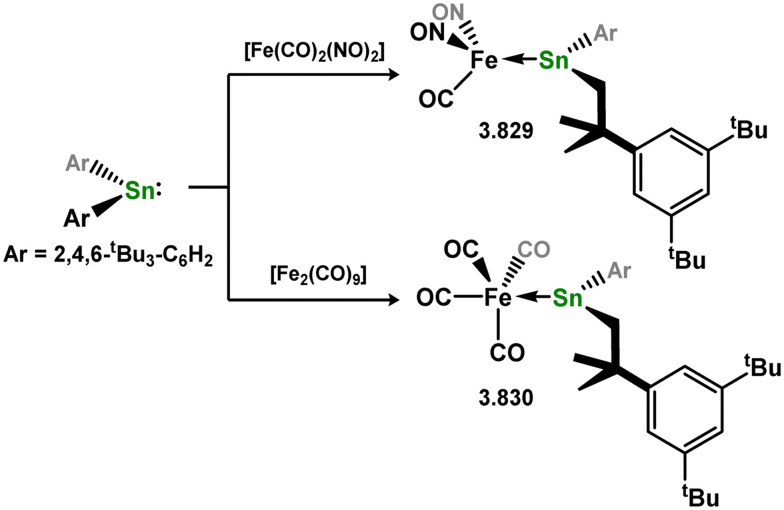
Formation of iron complexes of a rearranged bis(aryl)stannylene ligand.

**Scheme 206:**
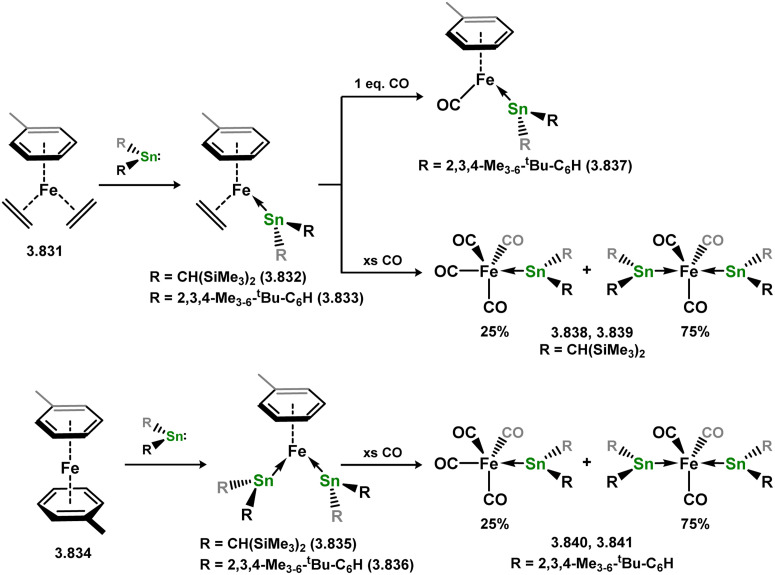
Ready formation of acyclic-stannylene iron(0) complexe utilising toluene-stabilised iron(0) synthons.

Numerous group 8 complexes in this class are known, the initial example being the bis(aryloxy)stannylene complex 3.798 describe above (Scheme 198). Following this, two examples were reported featuring the isomerised form of bis(aryl)stannylene 3.808, namely 3.830 and 3.831, with [Fe(CO)_4_] and [Fe(CO)(NO)_2_], synthesised through direct addition of the stannylene ligand to [Fe_2_(CO)_9_] and [Fe(CO)_2_(NO)_2_], respectively (Scheme 205).^363,365^ Schneider and co-workers later reported on the synthesis of toluene-bound iron(0) complexes of acyclic stannylene ligands, employing the metal-vapour synthesised iron(0) complexes (Scheme 206). In an initial report, it was shown that the bis-ethylene derivative 3.832 reacts with one equiv. of bis(alkyl) or bis(aryl) stannylenes to yield mono-stannylene complexes 3.833 and 3.834.^366^ Further substitution of the remaining ethylene ligand apparently is not possible. However, it was later reported that the direct reaction of the bis(toluene) complex 3.835 with two equiv. of the same stannylene ligands did lead to the bis-ligated complexes, 3.836 and 3.837.^367^ Addition of carbon monoxide to these various species led to a range of mono- and bis-stannylene complexes of [Fe(CO)_*n*_] (*n* = 1 (3.838); 3 (3.839 and 3.840); 4 (3.841 and 3.842)).

**Scheme 207:**
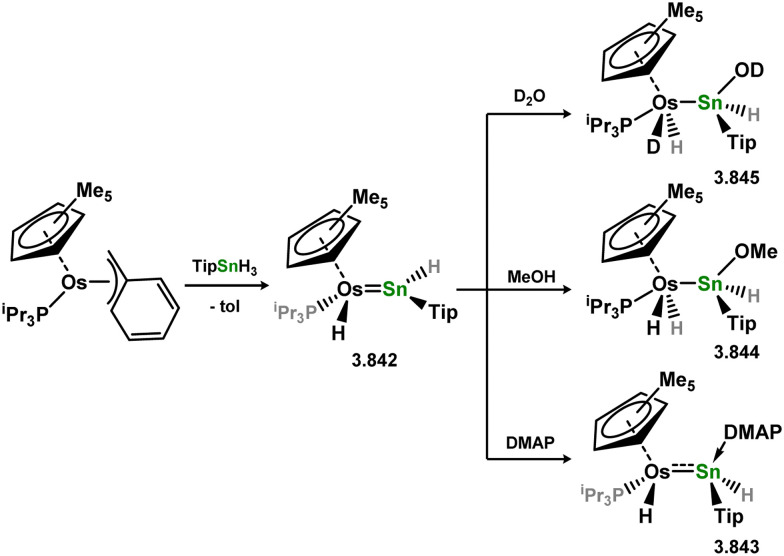
Formation of an osmium complex bearing an (aryl)(hydrido)stannylene ligand, and its subsequent reactivity.

**Scheme 208:**
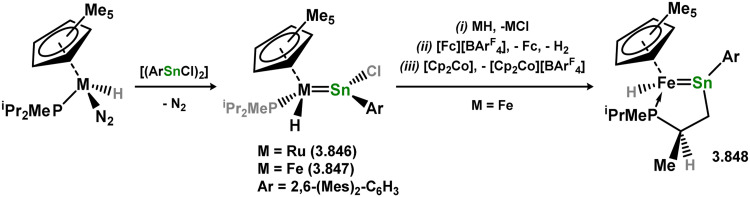
Synthesis of ruthenium and iron complexes bearing an (aryl)(chloro)stannylene ligand, and the further reactivity of the iron species leading to a novel phosphine-appended stannylene ligand.

Fe, Ru, and Os complexes of acyclic stannylenes have all been reported by the group of Tilley, generally featuring a Cp*M (M = Fe–Os) fragment at their core. The first report described the synthesis of the osmium system, which was achieved through the reaction of TripSnH_3_ to the allyl osmium complex [Cp*Os(^*i*^Pr_3_P)CH_2_Ph)], leading to elimination of toluene, and H-migration to Os in forming the (aryl)(hydrido)stannylene complex 3.843 (Scheme 207).^368^ Reactivity studies indicated a Lewis acidic Sn centre, indicated by both base coordination (3.844) and MeOH(D) addition across the Os–Sn bond (3.845 and 3.846) as well as tautomerisation to the metallostannylene form *via* a further H-migration (*vide infra*). This was later followed by the synthesis of the (aryl)(chloro)stannylene ruthenium complex 3.847, generated by direct addition of the stannylene ligand to the N_2_ complex [Cp*Ru(^i^Pr_2_MeP)(H)(N_2_)], in loss of gaseous N_2_ (Scheme 208).^369^ Here, it was found that the hydrido-stannylene complex cannot be isolated, given the significantly greater stability of the metallostannylene tautomer. A similar case was later observed for iron, where the (aryl)(chloro)stannylene complex 3.848 could be isolated, but the hydrido derivative also readily rearranges to the metallostannylene form (*vide infra*).^370^ Oxidation of this species led to H_2_ elimination, which undergoes ligand C–H activation when re-reduced in the absence H_2_, forming novel chelating (alkyl)(aryl)stannylene complex 3.849.

**Scheme 209:**
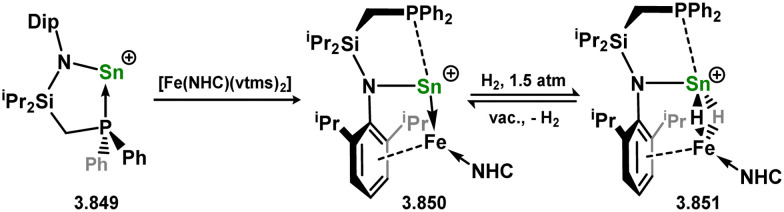
Formation of a cationic stannylene iron(0) complex, and its reversible H_2_ activation.

More recently, our group recently reported a cationic stannylene iron(0) complex and its revesible H_2_ activation, featuring our developed chealting phosphine functionalised amido scaffold (Scheme 209). This complex was accessed through the addition of phosphine-stabilised cationic stannylene 3.850 to the iron(0) complex [IPr·Fe·{η^2^-(vtms)}_2_], leading to loss of the two alkene ligands as per the earlier described germanium system and forming 3.851.^299^ This complex demonstrates the reversible activation of H_2_ across the Sn–Fe bond, leading to bridged hydride complex 3.852, under 1.5 atm of H_2_ at room temperature. 3.850 can also be reduced using a further equivalent of the Fe^0^ precursor, leading to the first example of a covalently bound iron(−i) compound (*vide infra*).

**Scheme 210:**
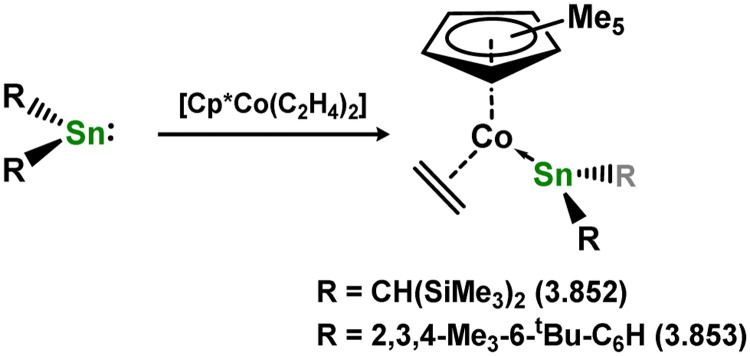
Formation of a cobalt(i) complex bearing an acyclic stannylene ligand.

**Scheme 211:**
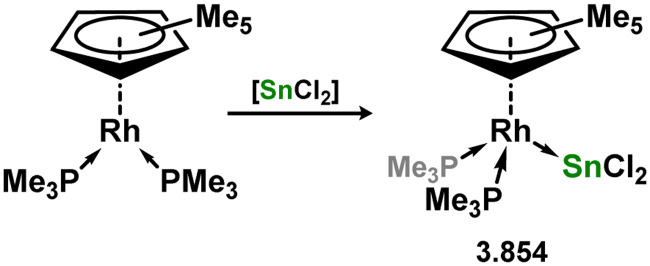
Dichloro stannylene as a Z-type ligand in the coordination sphere of rhodium.

**Scheme 212:**
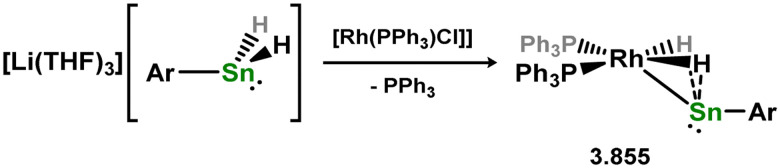
A dihydrido-metallostannylene species, which may be described as having a degree of (aryl)(hydrido)stannylene character.

**Scheme 213:**
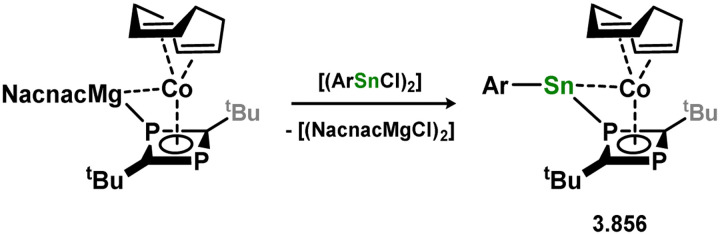
Formation of a cobalt complex bearing a diphosphacyclobutadiene-appended acyclic stannylene ligand.

Similar to toluene-bound iron complexes 3.832 and 3.833, Schneider and co-workers demonstrated that reaction of [CpCo(η^2^-C_2_H_4_)_2_] with bis(alkyl) and bis(aryl) stannylenes leads to mono-substitution of ethylene at ambient temperature, forming complexes 3.853 and 3.854 (Scheme 210).^371,372^ Heating the bis(aryl)stannylene complex 3.853 under mass spectrometric conditions, above 75 °C, leads to ethylene elimination in generating dimeric [(Cp*CoSnR_2_)_2_] (R = CH(SiMe_3_)_2_), though this was only observed in the mass spectrum. Some years later, Campos and co-workers reported the [SnCl_2_] adduct 3.855, in demonstrating the broad Lewis basic properties of the [Cp*Rh(PMe_3_)_2_] (Scheme 211).^302^ Wesemann and co-workers also described the reaction of the stannyl lithium species 3.814 with Rh^I^ chloride [(Ph_3_P)_3_RhCl], leading to double H-migration to Rh, in forming hydride-bridged metallostannylene 3.856, which can also be described as having some degree of (aryl)(hydrido)stannylene character (Scheme 212).^373^ Similar to the germanium(ii) system previously described, the (aryl)(diphosphabutadienyl)stannylene complex 3.857 can be accessed by reaction of the magnesium colbaltate complex with the corresponding chloro-stannylene (Scheme 213).

**Scheme 214:**
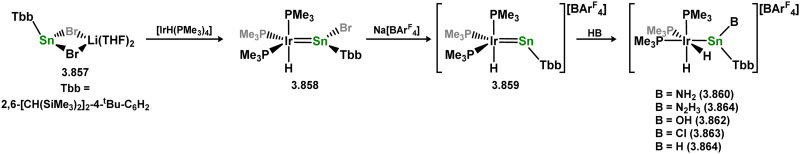
Direct access to an iridium complexes bearing an acyclic stannylene ligand, and its subsequent cooperative activation of protic substrates across the Ir–Sn bond.

Numerous acyclic stannylene complexes of iridium have been reported by Wesemann and co-workers, initially accessed through the straight-forward addition of (aryl)(bromo)stannylene 3.858 to [HIr(PMe_3_)_4_], forming 3.859 (Scheme 214).^301^ Again akin to the earlier described Ge system, bromide abstraction from this species with Na[BAr^F^_4_] led to metallotetrylene complex 3.860, which is capable of the activation of numerous protic nucleophiles across the Sn–Ir bond, the tin centre behaving as a Lewis acidic binding site in these activation reactions, so forming amido- (3.861 and 3.862), hydroxy- (3.863), chloro- (3.864), and hydrido-stannylene (3.865) complexes. This is a promising observation in regards to cooperative bond activation involving the heavier group 14 elements, which utilises the electrophilic nature of the group 14 element centre.

**Scheme 215:**
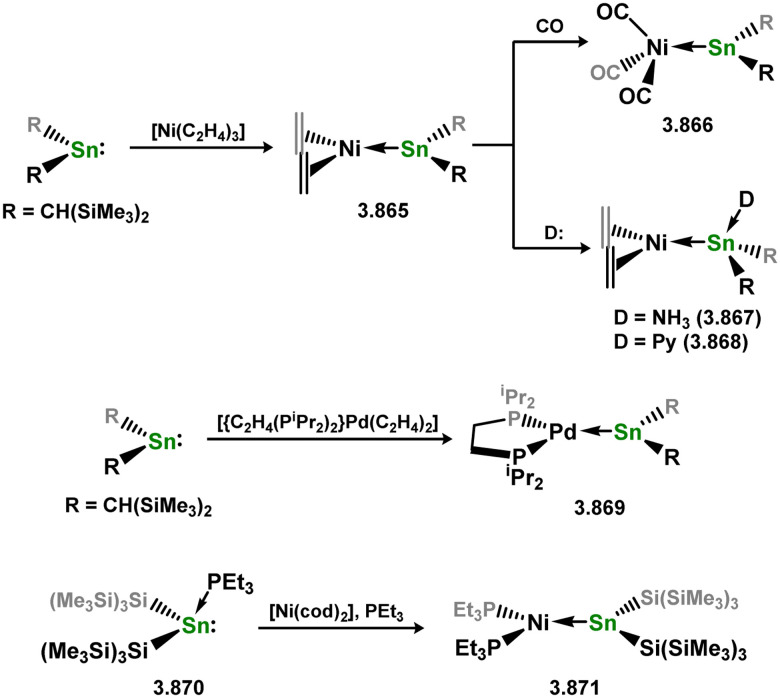
Direct access to a range of acyclic stannylene complexes of group 10 metals.

As already mentioned, Lappert and co-workers described as early as 1974 the synthesis of acyclic stannylene group 10 metal complexes. It was later shown that the addition of Lappert's bis(alkyl)stannylene 3.795 to [Ni(η^2^-C_2_H_4_)_3_] led to mono-ethylene substitution in the formation of 3.866, which remains a rather unique complex, featuring only ethylene ligands aside from the single stannylene (Scheme 215).^374^ Addition of CO to this complex leads to ethylene exchange, forming [Ni(CO)_3_] complex 3.867, whilst the addition of N-donors indicated selective binding at Sn, forming ammonia- and pyridine-adducts 3.868 and 3.869. The palladium complex 3.870, accessed through the addition of the same stannylene to palladium-ethylene complex [{(^i^Pr_2_P)_2_C_2_H_2_}Pd(C_2_H_4_)_2_], was shown to be an active catalyst for the synthesis of stannoles through [1+2+2] cycloaddition of acetylene with 3.795, presumably *via* sequential [2+2] cycloaddtion with the Pd–Sn bond.^375^ The bulky bis(silyl)stannylene 3.871 was shown to readily form a complex with Ni^0^ on reaction with [Ni(cod)_2_], generating complex 3.872, whilst the related reaction with Pd led rather to tautomerisation, in formation of a silastannene complex (*vide infra*).^376^

**Scheme 216:**
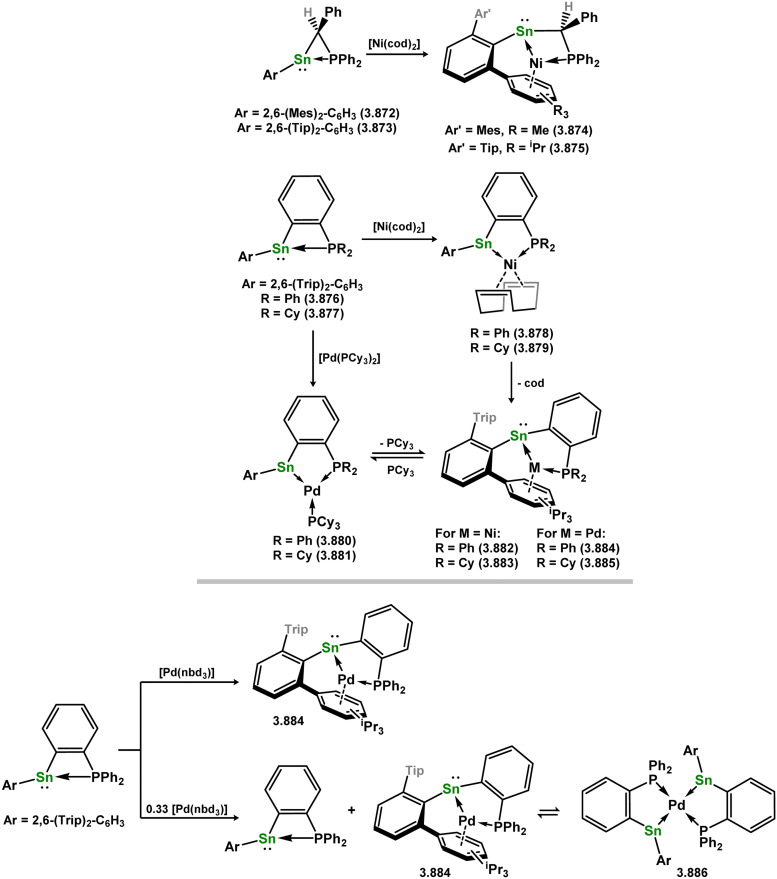
Formation of group 10 complexes bearing phosphine-appended acyclic stannylene ligands, leading to metal chelation and Z-type stannylene behaviour.

Wesemann and co-workers have reported on numerous complexes involving their phosphine-appended aryl-stannylene systems, which behave as chelating ligands towards a TM centre (Scheme 216). First, the direct addition of P-chelated stannylenes 3.873 and 3.874 to [Ni(cod)_2_] led to complexes 3.875 and 3.876, in which the Ni^0^ centre is stabilised by arene- and phosphine-coordination, whilst simultaneously acting as a Lewis-base towards Sn^II^. In the same submission, stannylenes 3.877 and 3.878 were shown to react with both [Ni(cod)_2_] and [Pd(PCy_3_)_2_] in the formation of [Ni(cod)] (3.879 and 3.880) and [Pd(PCy_3_)] (3.881 and 3.882) complexes, respectively.^377^ The initially formed [Ni(cod)] complexes eliminate their second cod ligand over time, forming an arene interaction with one ligand in forming 3.883 and 3.884, as observed in *e.g.*3.874. Similarly, the Pd complexes exists in equilibrium with the analogous species in solution (*viz.*3.885 and 3.886), through phosphine exchange. It was later shown that phosphine-free complex 3.884 can be selectively accessed using [Pd(nbe)_3_] in place of [Pd(PCy_3_)_2_], and that the bis(stannylene) complex 3.887 can also be accessed, despite the significant steric bulk of the aryl ligands in this systems.^378^

**Scheme 217:**
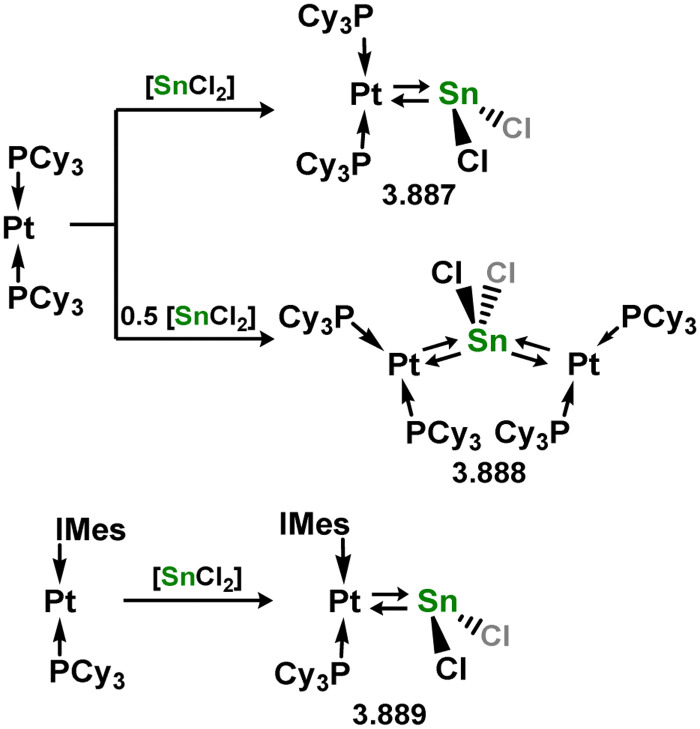
Double-σ-bonding in the dichlorostannylene complexes of platinum(0).

**Scheme 218:**
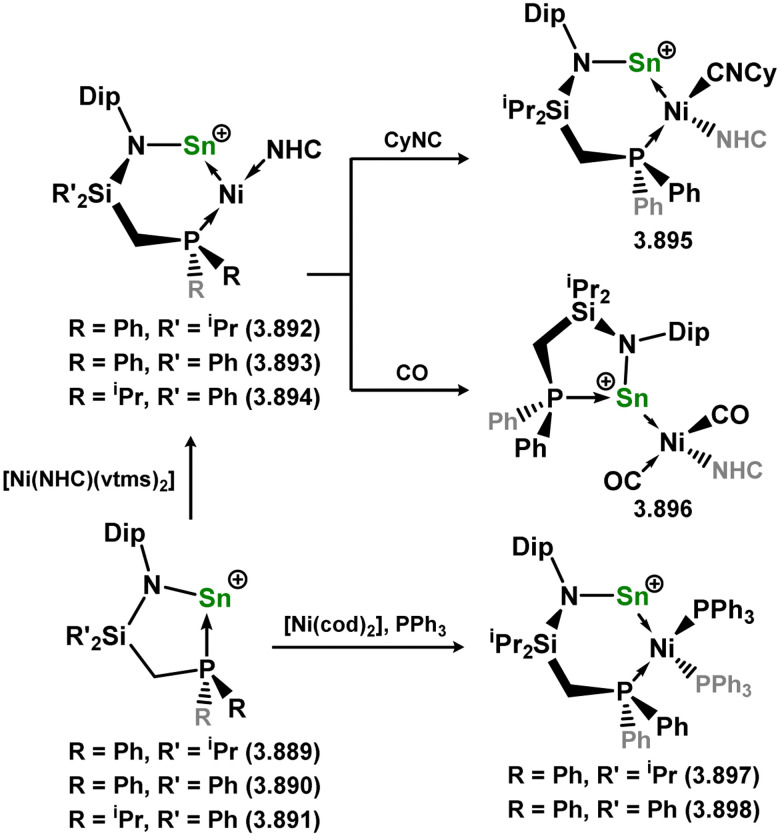
Formation of a geometrically constrained nickel(0) complexes bearing cationic stannylene ligands, leading to Z-type stannylene behaviour, and T-shaped geometries at nickel.

Braunschweig and co-workers demonstrated the Lewis basicity of [Pt(PCy_3_)_2_] through the addition of [SnCl_2_] to this species, analogous to earlier reactions described for the germanium congeners, in complexes 3.888, 3.889, and 3.890 (Scheme 217).^309,379^ Again, the Pt–Sn bonding here is described as ‘double-Sigma’ bonding, through simultaneous σ-donation from the Pt → Sn and Sn → Pt. In our own work, we have also demonstrated the Lewis basicity of the group 10 elements, extending this to Ni. As described earlier for Ge, the cationic stannylenes 3.849, 3.891, and 3.892 can be utilised in accessing T-shaped Ni complexes 3.893, 3.894, and 3.895, in which the stannylene behaves as a Z-type ligand (Scheme 218).^315^ These are the first such examples for nickel bearing stannylene ligands, and are active in the hydrogenation of unactivated alkenes, under relatively mild conditions. These species bind π-acceptor ligands at Ni (*viz.*3.896 and 3.897). Moving to such higher coordinate nickel systems, demonstrated in PPh_3_ complexes 3.898 and 3.899, shuts down the capacity for hydrogenation catalysis, but still allows for alkene hydrosilylation.^316^

**Scheme 219:**
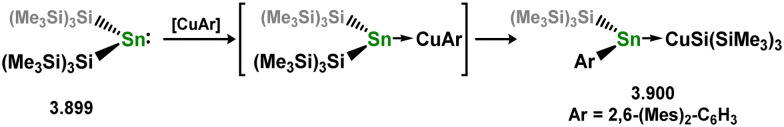
Ligand exchange processes observed in a copper(i) complex bearing an acyclic stannylene ligand.

One example of an acyclic stannylene compound of the group 11 metals has been reported, accessed through the addition of bis(silyl)stannylene 3.900 to [CuAr*], leading to 3.901 (Scheme 219; Ar* = 2,6-Mes_2_-C_6_H_3_; Mes = 2,4,6-Me_3_C_6_H_2_). Rather than the expected bis(silyl)stannylene complex, Ar-SiR_3_ group exchange occurs, so forming the observed product.^380^

No such complexes are known for the group 12 metals.

#### 3.3.4. Chelating bis(stannylene) systems

**Scheme 220:**
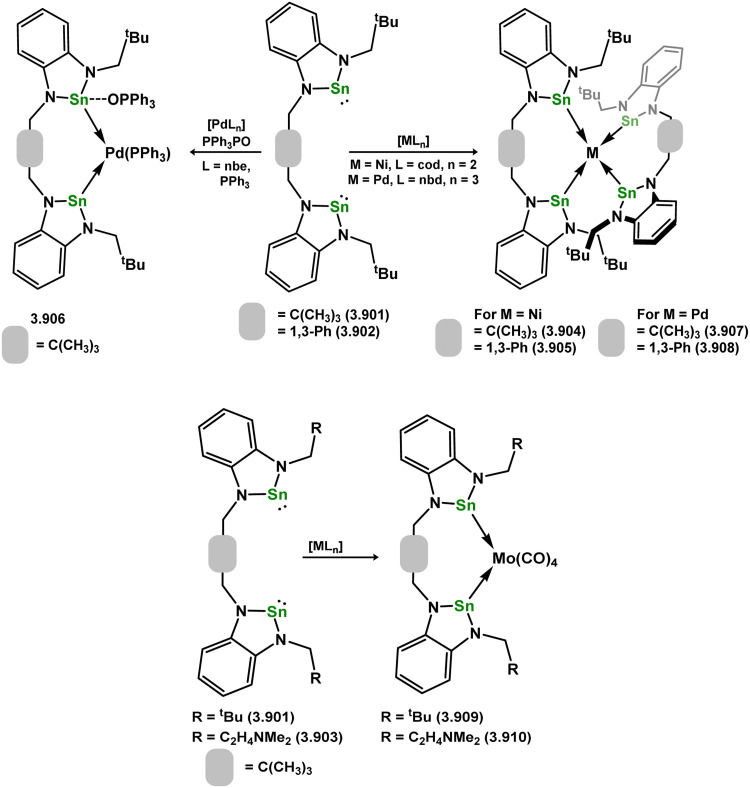
Reactivity of chelating bis(NHSn) ligands towards nickel, palladium, and molybdenum.

As per bis(germylene) ligands, chelating bis(stannylenes) have seen significantly less interest than their silicon counterparts, but have nevertheless led to some interesting species. The initial examples were reported by Hahn and co-workers, namely alkyl-bridged bis(NHSns) 3.902, 3.903, and 3.904, with bis(stannylene) 3.903 featuring and additional amino coordination arm (Scheme 220).^381–383^ These ligands were utilised in generating Ni (3.905 and 3.906), Pd (3.907, 3.908 and 3.909), and Mo (3.910 and 3.911) complexes, through simple combination with suitable metal(0) synthons. Importantly, it was found that the Sn^II^ centres in the complexed ligands are considerably more Lewis acidic than the Ge^II^ counterparts, with THF coordination observed at Sn in *e.g.*3.907, and Ph_3_PO coordination observed in 3.906. This gives a potential platform for the design of ligand non-innocence in stannylene ligands.

**Scheme 221:**
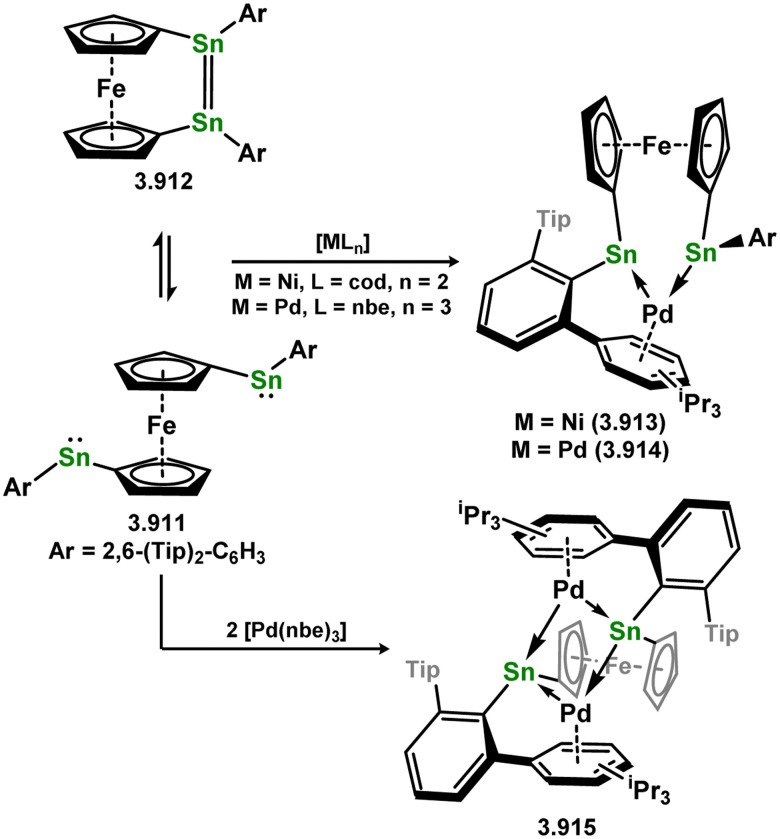
Fluctional behaviour of a bis(stannylene) ligand, and its coordination to palladium.

Considerably later, it was demonstrated by Wesemann and co-workers that bis(stannylene) ligands featuring two-coordinate Sn^II^ centres could be accessed, in the ferrocene-bridged ligand 3.912. This species, presumably related to the conformational freedom of the ferrocene unit, in fact forms an equilibrium with the Sn–Sn bonded distannene 3.913, favouring this species at low temperature.^384^ This isomer can be further favoured by ligand backbone modulation, less flexible xanthene or naphthalene preventing the bis(stannylene) form.^385–387^ Ferrocene-based bis(stannylene) 3.911 was shown to react with Ni^0^ and Pd^0^ synthons in forming chelating complexes 3.914 and 3.915 (Scheme 221), which are essentially isostructural. Here, all alkene ligands at the TM are displaced, in favour of an arene interaction with the ligand. Reaction of ligand 3.911 with two equiv. of the Pd^0^ synthon led to bis(palladium) complex 3.916, with the Sn^II^ centre bridging the two transition metals.

### 3.4. Plumbylene – transition metal chemistry

The low-valent organyl-lead chemistry of the TMs has arguably been the least explored. This is likely attributable to challenges associated with lead chemistry (*e.g.* ready access to radical chemistry, formation of elemental lead), and indeed the toxicity of lead and its compounds. This latter point has particularly influenced studies regarding the reactivity of lead complexes, which would typically find limited applicability. It follows that, of the tetrylenes, plumylene–TM complexes are the least common, with only a handful of examples known. Notably, though many (amidinato)tetrylene–TM complexes are known for Si–Sn, none are reported for lead. Indeed, only a small number of these ligands are known,^388,389^ again highlighting both challenges and applicability of this chemistry.

**Scheme 222:**
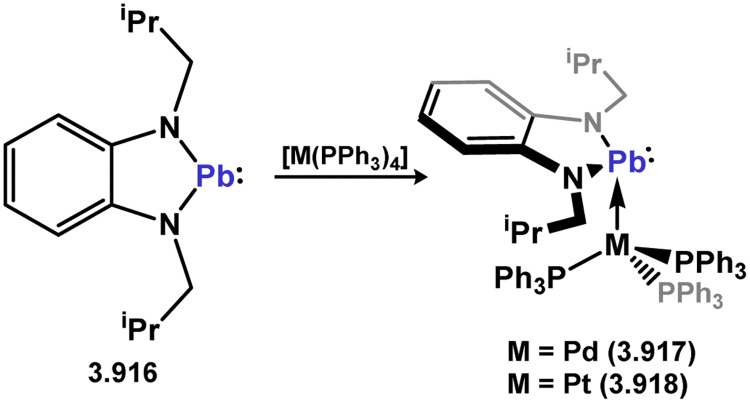
The Z-type behaviour of an NHPb ligand in the coordination sphere of palladium(0) and platinum(0).

The first formal plumbylene complexes of a TMs were accessed through addition of N-heterocyclic plumbylene (NHPb) 3.917 to [M(Ph_3_P)_4_] (M = Pd, Pt), in exchange of one Ph_3_P ligand on forming 3.918 and 3.919 (Scheme 222).^53^ A peculiar bonding situation was observed in these complexes, borne out by a ‘bent’ tetrylene binding angle. That is, the [NNPb] planes are bent by 125.5° (3.917) and 124.5° (3.918) out of the Pb–M bonding plane. This is due to a reversal in the bonding situation between Pb and M, the Pb^II^ centre now behaving as a Lewis acid as opposed to, as perhaps expected, a Lewis base, likely due to the low energy of the lone pair of electrons at this centre, in addition to the electron rich d^10^ M centres.

**Scheme 223:**
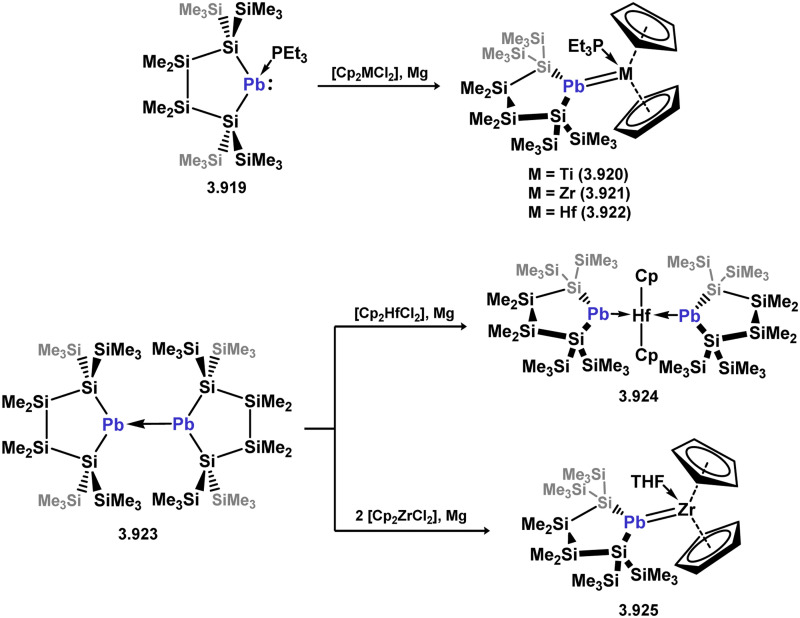
Access to group 4 complexes bearing cyclic bis(silyl) plumbylene ligands.

Later, a ‘classical’ tetrylene bonding of plumbylenes was demonstrated by the group of Müller, in combining cyclic bis(silyl) plumbylene 3.920 with [Cp_2_M] (M = Ti, Zr, Hf; generated *in situ* from [Cp_2_MCl_2_] and Mg), leading to complexes 3.921, 3.922, and 3.923 (Scheme 223).^347^ Here, the silyl ligands presumably raise the energy of the lone-electron pair at Pb, which, in combination with the electron deficient group 4 metals, leads to essentially trigonal planar geometries at Pb (sum of angles = 359.6° for 3.920 and 3.921; = 359.7° for 3.922). Utilising diplumbene 3.924 in place of the phosphine-adduct 3.919, the hafnium-bis(plumbylene) complex 3.925 could also be accessed through a similar protocol, which rather led to the THF-adduct of mono-plumbylene zirconocene (*viz.*3.926) when using [Cp_2_ZrCl_2_].

**Scheme 224:**
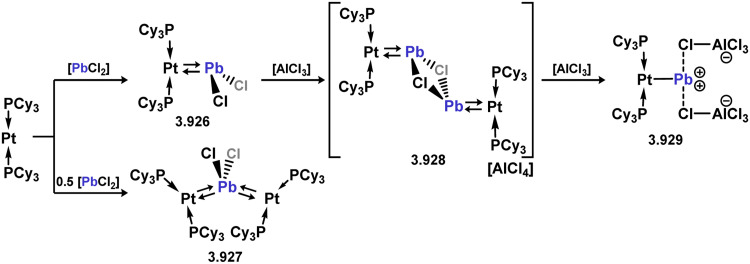
Double-σ-bonding in chloro plumbylene complexes of platinum.

**Scheme 225:**
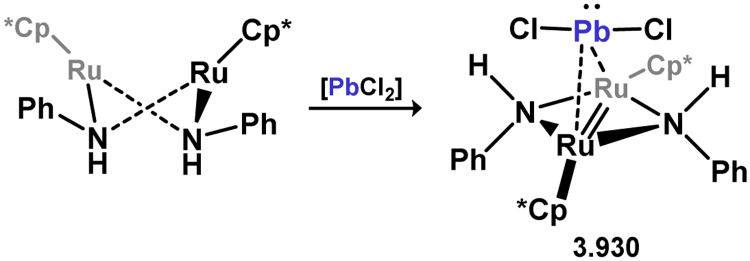
Accessing a dichloro plumbylene complex, with a seesaw geometry at lead.

Soon after this report, Braunschweig and co-workers reported further examples of ‘bent’ plumbylene complexes of d^10^ platinum(0) in [(Cy_3_P)_2_Pt], through reaction with [PbCl_2_], so forming monoplatinum complex 3.927, or bis-platinum complex 3.928, depending on stoichiometry (Scheme 224).^390^ The former complex features a significant bending angle of 110.93°, more acute than in the above described NHPb complex. As per earlier described Ge and Sn congeners, these complexes are described as featuring σ-donor-σ-acceptor bonding between Pb and Pt, as opposed to the classical case for Fischer-type bonding, with σ-donation and π-backdonation.^309^ It was later shown that chloride abstraction from 3.926 with [AlCl_3_] readily formed the related cationic plumbylene complex, which dimerises forming 3.929.^379^ This dimer is cleaved through the addition of DMAP, and undergoes I/Cl exchange when reacted with NaI. More interesting, however, is the reaction with a further equivalent of [AlCl_3_], leading to complex 3.930 with a near linear Cl_3_AlCl⋯Pb⋯ClAlCl_3_ angle of 177.90°, though no electronic explanation for this was discussed. Notably, a similar Cl–Pb–Cl geometry was observed in the later reported [PbCl_2_] complex of a ruthenium dimer, namely 3.931 (Scheme 225).^391^ The Pb centre in this species sits in a seesaw geometry, due to maintaining a stereochemically active lone pair of electrons whilst simultaneously receiving electron density from the two Ru centres. Further PbCl⋯HN interactions are thought to further linearise the Cl–Pb–Cl moiety.

**Scheme 226:**
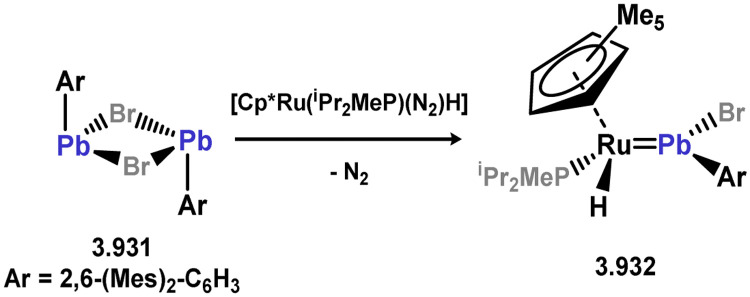
Direct access to an acyclic plumbylene complex of ruthenium.

**Scheme 227:**
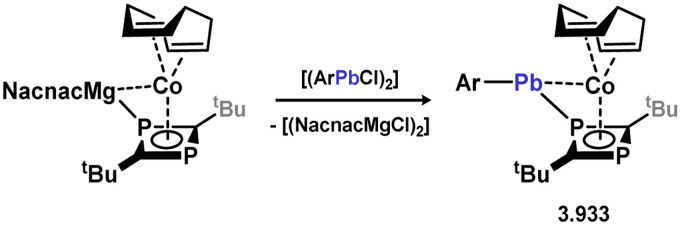
Formation of a cobalt complex bearing a diphosphacyclobutadiene-appended acyclic plumbylene ligand.

Tilley and co-workers have demonstrated that the bulky (aryl)(bromo)plumbylene 3.932 reacts with [Cp*Ru(N_2_)(^i^Pr_2_PMe)H] in N_2_ displacement, forming plumbylene complex 3.933, as part of a broader study into metallotetrylenes (Scheme 226).^369^ It was later shown that reaction of the closely related (aryl)(chloro)plumbylene with a cobaltate complex led to the formation of 3.934, which, as per earlier described Ge and Sn analogues, can be described as a cobalt–plumbylene complex (Scheme 227).^392^

There are no known examples of transition metal complexes bearing chelating bis(plumbylene) ligands.

### 3.5 Appraisal of the field of heavier tetrylene – transition metal chemistry

As is apparent from the above review of tetrylene complexation chemistry, this field has remained fervent since the first examples of heavier tetrylene complexes were reported by Lappert.^50^ Given the ease of access to Ge^II^ systems, *via* the commercially available dioxane adduct of [GeCl_2_], more than 300 complexes are known for monodentate germylene ligands. Perhaps given the abundance of silicon, silylene chemistry has been equally as lively, despite the lack or readily available Si^II^ synthons. Here different approaches are utilised: isolable silylene ligands are somewhat rare, but the coordination chemistry of those stable examples has been explored greatly. In addition, due to the importance of hydrosilylation catalysis, significant effort has gone towards formal silane dehydrogenation in forming metal silylene complexes. Importantly, given the prominence and ease of synthesis of the so-called Roesky silylene, a vast number of chelating bis(silylenes) are known which employ this strongly σ-donating moiety. As per germanium, easy access to Sn^II^ synthons has also led to significant progress in stannylene complexation, whilst related Pb^II^ chemistry remains very rare indeed.

The vast array of complexes discussed here gives us a clear understanding of the effects of periodicity and ligand design on the chemistry of tetrylene – transition metal complexes. The π-character of the E–M bond (E = Si–Pb, M = transition metal) is expected to be greater for the lighter group 14 elements,^20^ though this has not been demonstrated across a continuous, analogous series. The HOMO–LUMO gap in silylene complexes is also expected to be narrower than for heavier derivatives for similar systems: this can be seen to some degree in cationic silylene – nickel complex 3.291 reported by Kato and co-workers,^184^ in comparison to related cationic germylene (3.682–3.684) and stannylene (3.892–3.894)^315^ species reported by our group: the former rapidly cleaves dihydrogen and undergoes cycloaddition with unsaturated C–C bond, whilst our systems do not. This is an important factor in designing tetrylenes as ligands for catalytic processes: a stoichiometric reaction product should not be too stable so as to generate an energetic sink. As such, the cationic germylene and stannylene complexes mentioned above can in fact catalyse alkene hydrogenation, despite not demonstrating stoichiometric activation of either substrate on the NMR time scale. An increased electron deficiency at the tetryl centre also increases π-character, as shown, for example, by Holl and co-workers in moving from bis(amido)germylene (*d*_GeNi_ = 2.217 Å) to bis(fluoroaryl)germylene (*d*_GeNi_ = 2.182 Å)-nickel complexes 3.669 and 3.671, respectively.^306,307^ This electron deficient character also narrows the HOMO–LUMO gap, which may be described as a π–π* transition, and can thus lower the energetic barrier to bond activation.^393^ These attributes regard two-coordinate tetrylene ligands; given that formal π-bonding arises from simultaneous E → M σ-donation and M → E back-donation to a vacant p-orbital on E, base-stabilised tetrylene ligands do not infer E–M π-bonding interactions. This is somewhat borne out by comparison of the average Si–Ni bond lengths in two-coordinate silylene – nickel complexes, and formally base-stabilised (amidinato)silylene – nickel complexes, by a survey of the CCDC: the former are on average ∼0.05 Å shorter, which may be attributed to an increased Ni → Si back bonding. In addition to the described periodicity in bonding characteristics for the heavier tetrylenes, the more favoured tetravalent state for the lighter group 14 elements *vs.* divalent for the heavier elements has a strong effect on the energetics of a reaction: a number of a highly reactive silylene – transition metal complexes are known which readily activate small molecules irreversibly,^49,174,180,182,183^ whilst a greater number of reversible bond activation processes are known in, for example, germylene complexes (*e.g.* of CO_2_ and H_2_).^47,317,393^

All of these points are nuanced. There are essentially infinite combinations of supporting ligand framework, tetryl element, and transition metal, leading to an equally infinite range of E–M bonding energies within the complex classes described. The current state of the field works towards garnering a greater understanding of how these differing combinations may affect reactivity of these systems, and ultimately towards their utility as both spectator and non-innocent ligands in transition metal catalysed processes.

## 4. Heavier tetrylidyne/metallotetrylene systems

**Fig. 9:**
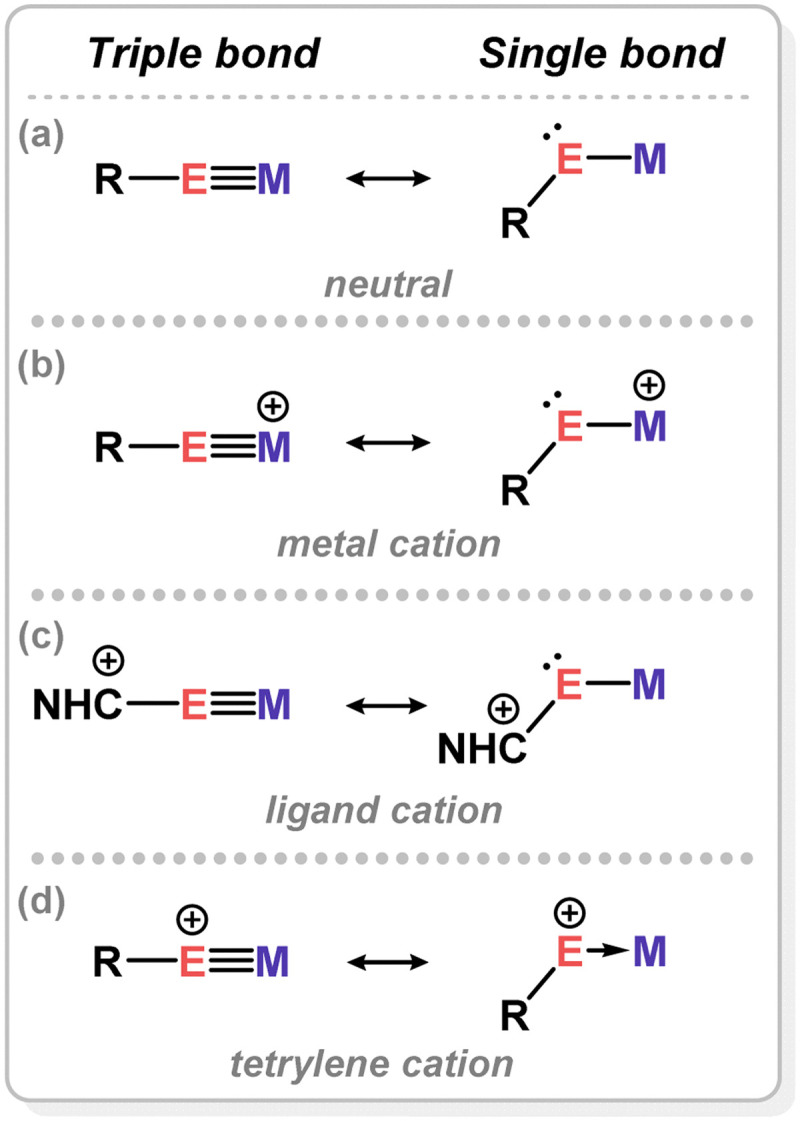
Resonance forms and bonding in difference classes of tetrylidyne/metallotetrylene complexes.

The chemistry of alkylidyne/carbyne complexes has garnered significant attention both from a fundamental perspective and in its applications within metathesis chemistry.^394,395^ In recent years, there has also been an exploration of related heavier group 14 chemistry, although it presents greater challenges due to the diminished inclination of heavier main group elements to engage in stable multiple bonding interactions.^396^ This leads to two key tautomeric forms: the so-described triply bonded tetrylidyne species, and singly bonded metallotetrylene species (Fig. 9); the latter is essentially non-existent for carbon species. These can be split again into formally covalently bound E–M species (*viz.* (a)–(c); Fig. 9), and exclusively datively bound species (*viz.* (d); Fig. 9), which we have shown in our work to feature unique dative triple bonds. In all cases, these tautomers have distinct geometrical differences, the former being near linear, and the latter being bent due to the presence of a stereo-active lone-pair of electrons at the tetryl centre. This chemistry has been thoroughly explored for all heavier tetryl elements, giving a distinct picture of factors effecting observed resonance forms, complex stability, and indeed their further reactivity.

**Fig. 10:**
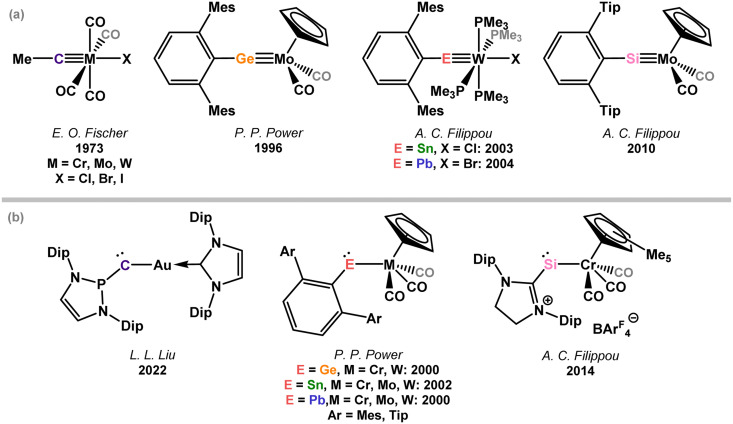
The historical precedent for (a) the tetrylidyne and (b) metallotetrylene analogogues for C-Pb.

Historically, carbyne complexes were established as an isolable compound class before the heavier tetrylidyne derivatives, by E. O. Fischer and co-workers in developing the further chemistry of their closely related carbene systems (Fig. 10(a)).^397^ It wasn’t until over 30 years later that the first example of a triply-bonded heavier tetrylidyne was reported, being a Ge derivative discovered by the group of Power, in which significant kinetic stabilisation was required in the form of a bulky terphenyl ligand at the Ge centre.^398^ This led to the isolation of related Sn, Pb, and Si derivatives from the group of Filippou in the years 2003,^399^ 2004,^400^ and 2010,^154^ respectively, all of which utilised (i) bulky terphenyl ligands at the tetrel centre, and (ii) the group 6 metals, which are known for their ability to readily form multiple bonds. These species laid the foundation for what remains a fervent area of research, in exploring the chemistry of multiply-bonding group 14-TM complexes. In contrast to the early discovery of carbyne complexes, the first example of a structurally characterised metallocarbene was only discovered very recently by Liu and co-workers (Fig. 10(b)),^401^ over 20 years after the first heavier derivative, which centred on germanium and was reported by Power and co-workers in 2000.^402^ That initial report featuring a metallogermylene was rapidly followed by Sn and Pb derivatives reported by the same group,^403,404^ with the first Si derivative forthcoming some years later from the group of Filippou.^405^ That these heavier derivatives were discovered significantly earlier than that for carbon aligns with the notion that multiple-bonding is less favoured for the heavier p-block elements, given their greater HOMO–LUMO separation, and thus a lessened propensity for their s-character lone electron pair to partake in bonding.^406^ Factors which lead a complex to form the tetrylidyne or metallotetrylene resonance forms are now well understood; synthetic methods for accessing these species, and their further chemsitry, has now been explored to a significant degree. The following part of this review aims to summarise these findings. Complexes here will be described sequentially, moving from silicon to lead, from early to late TMs, with key reactivity, electronic characteristics, and metrical parameters described in exceptional cases.

### 4.1. Silylidyne/metallosilylene systems

**Scheme 228:**
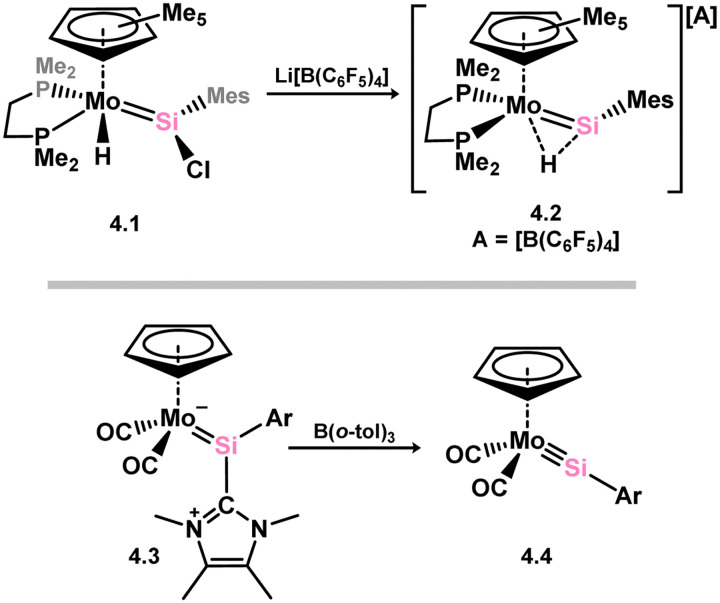
The first reported example of a silylidyne transition metal complex.

The initial report of a complex bearing substantial silicon–TM triple bond character, from Tilley and co-workers, was accessed *via* chloride abstraction from the (aryl)(chloro)silylene-molybdenum hydride complex 4.1, in the formation of 4.2.^148^ In the molecular structure of this cationic silylidyne complex, it was observed that the molybdenum hydride ligand bridges the silicon and molybdenum centers, likely causing some perturbation of the triple bond character between these two centres (Scheme 91). Subsequently, a hydride-free, neutral molybdenum silylidyne complex was documented, through carbene-abstraction from silylene complex 4.3 (*viz.*4.4, Scheme 228).^154^ It is noteworthy that this latter complex, lacking a Mo–H fragment, exhibits a longer Si–Mo bond compared to the hydride-bridged 4.2. This elongation may be attributed to the charged nature of this complex, leading to a contraction of the highly polarized Si–Mo bond.

**Scheme 229:**
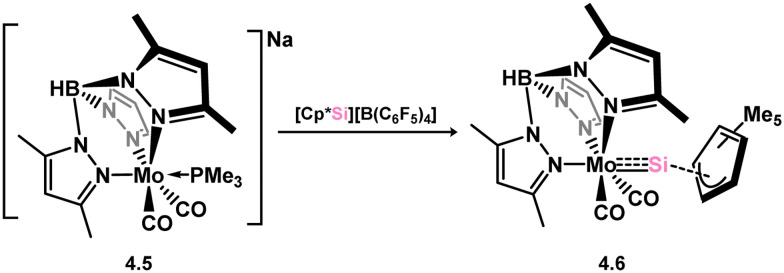
A molybdenum silylidyne complex, bearing the Cp* ligand at silicon.

More recently, it was demonstrated that the reaction of the cationic Si^II^ complex, [Cp*Si][B(C_6_F_5_)_4_], with the anionic tris(pyrazolyl)borate molybdenum complex 4.5, led to the Cp*-bound molybdenum silylidyne complex 4.6, in the loss of Na[BAr^F^_4_] (Scheme 229).^407^ Once again, the Si–Mo bond length in this complex surpasses that of the two previously mentioned species, highlighting the strong influence of the cationic charge in complex 4.2 on its Si–Mo bond.

**Scheme 230:**
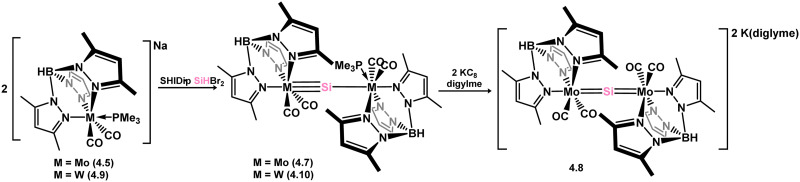
Formation of a metallo–silylidyne complex, and its reduction to a formally silicon(-iv) complex.

A noteworthy illustration of a silicon–metal triple bonded complex emerged in 2019, again reported by Filippou and co-workers, featuring the anionic complex 4.5 as its precursor. In this instance, two equivalents of 4.5 react with the Si^II^ dibromide complex, [SIDip·SiBr_2_] (SIDip = [H_2_CNDip]_2_C:), resulting in the elimination of sodium bromide and SIDip, yielding metalla-silylidyne 4.7 (Scheme 230).^408^ Within this species, the central silicon atom forms a triple bond with one Mo centre and a single bond with the second Mo centre. Moreover, this entity could be reduced with potassium metal, producing the dianionic 1,3-dimetalla-2-silaallene 4.8, wherein a two-coordinate central silicon atom possesses a double bond with each Mo center. Formally, in both complexes 4.7 and 4.8, the central Si atom retains a −4 oxidation state, marking the initial instance of a stable molecular species featuring silicon in this particular oxidation state. Subsequently, it was demonstrated that the corresponding anionic tungsten complex 4.9 is accessible, allowing for the synthesis of tungsten-derived silylidyne complex 4.10, using comparable methodologies.

**Scheme 231:**
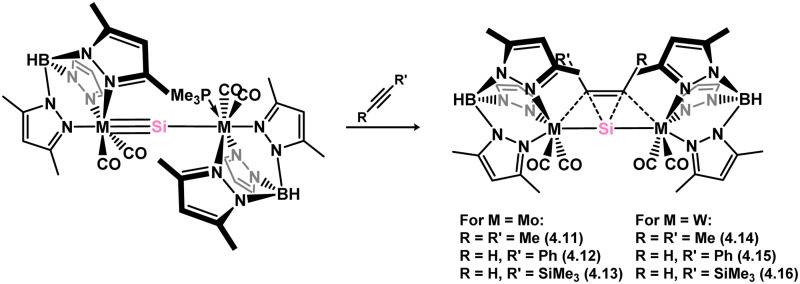
Synthesis of planar 4-coordinate silicon systems through the cycloaddition of alkynes to a metallo–silylidyne complex.

Remarkably, the reaction of both Mo and W complexes with a variety of alkynes resulted in the formation of unprecedented planar tricyclic [M_2_SiC_2_] complexes (Scheme 231; M = Mo (4.11, 4.12, and 4.13), W (4.14, 4.15, and 4.16)).^409^ These are denoted as ptSi (planar tetracoordinate silicon) complexes, which challenge the established principles of Le Bel and van't Hoff, who suggest such a geometry for carbon (referred to as ptC) is highly unstable due to the favourable energy associated with tetrahedral coordination. While the ptC configuration had been achieved in stable complexes several years ago, the described silicon species are the first for the heavier group 14 elements. Since this initial report, a handful of additional examples of ptSi species have emerged, though not featuring a TM centre.^410,411^

**Scheme 232:**
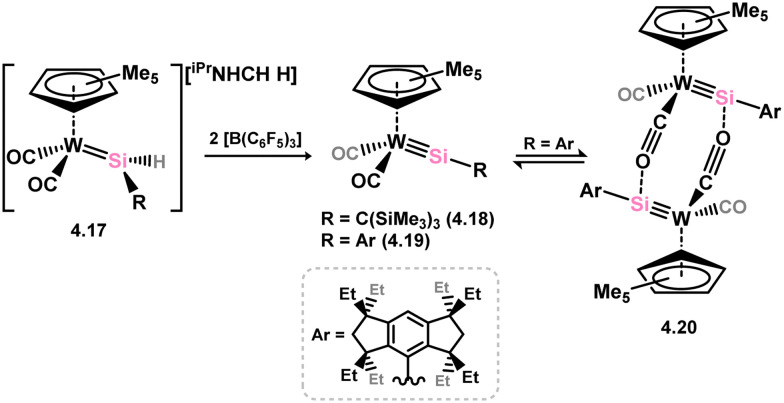
Formation of a tungsten silylidyne complex, and its monomer–dimer equilibrium.

Additional instances of tungsten silylidyne complexes have been documented. A distinct synthetic approach was introduced by Tobita and co-workers, in which stepwise introduction of two equivalents of B(C_6_F_5_)_3_ to the anionic (alkyl)(hydrido)silylene complex 4.17 led to formal H_2_ elimination and carbene abstraction, in forming 4.18, which features the [(Me_3_Si)_3_C] group at Si (Scheme 232).^152^ A similar method was utilised in the synthesis of silylidyne complex 4.19, bearing the bulky Eind aryl group at Si (denoted as Ar in Scheme 232), with significantly improved yields when compared with 4.18. Notably, silylidyne complex 4.19 adopts a CO-bridged dimeric configuration (*viz.*4.20) in the solid state, but forms a monomer–dimer equilibrium in solution. The Si–W triple bond in this species engages in [2+2] cycloaddition reactions with carbodimides and ketones, leading to the formation of metalla-sila-cyclobutene species, which are essentially cyclic silylene complexes and hence are discussed in Section 3.^156^

**Scheme 233:**
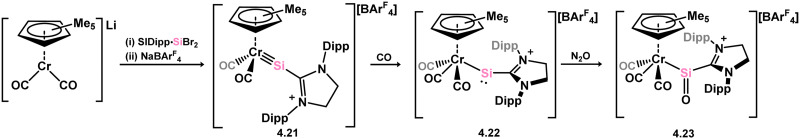
Formation an imidazolium-ligated chromium silylidyne complex, and its oxidation in formation of a metallo–silanone complex.

Only a single silylidyne complex of a first-row TM has been successfully synthesised, *via* addition of [SIDip·SiBr_2_] to Li[Cp*Cr(CO)_2_], followed by bromide abstraction in forming 4.21. Though the Si centre in this species is coordinated by an NHC, this is best described as an imidazolium ligand in this case, the cationic charge in this complex primarily localised on this ligand, making 4.21 formally a silylidyne complex (Scheme 233).^405^ Subsequent reaction with CO results in coordination at Cr and disruption of the triple bond, in forming metallosilylene complex 4.22. The oxidation of this species with N_2_O provided access to the first instance of a silanone functional group, characterised by a Si–O double bond, in complex 4.23.

**Scheme 234:**
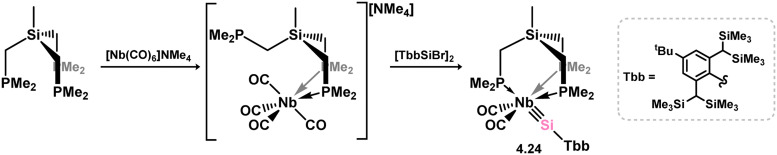
Synthesis of a niobium silylidyne complex.

One documented instance of a niobium silylidyne complex, obtained in a relatively straightforward manner in combining dimeric [{(Br)(Tbb)Si}_2_] with the anionic Nb^−I^ complex [{MeSi(CH_2_PMe_2_)_3_}Nb(CO)_4_]NMe_4_, resulting in the formation of octahedral silylidyne complex 4.24 (Scheme 234).^412^ It is worth noting that the synthesis of several additional silylidyne complexes, such as those involving Fe and Ni, are included in *e.g.* PhD theses. However, as these remain undocumented in peer-reviewed journals, and the relevant data is not readily accessible, they have not been included in this discussion.

**Scheme 235:**
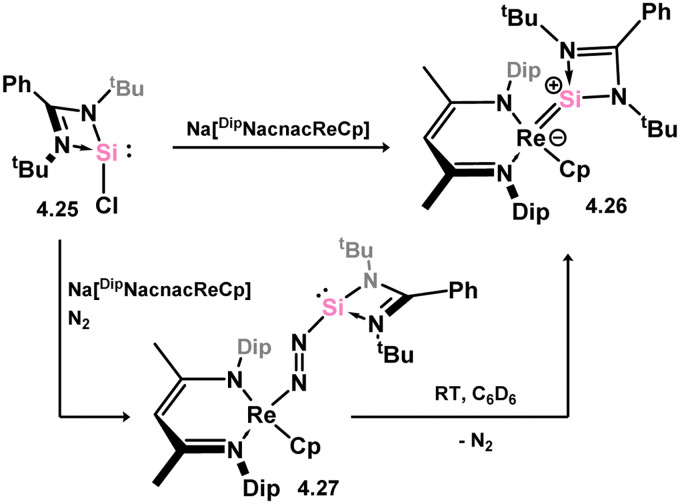
Synthesis of a rhenium metallosilylene complex, and its activation of N_2_.

Finally, one complex of Re which can be described as being intermediate between a metallosilylene and silylidyne has been reported, accessed through salt-metathesis of chloro silylene 4.25 with a Nacnac-bound anionic Re species, in forming 4.26. Interestingly, when carried out under an atmosphere of dinitrogen, the N_2_ complex 4.27 is observed, which slowly eliminates the N_2_ unit in solution at (Scheme 235) ambient temperature.^413^

### 4.2. Germylidyne/metallogermylene systems

There are a significant number of complexes in this class for germanium, the vast majority falling into the category of germylidynes of the heavier group 6 metals, Mo and W. Again this mirrors the utility of these transition metals in the metathesis chemistry of C–C triple bonds.^394,395^

**Scheme 236:**
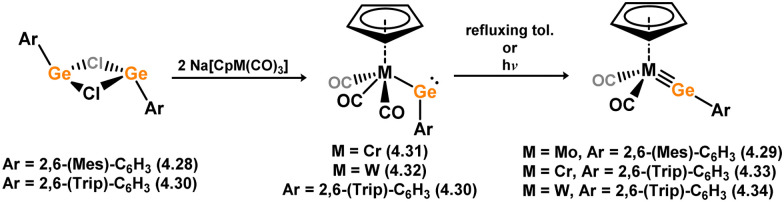
The initial examples of germylidyne complexes, utilising the group 6 metals, formed *via* their respective metallogermylene complexes.

The first example of such a species was reported by Power and co-workers, accessed through the addition of (aryl)(chloro)germylene 4.28 to Na[CpMo(CO)_3_], forming the molybdenum germylyne species 4.29, presumably forming an initial metallogermylene complex, which subsequently loses CO in the formation of a Mo–Ge triple bond (Scheme 236).^398^ This was borne out by the near linear L–Ge–Mo bond angle of 172.2(2)°, and a very short Ge–Mo bond length of 2.271(1) Å, comparing to *e.g.* 2.658 Å in germyl-molybdenum species.^414^ The related reactions for Cr and W were subsequently reported, utilising (aryl)(chloro)germylene 4.30. Here, however, it was found that the initial salt-metathesis reaction led to isolable metallogermylene complexes 4.31 and 4.32, which only formed the triply bonded germylidyne products 4.33 and 4.34 after thermo- or photolytic expulsion of one CO ligand.^402^ The structural characterisation of both tautomers allows for the direct observation of geometric changes at Ge in moving from germylenes 4.31 and 4.32 (∠_LGeM_ = 117.8(2)° and 114.7(6)°, respectively) to germylidynes 4.33 and 4.34 (∠_LGeM_ = 175.99(6)° and 170.9(3)°, respectively), the non-linear angles in the former indicative of stereoactive lone pairs of electrons at Ge.

**Scheme 237:**
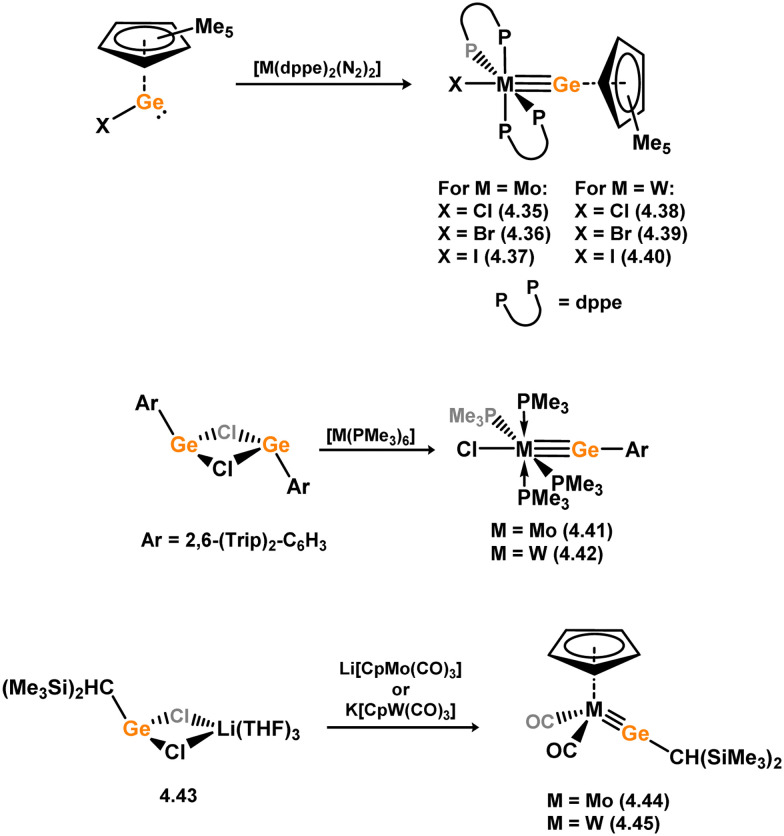
Further examples of group 6 germylidyne complexes.

**Scheme 238:**
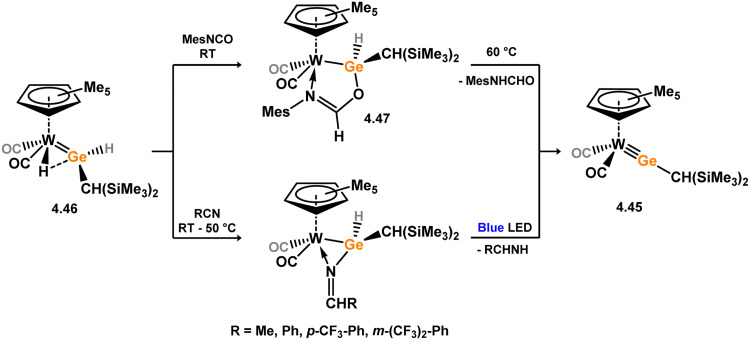
Tungsten–germylidyne complex formation through formation H_2_ abstraction by organic substrates.

Further examples of W and Mo germylyne complexes were later reported by Fillipou and co-workers (Scheme 237), synthesised through the oxidative addition of Cp*GeX (X = Cl, Br, I) to [M(dppe)(N_2_)_2_] (M = W, Mo), directly leading to the triply bonded Ge–Mo (4.35, 4.36, and 4.37) and Ge–W (4.38, 4.39, and 4.40) complexes, where the X and Cp*Ge ligands are *trans* in the octahedral products.^415,416^ DFT calculations here demonstrated that the GeM bonds feature a single σ-bond, and two π-bonds, as per alkylidyne complexes. The same group also demonstrated that related complexes 4.41 and 4.42 could be accessed through the addition of (aryl)(chloro)germylene 4.30 to [M(PMe_3_)_6_] (M = Mo, W), in elimination of 2 equiv. PMe_3_,^289^ and further that the alkyl-germylenoid 4.43 reacts with anionic Mo and W complexes (*i.e.* Li[CpMo(CO)_3_] and K[CpW(CO)_3_]) in forming alkyl-germylidyne complexes 4.44 and 4.45.^291^ Using a different approach, Hashimoto, Tobita, and co-workers showed that the hydridogermylene complex 4.46 reacts with mesityl isocyanate in forming germyl complex 4.47, which eliminates MesN(H)C(H)O in forming the above described germylidyne complex 4.45 (Scheme 238).^417^ A similar synthetic route was later reported employing nitrile substrates, forming imines and the target germylidyne species upon irradiation with blue light.^418^

**Scheme 239:**
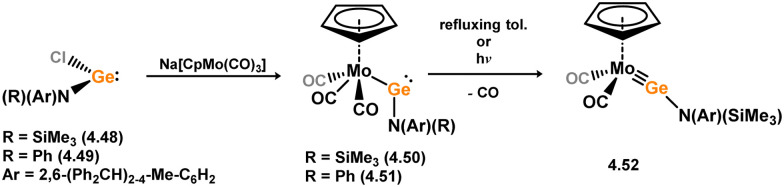
Formation of metallo(amido)germylene and amido–germylidyne complexes of molybdenum.

An amido derivative of such molybdenum germylidyne complexes has also been reported, through the salt-metathesis of a bulky (amido)(chloro)germylene with Na[CpMo(CO)_3_] (Scheme 239).^419^ Here, the (amido)(chloro) germylenes 4.48 and 4.49 lead to the formation of metallogermylene complexes 4.50 and 4.51. Only complex 4.50, which featurues an (aryl)(silyl)amido ligand, was shown to undergo CO elimination upon heating in forming the target germylidyne complex 4.52. In contrast, related metallogermylene 4.51, bearing a bis(aryl)amide ligand, does not form the target germylidyne complex, suggesting that the electronic nature of the supporting monoanionic ligand is important for such transformations.

**Scheme 240:**
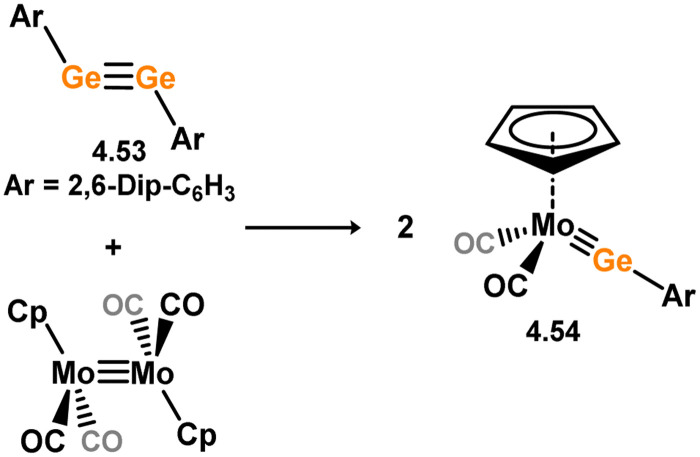
Cross-metathesis of germanium(i) and molybdenum(i) dimers, in the formation of a molybdenum–germylidyne complex.

Developing a further method for accessing molybdenum germylidyne complexes, Power and co-workers showed that the aryl-stabilised digermyne 4.53, featuring a Ge≡Ge triple bond, undergoes metathesis with Mo≡Mo dimer [{CpMo(CO)_2_}_2_] leading to the formation of germylidyne complex 4.54 (Scheme 240).^420^ This is a remarkable mirroring of classic alkyne metathesis chemistry, opening potential new vistas in the synthesis of heteroelemental multiple bonds of the heavier p-block elements.

**Scheme 241:**
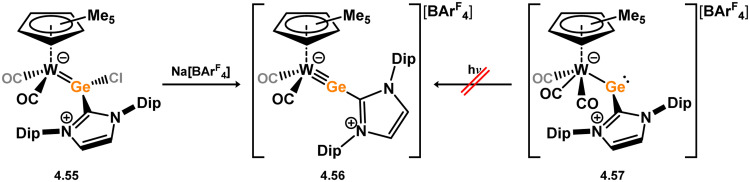
Formation of a cationic tungsten–germylidyne complex supported by an imidazolium ligand, and its related metallogermylene, which does not form the former upon radiation.

The NHC-stabilised chloro–germylene complex 4.55 was also utilised in the generation of a germylidyne complex, through chloride abstraction leading cationic species 4.56, in which one may consider the charge localised at the imidazole ligand (Scheme 241), thus making this species electronically similar to examples described above.^421^ Notably, the related tris-CO complex 4.57 is formally described as metallogermylene, which does not release a CO ligand to form triply bonded 4.56.

**Scheme 242:**
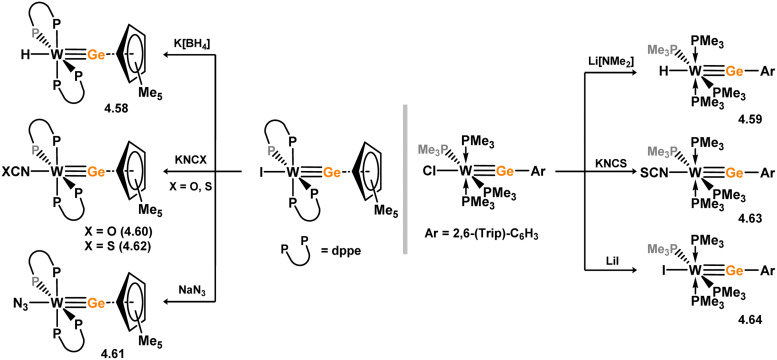
Formation of a range octahedral tungsten–germylidyne complexes through W–X salt-metathesis (X = Cl, I).

**Scheme 243:**
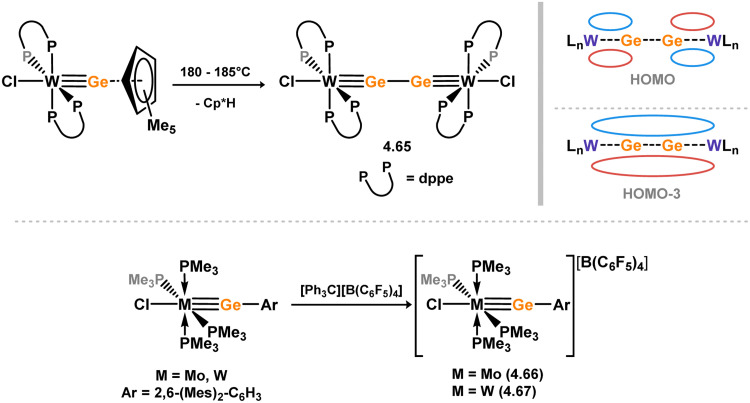
Formal reduction (above) and oxidation (below) of group 6 germylidyne complexes. Inset: Delocalised π-bonding in the [W_2_Ge_2_] unit.

Some interesting reactivity of described group 6 germylidyne complexes has been described. Filippou and co-workers showed that the halide ligands in 4.40 and 4.42 can be readily substituted in salt-metathesis reactions, with [H]^−^ (4.58 and 4.59), [NCO]^−^ (4.60), [N_3_]^−^ (4.61), [NCS]^−^ (4.62 and 4.63), and [I]^−^ (4.64; Scheme 242).^289,422^ These processes all proceed without affecting the Ge≡W bond, leading to only very small changes in this bond length. It was also demonstrated that chloro-substituted complex 4.38 undergoes a reductive Ge–Ge coupling reaction when heated in the solid state, forming an unprecedented [WGeGeW] unit in 4.65 (Scheme 243).^423^ The frontier orbitals of this species revealed a shared π-orbital across the [WGeGeW] core. In a related reaction, attempted group abstraction from Mo and W complexes 4.41 and 4.42 with the [Ph_3_C][BAr_4_^F^] system (Ar^F^ = C_6_F_5_) did not lead to Ge–R or Cl elimination, but rather oxidation, in the formation of the first examples of open-shell germylidyne complexes 4.66 and 4.67.^424^ These systems bare essentially unchanged Ge≡M bond distances, but elongated M–P distances, due to the now partially filled HOMO of these species reducing π-back donation to the PMe_3_ ligands.

**Scheme 244:**
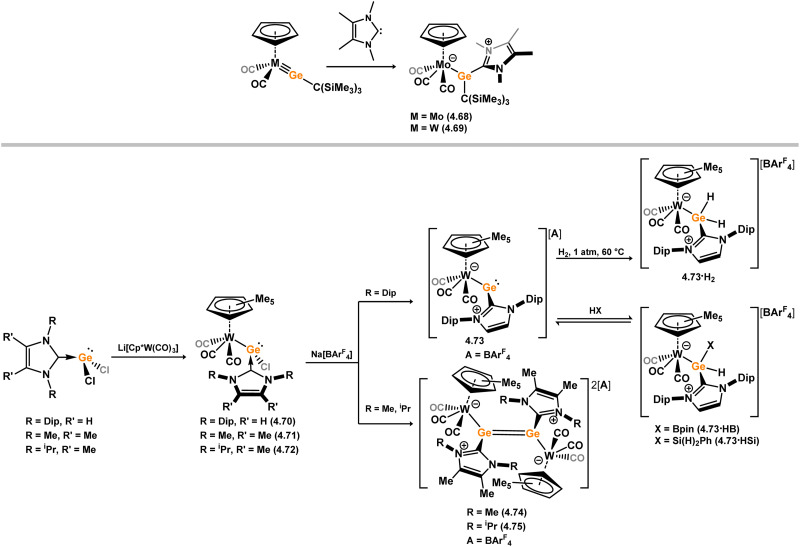
Formation of a cationic imidazolium-stabilised metallogermylene, and its dimeric derivatives.

It has also been shown that the addition of strong NHC donor ligands to germylidyne complexes 4.44 and 4.45 leads to coordination at Ge, leading to conversion to formal germylene complexes 4.68 and 4.69. Tobita and co-workers later showed that related higher coordinate tungsten complexes could be directly accessed through the addition of [NHC → GeCl_2_] adducts to [Li(THF)_2_][Cp*W(CO)_3_], forming 4.70, 4.71, and 4.72 (Scheme 244).^425^ Chloride abstraction from 4.70, featuring a bulky Dip-substituted NHC, led to the monomeric metallogermylene complex 4.73, whilst the same reaction involving less sterically encumbered NHC ligands led to the formation of 1,2-dimetallo-digermenes, 4.74 and 4.75. Notably, metallo-germylene 4.73 undergoes oxidative addition of H_2_, borane, and silane at the Ge centre (Scheme 244), the reaction being reversible in the latter two cases.^426^ This thus represents a rare example of oxidative addition/reductive elimination at group 14 element centre.

**Scheme 245:**
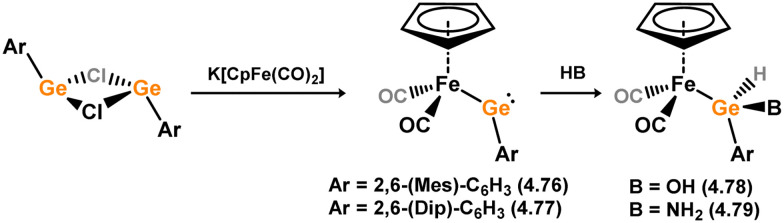
Formation of a ferratogermylene, and oxidative addition chemistry of the germanium(ii) centre.

**Scheme 246:**
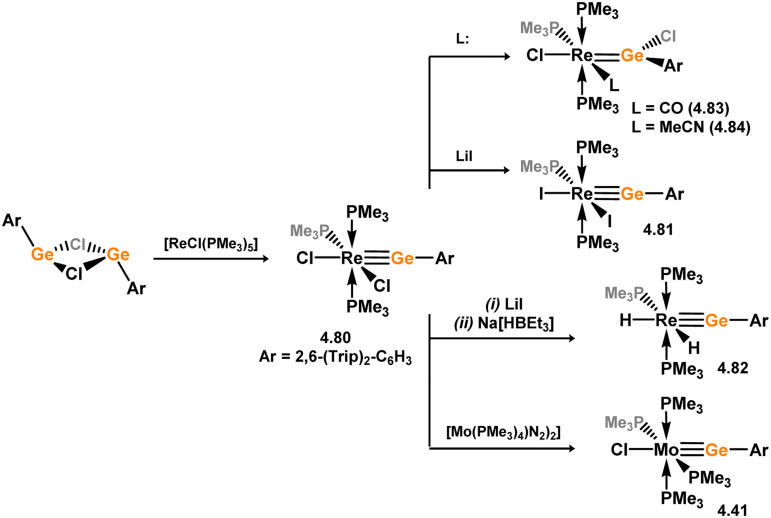
Formation and reactivity of a rhenium–germylidyne complex.

Beyond group 6, only a small number of germylidyne or metallogermylene complexes are known. The first was reported by Power and co-workers, in the ferrato-germylenes 4.76 and 4.77. These were synthesised through the salt-metathesis of (aryl)(chloro)germylenes with K[CpFe(CO)_2_], directly leading to the target compounds (Scheme 245).^427^ Surprisingly, no CO elimination was observed for these systems, under heating or through UV irradiation, in contrast to corresponding Sn systems (*vide infra*). It was later demonstrated that these species behave as classic germylenes, showing the capacity to oxidatively add both H_2_O and NH_3_ at the Ge centre, forming 4.78 and 4.79.^428^ Filippou later demonstrated that the same germylene precurors undergo an oxidative addition process with Re^I^ complex [Re(PMe_3_)_5_Cl], directly leading to germylidyne complex 4.80 (Scheme 246).^428^ This complex was shown to undergo Cl metathesis reactions (*i.e.* with [I]^−^ (4.81), [H]^−^ (4.82)), whilst addition of donor ligands (CO, MeCN) led to coordination at Re, and Cl migration to Ge in forming formal germylene complexes 4.83 and 4.84. Further, reaction of Re germylidyne complex 4.80 with [Mo(PMe_3_)_4_(N_2_)_2_] led to [RGe] transfer, so forming known Mo germylidyne complex 4.41.

**Scheme 247:**
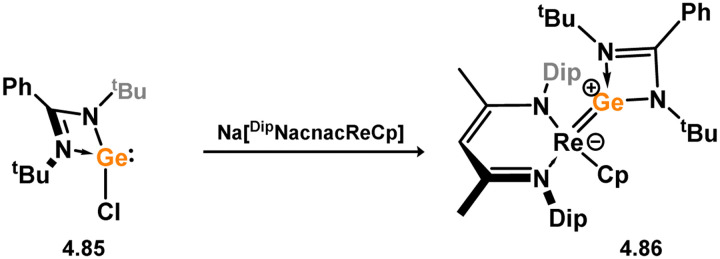
A salt-metathesis route to a doubly-bonded rhenium metallogermylene complex.

One further example of a rhenium complex is known, best described as a metallogermylene, generated through the reaction of chloro-germylene 4.85 with [^Dip^NacnacReCp]Na, in forming 4.86 (Scheme 247).^413^ Here, as per the silicon derivative described earlier in this review (*viz.* compound 4.26), there is a degree of π-bonding between the Ge and Re centres.

**Scheme 248:**
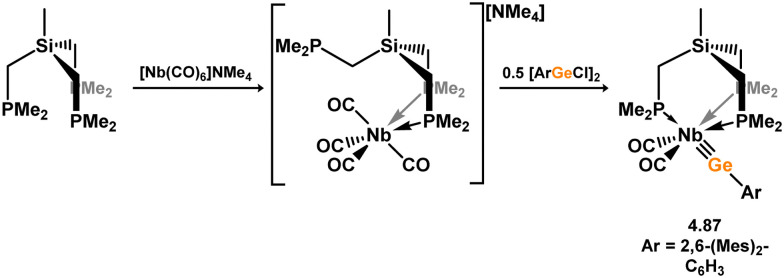
Access to a niobium–germylidyne complex.

Using a soft salt-metathesis route, Filippou and co-workers have also reported the synthesis of a Nb germylidyne complex, accessed through the addition of (aryl)(chloro)germylene to the anionic complex Me_4_N[{MeSi(CH_2_PMe_2_)_3_}Nb(CO)_4_], directly leading to the elimination of [Me_4_NCl] and two equiv. of CO, forming 4.87 (Scheme 248).^412^

**Scheme 249:**
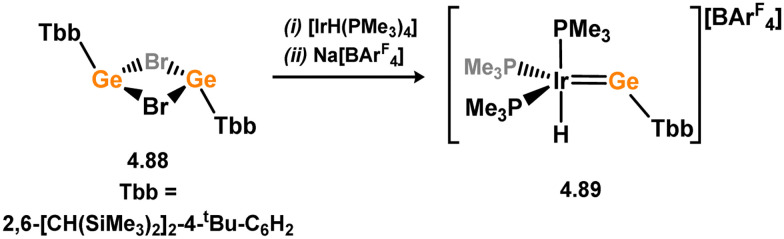
Access to a doubly-bonded metallogermylene complex of iridium.

Wesemann and co-workers have also demonstrated that Ir germylidyne complexes can be accessed, through the addition of an (aryl)(bromo)germylene 4.88 to [IrH(PMe_3_)_4_], followed by bromide abstraction with Na[BAr_4_^F^] (Scheme 249). The resulting complex (*viz.*4.89) is described as bearing a Ge–Ir double bond, due to perturbed back-bonding from Ir to Ge, lending a highly Lewis acidic character to the Ge centre.^301^ As such, a range of nucleophilic small-molecules (*e.g.* NH_3_, N_2_H_4_, H_2_O), as well as H_2_, could be activated across the Ge–Ir bond, as described in Section 3, leading to numerous novel acyclic germylene complexes. This activation mode stands in contrast to that for formal metallogermylene species, which undergo a single-centre oxidative addition reaction, opening concepts in ligand design for cooperative bond activation.

**Scheme 250:**
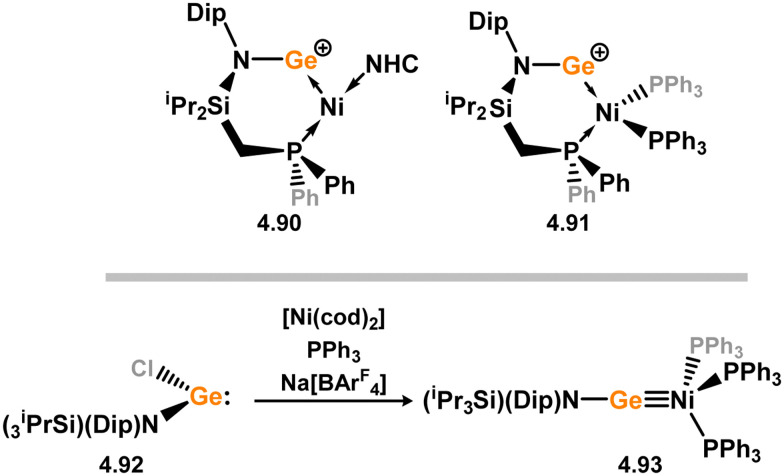
Formation of a dative triple-bond in a nickel–germylidyne complex, and its comparison to the geometrically constrained derivatives, which bear single Ge–Ni bonds.

In our group, we have specifically targeted such concepts through the development of cationic tetrylene ligands featuring a chelating arm. This has allowed for the synthesis of cationic germylene–Ni^0^ complexes 4.90 and 4.91 which may be considered as metallogermylene species, with strongly bent L–Ge–Ni angles and a high cationic charge localisation at germanium, due to a low degree of Ni → Ge back-bonding (Scheme 250, above).^316^ The (amido)(chloro)germylene 4.92 was utilised in accessing the related amido-germylidyne complex 4.93, without a chelating phosphine arm, synthesised through the one-pot reaction of PPh_3_, [Ni(cod)_2_], Na[BAr^F^_4_], and 4.92 (Scheme 250, below).^429^ This species bears a near linear L–Ge–Ni angle, formally with a donor–acceptor triple bond, indicating that the chelating phosphine arm in *e.g.*4.91 is central to its bent structure. This structure, which leads to a highly cationic Ge centre, makes 4.91 a robust catalysts for the hydrosilylation of alkenes, also demonstrating the reversible binding of ammonia at the Ge centre, as well as the ability of the Ge centre to abstract [F]^−^ from [SbF_6_]^−^. The linear derivative 4.93 is unstable under all of those conditions. We note that, given that the cationic charge in 4.90 and 4.91 resides largely at the Ge^II^ centres, these species and their derivatives are considered as germylene–Ni^0^ complexes, and as such are discussed in Section 3.

**Scheme 251:**
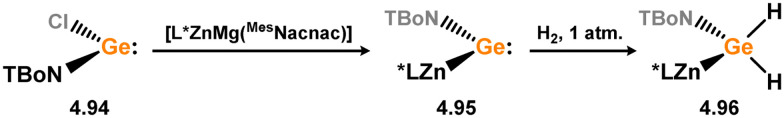
Formation of a zincogermylene, and its facile activation of H_2_.

Finally, Jones and co-workers demonstrated that a zinco-germylene could be accessed through addition of their [Mg–Zn] bonded reagent to the chloro-germylene [(^T^BoN)GeCl] (*viz.*4.94), forming 4.95 (Scheme 251).^430^ The high s-character of the Ge–Zn bond precluded significant π-bonding interactions, and also led to very narrow HOMO–LUMO gap, which pertains to the Ge-centred lone electron pair and vacant p-orbital, respectively. This allows for the activation of H_2_ at this Ge centre, so forming 4.96, although DFT calculations did show that the reaction coordinate for this activation process does involve the Zn centre in the H–H bond scission process.

### 4.3. Stannylidyne/metallostannylene systems

**Scheme 252:**
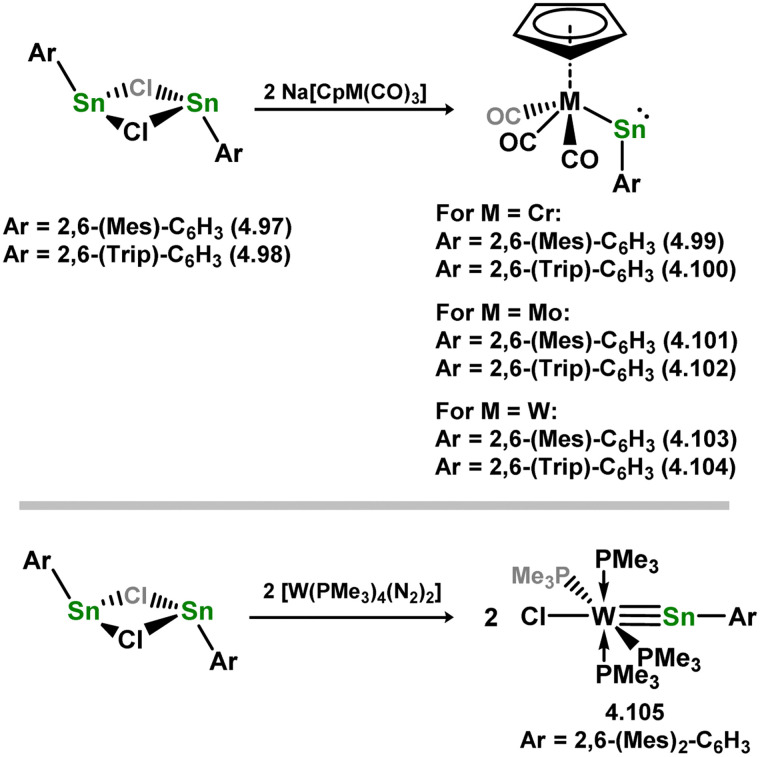
The initial examples of metallostannylene and stannylidyne complexes.

**Scheme 253:**
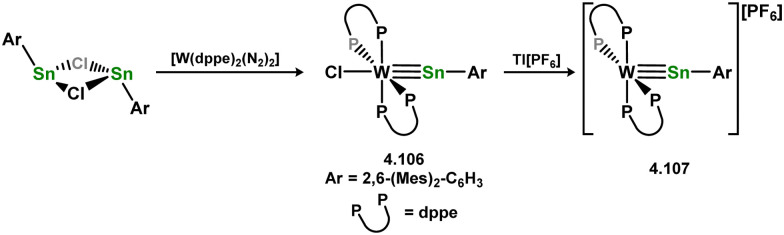
Formation of a tungsten–stannylidyne complex and its cation.

**Scheme 254:**
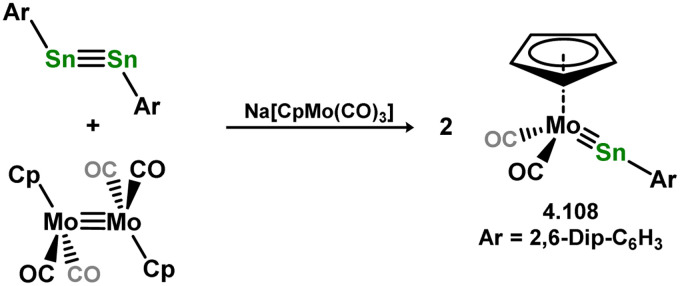
Cross-metathesis of tin(i) and molybdenum(i) dimers in the formation of a molybdenum–stannylidyne complex.

**Scheme 255:**
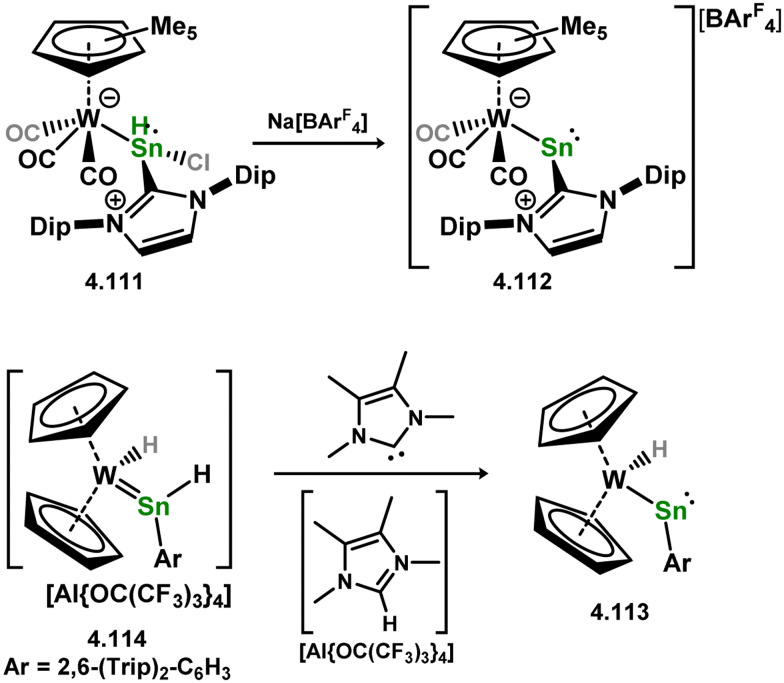
Formation of metallostannylene complexe of tungsten, with N-heterocyclic carbenes playing different roles.

As for both Ge and Si, the first examples of metallostannylenes and stannylidyne complexes were group 6 systems. Power and co-workers first reported metallostannylenes for all group 6 metals, through the addition of (aryl)(chloro)stannylenes 4.97 and 4.98 to Na[CpM(CO)_3_] (M = Cr–W), so forming complexes Cr- (4.99 and 4.100), Mo- (4.101 and 4.102), and W- (4.103 and 4.104) metallostannylene complexes with strongly bent L-*Sn*-M angles of between 106.7(10)° and 111.0(4)° (Scheme 252).^403^ Attempts to eliminate CO in these systems, to form the stannylidyne complexes, were not described. However, soon after this publication Filippou and co-workers described the synthesis of the initial example of a stannylidyne system, in tungsten complex 4.105 (Scheme 252).^399^ This species was accessed through the formal oxidative addition of (aryl)(chloro)stannylene 4.97 to [W(PMe_3_)_4_(N_2_)_2_], directly forming the target complex. The closely related chelating phosphine system (*viz.*4.106) and the subsequent cationic square-pyramidal complex 4.107 were reported soon after, the latter *via* chloride abstraction from 4.106 (Scheme 253).^431^ The triple Sn–W bonding in these two complexes is clearly borne our by their linear L–Sn–W angles (4.106 : 179.52(7)°; 4.107 : 178.77(9)°) and Sn–W bond distances (4.106 : 2.4901(7) Å; 4.107 : 2.4641(7) Å), the latter being significantly contracted relative to Power's related metallostannylenes (*i.e.*4.103 : 2.9045(10) Å; 4.104 : 2.9030(8) Å). Power and co-workers later demonstrated that molybdenum stannylidyne 4.108, related to a congener of their earlier reported metallostannylenes (*viz.*4.109), can be accessed *via* the metathesis of distannyne 4.110 with [{CpMo(CO)_2_}_2_],^420^ again with a contracted Sn–Mo bond distance relative to the metallostannylene derivative (4.109 : 2.8960(9) Å; 4.110 : 2.4691(7) Å) (Scheme 254). Filippou and co-workers also demonstrated that an imidazolium-stabilised metallostannylene is readily accessible through the chloride abstraction from 4.111, so forming 4.112 (Scheme 255).^421^ Here UV irradiation did not lead to CO elimination, but was effective in driving CO elimination from the related Ge complex (*vide supra*). Closely related, Wesemann and co-workers have also reported the synthesis of a tungsten metallostannylene, complex 4.113, generated through elimination of [NHC-H] from hydrido-stannylene complex 4.114 upon addition of a small NHC.^358^

**Scheme 256:**
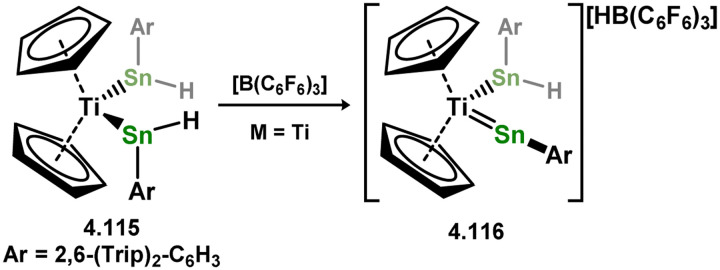
Formation of a partial titanium stannylidyne complex.

Moving to early TMs, the same group also reported that hydride abstraction from bis(hydrido-stannylene) titanium(ii) complex 4.115 led to stannylidyne-type complex 4.116, which is described as having a Sn–Ti double bond, rather than a triple bond, due to the electron poor Ti centre, thus disallowing significant back-bonding interactions (Scheme 256).^356^

**Scheme 257:**
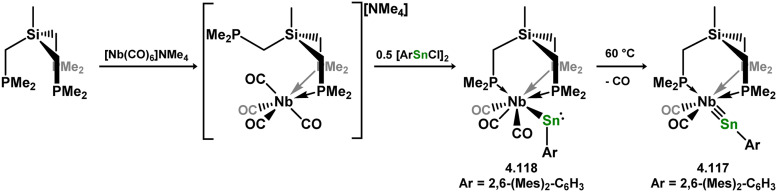
Synthesis of a niobium metallstannylene complex, and its coverstion to the stannylidyne tautomer through thermally driven CO elimination.

One group 5 stannylidyne complex is known, namely Nb complex 4.117, accessed in a similar manner to those for Ge and Si.^412^ That is, (aryl)(chloro)stannylene 4.97 reacts with Me_4_N[{MeSi(CH_2_PMe_2_)_3_}Nb(CO)_4_] through salt-metathesis, in the loss of Me_4_NCl. This initially forms tris-carbonyl complex 4.118, in contrast to aforementioned Si and Ge systems, eliminating CO to form stannylidyne complex 4.117 only upon heating (Scheme 257).

**Scheme 258:**
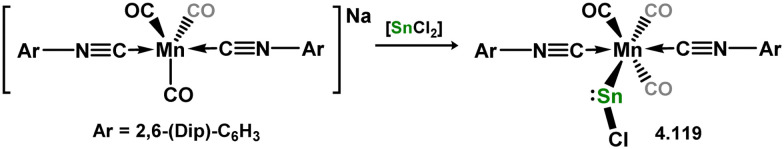
Generation of a chloro-metallostannylene of manganese through direct salt-metathesis.

**Scheme 259:**
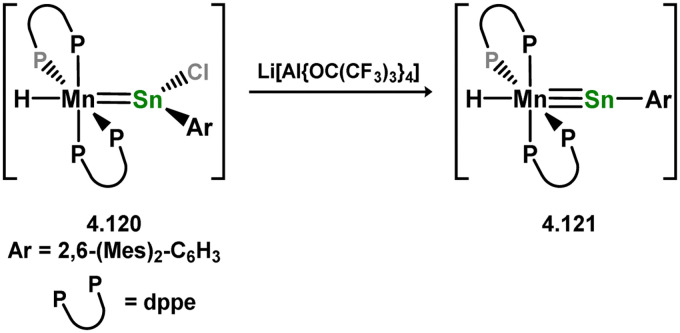
Formation of a manganese stannylidyne complex through chloride abstraction from a chloro-stannylene complex.

Group 7 metallostannylene and stannylidyne species are also known, reported by the groups of Figueroa and Filippou. The former group reported the chloro-metallostannylene complex 4.119, synthesised through the salt-metathesis of [SnCl_2_] with Na[(Ar*NC)_2_Mn(CO)_3_] (Scheme 258).^432^ Given the high coordination number at Mn it is perhaps not surprising that the stannylidyne complex is not formed, though it should be noted that such simple metallostannylene systems (*i.e.* bearing the [ClSn]^+^ ligand) are rare, and in this case is accessible due to the steric bulk of the terphenyl isocyanide ligands. Filippou and co-workers later reported that chloride abstraction from (aryl)(chloro)stannylene complex 4.120 led to the formation of complex 4.121, a formal manganese stannylidyne complex (Scheme 259).^364^

**Scheme 260:**
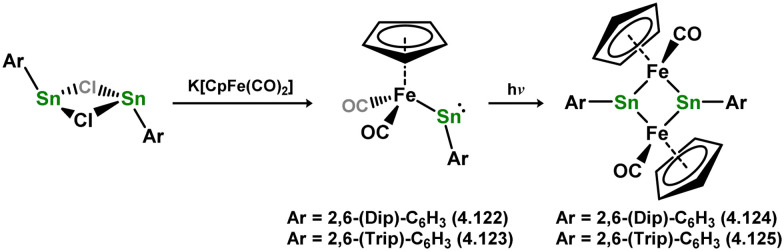
Formation of a ferratostannylene, and generation of its dimer upon CO elimination.

**Scheme 261:**
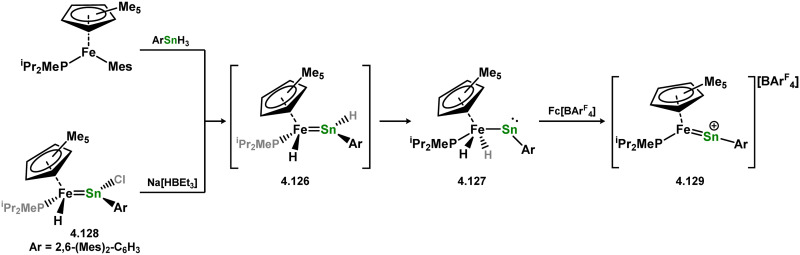
Varying routes to a ferratostannylene complex, and formation of its cation, featuring a high degree of stannylidyne character.

Moving to group 8, a number of interesting species on the continuum of metallostannylene to stannylidyne systems are known. For iron, no formal stannylidyne complexes are known, *i.e.* with triple Fe–Sn bonds. The first attempts to access such systems were reported by Power and co-workers, in complexes 4.122 and 4.123 (Scheme 260).^427^ These species do not form target stannylidyne complexes upon CO elimination, but rather form dimeric complexes 4.124 and 4.125, despite the steric bulk of the employed aryl ligands. Later, Tilley and co-workers reported a related iron metallostannylene complex, *via* Sn–to–Fe hydride-migration in (aryl)(hydrido)stannylene complex 4.126, so forming 4.127 (Scheme 261). The former stannylene complex is not observed, but is generated by either metathesis of aryl stannane with [Cp*Fe(P^i^Pr_2_Me)(Mes)], or salt-metathesis of (aryl)(chloro)stannylene complex 4.128. Oxidation of metallostannylene complex 4.127 with the ferrocenium cation leads to H_2_ elimination and formation of 4.129, in which a near linear L–Sn–Fe bond angle of 169.85(9)° is observed. Alongside the short Fe–Sn distance of 2.2889(6) Å in this species, a degree of multiple bonding is clear. This, however, is found by DFT calculations to be a double bond: the HOMO−12 and HOMO−1 are best described as σ- and π-bonding interactions, whilst the HOMO is an iron-centred lone electron pair, and the LUMO is a vacant p-orbital at tin. Nevertheless, 4.129 can be described as having significant stannylidyne character.

**Scheme 262:**
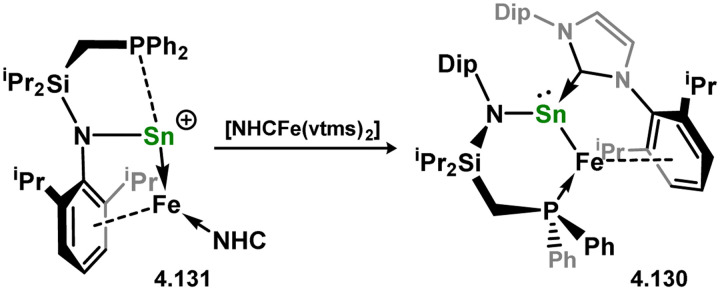
Reduction of a geometrically constratined cation-stannylene iron(0) complex, in formation of a covalently bound iron(−i) complex.

On the other extreme, our group reported a formal formally iron(−i) metallostannylene, with a long covalent Fe–Sn bond (*d* = 2.6489(9) Å), which represents the only reported example of a covalently bound iron(−i) species (*viz.*4.130, Scheme 262).^299^ This complex is readily accessed through the reduction of cationic-stannylene iron(0) complex 4.131, with the single free electron shown to have significant spin density at iron through EPR and DFT studies.

**Scheme 263:**
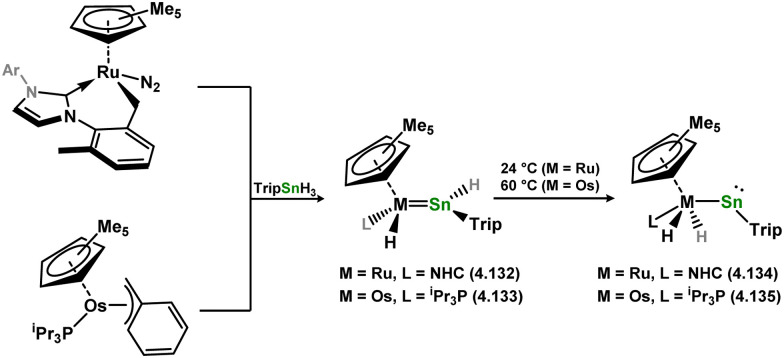
Hydride migration in hydrido–stannylene complexes, in the formation of group 8 metallostannylene complexes.

Ru and Os complexes which are direct analogues of iron-centred metallostannylene 4.127 are also known, reported by the group of Tilley. All systems were accessed *via* thermally induced hydride-migration in hydrido-stannylene complexes 4.132 and 4.133, forming metallostannylene species 4.134 and 4.135, Scheme 263).^368,369,433^

**Scheme 264:**
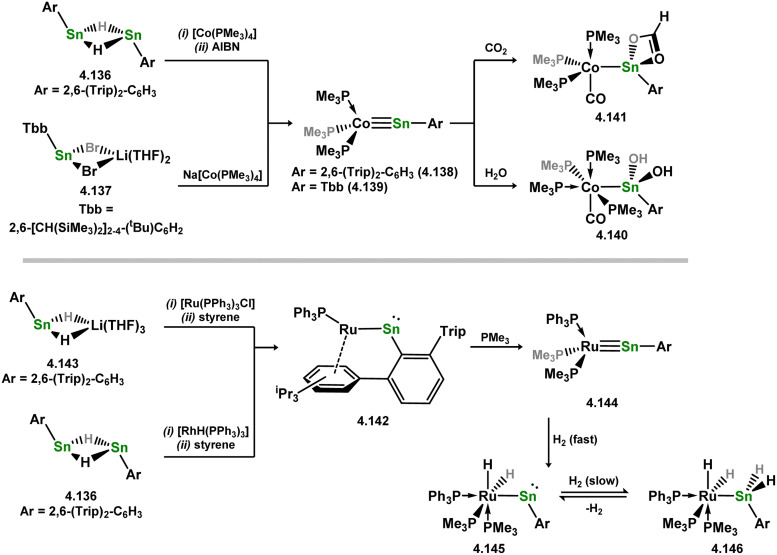
Formation and reactivity of group 9 metallostannylene and stannylidyne complexes.

Stannylidyne complexes are known for all group 9 elements, reported by the group of Wesemann. Addition of (aryl)(hydrido)stannylene 4.136 to [Co(PMe_3_)_4_] followed by the addition of AIBN, or direct salt-metathesis of stannate 4.137 with Na[Co(PMe_3_)_4_] led to cobalt complexes 4.138 and 4.139 (Scheme 264).^434^ Notably, these species were shown to activate two equivalents of H_2_O across the Sn–Co bond, and could also reduce CO_2_, forming complexes 4.140 and 4.141, respectively. The closely related Rh metallostannylene complex 4.142 was accessed through the addition of (aryl)(hydrido)stannylene 4.136 to rhodium(i) hydride complex [RhH(PPh_3_)_3_], or through the metathesis of hydrido stannate 4.143 with [RhCl(PPh_3_)_3_], forming metallostannylene 4.142, followed by alkene-mediated dihydrogen abstraction.^373^ Addition of PMe_3_ displaced the arene-interaction, leading to the formation of stannylidyne complex 4.144. This species was shown to activate H_2_, first at the Rh centre forming novel metallostannylene 4.145, and then *via* a second oxidative addition at the newly formed metallostannylene Sn centre, forming 4.146.

**Scheme 265:**
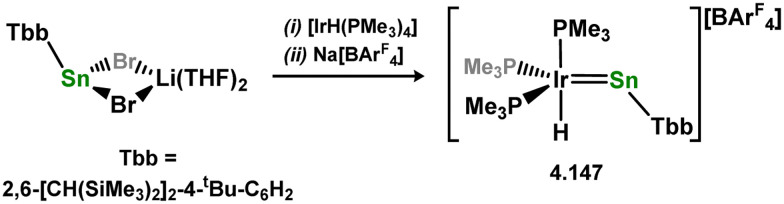
Formation of an iridium complex which sits between a formal metallostannylene and stannylidyne complex.

Ir complex 4.147 was similarly accessed through the addition of stannate 4.137 to [IrH(PMe_3_)_4_], followed by halide abstraction with Na[BAr^F^_4_] (Scheme 265).^301^ This species is best described as having an Ir–Sn double, in contrast to the formal triple bonds observed in Co and Rh complexes described above. Like the Ge congener, this leads to a significant degree of Lewis acidic character at the Sn centre, allowing for the facile activation of a range of nucleophilic small molecules across this bond, presumably *via* binding at Sn, forming a range of acyclic-stannylene complexes (*vide supra*).

**Scheme 266:**
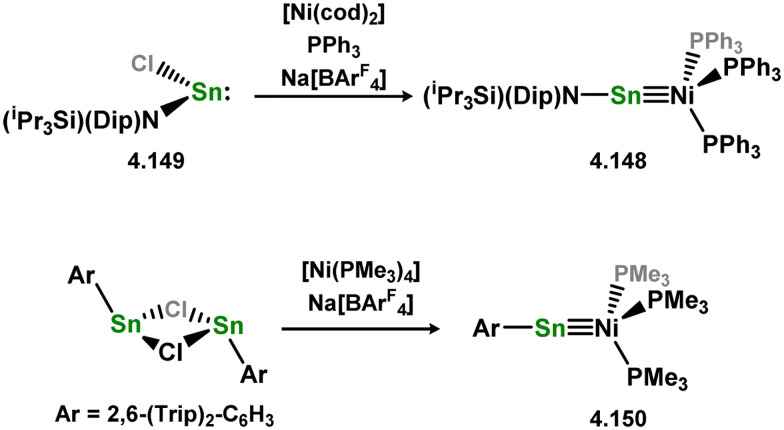
Synthesis of nickel-stannylidyne complexes.

Finally, two stannylidyne complexes are known for the group 10 metals, specifically for Ni, both of which were very recently reported, as per the corresponding Si and Ge derivatives. The initial example, 4.148, was reported by our group, and is accessed in a one-pot reaction between (amido)(chloro)stannylene 4.149, [Ni(cod)_2_], Na[BAr_4_^F^], and PPh_3_ (Scheme 266).^429^ Again, the synthesis of this system demonstrates the necessity of the chelating arm in accessing the highly bent cationic stannylene nickel complexes in Section 3.^315,316^ Following this, Filippou and co-workers reported a thorough computational investigation into the bonding situation in stannylidyne complexes for Ni, Pd, and Pt, with comparison to their isolated aryl stannylidyne 4.150, and our amido derivative 4.148.^435^ Computationally, it is observed that the heavier tetryl elements are expected to yield bent L–E–M geometries, thus deviating from tetrylidyne character. Another key point here, these group 10 metals tend to remain in their 0th oxidation state, that is with the formal charge in these cationic complexes remaining at the tetryl centre. As such, bent complexes predicted *e.g.* for platinum stannylidynes are described best as TM-stabilised cationic stannylenes, as opposed to metallostannylenes. Indeed, this is in keeping with earlier observations that the group 10 metals, including Ni, can behave as Z-type ligands towards highly Lewis acidic tetrylene centres.^183,309,315^ Nevertheless, calculated bond angles for 4.148 and 4.149 in the computational study reported by Filippou deviate by between 12 and 15° from those observed in the experimental structures, which may suggest that the degree of stannylidyne character is computational underestimated.

### 4.4. Plumbylidyne/metalloplumbylene systems

**Scheme 267:**
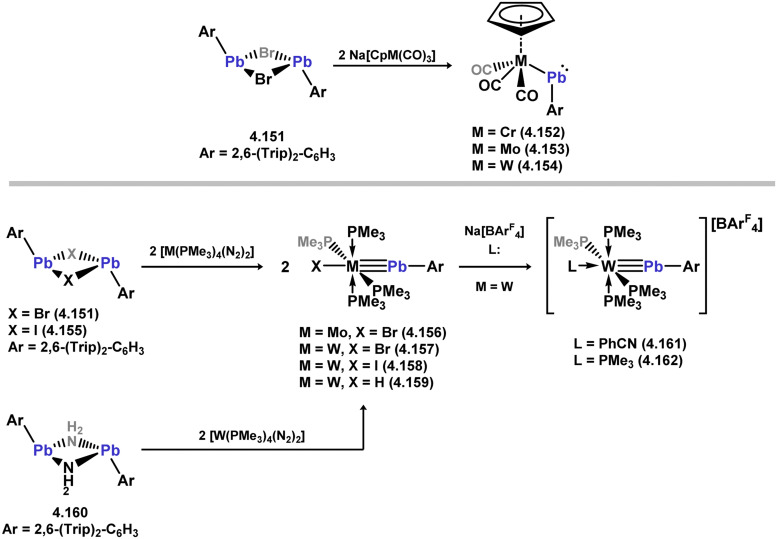
Initial examples of metalloplumbylene and plumbylidyne complexes.

**Scheme 268:**
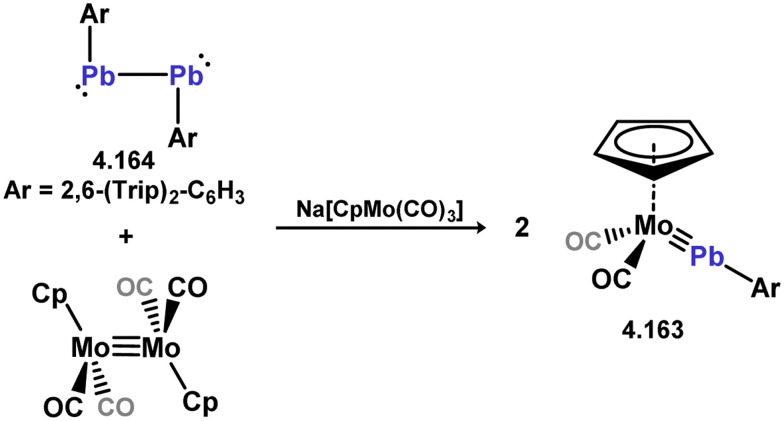
Cross-metathesis of dimeric lead(i) and molybdenum(i) species in the formation of a plumbylidyne complex.

Lead is the least likely of the group 14 elements to partake in multiple bonding, typically attributed to the so-called inert pair effect.^436,437^ As per the lighter elements, the majority of plumbylidynes involve the group 6 metals. Initial efforts in this direction were reported by Power and co-workers, whereby the addition of (aryl)(bromo)plumbylene 4.151 to Na[CpM(CO)_3_] (M = Cr, Mo, W) resulted in the formation of metalloplumbylenes for Cr (4.152), Mo (4.153), and W (4.154; Scheme 267).^404^ These species were reported as highly stable, and do not form the corresponding triply bonded complexes. Filippou later reported successful access to both Mo and W plumbylidyne complexes *via* the oxidative addition of (aryl)(bromo)- or (aryl)(iodo)-plumbylenes (4.151 and 4.155, respectively) to [M(PMe_3_)_4_(N_2_)_2_], forming complexes 4.156, 4.157, and 4.158, mirroring earlier described syntheses of Ge and Sn congeners^400,438^ It was later shown that plumbylidyne complex 4.159, with a terminal W–H ligand, can also be accessed through the activation of the (amido)(aryl)plumbylene 4.160.^439^ Additionally, halide abstraction from (bromo)tungsten-stannylidyne complex 4.157 with Na[BAr^F^_4_], in the presence of PhCN or PMe_3_, leads to the formation of cationic adducts 4.161 and 4.162. Power and co-workers were later able to demonstrate that plumbylidyne complex 4.163, closely related to the earlier reported metalloplumbylene 4.153, can be accessed through the cross-metathesis of their diplumbyne 4.164, better described as a bis(plumbylene), and [CpMo(CO)_2_]_2_, as discussed earlier for Ge and Sn (Scheme 268).^420^ As per those examples, comparison of metalloplumbylene and plumbylidyne complexes 4.153 and 4.163, respectively, clearly deomstrates a contraction in the Mo–Pb bond distance (*d*_MoPb_ in 4.153 = 2.9845(7) Å; in 4.163 = 2.5143(2) Å), and a switch from a strongly bent Ar–Pb–Mo angle in 4.153 (110.00(13)°) to a linear binding mode in 4.163 (175.03(7)°).

**Scheme 269:**
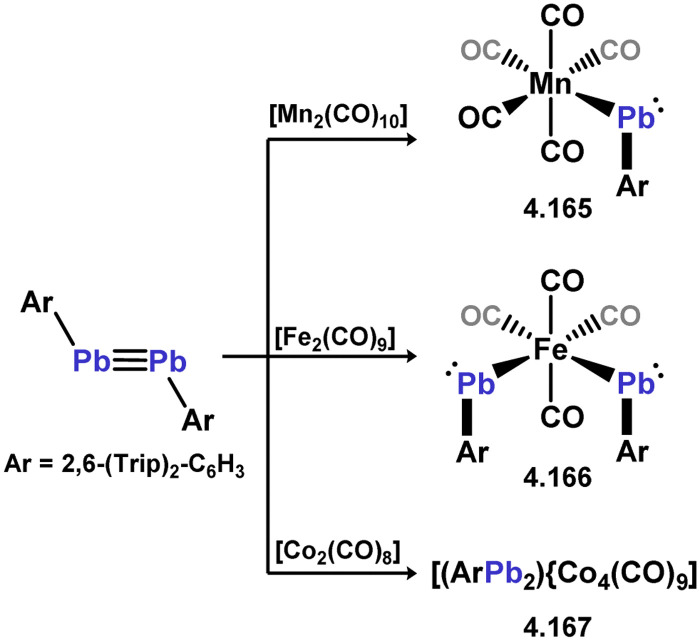
Cross-metathesis reactivity of a lead(i) dimer towards manganese(0), iron(0) and cobalt(0) carbonyl complexes.

Similar cross-metathesis chemistry has also been reported for diplumbyne 4.164 with Mn^0^, Fe^0^, and Co^0^ carbonyl species, leading to 3d transition metal metalloplumbylene systems 4.165, 4.166, and 4.167 (Scheme 269).^440^ These reactions proceed in the absence of light irradiation, *via* direct combination of 4.164 with the dimeric TM carbonyl species. Again, no loss of CO in the formation of plumbylidynes was observed.

**Scheme 270:**
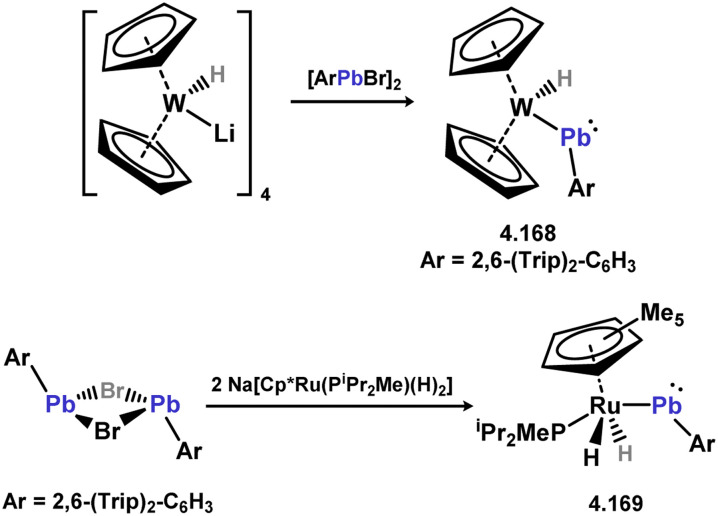
Salt-metathesis routes to metalloplumbylenes.

**Scheme 271:**
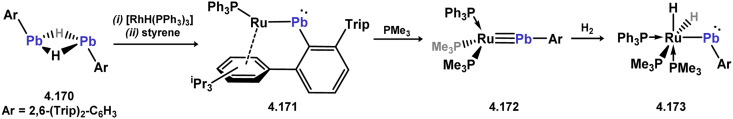
A ruthenium metalloplumbylene complex, its conversion to plumbylidyne species, and subsequent H_2_ activaiton.

A further example of a tungsten-centred metalloplumbylene was reported by Wesemann and co-workers, in the salt-metathesis reaction of (aryl)(bromo)plumbylene 4.151 with Li[Cp_2_W(H)], forming 4.168.^358^ A similar synthetic methodology has also been employed by Tilley and co-workers for the formation of Ru metalloplumbylene 4.169, in the reaction of [Na{Cp*Ru(^i^Pr_2_PMe)(H)_2_}]_2_ with the same plumbylene,^369^ again with no observation of *e.g.* H_2_ loss in the formation of a triply bonded species (Scheme 270). In contrast, Wesemann and co-workers later reported that thermally sensitive lead(ii) hydride 4.170 undergoes a formal oxidative addition to [Rh(PPh_3_)_3_H], forming dihydride complex 4.171, as earlier described for the related Sn system (Scheme 271).^373^ This species undergoes an H_2_ abstraction reaction when reacted with styrene, in the presence of PMe_3_, forming plumbylidyne 4.172. In contrast to the Sn congener, which activates H_2_ both at the Rh and Sn centres, 4.172 reacts with only one equiv. H_2_, exclusively at Rh, in forming a further metalloplumbylene complex, 4.173.

### 4.5. Appraisal of the field of tetrylidyne/metallotetrylene chemistry

The central deviation of the heavier group 14 elements relative to carbon in this class of compounds is the diverging propensity to form multiple bonds. This allows, to some degree, for tuning of the metal–tetrylyne interaction through ligand design, metal choice, and/or metal coordination environment to switch between metallotetrylene and tetrylidyne complexes. This is clearly demonstrated in initial syntheses of such complexes, whereby CO elimination from metallotetrylene systems reduces the coordination number at M, leading to E–M triple-bond formation in tetrylidyne complexes.^398^ Additionally, periodicity also plays a role here: multiple bonding is preferred for the lighter elements, leading to more ready formation of tetrylidyne species, *e.g.* through spontaneous CO elimination leading to germylidyne complex 4.29, than for the heavier elements, *e.g.* whereby analogous metallostannylene 4.101 does not eliminate CO even under heating or irradiation, as demonstrated by Power and co-workers.^403^ As discussed earlier in this review, and unsurprisingly, by far the most utilised transition metals for the formation of tetrylidyne complexes are the heavier group 6 metals, Mo and W, given their known propensity to form triple M–N and M–C bonds, with over 40 such examples in total for Si–Pb. Amongst these, Power and co-workers have reported an analogous series of molybdenum tetrylidyne complexes for Ge–Pb; a similar deviation of 0.46–0.49 Å from the sum of covalent radii (Ge: 2.74 Å; Sn: 2.93 Å; Pb: 3.00 Å) is observed in all cases, which would suggest that all demonstrate a similar degree of triple-bond character.^420^

What remains to be thoroughly explored is the reactive chemistry of both tetrylidyne and metallotetrylene species. A few key examples of known reactivity are mentioned here: power and coworkers have demonstrated that tetrylidyne complexes can be accessed by metathetical exchange between M–M and E–E dimers (M = Mo, W; E = Ge–Pb),^420^ which may later allow for E–E′ triple bond cross metathesis (E′ = Si–Pb; E ≠ E′). The groups of Power, Jones, Wesemann, and others have also demonstrated the ready oxidative addition of small molecules such as H_2_ and NH_3_ at the E centre of metallotetrylenes,^329,373,430^ whilst Wesemann and Tobita have shown that tetrylidynes tend to undergo addition across the E–M triple bond.^156,301,373^ What perhaps remains to be seen here is well-defined reversible examples, which may allow for further bond functionalisation and applications in catalytic transformations. In this regard, A firm understanding of ligand, M, and E effects in such chemistry will be of great utility.

## 5. Summary and outlook

As demonstrated by the sheer number of complexes described in this review, the fields of heavier tetrylene and tetrylyne transition metal chemistry have seen momentous interest since their respective inceptions towards the close of the 19th century. In a similar vein to carbene chemistry, this has flourished on several fronts: fundamental studies regarding the electronic nature and bonding in these complexes have been numerous, which has now led to an array of systems which show the capacity for cooperative bond activation in conjunction with a transition metal centre. Additionally, akin to NHCs, the number of tetrylenes employed as effective ligands in transition metal catalysis continues to grow, in some cases leading to activities exceeding those of classical ligand systems, and additionally leading to unique reactive mechanisms.

Some key trends can be summarised here: (i) as expected, lower E–TM bond orders are expected on decending the group 14 elements, in line with the lessened propensity for *s*-based electrons to partake in bonding interations, particularly for lead; (ii) unlike carbenes, heavier tetrylene/yne centres are typically electropositive when bound to a transition metal, which can allow for ligand centred nucleophile binding in both tetrylene and tetrylyne complexes; (iii) the mono- and divalent-states for the heavier group 14 elements are much more readily accessible than for carbon, and of course Si, Ge, Sn, and Pb each have a unique electronic character, giving an infinitely broad landscape for the development of non-innocent, and highly tunable ligand systems.

With these above points, the future looks bright for this field of low oxidation-state group 14 chemistry. With the cooperative, and sometimes reversible activation of synthetically key molecules such as CO_2_, H_2_, and even NH_3_ already known, it is only a matter of time until the catalytic utilisation of such processes is realised. With the growing interest in leveraging such chemistry, and the ever pressing challenges of establishing sustainable chemical technologies, we look forward to witnessing, and indeed being part of this new frontier in heavier p-block chemistry.

## Data availability

No primary research data are included in this review, and as such data sharing is not applicable here.

## Conflicts of interest

There are no conflicts of interest to declare.

## References

[cit1] Bertrand G. , Carbene chemistry: from fleeting intermediates to powerful reagents, ed., M. Dekker, FontisMedia, New York, Lausanne, Switzerland, 2002

[cit2] Hahn F. E., Jahnke M. C. (2008). Angew. Chem., Int. Ed..

[cit3] De Frémont P., Marion N., Nolan S. P. (2009). Coord. Chem. Rev..

[cit4] Contemporary Carbene Chemistry, ed. R. A. Moss and M. P. Doyle, Wiley, 1st edn, 2013

[cit5] Dötz K. H. , Metal Carbenes in Organic Synthesis, Springer Berlin Heidelberg, Berlin, Heidelberg, 2004, vol. 13

[cit6] Dötz K. H., Stendel J. (2009). Chem. Rev..

[cit7] Transition Metal Carbyne Complexes, ed. F. R. Kreißl, Springer, Netherlands, Dordrecht, 1993

[cit8] Carbyne complexes: this book is dedicated to Ernst Otto Fischer on the occasion of his 70. birthday, ed. H. Fischer and E. O. Fischer, VCH, Weinheim, New York, 1988

[cit9] Cui M., Jia G. (2022). J. Am. Chem. Soc..

[cit10] Schrock R. R., Czekelius C. (2007). Adv. Synth. Catal..

[cit11] Alkene Metathesis in Organic Synthesis, ed. A. Fürstner, Springer Berlin Heidelberg, Berlin, Heidelberg, 1998, vol. 1

[cit12] Olefin Metathesis: Theory and Practice, ed. K. Grela, Wiley, 1st edn, 2014

[cit13] Handbook of Metathesis, ed. R. H. Grubbs, A. G. Wenzel, D. J. O’Leary and E. Khosravi, Wiley, 1st edn, 2015

[cit14] Fürstner A. (2021). J. Am. Chem. Soc..

[cit15] Ge Y., Hu Y., Duan G., Jin Y., Zhang W. (2022). Trends Chem..

[cit16] Glorius F. , N-Heterocyclic Carbenes in Transition Metal Catalysis, Springer Berlin Heidelberg, Berlin, Heidelberg, 2007, vol. 21

[cit17] N-Heterocyclic Carbenes: From Laboratory Curiosities to Efficient Synthetic Tools, ed. S. Diez-Gonzalez, Royal Society of Chemistry, Cambridge, 2nd edn, 2016

[cit18] Nesterov V., Reiter D., Bag P., Frisch P., Holzner R., Porzelt A., Inoue S. (2018). Chem. Rev..

[cit19] Power P. P. (2007). Organometallics.

[cit20] Fischer R. C., Power P. P. (2010). Chem. Rev..

[cit21] Hadlington T. J., Driess M., Jones C. (2018). Chem. Soc. Rev..

[cit22] Waterman R., Hayes P. G., Tilley T. D. (2007). Acc. Chem. Res..

[cit23] Ghosh M., Sen N., Khan S. (2022). ACS Omega.

[cit24] Komuro T., Nakajima Y., Takaya J., Hashimoto H. (2022). Coord. Chem. Rev..

[cit25] Cabeza J. A., García-Álvarez P., Laglera-Gándara C. J. (2020). Eur. J. Inorg. Chem..

[cit26] Cabeza J. A., García-Álvarez P. (2023). Chem. – Eur. J..

[cit27] Somerville R. J., Campos J. (2021). Eur. J. Inorg. Chem..

[cit28] Spikes G. H., Fettinger J. C., Power P. P. (2005). J. Am. Chem. Soc..

[cit29] Protchenko A. V., Birjkumar K. H., Dange D., Schwarz A. D., Vidovic D., Jones C., Kaltsoyannis N., Mountford P., Aldridge S. (2012). J. Am. Chem. Soc..

[cit30] Protchenko A. V., Bates J. I., Saleh L. M. A., Blake M. P., Schwarz A. D., Kolychev E. L., Thompson A. L., Jones C., Mountford P., Aldridge S. (2016). J. Am. Chem. Soc..

[cit31] Takeuchi K., Ikoshi M., Ichinohe M., Sekiguchi A. (2010). J. Am. Chem. Soc..

[cit32] Peng Y., Guo J.-D., Ellis B. D., Zhu Z., Fettinger J. C., Nagase S., Power P. P. (2009). J. Am. Chem. Soc..

[cit33] Hadlington T. J., Hermann M., Frenking G., Jones C. (2015). Chem. Sci..

[cit34] Zhu H., Kostenko A., Franz D., Hanusch F., Inoue S. (2023). J. Am. Chem. Soc..

[cit35] Rodriguez R., Gau D., Kato T., Saffon-Merceron N., De Cózar A., Cossío F. P., Baceiredo A. (2011). Angew. Chem., Int. Ed..

[cit36] Lips F., Fettinger J. C., Mansikkamäki A., Tuononen H. M., Power P. P. (2014). J. Am. Chem. Soc..

[cit37] Wang Y., Kostenko A., Hadlington T. J., Luecke M.-P., Yao S., Driess M. (2019). J. Am. Chem. Soc..

[cit38] Ganesamoorthy C., Schoening J., Wölper C., Song L., Schreiner P. R., Schulz S. (2020). Nat. Chem..

[cit39] Reiter D., Holzner R., Porzelt A., Frisch P., Inoue S. (2020). Nat. Chem..

[cit40] Hadlington T. J., Kefalidis C. E., Maron L., Jones C. (2017). ACS Catal..

[cit41] Liu X., Xiao X.-Q., Xu Z., Yang X., Li Z., Dong Z., Yan C., Lai G., Kira M. (2014). Organometallics.

[cit42] Power P. P. (2010). Nature.

[cit43] Weetman C. , in Encyclopedia of Inorganic and Bioinorganic Chemistry, ed. R. A. Scott, Wiley, 2nd edn, 2021, pp. 1–27

[cit44] Oberdorf K., Lichtenberg C. (2023). Chem. Commun..

[cit45] Gutsulyak D. V., Piers W. E., Borau-Garcia J., Parvez M. (2013). J. Am. Chem. Soc..

[cit46] Brown R. M., Borau Garcia J., Valjus J., Roberts C. J., Tuononen H. M., Parvez M., Roesler R. (2015). Angew. Chem..

[cit47] Litz K. E., Henderson K., Gourley R. W., Holl M. M. B. (1995). Organometallics.

[cit48] Feldman J. D., Mitchell G. P., Nolte J.-O., Tilley T. D. (2003). Can. J. Chem..

[cit49] Hadlington T. J., Kostenko A., Driess M. (2020). Chem. – Eur. J..

[cit50] Cotton J. D., Davison P. J., Goldberg D. E., Lappert M. F., Thomas K. M. (1974). J. Chem. Soc., Chem. Commun..

[cit51] Lappert M. F., Miles S. J., Power P. P., Carty A. J., Taylor N. J. (1977). J. Chem. Soc., Chem. Commun..

[cit52] Grumbine S. D., Tilley T. D., Arnold F. P., Rheingold A. L. (1993). J. Am. Chem. Soc..

[cit53] Heitmann D., Pape T., Hepp A., Mück-Lichtenfeld C., Grimme S., Hahn F. E. (2011). J. Am. Chem. Soc..

[cit54] Davidson P. J., Lappert M. F. (1973). J. Chem. Soc., Chem. Commun..

[cit55] Davidson P. J., Harris D. H., Lappert M. F. (1976). J. Chem. Soc., Dalton Trans..

[cit56] Gynane M. J. S., Harris D. H., Lappert M. F., Power P. P., Rivière P., Rivière-Baudet M. (1977). J. Chem. Soc., Dalton Trans..

[cit57] Arduengo A. J., Harlow R. L., Kline M. (1991). J. Am. Chem. Soc..

[cit58] Denk M., Lennon R., Hayashi R., West R., Belyakov A. V., Verne H. P., Haaland A., Wagner M., Metzler N. (1994). J. Am. Chem. Soc..

[cit59] Glaser P. B., Tilley T. D. (2003). J. Am. Chem. Soc..

[cit60] Tuttle T., Wang D., Thiel W., Köhler J., Hofmann M., Weis J. (2007). J. Organomet. Chem..

[cit61] Blom B., Gallego D., Driess M. (2014). Inorg. Chem. Front..

[cit62] Benedek Z., Szilvási T. (2017). Organometallics.

[cit63] Straus D. A., Tilley T. D., Rheingold A. L., Geib S. J. (1987). J. Am. Chem. Soc..

[cit64] Zybill C., Müller G. (1987). Angew. Chem., Int. Ed. Engl..

[cit65] Zybill C., Mueller G. (1988). Organometallics.

[cit66] Zybill C., Wilkinson D. L., Leis C., Müller G. (1989). Angew. Chem., Int. Ed. Engl..

[cit67] Ueno K., Tobita H., Shimoi M., Ogino H. (1988). J. Am. Chem. Soc..

[cit68] Tobita H., Ueno K., Shimoi M., Ogino H. (1990). J. Am. Chem. Soc..

[cit69] So C., Roesky H. W., Magull J., Ostwald R. B. (2006). Angew. Chem., Int. Ed..

[cit70] Driess M., Yao S., Brym M., Van Wüllen C., Lentz D. (2006). J. Am. Chem. Soc..

[cit71] Sen S. S., Roesky H. W., Stern D., Henn J., Stalke D. (2010). J. Am. Chem. Soc..

[cit72] Junold K., Baus J. A., Burschka C., Tacke R. (2012). Angew. Chem., Int. Ed..

[cit73] Mück F. M., Junold K., Baus J. A., Burschka C., Tacke R. (2013). Eur. J. Inorg. Chem..

[cit74] Sun X., Simler T., Kraetschmer F., Roesky P. W. (2021). Organometallics.

[cit75] Blom B., Driess M., Gallego D., Inoue S. (2012). Chem. – Eur. J..

[cit76] Azhakar R., Ghadwal R. S., Roesky H. W., Hey J., Stalke D. (2012). Chem. – Asian J..

[cit77] Junold K., Baus J. A., Burschka C., Vent-Schmidt T., Riedel S., Tacke R. (2013). Inorg. Chem..

[cit78] Mück F. M., Kloß D., Baus J. A., Burschka C., Tacke R. (2014). Chem. – Eur. J..

[cit79] Azhakar R., Ghadwal R. S., Roesky H. W., Wolf H., Stalke D. (2012). J. Am. Chem. Soc..

[cit80] Yeong H.-X., Li Y., So C.-W. (2014). Organometallics.

[cit81] Breit N. C., Szilvási T., Inoue S. (2015). Chem. Commun..

[cit82] Garg P., Carpentier A., Douair I., Dange D., Jiang Y., Yuvaraj K., Maron L., Jones C. (2022). Angew. Chem., Int. Ed..

[cit83] Azhakar R., Sarish S. P., Roesky H. W., Hey J., Stalke D. (2011). Inorg. Chem..

[cit84] Azhakar R., Roesky H. W., Holstein J. J., Dittrich B. (2012). Dalton Trans..

[cit85] He Z., Xue X., Liu Y., Yu N., Krogman J. P. (2020). Dalton Trans..

[cit86] Blom B., Pohl M., Tan G., Gallego D., Driess M. (2014). Organometallics.

[cit87] Yang W., Fu H., Wang H., Chen M., Ding Y., Roesky H. W., Jana A. (2009). Inorg. Chem..

[cit88] Tacke R., Kobelt C., Baus J. A., Bertermann R., Burschka C. (2015). Dalton Trans..

[cit89] Du S., Jia H., Rong H., Song H., Cui C., Mo Z. (2022). Angew. Chem., Int. Ed..

[cit90] Takahashi S., Nakaya K., Ishii A., Nakata N. (2022). Molbank.

[cit91] Sodreau A., Lentz N., Frutos M., Mallet-Ladeira S., Nayral C., Delpech F., Madec D. (2019). Chem. Commun..

[cit92] Blom B., Enthaler S., Inoue S., Irran E., Driess M. (2013). J. Am. Chem. Soc..

[cit93] Breit N. C., Eisenhut C., Inoue S. (2016). Chem. Commun..

[cit94] Bai Y., Zhang J., Cui C. (2018). Chem. Commun..

[cit95] Tanabe Y., Nishibayashi Y. (2019). Coord. Chem. Rev..

[cit96] Azhakar R., Ghadwal R. S., Roesky H. W., Hey J., Krause L., Stalke D. (2013). Dalton Trans..

[cit97] Khoo S., Cao J., Yang M., Shan Y., Su M., So C. (2018). Chem. – Eur. J..

[cit98] Qi X., Sun H., Li X., Fuhr O., Fenske D. (2018). Dalton Trans..

[cit99] Cabeza J. A., García-Álvarez P., González-Álvarez L. (2017). Chem. Commun..

[cit100] Rottschäfer D., Blomeyer S., Neumann B., Stammler H., Ghadwal R. S. (2018). Chem. – Eur. J..

[cit101] Kaufmann S., Schäfer S., Gamer M. T., Roesky P. W. (2017). Dalton Trans..

[cit102] Poitiers N. E., Giarrana L., Huch V., Zimmer M., Scheschkewitz D. (2020). Chem. Sci..

[cit103] Mo Z., Kostenko A., Zhou Y., Yao S., Driess M. (2018). Chem. – Eur. J..

[cit104] Takahashi S., Sekiguchi J., Ishii A., Nakata N. (2021). Angew. Chem., Int. Ed..

[cit105] Sinha A., Wahidur Rahaman S. M., Sarkar M., Saha B., Daw P., Bera J. K. (2009). Inorg. Chem..

[cit106] Tan G., Blom B., Gallego D., Driess M. (2014). Organometallics.

[cit107] Khan S., Ahirwar S. K., Pal S., Parvin N., Kathewad N. (2015). Organometallics.

[cit108] Parvin N., Dasgupta R., Pal S., Sen S. S., Khan S. (2017). Dalton Trans..

[cit109] Parvin N., Pal S., Echeverría J., Alvarez S., Khan S. (2018). Chem. Sci..

[cit110] Parvin N., Hossain J., George A., Parameswaran P., Khan S. (2020). Chem. Commun..

[cit111] Parvin N., Pal S., Khan S., Das S., Pati S. K., Roesky H. W. (2017). Inorg. Chem..

[cit112] Schäfer S., Köppe R., Gamer M. T., Roesky P. W. (2014). Chem. Commun..

[cit113] Schäfer S., Köppe R., Roesky P. W. (2016). Chem. – Eur. J..

[cit114] Yadav S., Sangtani E., Dhawan D., Gonnade R. G., Ghosh D., Sen S. S. (2017). Dalton Trans..

[cit115] Hänninen M. M., Baldansuren A., Pugh T. (2017). Dalton Trans..

[cit116] Schmedake T. A., Haaf M., Paradise B. J., Millevolte A. J., Powell D. R., West R. (2001). J. Organomet. Chem..

[cit117] Clendenning S. B., Gehrhus B., Hitchcock P. B., Moser D. F., Nixon J. F., West R. (2002). J. Chem. Soc., Dalton Trans..

[cit118] Petri S. H. A., Eikenberg D., Neumann B., Stammler H.-G., Jutzi P. (1999). Organometallics.

[cit119] Zark P., Schäfer A., Mitra A., Haase D., Saak W., West R., Müller T. (2010). J. Organomet. Chem..

[cit120] Krahfuß M. J., Nitsch J., Bickelhaupt F. M., Marder T. B., Radius U. (2020). Chem. – Eur. J..

[cit121] Krahfuss M. J., Radius U. (2020). Inorg. Chem..

[cit122] Hänninen M. M., Pal K., Day B. M., Pugh T., Layfield R. A. (2016). Dalton Trans..

[cit123] Yoo H., Carroll P. J., Berry D. H. (2006). J. Am. Chem. Soc..

[cit124] Neumann E., Pfaltz A. (2005). Organometallics.

[cit125] Gehrhus B., Hitchcock P. B., Lappert M. F., Maciejewski H. (1998). Organometallics.

[cit126] Kong L., Zhang J., Song H., Cui C. (2009). Dalton Trans..

[cit127] Avent A. G., Gehrhus B., Hitchcock P. B., Lappert M. F., Maciejewski H. (2003). J. Organomet. Chem..

[cit128] Meltzer A., Präsang C., Milsmann C., Driess M. (2009). Angew. Chem., Int. Ed..

[cit129] Meltzer A., Präsang C., Driess M. (2009). J. Am. Chem. Soc..

[cit130] Meltzer A., Inoue S., Präsang C., Driess M. (2010). J. Am. Chem. Soc..

[cit131] Stoelzel M., Präsang C., Inoue S., Enthaler S., Driess M. (2012). Angew. Chem., Int. Ed..

[cit132] Jungton A.-K., Meltzer A., Präsang C., Braun T., Driess M., Penner A. (2010). Dalton Trans..

[cit133] Kira M., Ishida S., Iwamoto T., Kabuto C. (1999). J. Am. Chem. Soc..

[cit134] Kosai T., Ishida S., Iwamoto T. (2016). Angew. Chem., Int. Ed..

[cit135] Watanabe C., Inagawa Y., Iwamoto T., Kira M. (2010). Dalton Trans..

[cit136] Watanabe C., Iwamoto T., Kabuto C., Kira M. (2008). Angew. Chem., Int. Ed..

[cit137] Inagawa Y., Ishida S., Iwamoto T. (2014). Chem. Lett..

[cit138] Iimura T., Akasaka N., Iwamoto T. (2016). Organometallics.

[cit139] Abe S., Kosai T., Iimura T., Iwamoto T. (2020). Eur. J. Inorg. Chem..

[cit140] Iimura T., Akasaka N., Kosai T., Iwamoto T. (2017). Dalton Trans..

[cit141] Abe S., Inagawa Y., Kobayashi R., Ishida S., Iwamoto T. (2022). Organometallics.

[cit142] Rosas-Sánchez A., Alvarado-Beltran I., Baceiredo A., Saffon-Merceron N., Massou S., Branchadell V., Kato T. (2017). Angew. Chem., Int. Ed..

[cit143] Alvarado-Beltran I., Baceiredo A., Saffon-Merceron N., Branchadell V., Kato T. (2016). Angew. Chem., Int. Ed..

[cit144] Rekken B. D., Brown T. M., Fettinger J. C., Tuononen H. M., Power P. P. (2012). J. Am. Chem. Soc..

[cit145] Protchenko A. V., Schwarz A. D., Blake M. P., Jones C., Kaltsoyannis N., Mountford P., Aldridge S. (2013). Angew. Chem., Int. Ed..

[cit146] Hadlington T. J., Abdalla J. A. B., Tirfoin R., Aldridge S., Jones C. (2016). Chem. Commun..

[cit147] Mitchell G. P., Tilley T. D., Yap G. P. A., Rheingold A. L. (1995). Organometallics.

[cit148] Mork B. V., Tilley T. D. (2003). Angew. Chem., Int. Ed..

[cit149] Mork B. V., Tilley T. D., Schultz A. J., Cowan J. A. (2004). J. Am. Chem. Soc..

[cit150] Mork B. V., Tilley T. D. (2004). J. Am. Chem. Soc..

[cit151] Watanabe T., Hashimoto H., Tobita H. (2012). Chem. – Asian J..

[cit152] Ochiai M., Hashimoto H., Tobita H. (2012). Organometallics.

[cit153] Fukuda T., Hashimoto H., Sakaki S., Tobita H. (2016). Angew. Chem., Int. Ed..

[cit154] Filippou A. C., Chernov O., Stumpf K. W., Schnakenburg G. (2010). Angew. Chem., Int. Ed..

[cit155] Filippou A. C., Chernov O., Schnakenburg G. (2011). Angew. Chem., Int. Ed..

[cit156] Yoshimoto T., Hashimoto H., Hayakawa N., Matsuo T., Tobita H. (2016). Organometallics.

[cit157] Yoshimoto T., Hashimoto H., Ray M., Hayakawa N., Matsuo T., Chakrabarti J., Tobita H. (2020). Chem. Lett..

[cit158] Price J. S., Emslie D. J. H., Britten J. F. (2017). Angew. Chem., Int. Ed..

[cit159] Straus D. A., Grumbine S. D., Tilley T. D. (1990). J. Am. Chem. Soc..

[cit160] Grumbine S. K., Tilley T. D., Arnold F. P., Rheingold A. L. (1994). J. Am. Chem. Soc..

[cit161] Tobita H., Matsuda A., Hashimoto H., Ueno K., Ogino H. (2004). Angew. Chem., Int. Ed..

[cit162] Seyferth D., Annarelli D. C., Vick S. C. (1984). J. Organomet. Chem..

[cit163] Ishikawa M., Nishimura K., Sugisawa H., Kumada M. (1980). J. Organomet. Chem..

[cit164] Ohshita J., Honda N., Nada K., Iida T., Mihara T., Matsuo Y., Kunai A., Naka A., Ishikawa M. (2003). Organometallics.

[cit165] Boudjouk P., Samaraweera U., Sooriyakumaran R., Chrusciel J., Anderson K. R. (1988). Angew. Chem., Int. Ed. Engl..

[cit166] Saurwein A., Eisner T., Inoue S., Rieger B. (2022). Organometallics.

[cit167] Davidson I. M. T., Fenton A. (1985). Organometallics.

[cit168] Hayes P. G., Beddie C., Hall M. B., Waterman R., Tilley T. D. (2006). J. Am. Chem. Soc..

[cit169] Ochiai M., Hashimoto H., Tobita H. (2007). Angew. Chem., Int. Ed..

[cit170] Hayes P. G., Waterman R., Glaser P. B., Tilley T. D. (2009). Organometallics.

[cit171] Fasulo M. E., Lipke M. C., Tilley T. D. (2013). Chem. Sci..

[cit172] Takaoka A., Mendiratta A., Peters J. C. (2009). Organometallics.

[cit173] Frisch P., Szilvási T., Inoue S. (2020). Chem. – Eur. J..

[cit174] Zhang J., Foley B. J., Bhuvanesh N., Zhou J., Janzen D. E., Whited M. T., Ozerov O. V. (2018). Organometallics.

[cit175] Peters J. C., Feldman J. D., Tilley T. D. (1999). J. Am. Chem. Soc..

[cit176] Feldman J. D., Peters J. C., Tilley T. D. (2002). Organometallics.

[cit177] Calimano E., Tilley T. D. (2009). J. Am. Chem. Soc..

[cit178] Feldman J. D., Mitchell G. P., Nolte J.-O., Tilley T. D. (1998). J. Am. Chem. Soc..

[cit179] Sangtrirutnugul P., Tilley T. D. (2008). Organometallics.

[cit180] Hadlington T. J., Szilvási T., Driess M. (2017). Angew. Chem., Int. Ed..

[cit181] Hadlington T. J., Szilvási T., Driess M. (2019). J. Am. Chem. Soc..

[cit182] Hädinger P., Hinz A. (2023). Dalton Trans..

[cit183] Frutos M., Parvin N., Baceiredo A., Madec D., Saffon-Merceron N., Branchadell V., Kato T. (2022). Angew. Chem., Int. Ed..

[cit184] Takahashi S., Frutos M., Baceiredo A., Madec D., Saffon-Merceron N., Branchadell V., Kato T. (2022). Angew. Chem., Int. Ed..

[cit185] Raoufmoghaddam S., Zhou Y.-P., Wang Y., Driess M. (2017). J. Organomet. Chem..

[cit186] Wang W., Inoue S., Yao S., Driess M. (2010). J. Am. Chem. Soc..

[cit187] Wang W., Inoue S., Irran E., Driess M. (2012). Angew. Chem., Int. Ed..

[cit188] Brück A., Gallego D., Wang W., Irran E., Driess M., Hartwig J. F. (2012). Angew. Chem., Int. Ed..

[cit189] Gallego D., Brück A., Irran E., Meier F., Kaupp M., Driess M., Hartwig J. F. (2013). J. Am. Chem. Soc..

[cit190] Yang H., Hinz A., Fan Q., Xie S., Qi X., Huang W., Li Q., Sun H., Li X. (2022). Inorg. Chem..

[cit191] Wang W., Inoue S., Enthaler S., Driess M. (2012). Angew. Chem., Int. Ed..

[cit192] Luecke M.-P., Porwal D., Kostenko A., Zhou Y.-P., Yao S., Keck M., Limberg C., Oestreich M., Driess M. (2017). Dalton Trans..

[cit193] Gallego D., Inoue S., Blom B., Driess M. (2014). Organometallics.

[cit194] Kalra S., Pividori D., Fehn D., Dai C., Dong S., Yao S., Zhu J., Meyer K., Driess M. (2022). Chem. Sci..

[cit195] Metsänen T. T., Gallego D., Szilvási T., Driess M., Oestreich M. (2015). Chem. Sci..

[cit196] Li S., Wang Y., Yang W., Li K., Sun H., Li X., Fuhr O., Fenske D. (2020). Organometallics.

[cit197] He Z., Liu L., De Zwart F. J., Xue X., Ehlers A. W., Yan K., Demeshko S., Van Der Vlugt J. I., De Bruin B., Krogman J. (2022). Inorg. Chem..

[cit198] Xiong Y., Dong S., Yao S., Dai C., Zhu J., Kemper S., Driess M. (2022). Angew. Chem., Int. Ed..

[cit199] Hendi Z., Pandey M. K., Rachuy K., Singh M. K., Herbst-Irmer R., Stalke D., Roesky H. W. (2024). Chem. – Eur. J..

[cit200] Ren H., Zhou Y., Bai Y., Cui C., Driess M. (2017). Chem. – Eur. J..

[cit201] Arevalo R., Pabst T. P., Chirik P. J. (2020). Organometallics.

[cit202] Zhou Y., Mo Z., Luecke M., Driess M. (2018). Chem. – Eur. J..

[cit203] Wang Y., Kostenko A., Yao S., Driess M. (2017). J. Am. Chem. Soc..

[cit204] Kranenburg M., Van Der Burgt Y. E. M., Kamer P. C. J., Van Leeuwen P. W. N. M., Goubitz K., Fraanje J. (1995). Organometallics.

[cit205] Van Leeuwen P. W. N. M., Kamer P. C. J. (2018). Catal. Sci. Technol..

[cit206] Chen X., Wang H., Du S., Driess M., Mo Z. (2022). Angew. Chem., Int. Ed..

[cit207] Kostenko A., Driess M. (2018). J. Am. Chem. Soc..

[cit208] Wang Y., Karni M., Yao S., Apeloig Y., Driess M. (2019). J. Am. Chem. Soc..

[cit209] Lücke M.-P., Yao S., Driess M. (2021). Chem. Sci..

[cit210] Jones C., Rose R. P., Stasch A. (2008). Dalton Trans..

[cit211] Lentz N., Mallet-Ladeira S., Baceiredo A., Kato T., Madec D. (2018). Dalton Trans..

[cit212] Lentz N., Cuevas-Chavez C., Mallet-Ladeira S., Sotiropoulos J.-M., Baceiredo A., Kato T., Madec D. (2021). Inorg. Chem..

[cit213] Cabeza J. A., García-Álvarez P., Pérez-Carreño E., Polo D. (2014). Inorg. Chem..

[cit214] Cabeza J. A., García-Álvarez P., Gobetto R., González-Álvarez L., Nervi C., Pérez-Carreño E., Polo D. (2016). Organometallics.

[cit215] El Ezzi M., Kocsor T.-G., D’Accriscio F., Madec D., Mallet-Ladeira S., Castel A. (2015). Organometallics.

[cit216] Álvarez-Rodríguez L., Cabeza J. A., García-Álvarez P., Pérez-Carreño E., Polo D. (2015). Inorg. Chem..

[cit217] Álvarez-Rodríguez L., Cabeza J. A., Fernández-Colinas J. M., García-Álvarez P., Polo D. (2016). Organometallics.

[cit218] Álvarez-Rodríguez L., Cabeza J. A., García-Álvarez P., Pérez-Carreño E. (2018). Organometallics.

[cit219] Sodreau A., Mallet-Ladeira S., Lachaize S., Miqueu K., Sotiropoulos J.-M., Madec D., Nayral C., Delpech F. (2018). Dalton Trans..

[cit220] Poitiers N. E., Giarrana L., Leszczyńska K. I., Huch V., Zimmer M., Scheschkewitz D. (2020). Angew. Chem., Int. Ed..

[cit221] Yadav R., Goswami B., Simler T., Schoo C., Reichl S., Scheer M., Roesky P. W. (2020). Chem. Commun..

[cit222] Prashanth B., Singh S. (2016). Dalton Trans..

[cit223] Veith M., Müller A., Stahl L., Nötzel M., Jarczyk M., Huch V. (1996). Inorg. Chem..

[cit224] Matioszek D., Saffon N., Sotiropoulos J.-M., Miqueu K., Castel A., Escudié J. (2012). Inorg. Chem..

[cit225] Cabeza J. A., Fernández-Colinas J. M., García-Álvarez P., González-Álvarez L., Pérez-Carreño E. (2019). Dalton Trans..

[cit226] Cabeza J. A., García-Álvarez P., Pérez-Carreño E., Polo D. (2014). Chem. – Eur. J..

[cit227] Knorr M., Hallauer E., Huch V., Veith M., Braunstein P. (1996). Organometallics.

[cit228] Cabeza J. A., Fernández-Colinas J. M., García-Álvarez P., González-Álvarez L., Pérez-Carreño E. (2020). Organometallics.

[cit229] Feng Z., Jiang Y., Ruan H., Zhao Y., Tan G., Zhang L., Wang X. (2019). Dalton Trans..

[cit230] Cabeza J. A., García-Álvarez P., Laglera-Gándara C. J., Pérez-Carreño E. (2020). Dalton Trans..

[cit231] Zhong M., Wei J., Zhang W.-X., Xi Z. (2021). Organometallics.

[cit232] West J. K., Fondong G. L., Noll B. C., Stahl L. (2013). Dalton Trans..

[cit233] Álvarez-Rodríguez L., Cabeza J. A., García-Álvarez P., Polo D. (2015). Organometallics.

[cit234] Parvin N., Mishra B., George A., Neralkar M., Hossain J., Parameswaran P., Hotha S., Khan S. (2020). Chem. Commun..

[cit235] Hossain J., Gopinath J. S., Tothadi S., Parameswaran P., Khan S. (2022). Organometallics.

[cit236] Yadav S., Kumar R., Vipin Raj K., Yadav P., Vanka K., Sen S. S. (2020). Chem. – Asian J..

[cit237] Herrmann W. A., Denk M., Behm J., Scherer W., Klingan F., Bock H., Solouki B., Wagner M. (1992). Angew. Chem., Int. Ed. Engl..

[cit238] Kühl O., Lönnecke P., Heinicke J. (2003). Inorg. Chem..

[cit239] Ullah F., Kühl O., Bajor G., Veszprémi T., Jones P. G., Heinicke J. (2009). Eur. J. Inorg. Chem..

[cit240] Sharma M. K., Sinhababu S., Yadav D., Mukherjee G., Rajaraman G., Nagendran S. (2018). Chem. – Asian J..

[cit241] Brugos J., Cabeza J. A., García-Álvarez P., Pérez-Carreño E. (2018). Organometallics.

[cit242] Cabeza J. A., García-Álvarez P., Polo D. (2011). Inorg. Chem..

[cit243] Cabeza J. A., García-Álvarez P., Polo D. (2012). Inorg. Chem..

[cit244] Buil M. L., Cabeza J. A., Esteruelas M. A., Izquierdo S., Laglera-Gándara C. J., Nicasio A. I., Oñate E. (2021). Inorg. Chem..

[cit245] Kazakov G. G., Druzhkov N. O., Baranov E. V., Piskunov A. V., Cherkasov V. K. (2021). J. Organomet. Chem..

[cit246] Karwasara S., Siwatch R. K., Jha C. K., Nagendran S. (2015). Organometallics.

[cit247] Yadav D., Singh D., Sarkar D., Sinhababu S., Sharma M. K., Nagendran S. (2019). J. Organomet. Chem..

[cit248] Álvarez-Rodríguez L., Brugos J., Cabeza J. A., García-Álvarez P., Pérez-Carreño E., Polo D. (2017). Chem. Commun..

[cit249] Fernández-Buenestado M., Somerville R. J., López-Serrano J., Campos J. (2023). Chem. Commun..

[cit250] Gendy C., Mansikkamäki A., Valjus J., Heidebrecht J., Hui P. C., Bernard G. M., Tuononen H. M., Wasylishen R. E., Michaelis V. K., Roesler R. (2019). Angew. Chem., Int. Ed..

[cit251] Álvarez-Rodríguez L., Brugos J., Cabeza J. A., García-Álvarez P., Pérez-Carreño E. (2017). Chem. – Eur. J..

[cit252] Sharma M. K., Singh D., Mahawar P., Yadav R., Nagendran S. (2018). Dalton Trans..

[cit253] Dias H. V. R., Wang Z. (2000). Inorg. Chem..

[cit254] Ayers A. E., Dias H. V. R. (2002). Inorg. Chem..

[cit255] Dias H. V. R., Ayers A. E. (2002). Polyhedron.

[cit256] Yadav D., Kumar Siwatch R., Sinhababu S., Karwasara S., Singh D., Rajaraman G., Nagendran S. (2015). Inorg. Chem..

[cit257] Bestgen S., Rees N. H., Goicoechea J. M. (2018). Organometallics.

[cit258] Sinhababu S., Yadav D., Karwasara S., Sharma M. K., Mukherjee G., Rajaraman G., Nagendran S. (2016). Angew. Chem., Int. Ed..

[cit259] Sinhababu S., Sharma M. K., Mahawar P., Kaur S., Singh V. K., Paliwal A., Yadav D., Kashyap H. K., Nagendran S. (2019). Dalton Trans..

[cit260] Saur I., Rima G., Miqueu K., Gornitzka H., Barrau J. (2003). J. Organomet. Chem..

[cit261] Saur I., Garcia Alonso S., Gornitzka H., Lemierre V., Chrostowska A., Barrau J. (2005). Organometallics.

[cit262] Pineda L. W., Jancik V., Colunga-Valladares J. F., Roesky H. W., Hofmeister A., Magull J. (2006). Organometallics.

[cit263] Jana A., Samuel P. P., Roesky H. W., Schulzke C. (2010). J. Fluorine Chem..

[cit264] Leung W.-P., Chiu W.-K., Mak T. C. W. (2014). Organometallics.

[cit265] Nie P., Li Y., Yu Q., Li B., Zhu H., Wen T. (2017). Eur. J. Inorg. Chem..

[cit266] Arauzo A., Cabeza J. A., Fernández I., García-Álvarez P., García-Rubio I., Laglera-Gándara C. J. (2021). Chem. – Eur. J..

[cit267] Cabeza J. A., Fernández-Colinas J. M., García-Álvarez J., García-Álvarez P., Laglera-Gándara C. J., Ramos-Martín M. (2022). Chem. – Eur. J..

[cit268] Cabeza J. A., García-Álvarez P., Laglera-Gándara C. J., Pérez-Carreño E. (2020). Chem. Commun..

[cit269] Bazinet P., Yap G. P. A., Richeson D. S. (2001). J. Am. Chem. Soc..

[cit270] Cabeza J. A., Fernández I., García-Álvarez P., Laglera-Gándara C. J. (2019). Dalton Trans..

[cit271] Leung W.-P., So C.-W., Chong K.-H., Kan K.-W., Chan H.-S., Mak T. C. W. (2006). Organometallics.

[cit272] Arii H., Nakadate F., Mochida K. (2009). Organometallics.

[cit273] Ferro L., Hitchcock P. B., Coles M. P., Fulton J. R. (2012). Inorg. Chem..

[cit274] Zhao N., Zhang J., Yang Y., Zhu H., Li Y., Fu G. (2012). Inorg. Chem..

[cit275] Zhao N., Zhang J., Yang Y., Chen G., Zhu H., Roesky H. W. (2013). Organometallics.

[cit276] Hlina J., Baumgartner J., Marschner C., Zark P., Müller T. (2013). Organometallics.

[cit277] Lee V. Y., Sakai R., Takanashi K., Gapurenko O. A., Minyaev R. M., Gornitzka H., Sekiguchi A. (2021). Angew. Chem., Int. Ed..

[cit278] Dong Z., Bedbur K., Schmidtmann M., Müller T. (2018). J. Am. Chem. Soc..

[cit279] Dong Z., Reinhold C. R. W., Schmidtmann M., Müller T. (2016). Angew. Chem., Int. Ed..

[cit280] Wang L., Zhen G., Li Y., Kira M., Yan L., Chang X.-Y., Huang L., Li Z. (2022). Nat. Commun..

[cit281] Lee V. Y. (2022). Eur. J. Inorg. Chem..

[cit282] Jutzi P., Steiner W. (1976). Angew. Chem., Int. Ed. Engl..

[cit283] Nakata N., Aoki S., Lee V. Y., Sekiguchi A. (2015). Organometallics.

[cit284] Jutzi P., Steiner W., König E., Huttner G., Frank A., Schubert U. (1978). Chem. Ber..

[cit285] Jutzi P., Steiner W. (1976). Chem. Ber..

[cit286] Ueno K., Yamaguchi K., Ogino H. (1999). Organometallics.

[cit287] Koe J. R., Tobita H., Suzuki T., Ogino H. (1992). Organometallics.

[cit288] Tokitoh N., Manmaru K., Okazaki R. (1994). Organometallics.

[cit289] Filippou A. C., Weidemann N., Philippopoulos A. I., Schnakenburg G. (2006). Angew. Chem., Int. Ed..

[cit290] Shinohara A., McBee J., Tilley T. D. (2009). Inorg. Chem..

[cit291] Filippou A. C., Stumpf K. W., Chernov O., Schnakenburg G. (2012). Organometallics.

[cit292] Fukuda T., Hashimoto H., Tobita H. (2013). Chem. Commun..

[cit293] Filippou A. C., Chakraborty U., Schnakenburg G. (2013). Chem. – Eur. J..

[cit294] Hitchcock P. B., Lappert M. F., Thomas S. A., Thorne A. J., Carty A. J., Taylor N. J. (1986). J. Organomet. Chem..

[cit295] Ochiai T., Franz D., Wu X.-N., Inoue S. (2015). Dalton Trans..

[cit296] Lipke M. C., Neumeyer F., Tilley T. D. (2014). J. Am. Chem. Soc..

[cit297] Smart K. A., Mothes-Martin E., Vendier L., Perutz R. N., Grellier M., Sabo-Etienne S. (2015). Organometallics.

[cit298] Dhungana T. P., Hashimoto H., Tobita H. (2017). Dalton Trans..

[cit299] Keil P. M., Soyemi A., Weisser K., Szilvási T., Limberg C., Hadlington T. J. (2023). Angew. Chem..

[cit300] Hawkins S. M., Hitchcock P. B., Lappert M. F., Rai A. K. (1986). J. Chem. Soc., Chem. Commun..

[cit301] Auer M., Bolten J., Eichele K., Schubert H., Sindlinger C. P., Wesemann L. (2023). Chem. Sci..

[cit302] Bajo S., Alférez M. G., Alcaide M. M., López-Serrano J., Campos J. (2020). Chem. – Eur. J..

[cit303] Schnepf A. (2006). Z. Anorg. Allg. Chem..

[cit304] Rödl C., Hierlmeier G., Wolf R. (2022). Chem. Commun..

[cit305] Litz K. E., Kampf J. W., Banaszak Holl M. M. (1998). J. Am. Chem. Soc..

[cit306] Litz K. E., Bender J. E., Kampf J. W., Holl M. M. B. (1997). Angew. Chem., Int. Ed. Engl..

[cit307] Bender J. E., Shusterman A. J., Banaszak Holl M. M., Kampf J. W. (1999). Organometallics.

[cit308] Liao W.-H., Ho P.-Y., Su M.-D. (2013). Inorg. Chem..

[cit309] Hupp F., Ma M., Kroll F., Jimenez-Halla J. O. C., Dewhurst R. D., Radacki K., Stasch A., Jones C., Braunschweig H. (2014). Chem. – Eur. J..

[cit310] Watanabe T., Kasai Y., Tobita H. (2019). Chem. – Eur. J..

[cit311] Emerich B. M., Moore C. E., Fox B. J., Rheingold A. L., Figueroa J. S. (2011). Organometallics.

[cit312] Ortuño M. A., Conejero S., Lledós A. (2013). Beilstein J. Org. Chem..

[cit313] Barnett B. R., Moore C. E., Chandrasekaran P., Sproules S., Rheingold A. L., DeBeer S., Figueroa J. S. (2015). Chem. Sci..

[cit314] Bauer J., Braunschweig H., Dewhurst R. D. (2012). Chem. Rev..

[cit315] Schulz A., Kalkuhl T. L., Keil P. M., Hadlington T. J. (2023). Angew. Chem., Int. Ed..

[cit316] Keil P. M., Hadlington T. J. (2022). Angew. Chem., Int. Ed..

[cit317] Keil P. M., Szilvási T., Hadlington T. J. (2021). Chem. Sci..

[cit318] Theulier C. A., Bajo S., López-Serrano J., Campos J. (2024). Chem. – Eur. J..

[cit319] Bajo S., Alcaide M. M., López-Serrano J., Campos J. (2020). Chem. – Eur. J..

[cit320] Zabula A. V., Hahn F. E., Pape T., Hepp A. (2007). Organometallics.

[cit321] Hahn F. E., Zabula A. V., Pape T., Hepp A. (2008). Z. Anorg. Allg. Chem..

[cit322] Chen M., Zhang Z., Qiao Z., Zhao L., Mo Z. (2023). Angew. Chem..

[cit323] Zhou Y., Richeson D. S. (1996). J. Am. Chem. Soc..

[cit324] Aubrecht K. B., Hillmyer M. A., Tolman W. B. (2002). Macromolecules.

[cit325] Foley S. R., Zhou Y., Yap G. P. A., Richeson D. S. (2000). Inorg. Chem..

[cit326] Green S. P., Jones C., Lippert K.-A., Mills D. P., Stasch A. (2006). Inorg. Chem..

[cit327] Chlupatý T., Padělková Z., DeProft F., Willem R., Růžička A. (2012). Organometallics.

[cit328] Gyton M. R., Leverett A. R., Cole M. L., McKay A. I. (2020). Dalton Trans..

[cit329] Zhu Q., Fettinger J. C., Power P. P. (2021). Dalton Trans..

[cit330] Zhao X., Szilvási T., Hanusch F., Kelly J. A., Fujimori S., Inoue S. (2022). Angew. Chem., Int. Ed..

[cit331] Veith M., Stahl L., Huch V. (1989). Inorg. Chem..

[cit332] Veith M., Stahl L., Huch V. (1990). J. Chem. Soc., Chem. Commun..

[cit333] Veith M., Stahl L. (1993). Angew. Chem., Int. Ed. Engl..

[cit334] Day B. M., Dyer P. W., Coles M. P. (2012). Dalton Trans..

[cit335] Kircher P., Huttner G., Heinze K., Schiemenz B., Zsolnai L., Büchner M., Driess A. (1998). Eur. J. Inorg. Chem..

[cit336] Agustin D., Rima G., Gornitzka H., Barrau J. (2000). Eur. J. Inorg. Chem..

[cit337] Wang S., Li H.-J., Kuo T.-S., Shen L.-C., Liu H.-J. (2021). Organometallics.

[cit338] Mansell S. M., Russell C. A., Wass D. F. (2008). Inorg. Chem..

[cit339] Mansell S. M., Herber R. H., Nowik I., Ross D. H., Russell C. A., Wass D. F. (2011). Inorg. Chem..

[cit340] Kireenko M. M., Zaitsev K. V., Oprunenko Y. F., Churakov A. V., Tafeenko V. A., Karlov S. S., Zaitseva G. S. (2013). Dalton Trans..

[cit341] Dias H. V. R., Wang X., Diyabalanage H. V. K. (2005). Inorg. Chem..

[cit342] Leung W., Kan K., Chan Y., Mak T. C. W. (2014). Eur. J. Inorg. Chem..

[cit343] Jana A., Sarish S. P., Roesky H. W., Schulzke C., Samuel P. P. (2010). Chem. Commun..

[cit344] Jana A., Roesky H. W., Schulzke C., Samuel P. P. (2010). Inorg. Chem..

[cit345] Jana A., Azhakar R., Roesky H. W., Objartel I., Stalke D. (2011). Z. Anorg. Allg. Chem..

[cit346] Cabeza J. A., Fernández I., García-Álvarez P., García-Soriano R., Laglera-Gándara C. J., Toral R. (2021). Dalton Trans..

[cit347] Arp H., Baumgartner J., Marschner C., Zark P., Müller T. (2012). J. Am. Chem. Soc..

[cit348] Zhao H., Li J., Xiao X., Kira M., Li Z., Müller T. (2018). Chem. – Eur. J..

[cit349] Al-Rafia S. M. I., Malcolm A. C., Liew S. K., Ferguson M. J., Rivard E. (2011). J. Am. Chem. Soc..

[cit350] Harris D. H., Lappert M. F. (1974). J. Chem. Soc., Chem. Commun..

[cit351] Hitchcock P. B., Lappert M. F., Misra M. C. (1985). J. Chem. Soc., Chem. Commun..

[cit352] Al-Allaf T. A. K., Eaborn C., Hitchcock P. B., Lappert M. F., Pidcock A. (1985). J. Chem. Soc., Chem. Commun..

[cit353] Campbell G. K., Hitchcock P. B., Lappert M. F., Misra M. C. (1985). J. Organomet. Chem..

[cit354] Whittal R. M., Ferguson G., Gallagher J. F., Piers W. E. (1991). J. Am. Chem. Soc..

[cit355] Bareš J., Richard P., Meunier P., Pirio N., Padělková Z., Černošek Z., Císařová I., Růžička A. (2009). Organometallics.

[cit356] Maudrich J., Widemann M., Diab F., Kern R. H., Sirsch P., Sindlinger C. P., Schubert H., Wesemann L. (2019). Chem. – Eur. J..

[cit357] Eichler B. E., Power P. P. (2000). J. Am. Chem. Soc..

[cit358] Widemann M., Jeggle S., Auer M., Eichele K., Schubert H., Sindlinger C. P., Wesemann L. (2022). Chem. Sci..

[cit359] Ellis S. L., Hitchcock P. B., Holmes S. A., Lappert M. F., Slade M. J. (1993). J. Organomet. Chem..

[cit360] Weidenbruch M., Stilter A., Schlaefke J., Peters K., Schnering H. G. V. (1995). J. Organomet. Chem..

[cit361] Weidenbruch M., Stilter A., Peters K., Von Schnering H. G. (1996). Z. Anorg. Allg. Chem..

[cit362] Zhu Q., Fettinger J. C., Vasko P., Power P. P. (2022). Organometallics.

[cit363] Weidenbruch M., Stilter A., Saak W., Peters K., Von Schnering H. G. (1998). J. Organomet. Chem..

[cit364] Filippou A. C., Ghana P., Chakraborty U., Schnakenburg G. (2013). J. Am. Chem. Soc..

[cit365] Weidenbruch M., Stilter A., Peters K., Schnering H. G. V. (1996). Chem. Ber..

[cit366] Schneider J. J., Czap N., Bläser D., Boese R. (1999). J. Am. Chem. Soc..

[cit367] Schneider J. J., Czap N., Bläser D., Boese R., Ensling J., Gütlich P., Janiak C. (2000). Chem. – Eur. J..

[cit368] Hayes P. G., Gribble C. W., Waterman R., Tilley T. D. (2009). J. Am. Chem. Soc..

[cit369] Smith P. W., Handford R. C., Tilley T. D. (2019). Organometallics.

[cit370] Handford R. C., Nesbit M. A., Smith P. W., Britt R. D., Tilley T. D. (2022). J. Am. Chem. Soc..

[cit371] Schneider J. J., Hagen J., Bläser D., Boese R., Krüger C. (1997). Angew. Chem., Int. Ed. Engl..

[cit372] Schneider J. J., Czap N., Bläser D., Boese R. (1999). J. Organomet. Chem..

[cit373] Widemann M., Eichele K., Schubert H., Sindlinger C. P., Klenner S., Pöttgen R., Wesemann L. (2021). Angew. Chem., Int. Ed..

[cit374] Pluta C., Pörschke K. R., Mynott R., Betz P., Krüger C. (1991). Chem. Ber..

[cit375] Krause J., Pluta C., Pörschke K.-R., Goddard R. (1993). J. Chem. Soc., Chem. Commun..

[cit376] Arp H., Marschner C., Baumgartner J., Zark P., Müller T. (2013). J. Am. Chem. Soc..

[cit377] Krebs K. M., Freitag S., Schubert H., Gerke B., Pöttgen R., Wesemann L. (2015). Chem. – Eur. J..

[cit378] Krebs K. M., Freitag S., Maudrich J.-J., Schubert H., Sirsch P., Wesemann L. (2018). Dalton Trans..

[cit379] Braunschweig H., Celik M. A., Dewhurst R. D., Heid M., Hupp F., Sen S. S. (2015). Chem. Sci..

[cit380] Klett J., Klinkhammer K. W., Niemeyer M. (1999). Chem. – Eur. J..

[cit381] Zabula A. V., Pape T., Hepp A., Hahn F. E. (2008). Organometallics.

[cit382] Zabula A. V., Pape T., Hepp A., Hahn F. E. (2008). Dalton Trans..

[cit383] Hahn F. E., Zabula A. V., Pape T., Hepp A., Tonner R., Haunschild R., Frenking G. (2008). Chem. – Eur. J..

[cit384] Henoch J., Auch A., Diab F., Eichele K., Schubert H., Sirsch P., Block T., Pöttgen R., Wesemann L. (2018). Inorg. Chem..

[cit385] Henning J., Wesemann L. (2012). Angew. Chem., Int. Ed..

[cit386] Henning J., Eichele K., Fink R. F., Wesemann L. (2014). Organometallics.

[cit387] Schneider J., Henning J., Edrich J., Schubert H., Wesemann L. (2015). Inorg. Chem..

[cit388] Weiß S., Widemann M., Eichele K., Schubert H., Wesemann L. (2021). Dalton Trans..

[cit389] Jones C., Bonyhady S. J., Holzmann N., Frenking G., Stasch A. (2011). Inorg. Chem..

[cit390] Braunschweig H., Damme A., Dewhurst R. D., Hupp F., Jimenez-Halla J. O. C., Radacki K. (2012). Chem. Commun..

[cit391] Takemoto S., Yoshii K., Yamano T., Tsurusaki A., Matsuzaka H. (2021). Chem. Commun..

[cit392] Kelly J. A., Streitferdt V., Dimitrova M., Westermair F. F., Gschwind R. M., Berger R. J. F., Wolf R. (2022). J. Am. Chem. Soc..

[cit393] Keil P. M., Ezendu S., Schulz A., Kubisz M., Szilvási T., Hadlington T. J. (2024). J. Am. Chem. Soc..

[cit394] Fürstner A. (2013). Angew. Chem., Int. Ed..

[cit395] Ehrhorn H., Tamm M. (2019). Chem. – Eur. J..

[cit396] Jutzi P. (1975). Angew. Chem., Int. Ed. Engl..

[cit397] Fischer E. O., Kreis G., Kreiter C. G., Mülle J., Huttner G., Lorenz H. (1973). Angew. Chem..

[cit398] Simons R. S., Power P. P. (1996). J. Am. Chem. Soc..

[cit399] Filippou A. C., Portius P., Philippopoulos A. I., Rohde H. (2003). Angew. Chem., Int. Ed..

[cit400] Filippou A. C., Rohde H., Schnakenburg G. (2004). Angew. Chem., Int. Ed..

[cit401] Hu C., Wang X.-F., Wei R., Hu C., Ruiz D. A., Chang X.-Y., Liu L. L. (2022). Chemistry.

[cit402] Pu L., Twamley B., Haubrich S. T., Olmstead M. M., Mork B. V., Simons R. S., Power P. P. (2000). J. Am. Chem. Soc..

[cit403] Eichler B. E., Phillips A. D., Haubrich S. T., Mork B. V., Power P. P. (2002). Organometallics.

[cit404] Pu L., Power P. P., Boltes I., Herbst-Irmer R. (2000). Organometallics.

[cit405] Filippou A. C., Baars B., Chernov O., Lebedev Y. N., Schnakenburg G. (2014). Angew. Chem., Int. Ed..

[cit406] Power P. P. (1998). J. Chem. Soc., Dalton Trans..

[cit407] Ghana P., Arz M. I., Schnakenburg G., Straßmann M., Filippou A. C. (2018). Organometallics.

[cit408] Ghana P., Arz M. I., Chakraborty U., Schnakenburg G., Filippou A. C. (2018). J. Am. Chem. Soc..

[cit409] Ghana P., Rump J., Schnakenburg G., Arz M. I., Filippou A. C. (2021). J. Am. Chem. Soc..

[cit410] Ebner F., Greb L. (2021). Chemistry.

[cit411] Shan C., Dong S., Yao S., Zhu J., Driess M. (2023). J. Am. Chem. Soc..

[cit412] Filippou A. C., Hoffmann D., Schnakenburg G. (2017). Chem. Sci..

[cit413] Ouellette E. T., Carpentier A., Joseph Brackbill I., Lohrey T. D., Douair I., Maron L., Bergman R. G., Arnold J. (2021). Dalton Trans..

[cit414] Chan L. Y. Y., Dean W. K., Graham W. A. G. (1977). Inorg. Chem..

[cit415] Filippou A. C., Philippopoulos A. I., Portius P., Neumann D. U. (2000). Angew. Chem., Int. Ed..

[cit416] Filippou A. C., Portius P., Philippopoulos A. I. (2002). Organometallics.

[cit417] Hashimoto H., Fukuda T., Tobita H., Ray M., Sakaki S. (2012). Angew. Chem., Int. Ed..

[cit418] Dhungana T. P., Hashimoto H., Ray M., Tobita H. (2020). Organometallics.

[cit419] Hicks J., Hadlington T. J., Schenk C., Li J., Jones C. (2013). Organometallics.

[cit420] Queen J. D., Phung A. C., Caputo C. A., Fettinger J. C., Power P. P. (2020). J. Am. Chem. Soc..

[cit421] Lebedev Y. N., Das U., Schnakenburg G., Filippou A. C. (2017). Organometallics.

[cit422] Filippou A. C., Philippopoulos A. I., Portius P., Schnakenburg G. (2004). Organometallics.

[cit423] Filippou A. C., Schnakenburg G., Philippopoulos A. I., Weidemann N. (2005). Angew. Chem., Int. Ed..

[cit424] Filippou A. C., Barandov A., Schnakenburg G., Lewall B., van Gastel M., Marchanka A. (2012). Angew. Chem., Int. Ed..

[cit425] Inomata K., Watanabe T., Tobita H. (2014). J. Am. Chem. Soc..

[cit426] Inomata K., Watanabe T., Miyazaki Y., Tobita H. (2015). J. Am. Chem. Soc..

[cit427] Lei H., Guo J.-D., Fettinger J. C., Nagase S., Power P. P. (2011). Organometallics.

[cit428] Phung A. C., Fettinger J. C., Power P. P. (2021). Organometallics.

[cit429] Keil P. M., Hadlington T. J. (2022). Chem. Commun..

[cit430] Juckel M. M., Hicks J., Jiang D., Zhao L., Frenking G., Jones C. (2017). Chem. Commun..

[cit431] Filippou A. C., Philippopoulos A. I., Schnakenburg G. (2003). Organometallics.

[cit432] Stewart M. A., Moore C. E., Ditri T. B., Labios L. A., Rheingold A. L., Figueroa J. S. (2011). Chem. Commun..

[cit433] Liu H.-J., Guihaumé J., Davin T., Raynaud C., Eisenstein O., Tilley T. D. (2014). J. Am. Chem. Soc..

[cit434] Auer M., Zwettler K., Eichele K., Schubert H., Sindlinger C. P., Wesemann L. (2023). Angew. Chem., Int. Ed..

[cit435] Maurer L. R., Rump J., Filippou A. C. (2023). Inorganics.

[cit436] Seth M., Faegri K., Schwerdtfeger P. (1998). Angew. Chem., Int. Ed..

[cit437] Thayer J. S. (2005). J. Chem. Educ..

[cit438] Filippou A. C., Weidemann N., Schnakenburg G., Rohde H., Philippopoulos A. I. (2004). Angew. Chem., Int. Ed..

[cit439] Filippou A. C., Weidemann N., Schnakenburg G. (2008). Angew. Chem., Int. Ed..

[cit440] Zhu Q., Fettinger J. C., Vasko P., Power P. P. (2020). Organometallics.

